# Proteomic Analysis of Lipid Rafts from RBL-2H3 Mast Cells

**DOI:** 10.3390/ijms20163904

**Published:** 2019-08-11

**Authors:** Edismauro Garcia Freitas Filho, Luiz Augusto Marin Jaca, Lilian Cristiane Baeza, Célia Maria de Almeida Soares, Clayton Luiz Borges, Constance Oliver, Maria Célia Jamur

**Affiliations:** 1 Department of Cell and Molecular Biology and Pathogenic Bioagents, Ribeirão Preto Medical School, University of São Paulo, Av. Bandeirantes 3900, Ribeirão Preto, SP, 14049-900, Brazil; 2 Molecular Biology Laboratory, Biological Science Institute, Federal University of Goiás, Samambaia Campus II, ICB2, room 206, Goiânia, GO, 74690-900, Brazil

**Keywords:** lipid rafts, membrane proteins, protein localization, regulated secretion, signaling pathway, proteome, mast cells

## Abstract

Lipid rafts are highly ordered membrane microdomains enriched in cholesterol, glycosphingolipids, and certain proteins. They are involved in the regulation of cellular processes in diverse cell types, including mast cells (MCs). The MC lipid raft protein composition was assessed using qualitative mass spectrometric characterization of the proteome from detergent-resistant membrane fractions from RBL-2H3 MCs. Using two different post-isolation treatment methods, a total of 949 lipid raft associated proteins were identified. The majority of these MC lipid raft proteins had already been described in the RaftProtV2 database and are among highest cited/experimentally validated lipid raft proteins. Additionally, more than half of the identified proteins had lipid modifications and/or transmembrane domains. Classification of identified proteins into functional categories showed that the proteins were associated with cellular membrane compartments, and with some biological and molecular functions, such as regulation, localization, binding, catalytic activity, and response to stimulus. Furthermore, functional enrichment analysis demonstrated an intimate involvement of identified proteins with various aspects of MC biological processes, especially those related to regulated secretion, organization/stabilization of macromolecules complexes, and signal transduction. This study represents the first comprehensive proteomic profile of MC lipid rafts and provides additional information to elucidate immunoregulatory functions coordinated by raft proteins in MCs.

## 1. Introduction

Lipid rafts are dynamic ordered nanoscale assemblies in the plasma membrane and other intracellular membranes, and are enriched in cholesterol and glycosphingolipids [1,2]. They are characterized by the presence of proteins with lipid modifications, as well as proteins involved in signal transduction. Due to their composition, they are resistant to solubilization in nonionic mild detergents [1,3]. Lipid raft components can diffuse laterally in the plasma membrane, thus lipid raft microdomains have the ability to associate and dissociate on a subsecond timescale, and vary in stability, size, shape, lifetime, and molecular composition [3,4,5]. The compartmentalization of molecules into lipid rafts provides a favorable environment to facilitate interactions among the raft components. Therefore, lipid rafts act as platforms to segregate lipids, receptors, adaptors, kinases, scaffolding proteins, and cytoskeletal apparatus that trigger complex events and coordinate diverse biological processes [2,6,7,8,9,10].

Since the total protein content of lipid rafts can be extensive, these microdomains are an attractive target for mass-spectrometry (MS)-based proteomics. Proteomic strategies have been applied to lipid rafts from a variety of tissue and cell types, including rat brain [11], Hella cells [12], ovarian cancer cells [13], and immune cells, such as neutrophils [14,15], monocytes [16], and macrophages [17], as well as lymphocytes T [18,19], B [20], and natural killer cells [21], with the aim of better identifying the proteins present in lipid rafts [22,23,24,25]. Functional proteomic analysis of lipid raft proteins examines the relationship between individual proteins and clusters them based on characteristics such as structure, localization, binding partners, and post translational modification, which has led to the elucidation of novel molecular pathways and biological events coordinated by these membrane microdomains [23,25,26,27].

Mast cells (MCs) are multifunctional immune cells that, in addition to their well-established role in allergic and anaphylactic reactions, are implicated in innate and adaptive immunity, and in inflammation among other physiological and pathological processes [28,29,30]. Lipid rafts modulate many important biological processes related to these MC functions, such as degranulation, endocytosis, play a role in MC development and recruitment, and contribute to the preservation of MC structure and organization [6,31,32,33]. However, there is no MC lipid raft proteome available. Only a few studies report on the whole MC plasma membrane composition, using MS for lipid characterization [34] or protein identification using MALDI-TOF (matrix-assisted laser desorption/ionization and time-of-flight) or LC-MS/MS (liquid chromatography-mass spectrometry) [35,36,37]. Moreover, only a limited number of proteins have been annotated and a non-detailed functional analysis was performed.

The present study was undertaken to investigate the qualitative proteomic profile of MC lipid rafts using the rat mucosal MC line RBL-2H3. While the investigation of lipid rafts in MCs has largely been done using RBL-2H3 MCs [38], there is no proteomic profile of lipid rafts from RBL-2H3 MCs or any other MC. Although controversy exists over the exact character of RBL-2H3 MCs [39,40], they are still a widely accepted model for functional studies of MC dynamics. Since the RBL-2H3 MC line was first identified in 1973 [41], and later cloned and characterized [42], it has become one the most commonly used models to study MC function [40]. RBL-2H3 MCs provide many advantages over primary MCs. They may be grown in large amounts in culture and can easily be genetically manipulated. RBL-2H3 MCs have also been used to study signaling pathways following FcεRI (high-affinity IgE receptor) activation and MC regulated exocytosis, events in which lipid rafts are involved. More recently, RBL-2H3 MCs have also been used as a model for studies focused on the detection of allergens, diagnosis of allergic sensitization, and vaccine safety studies [40]. Even considering the problem of MC heterogeneity, the findings provided by RBL-2H3 MCs have substantially contributed to a global understanding of MC function. The results of the present investigation show that the methods applied here were efficient in identifying lipid raft proteins in MCs and these raft microdomains are involved in the regulated secretion, organization, and stabilization of macromolecular complexes, as well as signaling transduction pathways important to MC biological functions. This qualitative proteomic data should provide a more complete understanding of lipid rafts in MC biology.

## 2. Results

### 2.1. Obtention of Lipid Rafts from RBL-2H3 Mast Cells

**Figure 1:**
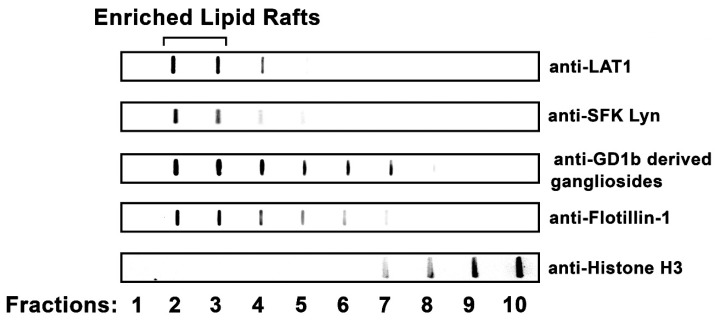
Identification of RBL-2H3 mast cell lipid rafts. Lysates of RBL-2H3 MCs were fractionated using sucrose density gradient ultracentrifugation, and fractions were immunoblotted using antibodies against lipid raft markers: LAT1, SFK Lyn, rodent MC-specific GD1b-derived gangliosides, and Flotillin-1. Proteins and lipids associated with lipid rafts were concentrated in Fractions 2 and 3. Fractions were also immunoblotted with anti-Histone H3, a nuclear protein. Data representative of three independent experiments is shown.

Lipid rafts were isolated from RBL-2H3 MCs using discontinuous sucrose-density gradient ultracentrifugation. LAT1 (linker for activation of T-cells 1), a lipid-raft-specific protein in immune cells [43,44], was used as a marker for the lipid rafts. Using immunoblotting, LAT1 was highly enriched in Fractions 2 and 3. In addition, the SFK (Src family kinase) Lyn, and the rodent MC-specific GD1b-derived gangliosides, both well-characterized MC lipid raft components [45,46], were also enriched in Fractions 2 and 3 (Figure 1). Additionally, Flotillin-1, a widely used marker of lipid rafts [8], was also concentrated in Fractions 2 and 3. Thus, Fractions 2 and 3 represent the lipid raft fractions in these preparations. In contrast, Histone H3, a nuclear protein, was concentrated in Fractions 9 and 10.

### 2.2. Identification of Mast Cell Lipid Raft Proteins Using Nano-UPLC-MS^E^

The lipid raft fractions (Fractions 2 and 3) obtained from three independent experiments were pooled. The resulting sample was divided and used for two different post-isolation treatment methods, Method I (MetI) and Method II (MetII). MetI eliminated the sucrose from the enriched lipid raft fractions prior to MS analysis. MetII was harsher than MetI and involved mixing the sample with OGP (octyl β-glucopyranoside) detergent followed by a final methanol-chloroform extraction. The solubilized proteins obtained from the lipid raft fractions after the post-isolation treatments were submitted to nano-UPLC-MS^E^ analysis. The samples were run in three technical replicates and only proteins identified in two out of the three replicates were considered for further analysis. The obtained UPLC-MS^E^ protein data generated by the PLGS was processed to verify the quality of the proteomic analysis (Figure S1: Dynamic range of the proteomic analysis). For reliable protein identification, a reverse sequence database of Rattus norvegicus was used to calculate the false rate. The false positive rates of proteins obtained from MetI and MetII were, respectively, 1.04% and 0.36%. Supplementary Figure S1 depicts the results obtained from an analysis of the dynamic range indicating that a 3 log difference in abundance and a good distribution of both high and low concentrations of the proteins were obtained with both methods. This approach ensured the selection of highly representative proteins.

**Figure 2:**
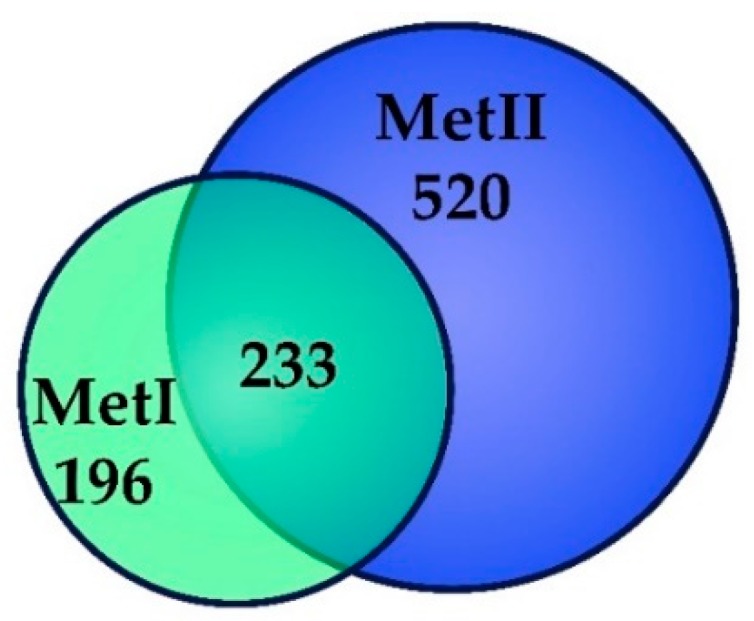
Proteomic identification of lipid raft enriched fractions of RBL-2H3 mast cells. MC lipid-raft-enriched Fractions 2 and 3 were used for Nano-UPLC-MS^E^ analysis. A Venn diagram depicting the number of overlapping and unique proteins present in MC lipid rafts processed using Method I (MetI) and Method II (MetII) is shown.

After processing, according to the criteria stated in Section 5.6, 429 proteins were identified in MetI (Table S1: Detailed annotation of proteins identified in Method I), and 753 in MetII (Table S2: Detailed annotation of proteins identified in Method II); 196 proteins were exclusively identified in MetI and 520 proteins were exclusively identified in MetII, and 233 proteins were common between the methods. In total, 949 proteins were identified (Figure 2) (Table S3: Detailed annotation of proteins identified in Methods I and II).

### 2.3. Characterization of Mast Cell Lipid Raft Proteins

The 949 identified proteins were then analyzed to determine whether they had been previously reported as lipid raft proteins or had characteristic modifications of lipid raft proteins. Of the 949 identified proteins, 855 (≈90%; 855/949) were found in the RaftProtV2 database as previously reported lipid raft proteins [26], while 94 of the proteins had not been previously annotated (Table S4: Mast cell lipid raft proteins absent from RaftProtV2 database, and Figure S2: Immuno-blot analysis of the β-subunit of FcεRI from RBL-2H3 MC lipid rafts). Of the previously annotated proteins, 172 were unique to MetI and 454 to MetII, thus indicating that MetII was more efficient in extracting lipid raft proteins (Table S5: Mast cell lipid raft proteins analyzed by RafProtV2 database). A total of 570 (≈67%; 570/855) of these proteins were classified as high-confidence lipid raft proteins based on experimental evidence according to the RaftProtV2 database, confirming that these proteins were indeed lipid raft proteins. The proteins with the highest number of citations supported by experimental evidence are listed in Table 1.

**Figure 3:**
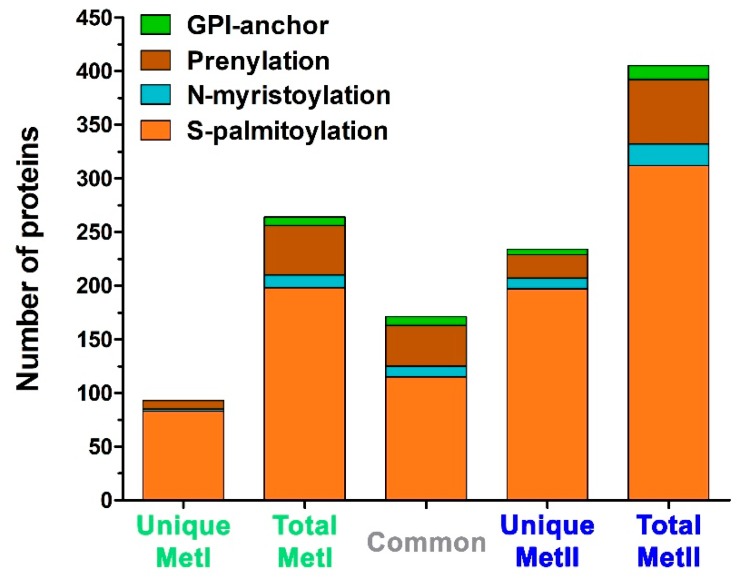
Proteins with an annotated lipid modification identified in mast cell lipid rafts isolated using Method I or Method II. Databases used: PhosphoSitePlus, SwissPalm, MYRbase, PRENbase, and PredGPI.

Lipid modifications are one of the characteristics of lipid-raft-associated proteins [1]. Therefore, all the identified proteins were examined for lipid modifications using bioinformatic analysis. Almost half of the identified proteins (46.8%; 444/949) had at least one lipid modification: S-palmitoylation, isoprenylation, N-myristoylation, or GPI (glycophosphatidylinositol) anchor. There was basically no difference in the percentage of proteins with lipid modifications extracted with either MetI (50.3%; 216/429) or MetII (48%; 361/753). However, MetII had a higher number of proteins with lipid modification in all categories analyzed (Figure 3).

**Figure 4:**
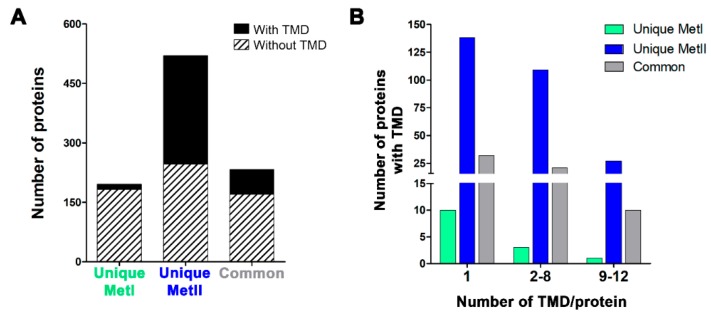
Analysis of transmembrane domain (TMD) present in unique and common mast cell lipid raft proteins identified using Method I or Method II. Prediction of the presence of TMD was done using the TMHMM server v2.0. (**A**) Number of proteins with and without TMDs is shown. (**B**) The number of proteins with TMD versus the number of TMD/protein is given.

Proteins with transmembrane domains are also known to be targeted to lipid rafts [47,48]. The presence of a transmembrane domain was predicted in all proteins identified by both methods using TMHMM server version 2.0 (Figure 4A,B). The common group of proteins contained a sizeable number of proteins with transmembrane domains (27%; 63/233). Few (7.14%; 14/196) unique proteins in MetI had transmembrane domains, and only four of these unique proteins had two or more transmembrane domains. In contrast, the majority (52%; 274/520) of the unique proteins obtained using MetII contained at least one transmembrane domain. Furthermore, the unique proteins isolated by MetII had a higher number of transmembrane domain/protein in comparison to the common proteins or unique proteins identified with MetI. Thus, MetII was more efficient at extracting proteins with transmembrane domains.

### 2.4. Functional Characterization of Mast Cell Lipid Raft Proteome

**Figure 5:**
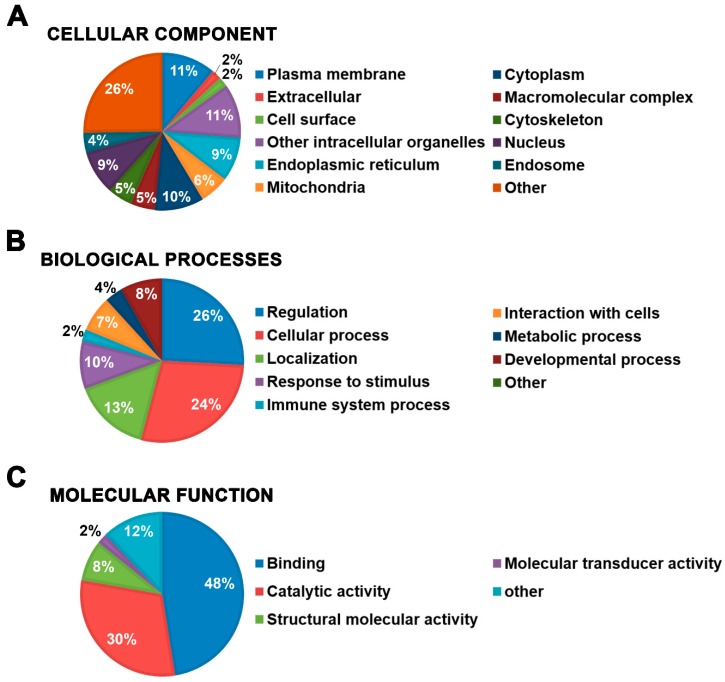
Graphical representation of the GO classes of biological domains association data with the 949 proteins identified in mast cell lipid rafts as annotated by STRAP. Identified proteins were grouped according to the three main GO classes: (**A**) cellular component, (**B**) biological processes, and (**C**) molecular function. The data is expressed as a percentage of proteins associated with each GO term.

Initially, all identified proteins from both MetI and MetII were used for global proteomic analysis. The 949 proteins were annotated according to the three classes of biological domains (cellular component, biological processes, and molecular function) from Gene Ontology (GO) using the Software Tool for Researching Annotations of Proteins (STRAP) [49]. In the cellular component class, 13% of the proteins were associated with the GO terms plasma membrane (11%) and cell surface (2%). Terms associated with cytoplasm (10%), other intracellular organelles (11%), endoplasmic reticulum (9%), mitochondria (6%), and cytoskeleton (5%) were also highly represented (Figure 5A). In the GO class biological processes, the largest groups were proteins categorized by their involvement in regulation (26%) and cellular process (24%). The terms localization (13%) and response to stimulus (10%) were also highly represented (Figure 5B). Finally, in the molecular function class, the GO terms binding (48%), catalytic activity (30%), and structural molecular activity (8%) were the most frequent (Figure 5C).

### 2.5. Distribution among GO Domains of the Mast Cell Lipid Raft Proteins Identified Using Different Post-Isolation Methods

**Figure 6:**
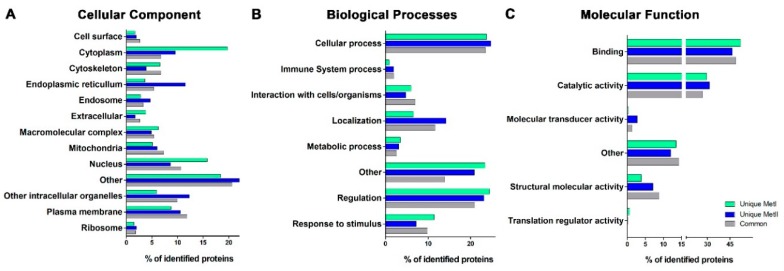
Analysis of unique and common proteins localized in mast cell lipid rafts isolated with MetI or MetII. The unique and common proteins identified using MetI and MetII in lipid rafts from RBL-2H3 MCs were analyzed according to the three main GO classes: (**A**) cellular component, (**B**) biological processes, and (**C**) molecular function. Data for the unique and common proteins identified using MetI and MetII in lipid rafts from RBL-2H3 MCs was annotated using STRAP.

In order to evaluate the differences between the methods, the identified proteins were divided into unique groups for each method and the common group. The proteins were analyzed according to the three classes of biological domains from GO by STRAP [49]. In the GO cellular component class, the unique MetI proteins had a higher percentage of proteins linked to the GO terms: cytoplasm, nucleus, and other. The unique MetII proteins had a higher percentage in cytoplasm, endoplasmic reticulum, other, other intracellular organelles, and plasma membrane. Moreover, the comparison between the methods showed an approximately 1.2–2.5-fold increase in unique MetII proteins linked to the terms endoplasmic reticulum, endosome, other, other intracellular organelles, and plasma membrane. The common group showed a high percentage of proteins associated with the terms nucleus, other, and plasma membrane (Figure 6A). The MetII unique proteins were distributed in practically all the terms in the cellular component in GO. The distribution of the MetII unique proteins with transmembrane domains was practically identical (Figure S3: Total identified proteins and unique MetII proteins with transmembrane domains (TMD) have a similar distribution in the cellular component GO class). In the biological processes GO class, there was an increased percentage of unique proteins from MetI associated with the terms cellular process, other, regulation, and response to stimulus. With MetII the highest percentage of proteins were associated with the terms cellular process, localization, other, and regulation. Among the common proteins, the highest percentage of proteins were associated with the terms cellular process, localization, other, regulation, and response to stimulus (Figure 6B). In the molecular function class, there was an increased percentage of unique proteins isolated with MetI associated with the terms binding, catalytic activity, and other. With MetII, the highest percentage of proteins were associated with the terms binding, catalytic activity, and other. In the common group, the highest percentage of proteins was associated with the terms binding, catalytic activity, and other (Figure 6C). The identified proteins were also analyzed by the highest percentage within each term (Table 2). These analyses demonstrate that functionally, the identified proteins were consistent with their being lipid raft components. Moreover, there was no bias between MetI or MetII as each method was associated with 10 GO terms and the common proteins were associated with 8 GO terms. However, the highest percent of proteins associated with a given GO term differed between the methods.

### 2.6. Functional Enrichment of the Mast Cell Lipid Raft Proteins

**Figure 7:**
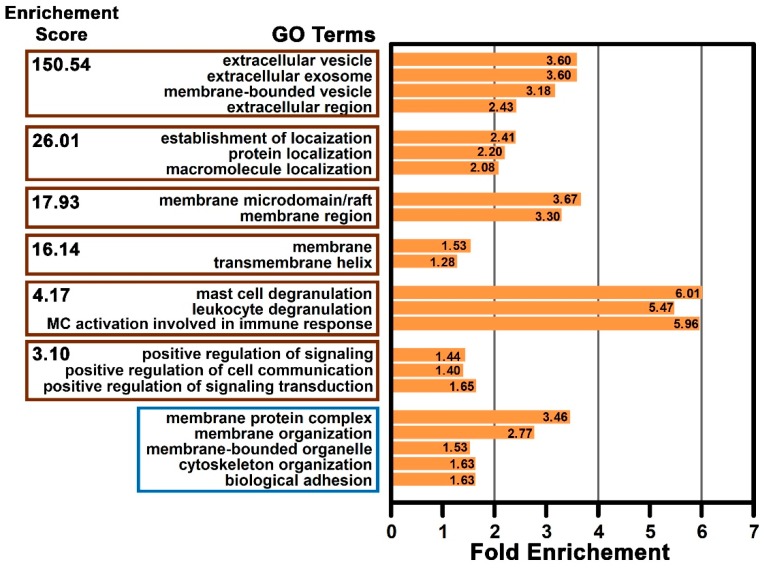
Functional relationship among the proteins and their associated GO terms in the mast cell lipid raft proteome. Groups with the highest enrichment scores following analysis using DAVID are shown. The enrichment score groups (brown rectangles) and non-grouped terms (blue rectangle) are indicated according to their biological significance. The fold enrichment factor (orange bars) is also shown. Data was analyzed using the DAVID Bioinformatics Resources database.

It is also important in proteomic studies to provide a functional assessment of the identified proteins for further system studies. Therefore, the functional relationship among the proteins identified in the MC lipid raft proteome was assessed. An analysis was carried out using the data from *Rattus norvegicus* proteins available in the DAVID Bioinformatics Resources database [50]. Many functional groups were identified in this proteome data. These groups were based on the enrichment score, the number of annotated proteins in each GO term, Fisher exact *p*-value, and false discovery rate (FDR) (Table S6: Enriched GO terms from mast cell lipid raft proteome analysis using DAVID Bioinformatic Resources). The groups with higher enrichment scores are shown in Figure 7. The terms associated with the highest group enrichment score were extracellular vesicle, extracellular exosome, membrane-bound vesicle, and extracellular regions; these terms are all consistent with a localization on or near the plasma membrane. However, the group with the highest fold enrichment was associated with the terms mast cell degranulation, leukocyte degranulation, and MC activation involved in immune response. Other significantly enriched single terms that were not grouped were membrane protein complex, membrane organization, membrane-bounded organelle, cytoskeleton organization, and biological adhesion (Figure 7). This analysis showed that based on the terms encompassed in the enriched groups, as well as the isolated terms, that proteins associated with processes such as vesicle-mediated secretion by immune cells, membrane associated protein localization and stabilization, and immune cell signaling response were significantly enriched in the MC lipid raft proteome.

## 3. Discussion

This proteomic study provides a comprehensive description of the protein composition of RBL-2H3 MC lipid rafts. It provides evidence that the use of two methods to extract lipid raft proteins increases the proteome coverage and improves the identification of integral and associated lipid raft proteins. These proteins are involved in various aspects of MC function, especially those related to MC secretion, organization and stabilization of macromolecules complexes, and signal transduction.

Although the structure, composition, and functional roles of lipid rafts has been extensively investigated in many cell types, there is no universal protocol for the isolation and enrichment of these microdomains [24,25,26]. The lack of a universal protocol for lipid raft isolation stems from the controversy over the biochemical aspects of raft microdomains, such as their instability, size, and highly dynamic nature [2,51,52]. However, the solubilization of whole samples with nonionic mild detergents, such as Triton X-100 at low temperature, followed by sucrose density gradient centrifugation and the recovery of the detergent resistant membranes (DRMs) from the light fractions of the gradient, is the most commonly used method to obtain lipid rafts [13,18,22,51,53,54,55]. This method requires careful interpretation, since differences in lipid raft isolation methods, such as type and concentration of the detergent, as well as the duration of the incubation, make results difficult to compare [21,56]. Moreover, evidence suggest that microdomain fractions obtained using detergent-free methods are less enriched in lipid raft proteins than those prepared with detergents [57].

The proteins found in the low-density fractions may be integral lipid raft constituents or associated with lipid raft components [23,44,47,48,58]. Some of these proteins, such as LAT1, are known to be lipid raft components and serve as a guide to elucidate the contents and properties of these microdomains [23,43,44]. Using LAT1 as a marker, the identification of lipid rafts in the low-density fractions of the sucrose gradient seen here is supported by previous results in RBL-2H3 MCs [6,59,60,61,62] and in bone marrow-derived MCs (BMMCs) [63]. However, in other cell types, other proteins, such as caveolin-1 [64,65], flotillin-1 [64,66,67], and CD-36 [56], are often used to identify lipid rafts. The findings seen here, as well as those reported in the literature, confirm that Fractions 2 and 3 from RBL-2H3 MC homogenates are enriched in lipid rafts, and that these fractions may be pooled and used for proteomic characterization.

Numerous other studies have examined the lipid raft proteome in various cell types and tissues and form the basis of the RaftProtV2 database [25]. The RaftProtV2 database has been used to compare proteomic data from previous studies with newly reported lipid raft proteomes. Other investigations have described a similar percentage of proteins already annotated in the RaftProtV2 database as was seen in the present study [12,13,68,69]. Furthermore, it has been suggested that less than 25% of the total reported membrane raft proteins in the RafProtV2 database fall into the high confidence category [25]. However, in the lipid raft proteome reported in this study, 65% of the proteins were high confidence raft proteins, indicating that the obtention and post-isolations methods used in this study resulted in an enriched lipid rafts fraction from MCs.

Part of the proteins identified in the present investigation had not been annotated in the RaftProtV2 database. However, some of the non-annotated proteins belonged to the same family of proteins as known lipid raft proteins. For example, although FcεRI subunit beta is absent from the RaftProtV2 database, the gamma subunit of the receptor is included [56,70]. The current study confirmed the presence of the FcεRI subunit beta in MC lipid rafts using proteomic and immuno-blot analysis. FcεRI subunit beta had previously been reported as a component of MC lipid rafts using western blots and immunomicroscopy [6,62,63]. Since the RaftProtV2 database does not contain any MC proteomes, not surprisingly, many of the RaftProtV2 non-annotated proteins are mainly expressed by MCs such as chymase, FcεRI subunit beta, mast cell carboxypeptidase A, and mast/stem cell growth factor receptor. Other non-annotated proteins are expressed by MCs and other immune cells, including arachidonate 5-lipoxygenase-activating protein, MHC class I, SAMNS1, macrophage stimulating 1 receptor, and interleukin-3 receptor subunit beta [71,72].

The use of more than one method to extract proteins from the lipid raft fractions may yield a more complete recovery of proteins [25]. Both MetI and MetII have been used extensively in other studies characterizing lipid raft proteomes from different tissues and cell types [1,3,12,14,73,74]. In the present study, extraction of proteins from the lipid raft fractions using MetI or MetII resulted in the identification of proteins common to both methods, as well as proteins unique to each method. The differences observed between MetI and MetII may be explained, in part, by the distinct post-isolation treatment used in each method. The strategy applied in MetI eliminates the sucrose from the enriched lipid raft fractions prior to MS analysis [16]. The procedure used in MetII mixes the sample with another detergent (OGP), followed by methanol-chloroform extraction, which aids in removing the interfering lipids that could be aggregated with the isolated proteins [73,74]. Extraction using MetII yielded a higher number of total proteins, as well as those with lipid modifications typical of membrane raft proteins [1,47].

Lipid modifications can be either permanent cotranslational additions or post-translational modifications [47,48]. The main lipid modifications are S-palmitoylation, isoprenylation, N-terminal myristic acid tails, GPI-anchors, and cysteine acylation. Conjugation to lipids seems to be the most widespread and consistent factor in determining whether a protein will partition into lipid rafts [47,75]. Moreover, the isolation and identification of transmembrane proteins represents one of the most difficult challenges for MS [25]. However, extraction with chloroform/methanol used in MetII greatly improved the yield of the predicted transmembrane proteins. Transmembrane domains typically consist of α–helices or β–sheets, which favor the entry of the proteins into membrane rafts [48,76]. MetII was more efficient in extracting proteins with lipid modification or transmembrane domains and is thus more suited toward providing MC lipid raft proteins for MS investigations. The post-isolation treatment used in MetII may expose a greater number of transmembrane proteins, thus making them available for trypsin digestion [74] and allowing for their subsequent MS identification.

The importance of proteomic studies goes beyond a simple catalogue of the proteins present in a given sample. It also provides information on the functional relationships among the identified proteins. The Gene Ontology (GO) project provides for consistent descriptions of gene products found in different databases. The association of the lipid raft proteins with the GO class biological processes and molecular function showed that the MC lipid raft proteins are associated with terms consistent with the central role of lipid rafts in a number of important cellular events. The terms such as cellular process and catalytic activity can be correlated with the function of lipid rafts in protein processing [77]. Moreover, the terms structural molecular activity, binding, and localization can be related to intracellular trafficking and sorting mechanisms [78,79]. Additionally, the terms regulation and response to stimulus integrate the function of lipid rafts in diverse signal transduction pathways [6,7,32,80].

In the analysis of the GO class cellular component, the proteins associated with terms other than plasma membrane may be explained in part by the fact that many proteins originally located in the cytoplasm or nucleus could be translocated and interact with plasma membrane constituents to form protein complexes [81]. Moreover, lipid rafts may also be found in cellular compartments other than the plasma membrane such as in endoplasmic reticulum and mitochondria [55,74,82,83,84]. Several lipids and proteins associated with lipid rafts are synthesized in the endoplasmic reticulum/Golgi apparatus before being transported to the plasma membrane [27], where they may move laterally within the plasma membrane as well as traffic continuously between the plasma membrane and internal compartments [78]. Finally, nuclear lipid microdomains are important in maintaining subnuclear structures and act as platforms for the transcription process during proliferation [85].

In addition, within the cellular component class, five percent of the identified proteins were associated with the GO term cytoskeleton. The structure and organization of lipid rafts is tightly integrated with the cell cytoskeleton [7,13,19,62]. Studies using live cell imaging have shown that the actin filaments are commonly co-localized with lipid rafts under a stimulus induced co-redistribution of raft components at the cell surface [86]. Moreover, the dynamic rearrangement of the cytoskeleton in MCs following stimulation can act to stabilize the lipid raft clusters [6,62,87,88].

The group with the highest enrichment score in the MC lipid raft proteome included the GO terms extracellular vesicle, membrane-bounded vesicle, extracellular exosome, and extracellular region. The terms that showed the greatest fold enrichment factor included the terms mast cell degranulation, leukocyte degranulation, and MC activation involved in immune response. Lipid rafts have ideal features for participating in intracellular membrane transport, acting as a crucial regulator of vesicle cargo and their consequent endocytosis and secretion [89,90,91]. Notably, the hallmark of MC activation via FcεRI is the immediate release via highly regulated exocytosis (degranulation) of inflammatory mediators that are presynthesized and stored in MC secretory granules [92,93]. Moreover, perturbation of the raft structure has a profound impact on FcεRI-mediated degranulation in MCs [6,62,94].

The group with the next-highest enrichment score included proteins that were involved with the establishment of localization, protein localization, and macromolecular localization. Many of the proteins associated with these terms are adaptors or scaffolding proteins. These proteins then act as docking sites for signaling molecules in lipid raft domains forming a multicomponent assembly, which facilitates signal transduction in diverse pathways [88,95]. Furthermore, other raft proteins identified in this study were also related to the signal transduction pathways in MCs. These proteins included CD45 (receptor-type tyrosine-protein phosphatase C), FcγRII (low affinity immunoglobulin Fc gamma receptor II), MC/stem cell growth factor receptor, IP_3_-receptor, integrins, phospholipid scramblase, serine/threonine-protein phosphatase PP1-alpha, protein-tyrosine phosphatase 1B, IQGAP1, calreticulin, calmodulin, DJ-1, RhoA, Gnai-2, and cdc42 [96,97,98,99,100,101,102].

Another group with highly enriched scores included the GO terms membrane microdomain/raft and membrane region. Several transmembrane proteins, known to be associated with lipid rafts, were also identified in our MC proteome such as the flotillins and prohibitins. Other scaffold proteins, such as tetraspanins (CD81 and CD63) and the important transmembrane adaptor proteins (TRAPs) in immunoreceptor signaling LAT1 and NTAL (non-T cell activation linker; LAT2) [103], were also identified in this lipid raft proteome. LAT1 and NTAL become rapidly phosphorylated in FcεRI-activation, resulting in its association with numerous signaling molecules [32,43,104]. Moreover, these TRAPs are also involved in the regulation of MC morphology, adhesion, and chemotaxis [104,105]. RACK1 (receptor for activated C kinase 1), a member of the tryptophan-aspartate repeat family of proteins, was also identified. RACK1 adopts a highly conserved seven-bladed β-propeller structure that serves as binding sites for multiple partners [106,107]. Recently, RACK1 was identified as a crucial component of a multiprotein complex formed in T-cell lipid rafts upon TCR (T-cell antigen receptor) activation [108]. Despite the significant role that RACK1 plays in shuttling and anchoring proteins and its involvement in immunoregulatory responses [107,108,109], RACK1 has not previously been described in MCs.

There was also an increased enrichment score of the group containing the GO terms MC degranulation, leukocyte degranulation, and MC activation involved in immune response. This group also showed the highest fold enrichment. This MC lipid raft proteome consistently identified many of the proteins already described to be involved in MC degranulation, such as SNAREs (soluble N-ethylmaleimide-sensitive-factor attachment protein receptor), including VAMP (vesicle-associated membrane protein) 7 [110], VAMP3, VAMP8 [110,111], SNAP-23 (synaptosomal-associated protein 23) [110,112], Syntaxin 3 [113], and Syntaxin 4 [113]. In addition to the accessory proteins the RAB GTPases (RAB3D, RAB5, RAB7, RAB9A, RAB11, RAB27A, RAB27B, and RAB43) [114,115,116], syntaxin-binding protein 2 (MUC18-2) [113], α-SNAP (alpha-soluble NSF attachment protein) [93], and syntaxin-binding protein 5 (Tomosyn-1) [117] were also identified.

The functionally related group that includes the GO terms positive regulation of signaling, positive regulation of cell communication, positive regulation of signal transduction was also enriched. Cell membrane lipids and lipid raft proteins have been implicated in various signaling events, including those dependent on the immunoglobulin-receptor superfamily in immune cells [18,32,80,118]. In MCs, the events immediately following FcεRI activation that result in downstream signaling are still not completely understood [32,87,92]. However, it is known that the aggregated receptors are translocated into lipid rafts where the SFKs are activated with subsequent phosphorylation of the receptor subunits [32,87,88]. The N-terminal sequences of SFKs allow them to anchor to saturated fatty acid derivatives in the inner leaflet of the plasma membrane, enabling their partitioning into lipid rafts [119,120]. Five of the eight members of SFKs, including Lyn, Fyn, Yes, Fgr, and Lck, were identified in this lipid raft proteome from non-activated RBL-2H3 MCs.

## 4. Conclusions

Based on the present data, a comprehensive study of the MC lipid raft proteome provides strong evidence that our two methods increased the proteome coverage and improved the identification of integral and associated lipid raft proteins. These proteins are involved in various aspects of MC function, especially those related to MC regulated secretion, organization and stabilization of macromolecules complexes, and signal transduction pathways. Thus, this identification of the raft membrane proteins could provide important tools for further investigation of molecular mechanisms related to the immunoregulatory functions of MCs.

## 5. Materials and Methods

### 5.1. Cell Culture

RBL-2H3, a rat MC line [42], was grown as monolayers in Dulbecco’s Minimum Essential Medium (DMEM) supplemented with 15% fetal calf serum and an antibiotic-antimycotic mixture (100 U/mL penicillin, 100 μg/mL streptomycin, and 0.25 μg/mL amphotericin B) in a humidified environment containing 5% CO_2_ in air at 37 °C. All reagents used for the cell culture were purchased from ThermoFisher Scientific (Thermo Fisher Scientific, Invitrogen, Carlsbad, CA, USA).

### 5.2. Isolation of Lipid Rafts from RBL-2H3 Mast Cells

The lipid rafts were isolated using sucrose density-gradient ultracentrifugation, essentially as previously described [120]. Briefly, (3–5) × 10^7^ RBL-2H3 MCs were plated in 150 mm tissue culture dishes (Corning Incorporated - Life Sciences, Oneonta, NY, USA). After 24 h, the cells were washed twice with ice-cold PBS and harvested and lysed on ice with 0.05% Triton X-100 (*v*/*v*) in 2.6 mL ice-cold MES buffer (25 mM 2-(4-Morpholino) ethane sulfonic acid, pH 6.5, 150 mM NaCl, 5 mM EDTA, and 1 mM Na_3_VO_4_) containing 2 mM PMSF and 50 μL/mL protease inhibitor cocktail. All reagents were purchased from Millipore Sigma (St. Louis, MO, USA). The resulting suspension was then homogenized 30 times on ice using a Dounce homogenizer with a tight-fitting piston, followed by incubation on ice for 15 min. The lysates were then centrifuged for 10 min at 900× *g* and the 2.6 mL of supernatant was overlaid on 2.6 mL 80% sucrose (*w*/*v*) in a MES buffer in the bottom of a 13 mL Beckman centrifuge tube (Beckman Coulter, Fullerton, CA, USA) and gently vortexed to give the final concentration of 40% sucrose. Thereafter, the sample was overlaid with 5.2 mL of 35% sucrose (*w*/*v*) in lysis buffer. Then, 2.6 mL 5% sucrose (*w*/*v*) in lysis buffer was added on top to form a discontinuous gradient. Samples were centrifuged using a Beckman SW40Ti rotor (Beckman Coulter) at 38,000 rpm for 20 h at 4 °C. Fractions 1.3 mL in volume were collected from the top of the tube.

### 5.3. Immunoblotting Analysis of Lipid Raft Enriched Fractions

In order to localize the lipid rafts in the gradient fractions, 5 μL from each fraction were immobilized on Hybond membranes (GE Healthcare Life Sciences, Marlborough, MA, USA) using the vacuum microfiltration system Bio Dot (Bio-Rad Laboratories, Inc., Hercules, CA, USA). The membranes were immunoblotted as previously described [121]. The following primary antibodies were used: goat polyclonal antibody anti-mouse LAT (Clone Q-20) (Santa Cruz Biotechnology Inc., Santa Cruz, CA, USA), rabbit polyclonal antibody anti-human Lyn (Clone 44 sc-15) (Santa Cruz Biotechnology Inc.), mouse mAb anti-FcεRI beta subunit antibodies generously provided by Dr. Reuben Siraganian (NIH—NIDCR, Bethesda, MD, USA), rabbit polyclonal antibody anti-human Flotillin-1 (ab41927) and rabbit polyclonal antibody anti-human Histone H3 (ab1791) (Abcam, Cambridge, MA, USA), and mouse monoclonal antibody anti-rat GD1b derived gangliosides (Clone AR32AA4) (BD Pharmingen, San Jose, CA, USA). The secondary antibodies used were donkey anti-goat IgG conjugated to horseradish peroxidase (HRP), donkey anti-rabbit IgG conjugated to HRP, and donkey anti-mouse IgG conjugated to HRP (Jackson ImmunoResearch Laboratories Inc., West Grove, PA, USA). The membranes were developed using enhanced chemiluminescence (ECL Kit; GE Healthcare) and the images were obtained with ImageQuant LAS 4000 (GE Healthcare).

### 5.4. Extraction and Digestion of Mast Cell Lipid Raft Proteins for Mass Spectrometry

The lipid raft enriched fractions (the low-density Fractions 2 and 3) obtained from three independent experiments were pooled and the sample was used for two different post-isolation treatment methods. In MetI, the samples were mixed with ice-cold 50 mM NH_4_HCO_3_ (Millipore Sigma) pH 7.9, washed, and concentrated using an Amicon^®^ Ultra-3 Centrifugal Filter (Merk Millipore, Burlington, MA, USA). In MetII, the pooled fractions were gently mixed with ice-cold MNE-buffer (25 mM MES, pH 6.5; 5 mM EDTA; 150 mM NaCl) and pelleted using centrifugation (200,000 × *g*, 1 h). Samples were subsequently mixed with 100 µL of 100 mM n-octyl-beta-D-gluco-pyranoside (OGP) followed by methanol-chloroform extraction as previously described [74].

For both methods, the proteins were enzymatically digested as described previously [122,123] with some modifications. Briefly, the protein content was quantified using the Bradford reagent (Millipore Sigma), and bovine serum albumin (BSA; Millipore Sigma) was used as a standard [124]. Then, 50 μg of protein from each method was added to 10 mL of 50 mM ammonium bicarbonate, pH 8.5. Then, 25 μL of RapiGEST^TM^ SF Surfactante (0.2% *v*/*v*) (Waters, Milford, PA, USA) was added, and the sample was vortexed and then incubated at 80 °C for 15 min. The sample was reduced via incubation with 2.5 μL of 100 mM dithiothreitol (DTT) (GE Healthcare) at 60 °C for 30 min and cysteine alkylation was done by incubating the samples with 2.5 μL of 300 mM iodocetamide (GE Healthcare) for 30 min at room temperature in the dark. The proteins were subsequently digested with 10 μL of trypsin (0.05 mg/μL; Promega, Madison, WI, USA) at 37 °C for 16 h. The samples were subsequently acidified with 10 μL of 5% trifluoracetic acid (*v*/*v*) (Millipore Sigma), followed by incubation at 37 °C for 90 min in order to stop the trypsin digestion and precipitate the RapiGEST™ SF Surfactante, and centrifugation at 21,000× *g* at 4 °C for 30 min was carried out. The supernatants were dried in a Savant™ SpeedVac™ Concentrator (ThermoFisher Scientific), and all obtained peptides were suspended in 49.5 μL of a solution containing 20 mM ammonium formate and 100 fmol/μL yeast enolase (MassPREP^TM^ protein; Waters) as an internal standard.

### 5.5. Nano-Electrospray Ionization Source (ESI) and Ultra-Performance Liquid Chromatography Mass Spectrometry (UPLC-MS^E^)

Nanoscale LC separation of tryptic peptides was performed using a nanoACQUITY^TM^ system (Waters) equipped with a nanoEase^TM^ 5 mm × Bridge^TM^ BEH130 C18 300 mm × 50 mm precolumn; trap column 5 mm, 180 mm × 20 mm; and BEH130 C18 1.7 mm, 100 mm × 100 mm analytical reversed-phase column (Waters). The peptides were separated into 10 fractions and the gradient elution was performed as follows: 8.7, 11.4, 13.2, 14.7, 16, 17.4, 18.9, 20.7, 23.4, and 65% acetonitrile/0.1% (*v*/*v*) formic acid, with a flow rate of 2000 mL/min. The source was operated in positive ionization mode nano-ESI (+). GFP [Glu]^1^-fibrinopeptide B human ([MC2H]^2+^ = 785.8426) (Millipore Sigma) was used for lock mass calibration of the apparatus, using a constant flow rate of 0.5 μL/min at a concentration of 200 fmol protein. MS analysis was performed on a Synapt G1 MS^TM^ (Waters) equipped with a NanoElectronSpray source and two mass analyzers: a quadrupole and a time-of-flight (TOF) operating in V-mode. The mass spectrometer was programmed in the data-dependent acquisition mode, in which a full scan in the m/z region of 50–2000 was used. Data were obtained using the instrument in the MS^E^ mode, which switched between the low energy (6 V) and elevated energy (40 V) acquisition modes every 0.4 s. Samples were analyzed using three replicates.

### 5.6. Data Processing and Protein Identification Analysis

The acquired MS raw data were processed using the ProteinLynx Global Server version 2.4 (PLGS) (Waters). The data were subjected to automatic background subtraction, deisotoping, and charge state deconvolution. After processing, each ion comprised an exact mass-retention time (EMRT) that contained the retention time, intensity-weighted average charge, inferred molecular weight based on charge, and m/z. The processed spectra were searched against *Rattus norvegicus* entries (29,952 sequences) from the UniProt database (http://www.uniprot.org). The mass error tolerance for peptide identification was under 50 ppm. The parameters for protein identification included: (I) the detection of at least two fragment ions per peptide; (II) five fragments per protein; (III) the determination of at least one peptide per protein; (IV) carbamidomethylation of cysteine as a fixed modification; (V) phosphorylation of serine, threonine, and tyrosine, and oxidation of methionine were considered as variable modifications; (VI) maximum protein mass (600 kDa); (VII) one missed cleavage site was allowed for trypsin; (VIII) and a maximum false positive ratio (FDR) of 4% was allowed. The minimum repeat rate for each protein in all replicates was two. The protein table was compared using the Spotfire^®^ v8.0 software, and graphs were generated for all data.

### 5.7. Bioinformatics Analysis

To detect the co-differentially presented protein in our data sets, we performed a comparative analysis of the overlaps using Venn diagrams (http://bioinformatics.psb.ugent.be/webtools/Venn/). RaftProtV2 database (http://raftprot.org) was used to systematically analyze the known lipid raft proteins [26]. Since proteomes of rat lipid rafts correspond to less than 13% of the included data [26], data obtained from human and mouse lipid raft proteomes was also used in this analysis.

The graph of experimentally determined lipid modification types was generated using PhosphoSitePlus (http://www.phosphosite.org) [125]; SwissPalm (http://www.swisspalm.org) [126]; PRENbase (http://mendel.imp.ac.at/PrePS/PRENbase) [127]; MYRbase (http://mendel.imp.ac.at/myristate/myrbase) [128]; and PredGPI (http://gpcr.biocomp.unibo.it/predgpi) [129]. In order to systematically investigate the denaturing properties of the applied methods, an analysis of potential transmembrane domains (TMD) was conducted using TMHMM 2.0 (http://www.cbs.dtu.dk/services/TMHMM/) on the complete data set [130,131].

Gene Ontology (GO) annotation charts based on the complete list of UniProt Knowledgebase accession entries were generated using STRAP (Software Tool for Researching Annotations of Proteins) [49]. The Database for Annotation Visualization and Integrated Discovery (DAVID; http://david.ncifcrf.gov), version 6.8, National Institute of Allergy and Infectious Diseases [50], was used for enrichment analysis, enrichment scores for annotation groups, and fold enrichment factors for individual GO terms, as well as Fisher’s exact *p*-values and false discovery rates (FDR) using Benjamini–Hochberg coefficients, adjusting for multiple comparisons.

## Figures and Tables

**Table 1:** Mast cell lipid raft proteins annotated with the highest number of citations supported by experimental evidence in RaftProtV2 database.

Method	Protein Accession	Protein Description	Gene Name	Experimental Evidence	Number of Citations
MetII	P63018	Heat shock cognate 71 kDa protein	Hspa8	H (3) M (3) R (3)	102
MetII	Q9Z1E1	Flotillin-1	Flot1	H (3) M (1) R (3)	101
MetI; MetII	P15999	ATP synthase subunit alpha mitochondrial	Atp5fla	H (3) M (3) R (3)	98
MetI	P10719	ATP synthase subunit beta mitochondrial	Atp5f1b	H (1) M (1) R (3)	97
MetI; MetII	Q9Z2L0	Voltage-dependent anion-selective channel protein 1	Vdac1	H (3) M (3) R (3)	97
MetI; MetII	P67779	Prohibitin	Phb	H (3) M (1) R (1)	91
MetI; MetII	P60711	Actin cytoplasmic 1	Actb	H (3) M (3) R (3)	90
MetII	Q9Z2S9	Flotillin-2	Flot2	H (3) M (3) R (3)	90
MetI; MetII	P81155	Voltage-dependent anion-selective channel protein 2	Vdac2	H (3) M (3) R (1)	90
MetI; MetII	P06685	Sodium/potassium-transporting ATPase subunit alpha-1	Atp1a1	H (1) M (1) R (1)	89
MetI; MetII	P04797	Glyceraldehyde-3-phosphate dehydrogenase	Gapdh	H (1) M (1) R (1)	88
MetI; MetII	P54311	Transducin beta-1	Gnb1	H (3) M (3) R (3)	87
MetI	Q07936	Annexin A2	Anxa2	H(1) M (3) R (3)	86
MetI; MetII	P04897	Guanine nucleotide-binding protein G(i) subunit alpha-2	Gnai2	H (3) M (3) R (3)	86
MetI; MetII	P06761	Endoplasmic reticulum chaperone BiP	Hspa5	H (1) M (1) R (1)	86
MetI; MetII	P35565	Calnexin	Canx	H (1) M (1) R (1)	83
MetI; MetII	G3V6P7	Myosin heavy chain 9	Myh9	H (3) M (3)	83
MetI	P31000	Vimentin	Vim	H (3) M (3) R (3)	81
MetI; MetII	P09527	Ras-related protein Rab-7a	Rab7a	H (1) M (1) R (1)	79
MetI; MetII	P26453	Basigin	Bsg	H (3) M (3) R (3)	78
MetI; MetII	D4A133	V-type proton ATPase catalytic subunit A	Atp6v1a	H (3) M (3)	78
MetI; MetII	P63102	14-3-3 protein zeta/delta	Ywhaz	H (1) M (1) R (1)	77
MetI; MetII	P11442	Clathrin heavy chain 1	Cltc	H (3) M (3) R (3)	77
MetII	F1M779	Clathrin heavy chain	Cltc	H (3) M (3) R (3)	77
MetII	Q5XI04	Erythrocyte band 7 integral membrane protein	Stom	H (1) M (1)	77
MetI; MetII	P54313	Transducin beta-2	Gnb2	H (3) M (3) R (3)	76
MetII	B5DEH2	Erlin-2	Erlin2	H (3) M (3) R (3)	74
MetII	O70377	Synaptosomal-associated protein 23 (SNAP-23)	Snap23	H (3) M (3) R (3)	74
MetI; MetII	P32551	Cytochrome b-c1 complex subunit 2 mitochondrial	Uqcrc2	H (3) M (3) R (3)	72
MetI; MetII	Q5XIH7	Prohibitin 2	Phb2	H (3) M (3) R (3)	71

Method—Post-isolation treatment method; Protein accession—UniProt protein accession number; Protein description—Functional description; Gene name—Name of gene that codes for the protein sequence; Experimental evidence—Experimental data validating inclusion as a lipid raft protein; (1) indicates protein identification by more than one biochemical extraction method; (3) indicates fulfillment of criteria (1) and the sensitivity to more than one raft perturbation technique; Number of citations—Number of studies with supporting experimental evidence describing the lipid raft protein; H—Human; M—Mouse; R—Rat.

**Table 2:** Highest percentage of annotated proteins within each GO term.

GO Term	Unique MetI	Unique MetII	Common
**Cellular Component Class**			
Cell surface			**+**
Cytoplasm	**+**		
Cytoskeleton	**+**		**+**
Endoplasmic reticulum		**+**	
Endosome		**+**	
Extracellular	**+**		
Macromolecular complex	**+**		
Mitochondria			**+**
Nucleus	**+**		
Other		**+**	
Other intracellular organelles		**+**	
Plasma membrane			**+**
Ribosomes		**+**	
**Biological Processes Class**			
Cellular process		**+**	
Immune system process		**+**	**+**
Interaction with cells/organelles			**+**
Localization		**+**	
Metabolic process	**+**		
Other	**+**		
Regulation	**+**		
Response to stimulus	**+**		
**Molecular Function Class**			
Binding	**+**		
Catalytic activity		**+**	
Molecular transducer activity		**+**	
Other			**+**
Structural molecular activity			**+**

## Supplementary Materials

Supplementary materials can be found at https://www.mdpi.com/1422-0067/20/16/3904/s1. Figure S1: Dynamic range of the proteomic analysis. Figure S2: Immuno-blot analysis of the β-subunit of FcεRI from RBL-2H3 MC lipid rafts. Figure S3: Total identified proteins and unique MetII proteins with transmembrane domains (TMD) have a similar distribution in the cellular component GO class. Table S1: Detailed annotation of proteins identified in Method I. Table S2: Detailed annotation of proteins identified in Method II. Table S3: Detailed annotation of proteins identified in Methods I and II. Table S4: Mast cell lipid raft proteins absent from RaftProtV2 database. Table S5: Mast cell lipid raft proteins analyzed by RafProtV2 database. Table S6: Enriched GO terms from mast cell lipid raft proteome analysis using DAVID Bioinformatic Resources.

## Abbreviations

MC	Mast cell
MetI	Method I
MetII	Method II

## References

[1] Pike L.J. (2009). The challenge of lipid rafts. J. Lipid Res..

[2] Simons K., Gerl M.J. (2010). Revitalizing membrane rafts: New tools and insights. Nat. Rev. Mol. Cell Biol..

[3] Bieberich E. (2018). Sphingolipids and lipid rafts: Novel concepts and methods of analysis. Chem. Phys. Lipids.

[4] Enoki T.A., Heberle F.A., Feigenson G.W. (2018). FRET Detects the Size of Nanodomains for Coexisting Liquid-Disordered and Liquid-Ordered Phases. Biophys. J..

[5] Sezgin E. (2017). Super-resolution optical microscopy for studying membrane structure and dynamics. J. Phys. Condens. Matter.

[6] Silveira E., Souza A.M., Mazucato V.M., Jamur M.C., Oliver C. (2011). Lipid rafts in mast cell biology. J. Lipids.

[7] Head B.P., Patel H.H., Insel P.A. (2014). Interaction of membrane/lipid rafts with the cytoskeleton: Impact on signaling and function: Membrane/lipid rafts, mediators of cytoskeletal arrangement and cell signaling. Biochim. Biophys. Acta.

[8] Lingwood D., Simons K. (2010). Lipid rafts as a membrane-organizing principle. Science.

[9] Sezgin E., Levental I., Mayor S., Eggeling C. (2017). The mystery of membrane organization: Composition, regulation and roles of lipid rafts. Nat. Rev. Mol. Cell Biol..

[10] Kusumi A., Suzuki K. (2005). Toward understanding the dynamics of membrane-raft-based molecular interactions. Biochim. Biophys. Acta.

[11] Jia J.Y., Lamer S., Schümann M., Schmidt M.R., Krause E., Haucke V. (2006). Quantitative proteomics analysis of detergent-resistant membranes from chemical synapses: Evidence for cholesterol as spatial organizer of synaptic vesicle cycling. Mol. Cell. Proteomics.

[12] Liu Y., Yan G., Gao M., Zhang X. (2018). Magnetic capture of polydopamine-encapsulated Hela cells for the analysis of cell surface proteins. J. Proteomics.

[13] Shah A.D., Inder K.L., Shah A.K., Cristino A.S., McKie A.B., Gabra H., Davis M.J., Hill M.M. (2016). Integrative Analysis of Subcellular Quantitative Proteomics Studies Reveals Functional Cytoskeleton Membrane-Lipid Raft Interactions in Cancer. J. Proteome Res..

[14] Feuk-Lagerstedt E., Movitz C., Pellmé S., Dahlgren C., Karlsson A. (2007). Lipid raft proteome of the human neutrophil azurophil granule. Proteomics.

[15] Nebl T., Pestonjamasp K.N., Leszyk J.D., Crowley J.L., Oh S.W., Luna E.J. (2002). Proteomic analysis of a detergent-resistant membrane skeleton from neutrophil plasma membranes. J. Biol. Chem..

[16] Zhang N., Shaw A.R., Li N., Chen R., Mak A., Hu X., Young N., Wishart D., Li L. (2008). Liquid chromatography electrospray ionization and matrix-assisted laser desorption ionization tandem mass spectrometry for the analysis of lipid raft proteome of monocytes. Anal. Chim. Acta.

[17] Dhungana S., Merrick B.A., Tomer K.B., Fessler M.B. (2009). Quantitative proteomics analysis of macrophage rafts reveals compartmentalized activation of the proteasome and of proteasome-mediated ERK activation in response to lipopolysaccharide. Mol. Cell. Proteomics.

[18] Bini L., Pacini S., Liberatori S., Valensin S., Pellegrini M., Raggiaschi R., Pallini V., Baldari C.T. (2003). Extensive temporally regulated reorganization of the lipid raft proteome following T-cell antigen receptor triggering. Biochem. J..

[19] Lin S.L., Chien C.W., Han C.L., Chen E.S., Kao S.H., Chen Y.J., Liao F. (2010). Temporal proteomics profiling of lipid rafts in CCR6-activated T cells reveals the integration of actin cytoskeleton dynamics. J. Proteome Res..

[20] Gupta N., Wollscheid B., Watts J.D., Scheer B., Aebersold R., DeFranco A.L. (2006). Quantitative proteomic analysis of B cell lipid rafts reveals that ezrin regulates antigen receptor-mediated lipid raft dynamics. Nat. Immunol..

[21] Man P., Novák P., Cebecauer M., Horváth O., Fiserová A., Havlícek V., Bezouska K. (2005). Mass spectrometric analysis of the glycosphingolipid-enriched microdomains of rat natural killer cells. Proteomics.

[22] Foster L.J., Chan Q.W. (2007). Lipid raft proteomics: More than just detergent-resistant membranes. Subcell. Biochem..

[23] Inder K.L., Davis M., Hill M.M. (2013). Ripples in the pond--using a systems approach to decipher the cellular functions of membrane microdomains. Mol. Biosyst..

[24] Minogue S., Waugh M.G. (2012). Lipid rafts, microdomain heterogeneity and inter-organelle contacts: Impacts on membrane preparation for proteomic studies. Biol. Cell.

[25] Mohamed A., Shah A.D., Chen D., Hill M.M. (2019). RaftProt V2: Understanding membrane microdomain function through lipid raft proteomes. Nucleic Acids Res..

[26] Mohamed A., Robinson H., Erramouspe P.J., Hill M.M. (2018). Advances and challenges in understanding the role of the lipid raft proteome in human health. Expert Rev. Proteomics.

[27] Zheng Y.Z., Berg K.B., Foster L.J. (2009). Mitochondria do not contain lipid rafts, and lipid rafts do not contain mitochondrial proteins. J. Lipid Res..

[28] Anand P., Singh B., Jaggi A.S., Singh N. (2012). Mast cells: An expanding pathophysiological role from allergy to other disorders. Naunyn Schmiedebergs Arch. Pharmacol..

[29] Da Silva E.Z., Jamur M.C., Oliver C. (2014). Mast cell function: A new vision of an old cell. J. Histochem. Cytochem..

[30] Galli S.J. (2016). The Mast Cell-IgE Paradox: From Homeostasis to Anaphylaxis. Am. J. Pathol..

[31] Jahn T., Leifheit E., Gooch S., Sindhu S., Weinberg K. (2007). Lipid rafts are required for Kit survival and proliferation signals. Blood.

[32] Holowka D., Baird B. (2015). Nanodomains in early and later phases of FcɛRI signalling. Essays Biochem..

[33] Mazucato V.M., Silveira E., Souza A.M., Nicoletti L.M., Jamur M.C., Oliver C. (2011). GD1b-derived gangliosides modulate FcεRI endocytosis in mast cells. J. Histochem. Cytochem..

[34] Fridriksson E.K., Shipkova P.A., Sheets E.D., Holowka D., Baird B., McLafferty F.W. (1999). Quantitative analysis of phospholipids in functionally important membrane domains from RBL-2H3 mast cells using tandem high-resolution mass spectrometry. Biochemistry.

[35] Sadroddiny E., Ai J., Carroll K., Pham T.K., Wright P., Pathak A., Helm B. (2012). Protein profiling of the secretome of FcεRI activated RBL-2H3.1 cells. Iran. J. Immunol..

[36] Gage M.C., Keen J.N., Buxton A.T., Bedi M.K., Findlay J.B. (2009). Proteomic analysis of IgE-mediated secretion by LAD2 mast cells. J. Proteome Res..

[37] Han X., Smith N.L., Sil D., Holowka D.A., McLafferty F.W., Baird B.A. (2009). IgE receptor-mediated alteration of membrane-cytoskeleton interactions revealed by mass spectrometric analysis of detergent-resistant membranes. Biochemistry.

[38] Dráber P., Dráberová L. (2002). Lipid rafts in mast cell signaling. Mol. Immunol..

[39] Passante E., Frankish N. (2009). The RBL-2H3 cell line: Its provenance and suitability as a model for the mast cell. Inflamm. Res..

[40] Falcone F.H., Wan D., Barwary N., Sagi-Eisenberg R. (2018). RBL cells as models for in vitro studies of mast cells and basophils. Immunol. Rev..

[41] Eccleston E., Leonard B.J., Lowe J.S., Welford H.J. (1973). Basophilic leukaemia in the albino rat and a demonstration of the basopoietin. Nat. New Biol..

[42] Barsumian E.L., Isersky C., Petrino M.G., Siraganian R.P. (1981). IgE-induced histamine release from rat basophilic leukemia cell lines: Isolation of releasing and nonreleasing clones. Eur. J. Immunol..

[43] Rivera J., Arudchandran R., Gonzalez-Espinosa C., Manetz T.S., Xirasagar S. (2001). A perspective: Regulation of IgE receptor-mediated mast cell responses by a LAT-organized plasma membrane-localized signaling complex. Int. Arch. Allergy Immunol..

[44] Janes P.W., Ley S.C., Magee A.I. (1999). Aggregation of lipid rafts accompanies signaling via the T cell antigen receptor. J. Cell Biol..

[45] Guo N.H., Her G.R., Reinhold V.N., Brennan M.J., Siraganian R.P., Ginsburg V. (1989). Monoclonal antibody AA4, which inhibits binding of IgE to high affinity receptors on rat basophilic leukemia cells, binds to novel alpha-galactosyl derivatives of ganglioside GD1b. J. Biol. Chem..

[46] Sheets E.D., Holowka D., Baird B. (1999). Critical role for cholesterol in Lyn-mediated tyrosine phosphorylation of FcepsilonRI and their association with detergent-resistant membranes. J. Cell Biol..

[47] Levental I., Grzybek M., Simons K. (2010). Greasing their way: Lipid modifications determine protein association with membrane rafts. Biochemistry.

[48] Lorent J.H., Levental I. (2015). Structural determinants of protein partitioning into ordered membrane domains and lipid rafts. Chem. Phys. Lipids.

[49] Bhatia V.N., Perlman D.H., Costello C.E., McComb M.E. (2009). Software tool for researching annotations of proteins: Open-source protein annotation software with data visualization. Anal. Chem..

[50] Huang D.W., Sherman B.T., Tan Q., Collins J.R., Alvord W.G., Roayaei J., Stephens R., Baseler M.W., Lane H.C., Lempicki R.A. (2007). The DAVID Gene Functional Classification Tool: A novel biological module-centric algorithm to functionally analyze large gene lists. Genome Biol..

[51] Simons K., Ikonen E. (1997). Functional rafts in cell membranes. Nature.

[52] Garner A.E., Smith D.A., Hooper N.M. (2008). Visualization of detergent solubilization of membranes: Implications for the isolation of rafts. Biophys. J..

[53] Brown D.A., Rose J.K. (1992). Sorting of GPI-anchored proteins to glycolipid-enriched membrane subdomains during transport to the apical cell surface. Cell.

[54] Kim D.K., Kim H.S., Kim A.R., Jang G.H., Kim H.W., Park Y.H., Kim B., Park Y.M., Beaven M.A., Kim Y.M. (2013). The scaffold protein prohibitin is required for antigen-stimulated signaling in mast cells. Sci. Signal..

[55] Poston C.N., Duong E., Cao Y., Bazemore-Walker C.R. (2011). Proteomic analysis of lipid raft-enriched membranes isolated from internal organelles. Biochem. Biophys. Res. Commun..

[56] Rabani V., Davani S., Gambert-Nicot S., Meneveau N., Montange D. (2016). Comparative lipidomics and proteomics analysis of platelet lipid rafts using different detergents. Platelets.

[57] Foster L.J., De Hoog C.L., Mann M. (2003). Unbiased quantitative proteomics of lipid rafts reveals high specificity for signaling factors. Proc. Natl. Acad. Sci. USA.

[58] Zech T., Ejsing C.S., Gaus K., de Wet B., Shevchenko A., Simons K., Harder T. (2009). Accumulation of raft lipids in T-cell plasma membrane domains engaged in TCR signalling. EMBO J..

[59] Souza S.A.M., Mazucato V.M., de Castro R.O., Matioli F., Ciancaglini P., de Paiva Paulino T., Jamur M.C., Oliver C. (2008). The alpha-galactosyl derivatives of ganglioside GD(1b) are essential for the organization of lipid rafts in RBL-2H3 mast cells. Exp. Cell Res..

[60] Hitomi T., Zhang J., Nicoletti L.M., Grodzki A.C., Jamur M.C., Oliver C., Siraganian R.P. (2004). Phospholipase D1 regulates high-affinity IgE receptor-induced mast cell degranulation. Blood.

[61] Wilson B.S., Pfeiffer J.R., Surviladze Z., Gaudet E.A., Oliver J.M. (2001). High resolution mapping of mast cell membranes reveals primary and secondary domains of Fc(epsilon)RI and LAT. J. Cell Biol..

[62] Holowka D., Sheets E.D., Baird B. (2000). Interactions between Fc(epsilon)RI and lipid raft components are regulated by the actin cytoskeleton. J. Cell Sci..

[63] Wang X., Ma D.W., Kang J.X., Kulka M. (2015). N-3 Polyunsaturated fatty acids inhibit Fc ε receptor I-mediated mast cell activation. J. Nutr. Biochem..

[64] Hashimoto N., Hamamura K., Kotani N., Furukawa K., Kaneko K., Honke K. (2012). Proteomic analysis of ganglioside-associated membrane molecules: Substantial basis for molecular clustering. Proteomics.

[65] Reeves V.L., Thomas C.M., Smart E.J. (2012). Lipid rafts, caveolae and GPI-linked proteins. Adv. Exp. Med. Biol..

[66] Kato N., Nakanishi M., Hirashima N. (2006). Flotillin-1 regulates IgE receptor-mediated signaling in rat basophilic leukemia (RBL-2H3) cells. J. Immunol..

[67] Stuermer C.A., Lang D.M., Kirsch F., Wiechers M., Deininger S.O., Plattner H. (2001). Glycosylphosphatidyl inositol-anchored proteins and fyn kinase assemble in noncaveolar plasma membrane microdomains defined by reggie-1 and -2. Mol. Biol. Cell.

[68] Kotani N., Nakano T., Ida Y., Ito R., Hashizume M., Yamaguchi A., Seo M., Araki T., Hojo Y., Honke K. (2018). Analysis of lipid raft molecules in the living brain slices. Neurochem. Int..

[69] Wang Z., Schey K.L. (2015). Proteomic Analysis of Lipid Raft-Like Detergent-Resistant Membranes of Lens Fiber Cells. Invest. Ophthalmol. Vis. Sci..

[70] Chowdhury S.M., Zhu X., Aloor J.J., Azzam K.M., Gabor K.A., Ge W., Addo K.A., Tomer K.B., Parks J.S., Fessler M.B. (2015). Proteomic Analysis of ABCA1-Null Macrophages Reveals a Role for Stomatin-Like Protein-2 in Raft Composition and Toll-Like Receptor Signaling. Mol. Cell Proteomics.

[71] Dwyer D.F., Barrett N.A., Austen K.F., Consortium I.G.P. (2016). Expression profiling of constitutive mast cells reveals a unique identity within the immune system. Nat. Immunol..

[72] Gschwandtner M., Paulitschke V., Mildner M., Brunner P.M., Hacker S., Eisenwort G., Sperr W.R., Valent P., Gerner C., Tschachler E. (2017). Proteome analysis identifies L1CAM/CD171 and DPP4/CD26 as novel markers of human skin mast cells. Allergy.

[73] Solstad T., Bjørgo E., Koehler C.J., Strozynski M., Torgersen K.M., Taskén K., Thiede B. (2010). Quantitative proteome analysis of detergent-resistant membranes identifies the differential regulation of protein kinase C isoforms in apoptotic T cells. Proteomics.

[74] Moltu K., Bjorgo E., Solstad T., Berge T., Thiede B., Taskén K. (2013). A proteomic approach to screening of dynamic changes in detergent-resistant membranes from activated human primary T cells. J. Proteomics Bioinform..

[75] Resh M.D. (2016). Fatty acylation of proteins: The long and the short of it. Prog. Lipid Res..

[76] Lin Q., London E. (2013). Transmembrane protein (perfringolysin o) association with ordered membrane domains (rafts) depends upon the raft-associating properties of protein-bound sterol. Biophys. J..

[77] De Gassart A., Geminard C., Fevrier B., Raposo G., Vidal M. (2003). Lipid raft-associated protein sorting in exosomes. Blood.

[78] Diaz-Rohrer B., Levental K.R., Levental I. (2014). Rafting through traffic: Membrane domains in cellular logistics. Biochim. Biophys. Acta.

[79] Salaün C., James D.J., Chamberlain L.H. (2004). Lipid rafts and the regulation of exocytosis. Traffic.

[80] Varshney P., Yadav V., Saini N. (2016). Lipid rafts in immune signalling: Current progress and future perspective. Immunology.

[81] Scorrano L., De Matteis M.A., Emr S., Giordano F., Hajnóczky G., Kornmann B., Lackner L.L., Levine T.P., Pellegrini L., Reinisch K. (2019). Coming together to define membrane contact sites. Nat. Commun..

[82] Kim B.W., Lee J.W., Choo H.J., Lee C.S., Jung S.Y., Yi J.S., Ham Y.M., Lee J.H., Hong J., Kang M.J. (2010). Mitochondrial oxidative phosphorylation system is recruited to detergent-resistant lipid rafts during myogenesis. Proteomics.

[83] Dubois L., Ronquist K.K., Ek B., Ronquist G., Larsson A. (2015). Proteomic Profiling of Detergent Resistant Membranes (Lipid Rafts) of Prostasomes. Mol. Cell Proteomics.

[84] Quintana A., Schwindling C., Wenning A.S., Becherer U., Rettig J., Schwarz E.C., Hoth M. (2007). T cell activation requires mitochondrial translocation to the immunological synapse. Proc. Natl. Acad. Sci. USA.

[85] Cascianelli G., Villani M., Tosti M., Marini F., Bartoccini E., Magni M.V., Albi E. (2008). Lipid microdomains in cell nucleus. Mol. Biol. Cell..

[86] Gómez-Llobregat J., Buceta J., Reigada R. (2013). Interplay of cytoskeletal activity and lipid phase stability in dynamic protein recruitment and clustering. Sci. Rep..

[87] Shelby S.A., Veatch S.L., Holowka D.A., Baird B.A. (2016). Functional nanoscale coupling of Lyn kinase with IgE-FcεRI is restricted by the actin cytoskeleton in early antigen-stimulated signaling. Mol. Biol. Cell.

[88] Suzuki R., Leach S., Liu W., Ralston E., Scheffel J., Zhang W., Lowell C.A., Rivera J. (2014). Molecular editing of cellular responses by the high-affinity receptor for IgE. Science.

[89] Surma M.A., Klose C., Simons K. (2012). Lipid-dependent protein sorting at the trans-Golgi network. Biochim. Biophys. Acta.

[90] Iaea D.B., Maxfield F.R. (2017). Membrane order in the plasma membrane and endocytic recycling compartment. PLoS ONE.

[91] Bissig C., Gruenberg J. (2013). Lipid sorting and multivesicular endosome biogenesis. Cold Spring Harb. Perspect. Biol..

[92] Wernersson S., Pejler G. (2014). Mast cell secretory granules: Armed for battle. Nat. Rev. Immunol..

[93] Blank U., Madera-Salcedo I.K., Danelli L., Claver J., Tiwari N., Sánchez-Miranda E., Vázquez-Victorio G., Ramírez-Valadez K.A., Macias-Silva M., González-Espinosa C. (2014). Vesicular trafficking and signaling for cytokine and chemokine secretion in mast cells. Front. Immunol..

[94] Kato N., Nakanishi M., Hirashima N. (2003). Cholesterol depletion inhibits store-operated calcium currents and exocytotic membrane fusion in RBL-2H3 cells. Biochemistry.

[95] Draber P., Halova I., Polakovicova I., Kawakami T. (2016). Signal transduction and chemotaxis in mast cells. Eur. J. Pharmacol..

[96] Psatha M., Koffer A., Erent M., Moss S.E., Bolsover S. (2004). Calmodulin spatial dynamics in RBL-2H3 mast cells. Cell Calcium..

[97] Bastan R., Peirce M.J., Peachell P.T. (2001). Regulation of immunoglobulin E-mediated secretion by protein phosphatases in human basophils and mast cells of skin and lung. Eur. J. Pharmacol..

[98] Grochowy G., Hermiston M.L., Kuhny M., Weiss A., Huber M. (2009). Requirement for CD45 in fine-tuning mast cell responses mediated by different ligand-receptor systems. Cell Signal..

[99] Fowlkes V., Wilson C.G., Carver W., Goldsmith E.C. (2013). Mechanical loading promotes mast cell degranulation via RGD-integrin dependent pathways. J. Biomech..

[100] Kassas A., Moura I.C., Yamashita Y., Scheffel J., Guérin-Marchand C., Blank U., Sims P.J., Wiedmer T., Monteiro R.C., Rivera J. (2014). Regulation of the tyrosine phosphorylation of Phospholipid Scramblase 1 in mast cells that are stimulated through the high-affinity IgE receptor. PLoS ONE.

[101] Cruse G., Metcalfe D.D., Olivera A. (2014). Functional deregulation of KIT: Link to mast cell proliferative diseases and other neoplasms. Immunol. Allergy Clin. North. Am..

[102] Yang T., Xie Z., Li H., Yue L., Pang Z., MacNeil A.J., Tremblay M.L., Tang J.T., Lin T.J. (2016). Protein tyrosine phosphatase 1B (PTP1B) is dispensable for IgE-mediated cutaneous reaction in vivo. Cell Immunol..

[103] Halova I., Draber P. (2016). Tetraspanins and Transmembrane Adaptor Proteins As Plasma Membrane Organizers-Mast Cell Case. Front. Cell Dev. Biol..

[104] Dráberová L., Shaik G.M., Volná P., Heneberg P., Tůmová M., Lebduska P., Korb J., Dráber P. (2007). Regulation of Ca2+ signaling in mast cells by tyrosine-phosphorylated and unphosphorylated non-T cell activation linker. J. Immunol..

[105] Tůmová M., Koffer A., Simíček M., Dráberová L., Dráber P. (2010). The transmembrane adaptor protein NTAL signals to mast cell cytoskeleton via the small GTPase Rho. Eur. J. Immunol..

[106] Ron D., Chen C.H., Caldwell J., Jamieson L., Orr E., Mochly-Rosen D. (1994). Cloning of an intracellular receptor for protein kinase C: A homolog of the beta subunit of G proteins. Proc. Natl. Acad. Sci. USA.

[107] Ron D., Adams D.R., Baillie G.S., Long A., O’Connor R., Kiely P.A. (2013). RACK1 to the future–A historical perspective. Cell Commun. Signal..

[108] Ballek O., Valečka J., Dobešová M., Broučková A., Manning J., Řehulka P., Stulík J., Filipp D. (2016). TCR Triggering Induces the Formation of Lck-RACK1-Actinin-1 Multiprotein Network Affecting Lck Redistribution. Front. Immunol..

[109] Li S., Esterberg R., Lachance V., Ren D., Radde-Gallwitz K., Chi F., Parent J.L., Fritz A., Chen P. (2011). Rack1 is required for Vangl2 membrane localization and planar cell polarity signaling while attenuating canonical Wnt activity. Proc. Natl. Acad. Sci. USA.

[110] Sander L.E., Frank S.P., Bolat S., Blank U., Galli T., Bigalke H., Bischoff S.C., Lorentz A. (2008). Vesicle associated membrane protein (VAMP)-7 and VAMP-8, but not VAMP-2 or VAMP-3, are required for activation-induced degranulation of mature human mast cells. Eur. J. Immunol..

[111] Puri N., Roche P.A. (2008). Mast cells possess distinct secretory granule subsets whose exocytosis is regulated by different SNARE isoforms. Proc. Natl. Acad. Sci. USA.

[112] Guo Z., Turner C., Castle D. (1998). Relocation of the t-SNARE SNAP-23 from lamellipodia-like cell surface projections regulates compound exocytosis in mast cells. Cell.

[113] Brochetta C., Suzuki R., Vita F., Soranzo M.R., Claver J., Madjene L.C., Attout T., Vitte J., Varin-Blank N., Zabucchi G. (2014). Munc18-2 and syntaxin 3 control distinct essential steps in mast cell degranulation. J. Immunol..

[114] Wilson J.D., Shelby S.A., Holowka D., Baird B. (2016). Rab11 Regulates the Mast Cell Exocytic Response. Traffic.

[115] Azouz N.P., Matsui T., Fukuda M., Sagi-Eisenberg R. (2012). Decoding the regulation of mast cell exocytosis by networks of Rab GTPases. J. Immunol..

[116] Singh R.K., Mizuno K., Wasmeier C., Wavre-Shapton S.T., Recchi C., Catz S.D., Futter C., Tolmachova T., Hume A.N., Seabra M.C. (2013). Distinct and opposing roles for Rab27a/Mlph/MyoVa and Rab27b/Munc13-4 in mast cell secretion. FEBS J..

[117] Madera-Salcedo I.K., Danelli L., Tiwari N., Dema B., Pacreau E., Vibhushan S., Birnbaum J., Agabriel C., Liabeuf V., Klingebiel C. (2018). Tomosyn functions as a PKCδ-regulated fusion clamp in mast cell degranulation. Sci. Signal..

[118] Nika K., Acuto O. (2015). Membrane nanodomains in T-cell antigen receptor signalling. Essays Biochem..

[119] Kovárová M., Tolar P., Arudchandran R., Dráberová L., Rivera J., Dráber P. (2001). Structure-function analysis of Lyn kinase association with lipid rafts and initiation of early signaling events after Fcepsilon receptor I aggregation. Mol. Cell Biol..

[120] Sada K., Zhang J., Siraganian R.P. (2001). SH2 domain-mediated targeting, but not localization, of Syk in the plasma membrane is critical for FcepsilonRI signaling. Blood.

[121] Filho E.G., da Silva E.Z., Zanotto C.Z., Oliver C., Jamur M.C. (2016). Cross-Linking Mast Cell Specific Gangliosides Stimulates the Release of Newly Formed Lipid Mediators and Newly Synthesized Cytokines. Mediators Inflamm..

[122] Lacerda Pigosso L., Baeza L.C., Vieira Tomazett M., Batista Rodrigues Faleiro M., Brianezi Dignani de Moura V.M., Melo Bailão A., Borges C.L., Alves Parente Rocha J., Rocha Fernandes G., Gauthier G.M. (2017). Paracoccidioides brasiliensis presents metabolic reprogramming and secretes a serine proteinase during murine infection. Virulence.

[123] Li G.Z., Vissers J.P., Silva J.C., Golick D., Gorenstein M.V., Geromanos S.J. (2009). Database searching and accounting of multiplexed precursor and product ion spectra from the data independent analysis of simple and complex peptide mixtures. Proteomics.

[124] Bradford M.M. (1976). A rapid and sensitive method for the quantitation of microgram quantities of protein utilizing the principle of protein-dye binding. Anal. Biochem..

[125] Hornbeck P.V., Kornhauser J.M., Tkachev S., Zhang B., Skrzypek E., Murray B., Latham V., Sullivan M. (2012). PhosphoSitePlus: A comprehensive resource for investigating the structure and function of experimentally determined post-translational modifications in man and mouse. Nucleic Acids Res..

[126] Blanc M., David F.P.A., van der Goot F.G. (2019). SwissPalm 2: Protein S-Palmitoylation Database. Methods Mol. Biol..

[127] Maurer-Stroh S., Koranda M., Benetka W., Schneider G., Sirota F.L., Eisenhaber F. (2007). Towards complete sets of farnesylated and geranylgeranylated proteins. PLoS Comput. Biol..

[128] Maurer-Stroh S., Gouda M., Novatchkova M., Schleiffer A., Schneider G., Sirota F.L., Wildpaner M., Hayashi N., Eisenhaber F. (2004). MYRbase: Analysis of genome-wide glycine myristoylation enlarges the functional spectrum of eukaryotic myristoylated proteins. Genome Biol..

[129] Pierleoni A., Martelli P.L., Casadio R. (2008). PredGPI: A GPI-anchor predictor. BMC Bioinformatics.

[130] Krogh A., Larsson B., von Heijne G., Sonnhammer E.L. (2001). Predicting transmembrane protein topology with a hidden Markov model: Application to complete genomes. J. Mol. Biol..

[131] Tulumello D.V., Deber C.M. (2012). Efficiency of detergents at maintaining membrane protein structures in their biologically relevant forms. Biochim. Biophys. Acta.

