# Supplementary material for: Proteomic Analysis of Lipid Rafts from RBL-2H3 Mast Cells
Source: Int J Mol Sci. 2019 Aug 11;20(16):3904. doi: 10.3390/ijms20163904 (PMC6720779; doi:10.3390/ijms20163904)
Supplement: Supplementary file 1 [file ijms-20-03904-s001.zip › Figure S1.pdf]

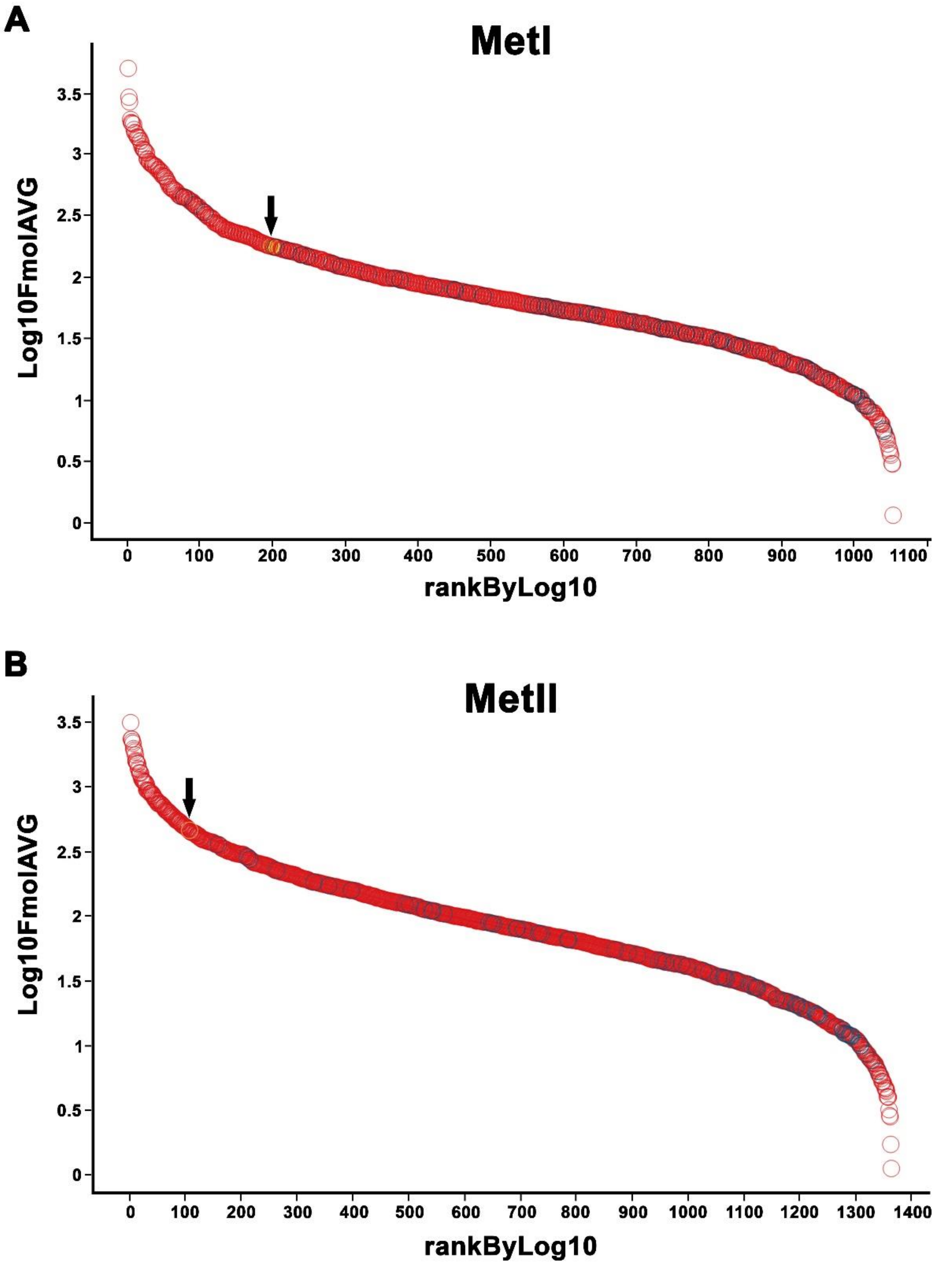

Figure S1. Dynamic range of the proteomic analysis. The graphs show the dynamic range from proteomic experiments for MetI (A) and MetII (B). Regular (red circles), reverse (blue circles) and internal standard (yellow circle; arrow) proteins are indicated. The regular and reverse proteins were identified using regular and reverse genomic databases from *Rattus norvegicus*. The yeast protein enolase was used as an internal standard.
