# Supplementary material for: Proteomic Analysis of Lipid Rafts from RBL-2H3 Mast Cells
Source: Int J Mol Sci. 2019 Aug 11;20(16):3904. doi: 10.3390/ijms20163904 (PMC6720779; doi:10.3390/ijms20163904)
Supplement: Supplementary file 1 [file ijms-20-03904-s001.zip › Figure S2.pdf]

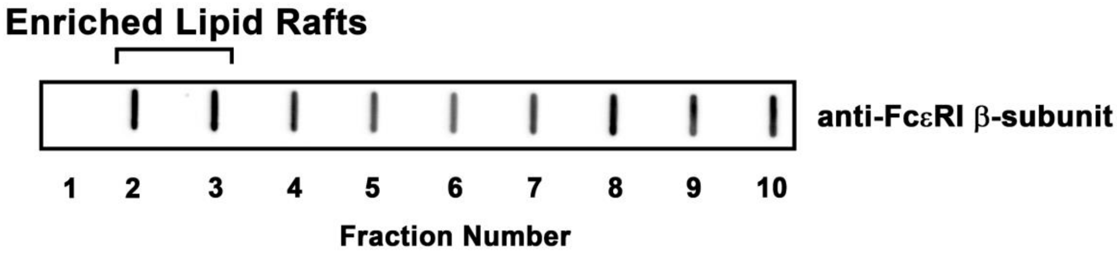

Figure S2. Immuno-blot analysis of the β-subunit of FcεRI from RBL-2H3 MC lipid rafts. Lysates of RBL-2H3 MCs were fractionated by sucrose density gradient ultracentrifugation, and fractions were immunoblotted using a pool of antibodies against the β-subunit of FcεRI. The β-subunit is found in all fractions. Data is representative of 3 independent experiments.
