# Supplementary material for: Proteomic Analysis of Lipid Rafts from RBL-2H3 Mast Cells
Source: Int J Mol Sci. 2019 Aug 11;20(16):3904. doi: 10.3390/ijms20163904 (PMC6720779; doi:10.3390/ijms20163904)
Supplement: Supplementary file 1 [file ijms-20-03904-s001.zip › Figure S3.pdf]

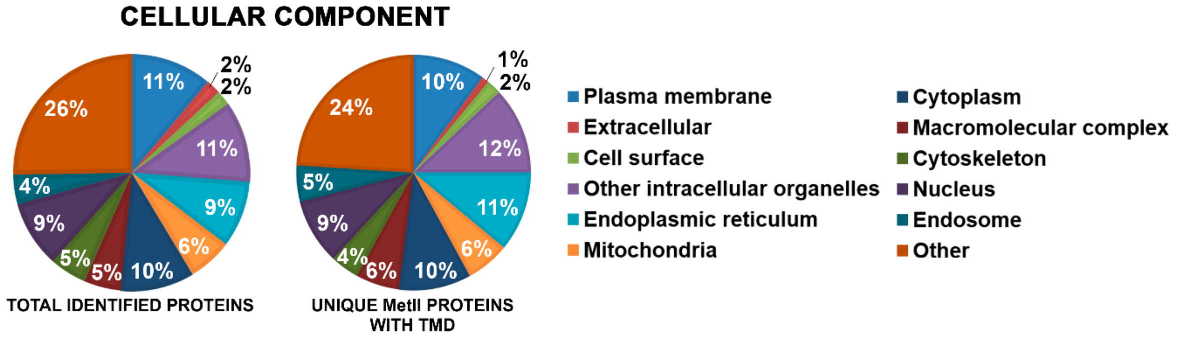

Figure S3: Total identified proteins and unique MetII proteins with transmembrane domains (TMD) have a similar distribution in the Cellular Component GO class. Total identified proteins and unique MetII proteins with TMD were grouped according to the cellular component class of GO. The data is expressed as percentage of proteins associated with each GO term.
