# Supplementary material for: Proteomic Analysis of Lipid Rafts from RBL-2H3 Mast Cells
Source: Int J Mol Sci. 2019 Aug 11;20(16):3904. doi: 10.3390/ijms20163904 (PMC6720779; doi:10.3390/ijms20163904)
Supplement: Supplementary file 1 [file ijms-20-03904-s001.zip › Table S1.pdf]

Supplementary Table 1. Detailed annotation of proteins identified in Method I

Protein ID	Protein accession	Protein description	Gene name	MW [kDa]	Score AVG	Peptides AVG
1433B_RAT	P35213	14-3-3 protein beta/alpha	Ywhab	28.05	11223.23	11
1433E_RAT	P62260	14-3-3 protein epsilon	Ywhae	29.17	26648.94	15
1433F_RAT	P68511	14-3-3 protein eta	Ywhah	28.21	9444.25	10
1433G_RAT	P61983	14-3-3 protein gamma	Ywhag	28.30	16888.38	10
1433T_RAT	P68255	14-3-3 protein theta	Ywhaq	27.78	6448.93	10
1433Z_RAT	P63102	14-3-3 protein zeta/delta	Ywhaz	27.77	51663.05	12
4F2_RAT	Q794F9	4F2 cell-surface antigen heavy chain	Slc3a2	58.07	3739.78	8
6PGD_RAT	P85968	6-phosphogluconate dehydrogenase decarboxylating	Pgd	53.24	1909.47	9
6PGL_RAT	P85971	6-phosphogluconolactonase	Pgls	27.23	21556.29	8
A0JN30_RAT	A0JN30	Canopy 2 homolog	Cnpy2	20.71	3486.07	6
A3RLA8_RAT	A3RLA8	Low affinity immunoglobulin gamma Fc region receptor II	Fcgr2b	37.97	1010.58	5
AATC_RAT	P13221	Aspartate aminotransferase cytoplasmic	Got1	46.43	1457.99	7
AATM_RAT	P00507	Aspartate aminotransferase mitochondrial	Got2	47.31	3826.02	11
ABCF1_RAT	Q6MG08	ATP-binding cassette sub-family F member 1	Abcf1	95.25	412.00	13
ACON_RAT	Q9ER34	Aconitate hydratase mitochondrial	Aco2	85.43	1156.77	10
ACTB_RAT	P60711	Actin cytoplasmic 1	Actb	41.74	71387.43	25
ACTH_RAT	P63269	Actin gamma-enteric smooth muscle	Actg2	41.88	36706.60	17
ADA_RAT	Q920P6	Adenosine deaminase	Ada	39.90	1325.03	8
ADT1_RAT	Q05962	ADP/ATP translocase 1	Slc25a4	32.99	5567.02	12
ADT2_RAT	Q09073	ADP/ATP translocase 2	Slc25a5	32.90	9236.08	10
AK1A1_RAT	P51635	Alcohol dehydrogenase [NADP(+)]	Akr1a1	36.51	9758.76	11
ALDOA_RAT	P05065	Fructose-bisphosphate aldolase A	Aldoa	39.35	6507.14	15
ALDR_RAT	P07943	Aldose reductase	Akr1b1	35.80	2510.29	12
AN32B_RAT	Q9EST6	Acidic leucine-rich nuclear phosphoprotein 32 family member B	Anp32b	31.06	6097.84	4
AN32E_RAT	Q5XIE0	Acidic leucine-rich nuclear phosphoprotein 32 family member E	Anp32e	29.42	1731.89	4
ANXA1_RAT	P07150	Annexin A1	Anxa1	38.83	28648.83	22
ANXA2_RAT	Q07936	Annexin A2	Anxa2	38.68	2992.42	10
ANXA5_RAT	P14668	Annexin A5	Anxa5	35.75	1609.09	9
ARL8B_RAT	Q66HA6	ADP-ribosylation factor-like protein 8B	Arl8b	21.54	1344.18	4
ASNS_RAT	P49088	Glutamine-dependent asparagine synthetase	Asns	64.25	2279.61	9
AT1A1_RAT	P06685	Sodium/potassium-transporting ATPase subunit alpha-1	Atp1a1	113.05	3308.45	25
AT1A2_RAT	P06686	Sodium/potassium-transporting ATPase subunit alpha-2	Atp1a2	112.22	786.52	18
AT1A3_RAT	P06687	Sodium/potassium-transporting ATPase subunit alpha-3	Atp1a3	111.69	2489.10	14

AT1A4_RAT	Q64541	Sodium/potassium-transporting ATPase subunit alpha-4	Atp1a4	114.01	228.37	10
AT1B3_RAT	Q63377	Sodium/potassium-transporting ATPase subunit beta-3	Atp1b3	31.83	1201.31	3
ATP5H_RAT	P31399	ATP synthase subunit D mitochondrial	Atp5pd	18.76	4450.09	5
ATP5I_RAT	P29419	ATP synthase subunit E mitochondrial	Atp5me	8.26	9619.80	1
ATP5J_RAT	P21571	ATP synthase-coupling factor 6 mitochondrial	Atp5pf	12.49	11843.67	4
ATPA_RAT	P15999	ATP synthase subunit alpha mitochondrial	Atp5f1a	59.75	23316.71	19
ATPB_RAT	P10719	ATP synthase subunit beta mitochondrial	Atp5f1b	56.35	30347.52	20
ATPO_RAT	Q06647	ATP synthase subunit O mitochondrial	Atp5po	23.40	11373.30	7
B0BN81_RAT	B0BN81	Ribosomal protein S5	Rps5	22.91	918.98	5
B0BNG3_RAT	B0BNG3	Vesicular integral-membrane protein VIP36	Lman2	40.39	3280.38	12
B0BNJ1_RAT	B0BNJ1	Sorcin	Sri	21.62	7389.84	7
B0K010_RAT	B0K010	Thioredoxin domain-containing 17	Txndc17	14.09	5852.90	4
B0K031_RAT	B0K031	60S ribosomal protein L7, isoform	Rpl7	30.31	1423.62	5
B2GUV5_RAT	B2GUV5	V-type proton ATPase subunit G 1	Atp6v1g1	13.71	1621.28	2
B2GUZ9_RAT	B2GUZ9	Fam49b protein	Fam49b	36.78	3721.41	10
B2GV72_RAT	B2GV72	Carbonyl reductase 3	Cbr3	30.84	2001.98	7
B2GV96_RAT	B2GV96	Coiled-coil domain containing 115	Ccdc115	19.82	854.01	5
B2GVB9_RAT	B2GVB9	Fermt3 protein	Fermt3	75.62	1507.74	10
B2MG_RAT	P07151	Beta-2-microglobulin	B2m	13.72	2979.99	3
B2RYC9_RAT	B2RYC9	Glucosylceramidase	Gba	57.49	834.24	8
B2RZ27_RAT	B2RZ27	SH3 domain-binding glutamic acid-rich-like protein 3	Sh3bgrl3	10.48	10096.01	3
B2RZD1_RAT	B2RZD1	Protein transport protein Sec61 subunit beta	Sec61b	9.99	4644.72	2
B5DEN5_RAT	B5DEN5	Eukaryotic translation elongation factor 1 beta 2	Eef1b2	24.68	2100.89	8
BAG6_RAT	Q6MG49	Large proline-rich protein BAG6	Bag6	120.01	616.14	10
BASI_RAT	P26453	Basigin	Bsg	42.44	6552.17	6
BASP1_RAT	Q05175	Brain acid soluble protein 1	Basp1	21.79	8583.31	5
C1QBP_RAT	O35796	Complement component 1 Q subcomponent-binding protein mitochondrial	C1qbp	31.00	8810.01	4
CAH2_RAT	P27139	Carbonic anhydrase 2	Ca2	29.11	14786.58	7
CALM_RAT	P0DP29	Calmodulin-1	Calm1	16.84	16895.13	6
CALR_RAT	P18418	Calreticulin	Calr	48.00	18376.84	15
CALX_RAT	P35565	Calnexin	Canx	67.26	1491.23	11
CAP1_RAT	Q08163	Adenylyl cyclase-associated protein 1	Cap1	51.59	3944.03	8
CAPG_RAT	Q6AYC4	Macrophage-capping protein	Capg	38.80	3876.14	7
CAPZB_RAT	Q5XI32	F-actin-capping protein subunit beta	Capzb	30.63	2134.28	8
CAZA2_RAT	Q3T1K5	F-actin-capping protein subunit alpha-2	Capza2	32.97	1161.94	5
CD48_RAT	P10252	CD48 antigen	Cd48	27.68	943.18	7
CDC37_RAT	Q63692	Hsp90 co-chaperone Cdc37	Cdc37	44.51	5308.82	5
CDC42_RAT	Q8CFN2	Cell division control protein 42 homolog (CDC42)	Cdc42	21.26	8806.95	5

CDV3_RAT	Q5XIM5	Protein CDV3 homolog	Cdv3	24.31	1885.86	5
CH10_RAT	P26772	10 kDa heat shock protein mitochondrial	Hspe1	10.90	17415.36	8
CISY_RAT	Q8VHF5	Citrate synthase mitochondrial	Cs	51.87	2329.63	9
CLH1_RAT	P11442	Clathrin heavy chain 1	Cltc	191.60	409.21	24
CLIC1_RAT	Q6MG61	Chloride intracellular channel protein 1	Clic1	26.98	24293.77	11
CNBP_RAT	P62634	Cellular nucleic acid-binding protein	Cnbp	19.46	3759.30	3
CNTN1_RAT	Q63198	Contactin 1	Cntn1	113.50	519.19	20
COF1_RAT	P45592	Cofilin-1	Cfl1	18.53	35166.62	5
COR1A_RAT	Q91ZN1	Coronin-1A	Coro1a	51.07	4032.92	11
COTL1_RAT	B0BNA5	Coactosin-like protein	Cotl1	15.93	27708.91	9
COX41_RAT	P10888	Cytochrome c oxidase subunit 4 isoform 1 mitochondrial	Cox4i1	19.52	1971.84	8
COX5A_RAT	P11240	Cytochrome c oxidase subunit 5A mitochondrial	Cox5a	16.13	5776.28	5
COX5B_RAT	P12075	Cytochrome c oxidase subunit 5B mitochondrial	Cox5b	13.92	4351.65	6
CP20A_RAT	Q6P7D4	Cytochrome P450 20A1	Cyp20a1	52.00	2134.28	14
CX6C2_RAT	P11951	Cytochrome c oxidase subunit 6C2	Cox6c2	8.46	4565.52	4
CYBP_RAT	Q6AYK6	Calcyclin-binding protein	Cacybp	26.54	1038.41	7
CYC_RAT	P62898	Cytochrome c somatic	Cycs	11.61	8475.21	6
D3ZD09_RAT	D3ZD09	Cytochrome c oxidase subunit 6B1	Cox6b1	10.07	3236.07	7
D3ZGM7_RAT	D3ZGM7	Peptidase inhibitor 16	Pi16	52.15	747.31	3
D3ZN21_RAT	D3ZN21	ATP-dependent RNA helicase DDX3Y	Ddx3y	72.99	1314.29	13
D3ZNQ6_RAT	D3ZNQ6	NEDD8-conjugating enzyme Ubc12	Ube2m	20.90	4121.65	6
D3ZPP2_RAT	D3ZPP2	ADP-ribosylation factor-like protein 8A	Arl8a	21.39	1338.81	5
D3ZPR0_RAT	D3ZPR0	Exportin-2	Cse1l	110.21	805.23	11
D3ZRN3_RAT	D3ZRN3	Beta-actin-like protein 2	Actbl2	41.96	20353.75	13
D3ZSA9_RAT	D3ZSA9	Nodal modulator 1	Nomo1	133.44	750.75	16
D3ZV75_RAT	D3ZV75	Major facilitator superfamily domain-containing protein 1	Mfsd1	51.20	583.26	1
D3ZYS7_RAT	D3ZYS7	Ras GTPase-activating protein 1	G3bp1	51.79	876.93	6
D3ZYW2_RAT	Q499R8	Heterogeneous nuclear ribonucleoprotein H	Hnrnph1	20.58	3022.02	9
D3ZZR9_RAT	D3ZZR9	Peptidylprolyl isomerase	Fkbp2	15.39	7827.55	4
D4A0Y9_RAT	D4A0Y9	Phosphatidylcholine translocator ABCB4	Abcb1b	141.05	356.53	8
D4A133_RAT	D4A133	V-type proton ATPase catalytic subunit A	Atp6v1a	68.27	963.97	17
D4A1D8_RAT	D4A1D8	Surfeit 4	Surf4	30.38	1071.58	3
D4A3S8_RAT	D4A3S8	NOL1/NOP2/Sun domain family member 2	Nsun2	88.09	666.64	10
D4A6A2_RAT	D4A6A2	Heterogeneous nuclear ribonucleoprotein A3-like	LOC100911361	36.91	1700.26	7
D4A720_RAT	D4A720	Serine/arginine-rich splicing factor 7 isoform 2	Srsf7	27.38	2400.56	5
D4A7U1_RAT	D4A7U1	Zyxin	Zyx	60.30	2967.96	8
D4A7U6_RAT	D4A7U6	U6 snRNA-associated Sm-like protein LSm3	Lsm3	11.85	1542.28	2
D4A9L2_RAT	D4A9L2	Serine/arginine-rich splicing factor 1	Srsf1	27.75	4640.91	7

D4A9X7_RAT	D4A9X7	Hypermethylated in cancer 2 protein	Hic2	67.20	320.60	4
D4AAE9_RAT	D4AAE9	CDGSH iron-sulfur domain-containing protein 2	Cisd2	15.29	4059.60	2
D4ADD7_RAT	D4ADD7	Glutaredoxin 5	Glrx5	16.43	7892.59	2
D4ADS9_RAT	D4ADS9	EFR3 homolog A	Efr3a	92.52	558.92	13
D4AEG2_RAT	D4AEG2	Ras-related protein Rab-32	Rab32	25.26	883.24	5
D4AEP0_RAT	D4AEP0	Adenylosuccinate synthetase isozyme 2	Adss	50.09	2287.64	6
DHSA_RAT	Q920L2	Succinate dehydrogenase [ubiquinone] flavoprotein subunit mitochondrial	Sdha	71.62	3794.74	13
DOPD_RAT	P80254	D-dopachrome decarboxylase	Ddt	13.13	650.05	3
DUT_RAT	P70583	Deoxyuridine 5'-triphosphate nucleotidohydrolase	Dut	22.00	2153.78	5
DX39B_RAT	Q63413	Spliceosome RNA helicase Ddx39b	Ddx39b	49.04	1057.85	9
E9PSV5_RAT	E9PSV5	Phosphoserine aminotransferase	Psat1	40.49	1062.32	7
E9PT29_RAT	E9PT29	ATP-dependent RNA helicase DDX17	Ddx17	72.46	2327.21	10
E9PT65_RAT	E9PT65	Radixin	Rdx	68.49	645.19	8
E9PTW1_RAT	E9PTW1	Secretory carrier-associated membrane protein	Scamp3	38.50	289.38	5
E9PTX9_RAT	E9PTX9	Solute carrier family 12 member 2	Slc12a2	130.37	1782.78	12
E9PU78_RAT	E9PU78	Isoform Bcl-X(S) of Bcl-2-like protein 1	---	19.06	348.72	3
EF1A1_RAT	P62630	Elongation factor 1-alpha 1	Eef1a1	50.11	16649.03	11
EF1G_RAT	Q68FR6	Elongation factor 1-gamma	Eef1g	50.06	1306.83	8
EF2_RAT	P05197	Elongation factor 2	Eef2	95.28	17558.08	34
ENOA_RAT	P04764	Alpha-enolase	Eno1	47.13	67299.46	25
ENOB_RAT	P15429	Beta-enolase	Eno3	47.01	12346.33	12
ENPL_RAT	Q66HD0	Endoplasmin	Hsp90b1	92.77	8923.66	26
ERO1A_RAT	Q8R4A1	ERO1-like protein alpha	Ero1a	54.02	1337.72	9
ERP29_RAT	P52555	Endoplasmic reticulum resident protein 29	Erp29	28.58	5384.18	4
EZRI_RAT	P31977	Ezrin	Ezr	69.39	3834.35	13
F10A1_RAT	P50503	Hsc70-interacting protein	St13	41.28	3653.34	7
F1LM33_RAT	F1LM33	Leucine-rich PPR motif-containing protein mitochondrial isoform	Lrpprc	156.68	666.82	18
F1LM66_RAT	F1LM66	116 kDa U5 small nuclear ribonucleoprotein component	Eftud2	109.35	1261.28	7
F1LM82_RAT	F1LM82	Heterogeneous nuclear ribonucleoproteins A2/B1	Hnrnpa2b1	32.47	6670.34	10
F1LMM5_RAT	F1LMM5	Arachidonate 5-lipoxygenase	Alox5	78.13	1841.63	16
F1LMP7_RAT	F1LMP7	Granulins	Grn	56.65	838.66	7
F1LPV0_RAT	F1LPV0	Asparagine--tRNA ligase, cytoplasmic	Nars	64.13	788.27	9
F1LQ81_RAT	F1LQ81	Vesicle-fusing ATPase	Nsf	82.63	4215.60	17
F1LQJ7_RAT	F1LQJ7	Phosphoenolpyruvate carboxykinase [GTP], mitochondrial	Pck2	71.21	471.15	8
F1LRK1_RAT	F1LRK1	Sodium/potassium-transporting ATPase subunit alpha	Atp4a	114.04	92.21	11
F1LSN4_RAT	F1LSN4	Fibroblast growth factor receptor	Fgfr3	87.67	130.74	10
F1LVV4_RAT	F1LVV4	Protein RCC2	Rcc2	46.65	1350.43	7
F1LZW6_RAT	F1LZW6	Solute carrier family 25 member 13	Slc25a13	54.10	1622.14	13

F1M0G0_RAT	F1M0G0	Regulator of G-protein signaling 17	Rgs17	24.32	1702.56	6
F1M0J3_RAT	F1M0J3	Sodium bicarbonate cotransporter 3, isophorm 4	Slc4a7	126.85	406.78	11
F1M2K3_RAT	F1M2K3	Small ubiquitin-related modifier	Sumo2	10.77	10781.10	1
F1M2P8_RAT	F1M2P8	Protein kinase C alpha	Prkca	72.77	335.43	9
F1M4T3_RAT	B2RYQ5	Enhancer of rudimentary homolog	Erh	12.26	520.09	2
F1M7T6_RAT	F1M7T6	Translocon-associated protein subunit gamma	Ssr3	7.38	976.79	2
F1M8X9_RAT	F1M8X9	Golgi brefeldin A-resistant guanine nucleotide exchange factor 1	Gbf1	206.52	487.28	14
F7EPH4_RAT	F7EPH4	Inorganic Pyrophosphatase	Ppa1	32.77	3875.81	8
F7EWC1_RAT	F7EWC1	Vasodilator-stimulated phosphoprotein	Vasp	39.49	418.42	5
F7F4S8_RAT	B2RYB8	Integrin beta 2	Itgb2	84.82	658.56	12
F7FFV2_RAT	F7FFV2	keratin 5	Krt5	44.02	1939.83	7
F7FP91_RAT	Q95II0	MHC class Ib antigen	RT1-CE5	42.23	4291.37	4
FCERB_RAT	P13386	High affinity immunoglobulin epsilon receptor subunit beta	Ms4a2	26.73	1896.05	4
FEN1_RAT	Q5XIP6	Flap endonuclease 1	Fen1	42.61	557.15	4
FGR_RAT	Q6P6U0	Tyrosine-protein kinase Fgr	Fgr	58.82	140.71	5
FKB1A_RAT	Q62658	Peptidyl-prolyl cis-trans isomerase FKBP1A	Fkbp1a	11.92	4920.10	3
FPPS_RAT	P05369	Farnesyl pyrophosphate synthase	Fdps	40.83	552.94	9
G3P_RAT	P04797	Glyceraldehyde-3-phosphate dehydrogenase	Gapdh	35.83	31578.66	10
G3V679_RAT	G3V679	Transferrin receptor protein 1	Tfrc	85.88	840.95	15
G3V6D3_RAT	G3V6D3	ATP synthase subunit beta	Atp5f1b	56.35	30347.52	6
G3V6G5_RAT	G3V6G5	F-box-like/WD repeat-containing protein TBL1X	Tbl1x	56.80	308.26	6
G3V6P7_RAT	G3V6P7	Myosin heavy chain 9	Myh9	226.41	458.29	30
G3V6P8_RAT	G3V6P8	Guanine nucleotide-binding protein gamma-12	Gng12	7.99	12311.67	3
G3V6T7_RAT	G3V6T7	Protein disulfide-isomerase A4	Pdia4	72.75	3414.59	20
G3V6W2_RAT	G3V6W2	Prolactin regulatory element-binding protein	Preb	45.37	360.95	3
G3V741_RAT	G3V741	Solute carrier family 25 member 3	Slc25a3	39.53	10300.27	9
G3V7J7_RAT	G3V7J7	Eukaryotic translation initiation factor 5A-2	Eif5a2	16.79	1875.77	6
G3V7Q7_RAT	G3V7Q7	Ras GTPase-activating-like protein IQGAP1	Iqgap1	188.83	652.63	17
G3V852_RAT	G3V852	Talin-1	Tln1	269.67	737.61	31
G3V8A5_RAT	G3V8A5	Vacuolar protein sorting-associated protein 35	Vps35	91.73	564.08	9
G3V8C4_RAT	G3V8C4	Chloride intracellular channel protein 4	Clic4	28.67	4051.87	7
G3V8G5_RAT	G3V8G5	Golgi apparatus protein 1	Glg1	133.55	545.01	9
G3V908_RAT	G3V908	keratin, type II Kb15	Kb15	57.61	1787.99	7
G3V936_RAT	G3V936	Citrate synthase	Cs	51.83	1051.28	10
G3V993_RAT	G3V993	SAM domain, SH3 domain and nuclear localization signals, 1 (SAMNS1)	Samsn1	40.73	2311.95	8
G3V9A3_RAT	G3V9A3	14-3-3 protein sigma	Sfn	27.75	2008.64	5
G3V9U7_RAT	G3V9U7	Sarcoplasmic/endoplasmic reticulum calcium ATPase 3 (SERCA3)	Atp2a3	109.37	317.82	15
GBB1_RAT	P54311	Transducin beta-1	Gnb1	37.38	310.34	10

GBB2_RAT	P54313	Transducin beta-2	Gnb2	37.33	1658.05	9
GDIA_RAT	P50398	Rab GDP dissociation inhibitor alpha	Gdi1	50.54	2846.46	9
GDIB_RAT	P50399	Rab GDP dissociation inhibitor beta	Gdi2	50.54	8115.68	11
GDIR1_RAT	Q5XI73	Rho GDP dissociation inhibitor 1	Arhgdia	23.41	45101.02	7
GELS_RAT	Q68FP1	Gelsolin	Gsn	86.07	1453.52	10
GFAP_RAT	P47819	Glial fibrillary acidic protein	Gfap	49.96	161.47	5
GLOD4_RAT	Q5I0D1	Glyoxalase domain-containing protein 4	Glod4	33.27	2020.22	8
GNAI2_RAT	P04897	Guanine nucleotide-binding protein G(i) subunit alpha-2	Gnai2	40.50	4498.67	16
GNAI3_RAT	P08753	Guanine nucleotide-binding protein G(k) subunit alpha	Gnai3	40.52	1366.32	10
GNAS1_RAT	Q63803	Guanine nucleotide-binding protein G(s) subunit alpha isoforms XLas	Gnas	122.89	1083.32	3
GNAS2_RAT	P63095	Guanine nucleotide-binding protein G(s) subunit alpha	Gnas	45.66	1301.58	8
GNAZ_RAT	P19627	Guanine nucleotide-binding protein G(z) subunit alpha	Gnaz	40.88	1912.17	8
GPDM_RAT	P35571	Glycerol-3-phosphate dehydrogenase mitochondrial	Gpd2	80.97	413.42	8
GRP75_RAT	P48721	Mortalin	Hspa9	73.86	4225.23	16
GRP78_RAT	P06761	Endoplasmic reticulum chaperone BiP	Hspa5	72.35	23724.92	28
GTR1_RAT	P11167	Solute carrier family 2, facilitated glucose transporter member 1	Slc2a1	53.96	797.33	3
GTR3_RAT	Q07647	Solute carrier family 2, facilitated glucose transporter member 3	Slc2a3	53.58	4665.32	9
H14_RAT	P15865	Histone H1.4	Hist1h1e	21.99	3178.50	6
H4_RAT	P62804	Histone H4	Hist1h4b; Hist1h4m; Hist4h4	11.37	2107.98	4
HAP28_RAT	Q62785	28 kDa heat- and acid-stable phosphoprotein	Pdap1	20.61	3318.89	3
HMCS1_RAT	P17425	Hydroxymethylglutaryl-CoA synthase cytoplasmic	Hmgcs1	57.43	1203.94	9
HS90A_RAT	P82995	Heat shock protein HSP 90-alpha	Hsp90aa1	84.82	16741.41	27
HS90B_RAT	P34058	Heat shock protein HSP 90-beta	Hsp90ab1	83.28	34505.82	32
HSP72_RAT	P14659	Heat shock-related 70 kDa protein 2	Hspa2	69.64	24993.69	16
HSP74_RAT	O88600	Heat shock 70 kDa protein 4	Hspa4	94.06	3185.07	22
HXK1_RAT	P05708	Hexokinase-1	Hk1	102.41	352.55	17
HXK2_RAT	P27881	Hexokinase-2	Hk2	102.54	79.98	6
ICK_RAT	Q62726	Serine/threonine-protein kinase ICK	Ick	70.57	130.74	3
IF4A2_RAT	Q5RKI1	Eukaryotic initiation factor 4A-II	Eif4a2	46.40	4299.29	10
IF4A3_RAT	Q3B8Q2	Eukaryotic initiation factor 4A-III	Eif4a3	46.84	709.67	7
IF5A1_RAT	Q3T1J1	Eukaryotic translation initiation factor 5A-1	Eif5a	16.83	2240.27	7
IF6_RAT	Q3KRD8	Eukaryotic translation initiation factor 6	Eif6	26.57	2731.00	5
ILEUA_RAT	Q4G075	Leukocyte elastase inhibitor	Serpinb1a	42.73	1485.99	8
ILRL1_RAT	Q62611	Interleukin 1 receptor-like 1	Il1rl1	64.41	970.66	10
IMB1_RAT	P52296	Importin subunit beta-1	Kpnb1	97.12	727.21	10
IMMT_RAT	Q3KR86	MICOS complex subunit Mic60	Immt	67.18	2906.70	13
ITFG3_RAT	Q5M7W6	Protein FAM234A	Fam234a	60.64	348.83	7
ITM2C_RAT	Q5PQL7	Integral membrane protein 2C	Itm2c	30.48	2043.92	5

K1C10_RAT	Q6IFW6	Keratin, type I cytoskeletal 10	Krt10	56.51	299.44	6
K1C14_RAT	Q6IFV1	Keratin, type I cytoskeletal 14	Krt14	52.68	662.16	7
K1C15_RAT	Q6IFV3	Keratin, type I cytoskeletal 15	Krt15	48.87	703.90	11
K1C17_RAT	Q6IFU8	Keratin, type I cytoskeletal 17	Krt17	48.12	706.33	8
K2C1_RAT	Q6IMF3	Keratin, type II cytoskeletal 1	Krt1	64.83	2068.50	11
K2C1B_RAT	Q6IG01	Keratin, type II cytoskeletal 1B	Krt77	57.26	936.67	7
K2C5_RAT	Q6P6Q2	Keratin, type II cytoskeletal 5	Krt5	61.83	1967.47	8
K2C6A_RAT	Q4FZU2	Keratin, type II cytoskeletal 6A	Krt6a	59.25	2511.33	7
K2C73_RAT	Q6IG03	Keratin, type II cytoskeletal 73	Krt73	60.39	1167.59	7
K2C75_RAT	Q6IG05	Keratin, type II cytoskeletal 75	Krt75	59.03	1766.46	9
K2C8_RAT	Q10758	Keratin, type II cytoskeletal 8	Krt8	54.02	99.78	8
KAP0_RAT	P09456	cAMP-dependent protein kinase type I-alpha regulatory subunit	Prkar1a	43.10	5804.33	8
KCY_RAT	Q4KM73	UMP-CMP kinase	Cmpk1	22.17	2624.88	6
KPYM_RAT	P11980	Pyruvate kinase PKM	Pkm	57.82	25113.64	25
KPYR_RAT	P12928	Pyruvate kinase PKLR	Pklr	62.20	1366.61	6
LAP2_RAT	Q62733	Lamina-associated polypeptide 2 isoform beta	Tmpo	50.28	2803.44	14
LAT_RAT	O70601	Linker for activation of T-cells family member 1 (LAT)	Lat	26.19	1279.26	5
LCK_RAT	Q01621	Tyrosine-protein kinase LCK	Lck	57.89	130.74	5.3
LDHA_RAT	P04642	L-lactate dehydrogenase A chain	Ldha	36.45	26968.88	17
LEG1_RAT	P11762	Galectin-1	Lgals1	14.86	7991.49	4
LPPRC_RAT	Q5SGE0	Leucine-rich PPR motif-containing protein mitochondrial	Lrpprc	156.65	996.19	18
LRC59_RAT	Q5RJR8	Leucine-rich repeat-containing protein 59	Lrrc59	34.87	1701.89	6
LTOR1_RAT	Q6P791	Ragulator complex protein LAMTOR1	Lamtor1	17.72	3092.09	5
LYN_RAT	Q07014	Tyrosine-protein kinase Lyn	Lyn	58.66	590.98	17
M0R5K9_RAT	M0R5K9	40S ribosomal protein S18	---	17.66	2436.05	5
M0R866_RAT	M0R866	Uncharacterized protein	---	27.02	1094.06	7
M0R9L0_RAT	M0R9L0	Nascent polypeptide-associated complex subunit alpha	Naca	220.20	6601.05	14
M0RAU0_RAT	M0RAU0	Advanced glycosylation end product-specific receptor	Ager	13.93	2946.14	2
M0RDJ4_RAT	M0RDJ4	Glia maturation factor beta	Gmfb	16.45	2318.48	5
MAK_RAT	P20793	Serine/threonine-protein kinase MAK	Mak	69.90	130.74	4.3
MANF_RAT	P0C5H9	Mesencephalic astrocyte-derived neurotrophic factor	Manf	20.39	1906.83	6
MARE1_RAT	Q66HR2	Microtubule-associated protein RP/EB family member 1	Mapre1	30.00	3227.68	7
MDHC_RAT	O88989	Malate dehydrogenase cytoplasmic	Mdh1	36.48	5681.27	10
MDHM_RAT	P04636	Malate dehydrogenase mitochondrial	Mdh2	35.68	29096.08	15
MK03_RAT	P21708	Mitogen-activated protein kinase 3	Mapk3	43.08	795.09	5
MOT1_RAT	P53987	Monocarboxylate transporter 1	Slc16a1	53.24	1041.20	2
MPRD_RAT	Q6AY20	Cation-dependent mannose-6-phosphate receptor	M6pr	31.10	2601.04	6
MTPN_RAT	P62775	Myotrophin	Mtpn	12.86	17148.85	1

MYL6_RAT	Q64119	Myosin light polypeptide 6	Myl6	16.98	4308.61	5
NCF2_RAT	A7E3N2	Neutrophil cytosol factor 2	Ncf2	59.57	292.81	7
NCPR_RAT	P00388	NADPH--cytochrome P450 reductase	Por	76.96	850.87	14
NDKA_RAT	Q05982	Nucleoside diphosphate kinase A	Nme1	17.19	13543.53	6
NDKB_RAT	P19804	Nucleoside diphosphate kinase B	Nme2	17.28	10973.40	5
NDUS1_RAT	Q66HF1	NADH-ubiquinone oxidoreductase 75 kDa subunit mitochondrial	Ndufs1	79.41	478.50	12
NDUV2_RAT	P19234	NADH dehydrogenase [ubiquinone] flavoprotein 2 mitochondrial	Ndufv2	27.38	1011.01	5
NEDD4_RAT	Q62940	E3 ubiquitin-protein ligase NEDD4	Nedd4	102.40	725.16	9
NHRF1_RAT	Q9JJ19	Na(+)/H(+) exchange regulatory cofactor NHE-RF1	Slc9a3r1	38.83	404.97	8
NICA_RAT	Q8CGU6	Nicastrin	Ncstn	78.40	574.06	5
NP1L4_RAT	Q5U2Z3	Nucleosome assembly protein 1-like 4	Nap1l4	43.92	586.05	3
NPM_RAT	P13084	Nucleophosmin	Npm1	32.56	2741.26	2.8
NQO1_RAT	P05982	NAD(P)H dehydrogenase [quinone] 1	Nqo1	30.95	30225.76	8
NSDHL_RAT	Q5PPL3	Sterol-4-alpha-carboxylate 3-dehydrogenase decarboxylating	Nsdhl	40.41	281.17	5
NSF1C_RAT	O35987	NSFL1 cofactor p47	Nsfl1c	40.68	2478.01	10
NTF2_RAT	P61972	Nuclear transport factor 2	Nutf2	14.48	2377.15	3
NUCL_RAT	P13383	Nucleolin	Ncl	77.15	2332.35	17
ODP2_RAT	P08461	Pyruvate dehydrogenase component E2	Dlat	67.17	986.99	8
ORN_RAT	Q5U1X1	Oligoribonuclease mitochondrial	Rexo2	26.75	617.30	7
OTUB1_RAT	B2RYG6	Ubiquitin thioesterase OTUB1	Otub1	31.27	1483.33	6
PAIRB_RAT	Q6AXS5	Plasminogen activator inhibitor 1 RNA-binding protein	Serbp1	44.75	10414.05	8
PARK7_RAT	O88767	Protein/nucleic acid deglycase DJ-1	Park7	19.97	12049.97	7
PCNA_RAT	P04961	Proliferating cell nuclear antigen	Pcna	28.75	8018.69	7
PDIA1_RAT	P04785	Protein disulfide-isomerase	P4hb	56.95	17035.55	26
PDIA3_RAT	P11598	Protein disulfide-isomerase A3	Pdia3	56.62	13336.71	26
PDIA6_RAT	Q63081	Protein disulfide-isomerase A6	Pdia6	48.17	10114.17	11
PEBP1_RAT	P31044	Phosphatidylethanolamine-binding protein 1	Pebp1	20.80	34833.09	8
PGAM1_RAT	P25113	Phosphoglycerate mutase 1	Pgam1	28.83	15635.43	9
PGH2_RAT	P35355	Prostaglandin G/H synthase 2	Ptgs2	69.16	334.63	7
PGK1_RAT	P16617	Phosphoglycerate kinase 1	Pgk1	44.54	24901.38	19
PGRC1_RAT	P70580	Membrane-associated progesterone receptor component 1	Pgrmc1	21.60	505.15	4
PHB_RAT	P67779	Prohibitin	Phb	29.82	4371.60	12
PHB2_RAT	Q5XIH7	Prohibitin 2	Phb2	33.31	1214.79	8
PLS1_RAT	P58195	Phospholipid scramblase 1	Plscr1	36.71	679.03	5
PP2AA_RAT	P63331	Serine/threonine-protein phosphatase 2A catalytic subunit alpha isoform	Ppp2ca	35.61	674.64	6
PPIA_RAT	P10111	Peptidyl-prolyl cis-trans isomerase A	Ppia	17.87	47501.08	12
PPIB_RAT	P24368	Peptidyl-prolyl cis-trans isomerase B	Ppib	23.80	6691.57	11
PRAF3_RAT	Q9ES40	PRA1 family protein 3	Arl6ip5	21.55	717.39	3

PRDX1_RAT	Q63716	Peroxiredoxin-1	Prdx1	22.11	29448.13	11
PRDX2_RAT	P35704	Peroxiredoxin-2	Prdx2	21.78	4001.95	6
PROF1_RAT	P62963	Profilin-1	Pfn1	14.96	50467.21	7
PUR9_RAT	O35567	Bifunctional purine biosynthesis protein PURH	Atic	64.21	3332.23	15
PYGL_RAT	P09811	Glycogen phosphorylase liver form	Pygl	97.48	289.66	13
Q2I6B2_RAT	Q2I6B2	V-type proton ATPase subunit A1	Atp6v0a1	95.59	1994.21	15
Q32PX6_RAT	Q32PX6	Rho-related GTP-binding protein RhoG	Rhog	21.31	12262.52	8
Q4G002_RAT	Q4G002	Histocompatibility 2 T region locus 24	RT1-T24-4	42.46	4123.62	4
Q4KM97_RAT	Q4KM97	Tyrosine-protein kinase BLK	Blk	56.65	226.26	8
Q4V7D1_RAT	Q4V7D1	Translocon-associated protein subunit alpha	Ssr1	32.21	1369.80	3
Q4V7D9_RAT	Q4V7D9	Acid sphingomyelinase-like phosphodiesterase 3B	Smpdl3b	51.65	2105.85	7
Q5M7T6_RAT	Q5M7T6	V-type proton ATPase subunit D 1	Atp6v0d1	40.30	843.55	11
Q5M860_RAT	Q5M860	Rho GDP dissociation inhibitor 2	Arhgdib	22.88	33388.18	9
Q5RJK5_RAT	Q5RJK5	Chromobox 3	Cbx3	20.81	7670.66	5
Q5U1Y2_RAT	Q5U1Y2	Ras-related C3 botulinum toxin substrate 2 (Rac2)	Rac2	21.44	9647.45	7
Q5U362_RAT	Q5U362	Annexin A4	Anxa4	35.89	1216.83	7
Q5U3Z7_RAT	Q5U3Z7	Serine hydroxymethyltransferase	Shmt2	55.77	2111.16	11
Q5XI34_RAT	Q5XI34	Serine/threonine-protein phosphatase 2A 65 kDa regulatory subunit A alpha isoform	Ppp2r1a	65.32	510.75	10
Q5XI38_RAT	Q5XI38	Lymphocyte cytosolic protein 1	Lcp1	70.12	4833.94	12
Q5XI77_RAT	Q5XI77	Annexin A11	Anxa11	54.16	2775.25	11
Q5XIM7_RAT	Q5XIM7	Lysine--tRNA ligase	Kars	71.62	319.66	10
Q5XIV1_RAT	Q5XIV1	Phosphoglycerate kinase 2	Pgk2	45.01	4224.74	9
Q63116_RAT	Q63116	Mast/stem cell growth factor receptor	Kit	109.34	274.17	12
Q68FX4_RAT	Q68FX4	Hematopoietic cell-specific LYN substrate 1 (hcls1)	Hcls1	52.82	1030.79	8
Q6AYD3_RAT	Q6AYD3	Proliferation-associated protein 2G4	Pa2g4	43.66	2398.25	11
Q6AYI1_RAT	Q6AYI1	Probable ATP-dependent RNA helicase DDX5	Ddx5	69.24	2618.88	14
Q6IFU9_RAT	Q6IFU9	keratin 16	Krt16	50.78	550.92	6
Q6IN37_RAT	Q6IN37	GM2 ganglioside activator	Gm2a	21.49	13499.93	5
Q6MGA3_RAT	Q6MGA3	Antigen peptide transporter 2	Tap2	77.75	710.30	7
Q6P3V8_RAT	Q6P3V8	Eukaryotic initiation factor 4A-I	Eif4a1	46.15	7441.99	10
Q6P685_RAT	Q6P685	Eukaryotic translation initiation factor 2 subunit 2	Eif2s2	38.24	2854.59	6
Q6P6T6_RAT	Q6P6T6	Cathepsin D	Ctsd	44.62	7200.07	10
Q6TXF3_RAT	Q6TXF3	Acyl-CoA-binding protein	Dbi	66.45	9389.79	5
Q76GL9_RAT	Q76GL9	Neutral amino acid transporter A	Slc1a4	55.90	585.50	2
Q78P65_RAT	Q78P65	Rap2A-like protein	Rap2a	11.95	1466.66	7.6
Q7TP15_RAT	Q7TP15	S-methyl-5'-thioadenosine phosphorylase	Mtap	43.08	549.50	3
Q9JKB7_RAT	Q9JKB7	Guanine deaminase	Gda	50.90	1391.87	10
Q9QX80_RAT	Q9QX80	Heterogeneous nuclear ribonucleoprotein A/B	Hnrnpab	30.85	4882.10	6

QCR1_RAT	Q68FY0	Cytochrome b-c1 complex subunit 1 mitochondrial	Uqcrc1	52.85	2700.23	9
QCR2_RAT	P32551	Cytochrome b-c1 complex subunit 2 mitochondrial	Uqcrc2	48.40	2697.00	10
QCR6_RAT	Q5M9I5	Cytochrome b-c1 complex subunit 6 mitochondrial	Uqcrh	10.42	13352.32	1
R9PXR7_RAT	B2GV92	Prostaglandin E synthase 3	Ptges3	18.72	1704.81	7
R9PXU4_RAT	R9PXU4	Thioredoxin reductase 1 cytoplasmic	Txnrd1	54.51	651.76	6
RAB15_RAT	P35289	Ras-related protein Rab-15	Rab15	24.28	441.17	3
RAB1A_RAT	Q6NYB7	Ras-related protein Rab-1a	Rab1A	22.68	18707.79	9
RAB2A_RAT	P05712	Ras-related protein Rab-2a	Rab2a	23.54	5913.19	9
RAB35_RAT	Q5U316	Ras-related protein Rab-35	Rab35	23.03	9690.18	4
RAB4B_RAT	P51146	Ras-related protein Rab-4b	Rab4b	23.63	7202.53	5
RAB7A_RAT	P09527	Ras-related protein Rab-7a	Rab7a	23.50	2888.40	8
RAB8B_RAT	P70550	Ras-related protein Rab-8b	Rab8b	23.60	8159.46	5
RAC1_RAT	Q6RUV5	Ras-related C3 botulinum toxin substrate 1 (Rac1)	Rac1	21.45	7675.92	6
RACK1_RAT	P63245	Receptor of activated protein C kinase 1 (RACK1)	Rack1	35.08	10611.07	13
RAN_RAT	P62828	GTP-binding nuclear protein Ran	Ran	24.42	25542.29	8
RAP1A_RAT	P62836	Ras-related protein Rap-1a	Rap1a	20.99	5357.49	8
RAP1B_RAT	Q62636	Ras-related protein Rap-1b	Rap1b	20.80	9674.37	9
RAP2B_RAT	P61227	Ras-related protein Rap-2b	Rap2b	20.50	4624.50	6
RB11B_RAT	O35509	Ras-related protein Rab-11b	Rab11b	24.49	4738.40	5
RGS19_RAT	O70521	Regulator of G-protein signaling 19	Rgs19	24.74	2000.75	6
RHOA_RAT	P61589	Transforming protein RhoA	Rhoa	21.78	13836.53	7
RL10A_RAT	P62907	60S ribosomal protein L10a	Rpl10a	24.83	1439.95	6
RL30_RAT	P62890	60S ribosomal protein L30	Rpl30	12.78	3993.24	3
RL40_RAT	P62986	Ubiquitin-60S ribosomal protein L40	Uba52	14.73	35049.19	6
RL5_RAT	P09895	60S ribosomal protein L5	Rpl5	34.46	1356.49	9
RL7_RAT	P05426	60S ribosomal protein L7	Rpl7	30.33	809.21	2.3
RLA1_RAT	P19944	60S acidic ribosomal protein P1	Rplp1	11.50	14697.37	3
RLA2_RAT	P02401	60S acidic ribosomal protein P2	Rplp2	11.69	11381.50	4
ROA3_RAT	Q6URK4	Heterogeneous nuclear ribonucleoprotein A3	Hnrnpa3	39.65	1531.91	7
RPN1_RAT	P07153	Dolichyl-diphosphooligosaccharide protein glycosyltransferase subunit 1	Rpn1	68.30	4482.31	18
RPN2_RAT	P25235	Dolichyl-diphosphooligosaccharide protein glycosyltransferase subunit 2	Rpn2	69.08	2932.00	9
RS19_RAT	P17074	40S ribosomal protein S19	Rps19	16.09	2635.92	5
RS28_RAT	P62859	40S ribosomal protein S28	Rps28	7.84	11656.72	2
RS3_RAT	P62909	40S ribosomal protein S3	Rps3	26.67	3086.85	11
RS7_RAT	P62083	40S ribosomal protein S7	Rps7	22.13	1601.40	4
RS8_RAT	P62243	40S ribosomal protein S8	Rps8	24.21	2727.93	7
RSSA_RAT	P38983	40S ribosomal protein SA	Rpsa	32.82	15306.53	7
RTN4_RAT	Q9JK11	Reticulon-4	Rtn4	126.39	600.97	11

S38A2_RAT	Q9JHE5	Sodium-coupled neutral amino acid transporter 2	Slc38a2	55.55	1988.21	3
SAE1_RAT	Q6AXQ0	SUMO-activating enzyme subunit 1	Sae1	38.51	1620.00	6
SAP_RAT	P10960	Prosaposin	Psap	61.12	2213.07	10
SC22B_RAT	Q4KM74	Vesicle-trafficking protein SEC22b	Sec22b	24.74	3487.08	6
SEPT2_RAT	Q91Y81	Septin-2	Sept2	41.59	701.70	5
SERA_RAT	O08651	D-3-phosphoglycerate dehydrogenase	Phgdh	56.49	1111.31	9
SET_RAT	Q63945	Protein SET	Set	33.41	6928.88	4
SFXN1_RAT	Q63965	Sideroflexin-1	Sfxn1	35.55	1833.89	7
SNAA_RAT	P54921	Alpha-soluble NSF attachment protein (SNAP-alpha)	Napa	33.19	486.89	8
SND1_RAT	Q66X93	Staphylococcal nuclease domain-containing protein 1	Snd1	101.95	5635.33	25
SODC_RAT	P07632	Superoxide dismutase [Cu-Zn]	Sod1	15.91	19388.87	8
SRSF2_RAT	Q6PDU1	Serine/arginine-rich splicing factor 2	Srsf2	25.48	2693.82	5
STX12_RAT	G3V7P1	Syntaxin-12	Stx12	31.19	2343.64	8
SYAC_RAT	P50475	Alanine--tRNA ligase cytoplasmic	Aars	106.79	410.41	13
SYG_RAT	Q5I0G4	Glycine--tRNA ligase	Gars	72.03	1604.88	13
SYSC_RAT	Q6P799	Serine--tRNA ligase cytoplasmic	Sars	58.59	895.43	8
SYYC_RAT	Q4KM49	Tyrosine--tRNA ligase cytoplasmic	Yars	59.12	1173.85	16
TAGL2_RAT	Q5XFX0	Transgelin-2	Tagln2	22.39	23459.84	10
TBA1A_RAT	P68370	Tubulin alpha-1A	Tuba1a	50.14	42173.61	17
TBA1C_RAT	Q6AYZ1	Tubulin alpha-1C	Tuba1c	49.94	28941.29	15
TBA4A_RAT	Q5XIF6	Tubulin alpha-4A	Tuba4a	49.92	12377.59	13
TBB2A_RAT	P85108	Tubulin beta-2A	Tubb2a	49.91	31653.95	16
TBB2B_RAT	Q3KRE8	Tubulin beta-2B	Tubb2b	49.95	31408.71	17
TBB3_RAT	Q4QRB4	Tubulin beta-3	Tubb3	50.42	9224.14	11
TBB4B_RAT	Q6P9T8	Tubulin beta-4B	Tubb4b	49.80	10186.00	16
TBB5_RAT	P69897	Tubulin beta-5	Tubb5	49.67	38708.10	16
TCP4_RAT	Q63396	Activated RNA polymerase II transcriptional coactivator p15	Sub1	14.44	3159.79	4
TERA_RAT	P46462	Transitional endoplasmic reticulum ATPase	Vcp	89.35	1961.14	16
THIO_RAT	P11232	Thioredoxin	Txn	11.67	19963.77	5
THY1_RAT	P01830	Thy-1 membrane glycoprotein	Thy1	18.17	1348.38	5
TKT_RAT	P50137	Transketolase	Tkt	67.64	16757.68	22
TM165_RAT	Q4V899	Transmembrane protein 165	Tmem165	34.81	1066.11	4
TMED7_RAT	D3ZTX0	Transmembrane emp24 domain-containing protein 7	Tmed7	25.48	2409.23	5
TMED9_RAT	Q5I0E7	Transmembrane emp24 domain-containing protein 9	Tmed9	27.03	6239.15	7
TMEDA_RAT	Q63584	Transmembrane emp24 domain-containing protein 10	Tmed10	24.86	3549.54	5
TPIS_RAT	P48500	Triosephosphate isomerase	Tpi1	26.85	19579.29	12
TPM3_RAT	Q63610	Tropomyosin alpha-3	Tpm3	29.01	2366.42	13
TPM4_RAT	P09495	Tropomyosin alpha-4	Tpm4	28.51	8519.55	15

TRPV2_RAT	Q9WUD2	Transient receptor potential cation channel subfamily V member 2 (TRPV2)	Trpv2	86.71	1115.42	14
TRY1_RAT	P00762	Anionic trypsin-1	Prss1	25.96	1052.53	1
TSH3_RAT	D3ZKB9	Teashirt homolog 3	Tshz3	117.46	233.93	7.8
TXNL1_RAT	Q920J4	Thioredoxin-like protein 1	Txnl1	32.25	4646.14	8
TYB4_RAT	P62329	Thymosin beta-4	Tmsb4x	5.05	23782.26	2
UBA1_RAT	Q5U300	Ubiquitin-like modifier-activating enzyme 1	Uba1	117.79	5052.02	25
UBE2N_RAT	Q9EQX9	Ubiquitin-conjugating enzyme E2 N	Ube2n	17.12	7043.94	8
VAMP3_RAT	P63025	VAMP3	Vamp3	11.48	440.77	3
VAT1_RAT	Q3MIE4	Synaptic vesicle membrane protein VAT-1 homolog	Vat1	43.12	4179.74	8
VATB2_RAT	P62815	V-type proton ATPase subunit B	Atp6v1b2	56.55	795.64	15
VDAC1_RAT	Q9Z2L0	Voltage-dependent anion-selective channel protein 1	Vdac1	30.76	2829.24	11
VDAC2_RAT	P81155	Voltage-dependent anion-selective channel protein 2	Vdac2	31.75	695.17	10
VIME_RAT	P31000	Vimentin	Vim	53.73	1477.31	12
VWA5A_RAT	Q75WE7	Von Willebrand factor A domain-containing protein 5A	Vwa5a	91.48	5986.09	18
YBOX3_RAT	Q62764	Y-box-binding protein 3	Ybx3	38.85	421.43	4
YMEL1_RAT	Q925S8	ATP-dependent zinc metalloprotease YME1L1	Yme1l1	79.87	329.19	9

Header line description of worksheets	
**Method**	Protein identification with which Method
**Protein ID**	Protein identification of UniProt
**Protein accession**	Protein accession number of UniProt
**Protein description**	Protein functional description
**Gene name**	Indicates the name of the gene that code for the protein sequence
**MW [kDa]**	Protein molecular weight ,unit [kDa]
**Score AVG**	A simple rule to be used to judge whether a result is significant or not
**Peptides AVG**	Number of peptides whitch spectrum hit
