# Supplementary material for: Proteomic Analysis of Lipid Rafts from RBL-2H3 Mast Cells
Source: Int J Mol Sci. 2019 Aug 11;20(16):3904. doi: 10.3390/ijms20163904 (PMC6720779; doi:10.3390/ijms20163904)
Supplement: Supplementary file 1 [file ijms-20-03904-s001.zip › Table S2.pdf]

Supplementary Table 2. Detailed annotation of proteins identified in Method II

Protein ID	Protein accession	Protein description	Gene name	MW [kDa]	Score AVG	Peptides AVG
1433B_RAT	P35213	14-3-3 protein beta/alpha	Ywhab	28.05	1578.545	8
1433E_RAT	P62260	14-3-3 protein epsilon	Ywhae	29.17	4654.923	10
1433F_RAT	P68511	14-3-3 protein eta	Ywhah	28.21	2128.056	9
1433G_RAT	P61983	14-3-3 protein gamma	Ywhag	28.30	4878.047	11
1433T_RAT	P68255	14-3-3 protein theta	Ywhaq	27.78	1600.605	8
1433Z_RAT	P63102	14-3-3 protein zeta/delta	Ywhaz	27.77	17064.52	11
4F2_RAT	Q794F9	4F2 cell-surface antigen heavy chain	Slc3a2	58.07	25879.06	20
A0JN29_RAT	A0JN29	Lunapark ER junction formation factor	Lnpk	42.17	5453.1016	12
A0JN30_RAT	A0JN30	Canopy 2 homolog	Cnpy2	20.71	1589.0969	4
A1L1J8_RAT	A1L1J8	Ras-related protein Rab-5b	Rab5b	23.68	11794.084	8
A1L1L6_RAT	A1L1L6	Mitochondrial Rho GTPase 1	Rhot1	72.16	571.15546	10
A3RLA8_RAT	A3RLA8	Low affinity immunoglobulin gamma Fc region receptor II	Fcgr2b	37.97	6999.2134	9
AATM_RAT	P00507	Aspartate aminotransferase mitochondrial	Got2	47.31	1015.1728	11
ABCB6_RAT	O70595	ATP-binding cassette sub-family B member 6	Abcb6	93.31	5574.2964	13
ABCB8_RAT	Q5RKI8	ATP-binding cassette sub-family B member 8	Abcb8	77.77	1087.13	13
ABHGA_RAT	Q6MG55	Abhydrolase domain containing 16A	Abhd16a	63.04	529.3098	12
ACSL3_RAT	Q63151	Long chain fatty acid CoA ligase 3	Acsl3	80.46	318.20175	13
ACSL5_RAT	O88813	Long chain fatty acid CoA ligase 5	Acsl5	76.41	1395.4258	17
ACTB_RAT	P60711	Actin cytoplasmic 1	Actb	41.74	34703.336	21
ADA15_RAT	Q9QYV0	Disintegrin and metalloproteinase domain-containing protein 15	Adam15	93.31	474.3245	9
ADT1_RAT	Q05962	ADP/ATP translocase 1	Slc25a4	32.99	14974.186	13
ADT2_RAT	Q09073	ADP/ATP translocase 2	Slc25a5	32.90	25205.96	22
AIFM1_RAT	Q9JM53	Apoptosis-inducing factor 1	Aifm1	66.72	561.40063	11
AKAP1_RAT	O88884	A-kinase anchor protein 1, mitochondrial	Akap1	91.75	2523.194	13
AL5AP_RAT	P20291	Arachidonate 5-lipoxygenase-activating protein	Alox5ap	18.07	15126.04	5
ALDOA_RAT	P05065	Fructose-bisphosphate aldolase A	Aldoa	39.35	889.46655	7
ANXA1_RAT	P07150	Annexin A1	Anxa1	38.83	850.72046	10
APAF_RAT	Q9EPV5	Apoptotic protease-activating factor 1	Apaf1	141.15	424.95	9
ARL8B_RAT	Q66HA6	ADP-ribosylation factor-like protein 8B	Arl8b	21.54	4317.4966	10
ARM10_RAT	B1WBW4	Armadillo repeat-containing protein 10	Armc10	33.42	6899.171	9
ARMX3_RAT	Q5XID7	Armadillo repeat-containing X-linked protein 3	Armcx3	42.55	1883.8921	9
AT1A1_RAT	P06685	Sodium/potassium-transporting ATPase subunit alpha-1	Atp1a1	113.05	18895.371	42
AT1A4_RAT	Q64541	Sodium/potassium-transporting ATPase subunit alpha-4	Atp1a4	114.01	3007.623	12

AT1B3_RAT	Q63377	Sodium/potassium-transporting ATPase subunit beta-3	Atp1b3	31.83	13653.33	8
AT2A1_RAT	Q64578	Sarcoplasmic/endoplasmic reticulum calcium ATPase 1 (SERCA1)	Atp2a1	109.41	7037.0156	18
AT5F1_RAT	P19511	ATP synthase F(0) complex subunit B1 mitochondrial	Atp5pb	28.87	6731.9316	11
ATAD1_RAT	Q505J9	ATPase family AAA domain-containing protein 1	Atad1	40.72	3386.4302	10
ATAD3_RAT	Q3KRE0	ATPase family AAA domain-containing protein 3	Atad3	66.76	3541.2605	21
ATP4A_RAT	P09626	Potassium-transporting ATPase alpha chain 1	Atp4a	114.04	1110.19	12
ATP5H_RAT	P31399	ATP synthase subunit D mitochondrial	Atp5pd	18.76	12645.211	7
ATP5I_RAT	P29419	ATP synthase subunit E mitochondrial	Atp5me	8.26	26547.965	5
ATP5J_RAT	P21571	ATP synthase-coupling factor 6 mitochondrial	Atp5pf	12.49	18856.01	5
ATPA_RAT	P15999	ATP synthase subunit alpha mitochondrial	Atp5f1a	59.75	48943.023	29
ATPG_RAT	P35435	ATP synthase subunit gamma mitochondrial	Atp5f1c	30.19	9105.262	11
ATPK_RAT	D3ZAF6	ATP synthase subunit F mitochondrial	Atp5mf	10.45	4065.0117	2
ATPO_RAT	Q06647	ATP synthase subunit O mitochondrial	Atp5po	23.40	20299.375	12
ATRAP_RAT	Q642A2	Type-1 angiotensin II receptor-associated protein	Agtrap	17.40	5631.5186	2
AUP1_RAT	A1L134	Ancient ubiquitous protein 1	Aup1	46.20	1158.9775	8
B0BN02_RAT	B0BN02	Metaxin 1	Mtx1	48.44	1487.6011	7
B0BN30_RAT	B0BN30	Mitochondrial carrier homolog 1	Mtch1	41.59	987.7393	6
B0BN52_RAT	B0BN52	Mitochondrial carrier homolog 2	Mtch2	33.53	5936.334	8
B0BN81_RAT	B0BN81	Ribosomal protein S5	Rps5	22.91	5174.41	5
B0BNG3_RAT	B0BNG3	Vesicular integral-membrane protein VIP36	Lman2	40.39	24611.645	18
B0BNK1_RAT	B0BNK1	Ras-related protein Rab-5c	Rab5c	23.43	19618.02	9
B1WBY5_RAT	B1WBY5	DnaJ homolog subfamily C member 11	Dnajc11	63.21	1092.5051	14
B1WBY7_RAT	B1WBY7	Erlin-1	Erlin1	39.27	4103.746	9
B1WC50_RAT	B1WC50	RNA-binding protein EWS-like	Ewsr1	68.33	1089.7468	9
B1WC61_RAT	B1WC61	Acyl-CoA dehydrogenase family member 9	Acad9	68.84	698.94855	13
B1WC67_RAT	B1WC67	Solute carrier family 25 member 24	Slc25a24	52.89	859.5758	10
B2GUV5_RAT	B2GUV5	V-type proton ATPase subunit G 1	Atp6v1g1	13.71	1920.5234	4
B2GUX5_RAT	B2GUX5	5'-nucleotidase	Nt5c3a	33.81	4055.9336	12
B2GUZ9_RAT	B2GUZ9	Fam49b protein	Fam49b	36.78	1878.8369	7
B2GV96_RAT	B2GV96	Coiled-coil domain containing 115	Ccdc115	19.82	2758.0981	7
B2MG_RAT	P07151	Beta-2-microglobulin	B2m	13.72	3851.3464	3
B2RYC9_RAT	B2RYC9	Glucosylceramidase	Gba	57.49	2284.374	13
B2RYD7_RAT	B2RYD7	STT3B, catalytic subunit of the oligosaccharyltransferase complex	Stt3b	93.44	1266.1827	12
B2RYF6_RAT	B2RYF6	Cleft lip and palate associated transmembrane protein 1	Clptm1	75.28	3689.6953	13
B2RYQ2_RAT	B2RYQ2	Serine/threonine-protein phosphatase 2A activator	Ptpa	36.62	738.1688	6
B2RZB3_RAT	B2RZB3	Eva-1 homolog B	Eva1b	24.58	966.3343	4
B2RZD1_RAT	B2RZD1	Protein transport protein Sec61 subunit beta	Sec61b	9.99	28681.2	3
B3A2_RAT	P23347	Anion exchange protein 2	Slc4a2	136.64	721.1465	20

B4F7C2_RAT	B4F7C2	Tubulin beta-4A	Tubb4a	49.59	2889.181	16
B4F7C9_RAT	B4F7C9	STT3A, catalytic subunit of the oligosaccharyltransferase complex	Stt3a	80.60	5105.618	19
B4GN1_RAT	Q10468	Beta-1,4 N-acetylgalactosaminyltransferase 1	B4galnt1	59.28	1427.76	12
B5DEL8_RAT	B5DEL8	NADH dehydrogenase (Ubiquinone) iron-sulfur protein 5	Ndufs5	12.70	652.6625	2
B5DEZ3_RAT	B5DEZ3	Coiled-coil domain containing 126	Ccdc126	15.53	4296.792	3
B5DFJ2_RAT	B5DFJ2	Protein FAM3A	Fam3a	25.15	1369.282	7
B6RK61_RAT	B6RK61	Myosin heavy chain 7B	Myh7b	221.63	18.7633	1
BAF_RAT	Q9R1T1	Barrier-to-autointegration factor	Banf1	10.05	2330.153	4
BASI_RAT	P26453	Basigin	Bsg	42.44	11856.585	8
BASP1_RAT	Q05175	Brain acid soluble protein 1	Basp1	21.79	41388.22	9
BET1_RAT	Q62896	Bet1 golgi vesicular membrane trafficking protein	Bet1	13.23	8325.793	2
BET1L_RAT	O35152	BET1-like protein	Bet1l	12.42	1466.276	2
C1GLC_RAT	Q499P3	C1GALT1-specific chaperone 1	C1galt1c1	36.04	1047.14	5
C1GLT_RAT	Q9JJ05	Glycoprotein-N-acetylgalactosamine 3-beta-galactosyltransferase 1	C1galt1	42.31	2600.0864	8
C1QBP_RAT	O35796	Complement component 1 Q subcomponent-binding protein mitochondrial	C1qbp	31.00	17896.625	7
CADM3_RAT	Q1WIM3	Cell adhesion molecule 3	Cadm3	42.91	6705.5596	11
CAH8_RAT	Q5PPN4	Carbonic anhydrase-related protein	Ca8	32.96	631.64404	5
CALR_RAT	P18418	Calreticulin	Calr	48.00	8945.08	11
CALX_RAT	P35565	Calnexin	Canx	67.26	19625.45	23
CBPA3_RAT	P21961	Mast cell carboxypeptidase A	Cpa3	47.94	1407.913	10
CCD47_RAT	Q5U2X6	Coiled-coil domain-containing protein 47	Ccdc47	55.73	10018.979	18
CD38_RAT	Q64244	ADP-ribosyl cyclase/cyclic ADP-ribose hydrolase 1	Cd38	34.44	4465.1885	9
CD48_RAT	P10252	CD48 antigen	Cd48	27.68	44269.734	14
CDC42_RAT	Q8CFN2	Cell division control protein 42 homolog (CDC42)	Cdc42	21.26	26481.715	8
CH10_RAT	P26772	10 kDa heat shock protein mitochondrial	Hspe1	10.90	11615.26	5
CH60_RAT	P63039	60 kDa heat shock protein	Hspd1	60.96	8192.467	24
CHPT1_RAT	Q66H21	Cholinephosphotransferase 1	Chpt1	44.60	1437.133	3
CISD1_RAT	B0K020	CDGSH iron-sulfur domain-containing protein 1	Cisd1	12.10	39811.453	7
CISY_RAT	Q8VHF5	Citrate synthase mitochondrial	Cs	51.87	1031.1398	6
CK5P3_RAT	Q9JLH7	CDK5 regulatory subunit-associated protein 3	Cdk5rap3	57.05	4572.61	16
CL2DB_RAT	Q0H8B9	C-type lectin domain family 2 member D11	Clec2d11	23.54	3521.782	7
CLCN5_RAT	P51796	Chloride intracellular channel protein 5	Clcn5	83.07	1283.8569	8
CLH1_RAT	P11442	Clathrin heavy chain 1	Cltc	191.60	613.12506	26
CMA1_RAT	P50339	Chymase 1	Cma1	27.57	1508.505	3
CNTN1_RAT	Q63198	Contactin 1	Cntn1	113.50	1301.9305	20
COF1_RAT	P45592	Cofilin-1	Cfl1	18.53	2967.661	6
COMT_RAT	P22734	Catechol O-methyltransferase	Comt	29.60	1367.642	7
COPB_RAT	P23514	Coatomer subunit beta 1	Copb1	107.01	1067.6825	20

COPB2_RAT	O35142	Coatomer subunit beta 2	Copb2	102.55	1245.673	16
COPD_RAT	Q66H80	Coatomer subunit delta	Arcn1	57.20	564.4874	9
COPG1_RAT	Q4AEF8	Coatomer subunit gamma 1	Copg1	97.61	405.6862	16
COX41_RAT	P10888	Cytochrome c oxidase subunit 4 isoform 1 mitochondrial	Cox4i1	19.52	12578.01	11
COX5A_RAT	P11240	Cytochrome c oxidase subunit 5A mitochondrial	Cox5a	16.13	17269.785	9
COX5B_RAT	P12075	Cytochrome c oxidase subunit 5B mitochondrial	Cox5b	13.92	6169.5947	6
CP20A_RAT	Q6P7D4	Cytochrome P450 20A1	Cyp20a1	52.00	704.8057	7
CP51A_RAT	Q64654	Lanosterol 14-alpha demethylase	Cyp51a1	56.71	2204.413	14
CR1L_RAT	Q63135	Complement component receptor 1-like protein	Cr1l	61.68	7281.857	19
CX6C2_RAT	P11951	Cytochrome c oxidase subunit 6C2	Cox6c2	8.46	6444.0205	4
CX7A2_RAT	P35171	Cytochrome c oxidase subunit 7A2	Cox7a2	9.35	2968.9531	3
CYB5B_RAT	P04166	Cytochrome b5 type B	Cyb5b	16.27	8170.613	5
D3Z9L0_RAT	D3Z9L0	Acylglycerol kinase	Agk	54.26	433.50516	8
D3ZAS9_RAT	D3ZAS9	DDRGK domain containing 1	Ddrgk1	38.61	10160.611	6
D3ZAY7_RAT	D3ZAY7	Band 4.1-like protein 2	Epb41l2	89.13	739.45105	17
D3ZCZ9_RAT	D3ZCZ9	NADH dehydrogenase [ubiquinone] iron-sulfur protein 6	LOC100912599	13.04	885.649	3
D3ZD09_RAT	D3ZD09	Cytochrome c oxidase subunit 6B1	Cox6b1	10.07	13217.445	7
D3ZD11_RAT	D3ZD11	Signal peptidase complex subunit 2	Spcs2	24.97	11044.09	13
D3ZD83_RAT	D3ZD83	Major facilitator superfamily domain-containing protein 10	Mfsd10	49.41	791.0322	5
D3ZDR2_RAT	D3ZDR2	Charged multivesicular body protein 6	Chmp6	23.29	1383.381	5
D3ZF11_RAT	D3ZF11	Ragulator complex protein LAMTOR5	Lamtor5	16.39	8052.9424	4
D3ZF12_RAT	D3ZF12	Signal peptidase complex subunit 3	Spcs3	20.33	6419.79	7
D3ZF54_RAT	D3ZF54	Anoctamin-10	Ano10	79.04	598.88275	10
D3ZF97_RAT	D3ZF97	Endoplasmic reticulum lectin 1	Erlec1	54.67	833.27783	10
D3ZFK5_RAT	D3ZFK5	Signal peptidase complex subunit 1	Spcs1	18.10	34303.133	4
D3ZG43_RAT	D3ZG43	NADH dehydrogenase (ubiquinone) iron-sulfur protein 3	Ndufs3	30.23	3831.0947	7
D3ZGM7_RAT	D3ZGM7	Peptidase inhibitor 16	Pi16	52.15	644.4734	2
D3ZGV2_RAT	D3ZGV2	Peroxisomal membrane protein PEX16	Pex16	35.30	343.32764	4
D3ZHA7_RAT	D3ZHA7	Myosin light chain 6B	RGD1560334_predicted	22.81	6198.9604	5
D3ZIE9_RAT	D3ZIE9	Delta-1-pyrroline-5-carboxylate synthase	Aldh18a1	87.33	369.4042	16
D3ZIJ5_RAT	D3ZIJ5	Dolichyl-phosphate mannosyltransferase subunit 3	Dpm3	10.09	3345.474	1
D3ZIP8_RAT	D3ZIP8	Endonuclease domain containing 1	---	38.11	6498.826	9
D3ZJ32_RAT	D3ZJ32	Extended synaptotagmin 2	Esyt2	93.95	1500.0415	16
D3ZJS3_RAT	D3ZJS3	Translocase of outer mitochondrial membrane 6	Tomm6	7.89	9962.432	2
D3ZJX5_RAT	D3ZJX5	Mitochondrial import inner membrane translocase subunit TIM50	Timm50	39.86	1962.5115	8
D3ZK56_RAT	D3ZK56	Ras-related protein Rap-2c	Rap2c	20.75	31541.605	9
D3ZKE6_RAT	D3ZKE6	Sarcolemmal membrane-associated protein	Slmap	90.56	1028.4661	30
D3ZL85_RAT	D3ZL85	Cytochrome c heme lyase	Hccs	31.13	1843.668	6

D3ZLC4_RAT	D3ZLC4	Slc2a6 protein	Slc2a6	35.90	1620.044	5
D3ZLT7_RAT	D3ZLT7	Uncharacterized protein KIAA2013	RGD1305350	69.48	1386.548	6
D3ZM19_RAT	D3ZM19	Protein EVI2B	Evi2b	45.62	893.4126	4
D3ZMM8_RAT	D3ZMM8	Solute carrier family 7 member 6	Slc7a6	56.82	422.52615	4
D3ZMR1_RAT	D3ZMR1	Translocase of outer mitochondrial membrane 7	Tomm7	6.18	2952.162	1
D3ZN21_RAT	D3ZN21	ATP-dependent RNA helicase DDX3Y	Ddx3y	72.99	1069.654	9
D3ZNG3_RAT	D3ZNG3	Signal peptide peptidase-like 2A	Sppl2a	59.84	411.4917	5
D3ZNS8_RAT	D3ZNS8	Aladin	Aaas	59.36	729.3629	7
D3ZP13_RAT	D3ZP13	Sulfhydryl oxidase 2	Qsox2	79.20	237.4855	5
D3ZP98_RAT	D3ZP98	Minor histocompatibility antigen H13	Hm13	40.28	1971.5709	9
D3ZPR0_RAT	D3ZPR0	Exportin-2	Cse1l	110.21	443.69458	13
D3ZQG9_RAT	D3ZQG9	RT1 class I locus CE4	RT1-CE4	40.08	6122.714	9
D3ZRN3_RAT	D3ZRN3	Beta-actin-like protein 2	Actbl2	41.96	9906.157	10
D3ZSA9_RAT	D3ZSA9	Nodal modulator 1	Nomo1	133.44	9235.345	36
D3ZSY5_RAT	D3ZSY5	keratin 84	Krt84	61.20	202.2945	3
D3ZTJ0_RAT	D3ZTJ0	Protein XRP2	Rp2	39.32	1668.8065	9
D3ZU83_RAT	D3ZU83	Endoplasmic reticulum-Golgi intermediate compartment 3	Ergic3	43.22	858.6881	6
D3ZUD8_RAT	D3ZUD8	Transmembrane 9 superfamily member 3	Tm9sf3	69.97	2607.2104	13
D3ZUQ7_RAT	D3ZUQ7	Pantothenate kinase 3	Pank3	41.09	151.1794	1
D3ZUX5_RAT	D3ZUX5	MICOS complex subunit MIC-19	Chchd3	26.44	7058.3394	11
D3ZUZ4_RAT	D3ZUZ4	Glycosaminoglycan xylosylkinase	Fam20b	46.57	2274.256	10
D3ZV75_RAT	D3ZV75	Major facilitator superfamily domain-containing protein 1	Mfsd1	51.20	1743.58	3
D3ZWS0_RAT	D3ZWS0	Protein scribble homolog	Scrib	179.85	1428.7123	20
D3ZWZ1_RAT	D3ZWZ1	Integrin alpha X	Itgax	130.18	9389.506	41
D3ZXF9_RAT	D3ZXF9	39S ribosomal protein L12, mitochondrial	Mrpl12	29.44	1390.9845	8
D3ZYM4_RAT	D3ZYM4	Macrophage stimulating 1 receptor	Mst1r	150.24	69.8444	1
D3ZYW2_RAT	Q499R8	Heterogeneous nuclear ribonucleoprotein H	Hnrnph1	20.58	671.74725	7
D3ZZR9_RAT	D3ZZR9	Peptidylprolyl isomerase	Fkbp2	15.39	281.2132	11
D3ZZS8_RAT	D3ZZS8	V-type proton ATPase subunit B, kidney isoform	Atp6v1b1	67.25	912.30475	9
D4A017_RAT	D4A017	Transmembrane protein 87 A	Tmem87a	61.67	1767.698	11
D4A0E2_RAT	D4A0E2	NSF attachment protein gamma	Napg	25.60	613.8713	6
D4A0G7_RAT	D4A0G7	Ras-related protein Rab-37	Rab37	24.66	6001.734	8
D4A0T0_RAT	D4A0T0	NADH dehydrogenase 1 beta subcomplex subunit 10	Ndufb10	20.86	4477.3477	8
D4A0T8_RAT	D4A0T8	Dehydrogenase/reductase SDR family member 7	Dhrs7	38.49	3295.6035	6
D4A0W1_RAT	D4A0W1	ER membrane protein complex subunit 4	Emc4	20.16	2497.1655	5
D4A133_RAT	D4A133	V-type proton ATPase catalytic subunit A	Atp6v1a	68.27	5861.986	31
D4A1D8_RAT	D4A1D8	Surfeit 4	Surf4	30.38	16413.965	7
D4A2H2_RAT	D4A2H2	Serine palmitoyltransferase 1	Sptlc1	52.55	629.18225	7

D4A2Z6_RAT	D4A2Z6	Translocation protein SEC63 homolog	Sec63	83.59	2278.4956	18
D4A376_RAT	D4A376	Ras-related protein Rab-12	Rab12	27.38	3937.3467	5
D4A416_RAT	D4A416	Cleft lip and palate transmembrane protein 1-like protein	Clptm1l	62.29	1412.2905	10
D4A465_RAT	D4A465	Ragulator complex protein LAMTOR2	Lamtor2	13.48	35403.906	6
D4A4J3_RAT	D4A4J3	Alpha-mannosidase 2x	Man2a2	131.96	171.00595	5
D4A4T0_RAT	D4A4T0	STIP1 homology and U box-containing protein 1	Stub1	34.89	338.5735	7
D4A533_RAT	D4A533	Transmembrane anterior posterior transformation protein 1	Tapt1	69.56	319.5562	10
D4A5K6_RAT	D4A5K6	Zinc metallopeptidase STE24	Zmpste24	54.85	8281.583	9
D4A781_RAT	D4A781	Importin 5	Ipo5	123.71	438.79364	13
D4A7Y9_RAT	D4A7Y9	Essential MCU regulator, mitochondrial	Smdt1	11.59	1977.516	4
D4A994_RAT	D4A994	ER membrane protein complex subunit 1	Emc1	111.19	4429.3115	21
D4A9L2_RAT	D4A9L2	Serine/arginine-rich splicing factor 1	Srsf1	27.75	1289.941	5
D4A9X7_RAT	D4A9X7	Hypermethylated in cancer 2 protein	Hic2	67.20	609.6509	4
D4AA91_RAT	D4AA91	Ceramide synthase 5	Cers5	51.32	805.07056	7
D4AAE9_RAT	D4AAE9	CDGSH iron-sulfur domain-containing protein 2	Cisd2	15.29	24451.86	7
D4AAL4_RAT	D4AAL4	Adhesion G protein-coupled receptor L3	Adgrl3	172.45	460.62485	24
D4ABI7_RAT	D4ABI7	Very-long-chain (3R)-3-hydroxyacyl-CoA dehydratase	Hacd3	43.10	3264.7783	7
D4ABM3_RAT	D4ABM3	Dishevelled associated activator of morphogenesis 1	Daam1	112.64	2665.12	29
D4ABY2_RAT	D4ABY2	Coatomer subunit gamma 2	Copg2	80.49	678.3458	11
D4ACG2_RAT	D4ACG2	Acetolactate synthase-like protein	Ilvbl	72.27	731.1177	12
D4ADE8_RAT	D4ADE8	ATP-dependent RNA helicase DDX3X	Ddx3x	34.34	672.98505	5
D4ADS9_RAT	D4ADS9	EFR3 homolog A	Efr3a	92.52	3957.558	27
D4ADV4_RAT	Q80ZA5	Sodium-driven chloride bicarbonate exchanger	Slc4a10	125.64	939.1171	6
D4AEG2_RAT	D4AEG2	Ras-related protein Rab-32	Rab32	25.26	8105.4756	8
DAD1_RAT	P61805	Dolichyl-diphosphooligosaccharide protein glycosyltransferase subunit DAD1	Dad1	12.50	20682.31	3
DHC24_RAT	Q5BQE6	Delta(24)-sterol reductase	Dhcr24	60.05	924.7401	8
DHCR7_RAT	Q9Z2Z8	7-dehydrocholesterol reductase	Dhcr7	54.16	1749.263	9
DHE3_RAT	P10860	Glutamate dehydrogenase 1	Glud1	61.42	441.7484	11
DHPR_RAT	P11348	Dihydropteridine reductase	Qdpr	25.55	408.2444	4
DHSA_RAT	Q920L2	Succinate dehydrogenase [ubiquinone] flavoprotein subunit mitochondrial	Sdha	71.62	5168.7197	23
DHSB_RAT	P21913	Succinate dehydrogenase [ubiquinone] iron-sulfur subunit mitochondrial	Sdhb	31.83	2446.2795	9
E2AK3_RAT	Q9Z1Z1	Eukaryotic translation initiation factor 2-alpha kinase 3	Eif2ak3	124.77	602.3982	19
E9PT10_RAT	Q861Q1	MHC class I antigen	RT1-CE4	42.08	5165.4585	6
E9PT29_RAT	E9PT29	ATP-dependent RNA helicase DDX17	Ddx17	72.46	985.8777	9
E9PTI1_RAT	E9PTI1	V-type proton ATPase subunit H	Atp6v1h	50.83	2445.845	6
E9PTU4_RAT	E9PTU4	Myosin heavy chain 11	Myh11	227.30	458.32947	28
E9PTW1_RAT	E9PTW1	Secretory carrier-associated membrane protein	Scamp3	38.50	15290.525	8
E9PTX9_RAT	E9PTX9	Solute carrier family 12 member 2	Slc12a2	130.37	1066.7125	16

E9PU37_RAT	E9PU37	Palmitoyltransferase	Zdhhc13	70.77	750.1116	12
E9PU77_RAT	E9PU77	Synaptotagmin-like protein 3	Sytl3	68.69	2004.4719	15
ECHA_RAT	Q64428	Trifunctional enzyme subunit alpha	Hadha	82.67	3063.2075	23
EF1G_RAT	Q68FR6	Elongation factor 1-gamma	Eef1g	50.06	1391.5375	11
EF2_RAT	P05197	Elongation factor 2	Eef2	95.28	1866.2566	22
EMC2_RAT	B0BNG0	ER membrane protein complex subunit 2	Emc2	34.87	10491.857	10
EMC3_RAT	Q5U2V8	ER membrane protein complex subunit 3	Emc3	29.98	2137.417	8
EMC8_RAT	Q5FVL2	ER membrane protein complex subunit 8	Emc8	23.41	10935.938	8
ENOA_RAT	P04764	Alpha-enolase	Eno1	47.13	2993.3784	16
ENPL_RAT	Q66HD0	Endoplasmin	Hsp90b1	92.77	3524.756	23
ENPP3_RAT	P97675	Ectonucleotide pyrophosphatase/phosphodiesterase family member 3	Enpp3	99.07	3102.8271	22
ERLN2_RAT	B5DEH2	Erlin-2	Erlin2	37.71	6296.634	13
ESYT1_RAT	Q9Z1X1	Extended synaptotagmin 1	Esyt1	121.16	7809.712	43
F1LM66_RAT	F1LM66	116 kDa U5 small nuclear ribonucleoprotein component	Eftud2	109.35	61.3982	1
F1LMG7_RAT	F1LMG7	Leucine-rich repeat-containing protein 7	Lrrc7	56.68	101.5547	3
F1LMK0_RAT	F1LMK0	A-kinase anchoring protein 1 isoform 2	Akap1	57.65	3006.1821	14
F1LMM5_RAT	F1LMM5	Arachidonate 5-lipoxygenase	Alox5	78.13	6856.393	23
F1LN88_RAT	F1LN88	Aldehyde dehydrogenase	Aldh2	56.52	406.324	8
F1LNC4_RAT	F1LNC4	Vesicle transport through interaction with t-SNAREs homolog 1B	Vti1b	23.10	7061.8354	10
F1LNF0_RAT	F1LNF0	Myosin heavy chain 14	Myh14	228.91	502.265	28
F1LNT8_RAT	F1LNT8	VAMP8	Vamp8	11.19	10217.439	5
F1LNX2_RAT	F1LNX2	Calcium release-activated calcium channel protein 1 (Orai1)	Orai1	32.44	1446.3807	2
F1LP82_RAT	F1LP82	Ras-related protein Rab-2a	Rab2a	22.81	28618.023	13
F1LPK9_RAT	F1LPK9	Phosphatidylinositol glycan anchor biosynthesis class U protein	Pigu	49.80	2507.71	3
F1LQ81_RAT	F1LQ81	Vesicle-fusing ATPase	Nsf	82.63	10610.1	29
F1LQ92_RAT	F1LQ92	Sel-1 homolog 1	Sel1l	88.44	436.3137	13
F1LQR8_RAT	F1LQR8	Inositol 1,4,5-trisphosphate receptor type 2	Itpr2	234.15	1721.356	50
F1LRK1_RAT	F1LRK1	Sodium/potassium-transporting ATPase subunit alpha	Atp4a	114.04	929.3479	10
F1LRV9_RAT	F1LRV9	Myosin heavy chain 1	Myh1	223.40	18.7633	1
F1LSI5_RAT	F1LSI5	Ras GTPase-activating protein 3	Rasa3	18.93	554.1449	18
F1LSV4_RAT	Q3B7D2	Serine palmitoyltransferase 2	Sptlc2	62.96	340.8114	9
F1LT70_RAT	F1LT70	Pantothenate kinase 2	Pank2	48.70	151.1794	1
F1LU48_RAT	F1LU48	Endoplasmic reticulum-golgi intermediate compartment 1	Ergic1	35.01	2118.4985	8
F1LVC3_RAT	F1LVC3	Ras-related protein Rab-6b	Rab6b	19.88	14459.689	10
F1LW77_RAT	F1LW77	Ras-related protein Rab-33b	Rab33b	25.75	6495.215	3
F1LX07_RAT	F1LX07	Solute carrier family 25 member 12	Slc25a12	71.92	2848.0625	19
F1LZW6_RAT	F1LZW6	Solute carrier family 25 member 13	Slc25a13	54.10	4341.621	24
F1LZX9_RAT	F1LZX9	Integrin alpha 5	Itgav	115.58	4434.619	32

F1M0G0_RAT	F1M0G0	Regulator of G-protein signaling 17	Rgs17	24.32	18100.955	8
F1M0J3_RAT	F1M0J3	Sodium bicarbonate cotransporter 3, isophorm 4	Slc4a7	126.85	2402.228	25
F1M1U0_RAT	F1M1U0	Matrix remodeling associated 7	Mxra7	15.89	510.5609	1
F1M391_RAT	F1M391	Stimulator of interferon genes protein	Tmem173	42.66	3505.448	6
F1M5Z5_RAT	F1M5Z5	Anoctamin-6	Ano6	92.23	340.6557	10
F1M6W2_RAT	F1M6W2	Endoplasmic reticulum metallopeptidase 1	Ermp1	99.98	1936.6069	13
F1M779_RAT	F1M779	Clathrin heavy chain	Cltc	191.56	394.4044	24
F1M790_RAT	F1M790	Prostaglandin F2 receptor negative regulator	Ptgfrn	98.67	1393.22	20
F1M7T6_RAT	F1M7T6	Translocon-associated protein subunit gamma	Ssr3	7.38	20326.365	3
F1M8F6_RAT	F1M8F6	Myosin heavy chain 8	Myh8	166.64	18.7633	1
F1MA50_RAT	F1MA50	transmembrane protein 123	Tmem123	15.22	1612.2595	2
F1MA89_RAT	F1MA89	Cyclin Y	Ccny	39.37	839.9437	7
F1MAS0_RAT	F1MAS0	keratin 32	Krt32	51.53	1196.745	7
F6T9E7_RAT	F6T9E7	P-selectin glycoprotein ligand 1	Selplg	44.44	739.8876	2
F7EP67_RAT	Q6IN10	Endothelin-converting enzyme 1 Isoform	Ece1	85.59	459.2204	15
F7F4S8_RAT	B2RYB8	Integrin beta 2	Itgb2	84.82	20343.656	36
F7F744_RAT	Q6S395	Plectin	Plec	517.53	3118.4915	158
F7FFV2_RAT	F7FFV2	keratin 5	Krt5	44.02	4067.2588	12
F7FKI5_RAT	F7FKI5	Pyruvate dehydrogenase component E1 alpha	Pdha1	43.11	1030.9702	12
F7FNS3_RAT	F7FNS3	Derlin	Derl1	28.85	1641.825	4
FACR1_RAT	Q66H50	Fatty acyl-CoA reductase 1	Far1	59.27	2376.1807	14
FADS2_RAT	Q9Z122	Acyl-CoA 6-desaturase	Fads2	52.38	4900.399	10
FAF2_RAT	Q5BK32	FAS-associated factor 2	Faf2	41.08	2111.1401	11
FAM3C_RAT	Q810F4	Protein FAM3C	Fam3c	24.71	15365.18	10
FCERB_RAT	P13386	High affinity immunoglobulin epsilon receptor subunit beta	Ms4a2	26.73	17897.814	9
FCERG_RAT	P20411	High affinity immunoglobulin epsilon receptor subunit gamma	Fcer1g	9.77	12236.995	4
FGOP2_RAT	Q6TA25	FGFR1 oncogene partner 2 homolog	Fgfr1op2	29.37	643.8412	9
FGR_RAT	Q6P6U0	Tyrosine-protein kinase Fgr	Fgr	58.82	508.04712	3
FIS1_RAT	P84817	Mitochondrial fission 1 protein	Fis1	17.00	3646.877	4
FKBP8_RAT	Q3B7U9	Peptidyl-prolyl cis-trans isomerase FKBP8	Fkbp8	43.56	11431.855	10
FLOT1_RAT	Q9Z1E1	Flotillin-1	Flot1	47.50	2314.9595	18
FLOT2_RAT	Q9Z2S9	Flotillin-2	Flot2	47.04	3362.4966	17
FRK_RAT	Q62662	Tyrosine-protein kinase FRK	Frk	58.17	76.13575	1
FUT8_RAT	Q6EV76	Alpha-(1,6)-fucosyltransferase	Fut8	66.49	1289.239	12
G3P_RAT	P04797	Glyceraldehyde-3-phosphate dehydrogenase	Gapdh	35.83	17496.055	14
G3PT_RAT	Q9ESV6	Glyceraldehyde-3-phosphate dehydrogenase testis-specific	Gapdhs	46.71	2802.8984	6
G3V608_RAT	G3V608	Interleukin-3 receptor subunit beta	Csf2rb	99.14	616.4434	14
G3V637_RAT	G3V637	Syntaxin-binding protein 2, isoform	Stxbp2	66.65	3765.574	24

G3V667_RAT	G3V667	Integrin alpha 6	Itga6	119.48	743.091	17
G3V679_RAT	G3V679	Transferrin receptor protein 1	Tfrc	85.88	3309.5024	26
G3V6D3_RAT	G3V6D3	ATP synthase subunit beta	Atp5f1b	56.35	51747.17	23
G3V6E1_RAT	G3V6E1	Myosin heavy chain 2	Myh2	219.58	18.7633	1
G3V6H0_RAT	G3V6H0	Ras-related protein Rab-1b	LOC100363782	22.18	55245.715	14
G3V6H5_RAT	G3V6H5	Solute carrier family 25 member 11	Slc25a11	34.14	3730.7212	12
G3V6N2_RAT	G3V6N2	Transmembrane emp24 domain-containing protein 4	Tmed4	26.12	10377.479	9
G3V6P7_RAT	G3V6P7	Myosin heavy chain 9	Myh9	226.41	7561.104	86
G3V6P8_RAT	G3V6P8	Guanine nucleotide-binding protein gamma-12	Gng12	7.99	77053.73	5
G3V6R4_RAT	G3V6R4	Leucine-rich repeat-containing protein 1	Lrrc1	59.39	2278.051	15
G3V6T1_RAT	G3V6T1	Coatomer subunit alpha	Copa	138.36	777.2628	27
G3V6W2_RAT	G3V6W2	Prolactin regulatory element-binding protein	Preb	45.37	6251.9053	13
G3V712_RAT	G3V712	keratin 7	Krt7	50.81	490.895	9
G3V741_RAT	G3V741	Solute carrier family 25 member 3	Slc25a3	39.53	20163.805	20
G3V784_RAT	G3V784	ADP-dependent glucokinase	Adpgk	53.92	1159.181	10
G3V7A5_RAT	G3V7A5	Low density lipoprotein receptor isoform	Ldlr	96.65	1045.3892	14
G3V7I0_RAT	G3V7I0	Peroxiredoxin-3	Prdx3	28.30	1017.8773	7
G3V7I3_RAT	G3V7I3	Manganese-transporting ATPase 13A1	Atp13a1	131.92	3255.567	36
G3V7L8_RAT	G3V7L8	V-type proton ATPase subunit E 1 isoform	Atp6v1e1	26.14	295.3137	12
G3V7M2_RAT	G3V7M2	Integrin beta 7	Itgb7	87.63	943.3401	13
G3V7N0_RAT	G3V7N0	Heparan sulfate 2-O-sulfotransferase 1	Hs2st1	41.85	2052.1785	9
G3V7N6_RAT	G3V7N6	Lysophospholipid acyltransferase 7	Mboat7l1	53.38	6267.8555	4
G3V7Q7_RAT	G3V7Q7	Ras GTPase-activating-like protein IQGAP1	Iqgap1	188.83	242.0032	20
G3V7U6_RAT	G3V7U6	Calpain 5	Capn5	84.51	2057.5771	20
G3V7Y9_RAT	P28494	Alpha-mannosidase 2	Man2a1	131.24	3329.9321	30
G3V878_RAT	G3V878	Signal peptidase complex catalytic subunit SEC11	Sec11c	21.66	4794.677	6
G3V887_RAT	G3V887	V-type proton ATPase subunit A	Tcirg1	93.21	1935.7375	14
G3V8F5_RAT	G3V8F5	Mitochondrial import receptor subunit TOM40 homolog	Tomm40	37.92	5112.55	10
G3V8G5_RAT	G3V8G5	Golgi apparatus protein 1	Glg1	133.55	5129.583	42
G3V8I4_RAT	G3V8I4	Syntaxin-4A	Stx4	34.20	1110.8849	8
G3V8L7_RAT	G3V8L7	Integrin alpha M	Itgam	127.19	2563.312	30
G3V8Q1_RAT	G3V8Q1	Coatomer subunit epsilon	Cope	34.65	1239.242	8
G3V8V4_RAT	G3V8V4	Ceramide synthase 2	Cers2	44.95	1771.848	5
G3V8Z1_RAT	G3V8Z1	Linker for activation of T-cells family member 2 (NTAL)	Lat2	23.10	6248.498	3
G3V908_RAT	G3V908	keratin, type II Kb15	Kb15	57.61	7247.892	15
G3V936_RAT	G3V936	Citrate synthase	Cs	51.83	1031.1398	6
G3V964_RAT	G3V964	Neurotrimin	Ntm	37.62	943.8691	6
G3V9N0_RAT	G3V9N0	Polyadenylate-binding protein 4	Pabpc4	70.83	1690.4026	16

G3V9N9_RAT	G3V9N9	alpha-1,2-Mannosidase	Man1a1	73.13	1150.4861	16
G3V9S3_RAT	G3V9S3	Tyrosine-protein phosphatase non-receptor type 13	Ptpn13	270.44	13.86	1
G3V9U7_RAT	G3V9U7	Sarcoplasmic/endoplasmic reticulum calcium ATPase 3 (SERCA3)	Atp2a3	109.37	8865.377	29
G3V9W2_RAT	G3V9W2	Tyrosine-protein kinase JAK1	Jak1	133.26	264.684	22
G3V9X8_RAT	G3V9X8	Magnesium transporter protein 1	Magt1	37.94	919.5995	4
G3V9Y2_RAT	G3V9Y2	Phospholipid phosphatase 1	Plpp1	31.91	616.2256	3
GALT1_RAT	Q10473	Polypeptide N-acetylgalactosaminyltransferase 1	Galnt1	64.23	828.2299	13
GALT7_RAT	Q9R0C5	Polypeptide N-acetylgalactosaminyltransferase 7	Galnt7	75.34	2017.703	13
GBB1_RAT	P54311	Transducin beta-1	Gnb1	37.38	40187.645	18
GBB2_RAT	P54313	Transducin beta-2	Gnb2	37.33	53010.633	16
GBB4_RAT	O35353	Transducin beta-4	Gnb4	37.36	18455	12
GFAP_RAT	P47819	Glial fibrillary acidic protein	Gfap	49.96	3110.5884	10
GHITM_RAT	Q5XIA8	Growth hormone-inducible transmembrane protein	Ghitm	37.18	1950.2825	5
GL8D1_RAT	Q6AYF6	Glycosyltransferase 8 domain-containing protein 1	Glt8d1	42.14	669.46295	5
GNA11_RAT	Q9JID2	Guanine nucleotide-binding protein alpha-11	Gna11	42.03	7346.748	10
GNAI2_RAT	P04897	Guanine nucleotide-binding protein G(i) subunit alpha-2	Gnai2	40.50	78538.42	26
GNAI3_RAT	P08753	Guanine nucleotide-binding protein G(k) subunit alpha	Gnai3	40.52	49947.156	24
GNAQ_RAT	P82471	Guanine nucleotide-binding protein G(q) subunit alpha	Gnaq	42.14	7457.616	15
GNAS2_RAT	P63095	Guanine nucleotide-binding protein G(s) subunit alpha	Gnas	45.66	16917.3	16
GNAZ_RAT	P19627	Guanine nucleotide-binding protein G(z) subunit alpha	Gnaz	40.88	8040.4985	13
GOGA2_RAT	Q62839	Golgin subfamily A member 2	Golga2	112.87	286.6845	12
GOGA7_RAT	Q6AYQ1	Golgin subfamily A member 7	Golga7	15.78	2409.3254	4
GOLI4_RAT	Q5BJK8	Golgi integral membrane protein 4	Golim4	76.60	4551.551	16
GOSR2_RAT	O35165	Golgi SNAP receptor complex member 2	Gosr2	24.61	1689.4465	4
GPDM_RAT	P35571	Glycerol-3-phosphate dehydrogenase mitochondrial	Gpd2	80.97	1379.1694	17
GPSM3_RAT	Q6MG88	G-protein-signaling modulator 3	Gpsm3	17.49	2360.417	3
GRP75_RAT	P48721	Mortalin	Hspa9	73.86	973.8264	18
GRP78_RAT	P06761	Endoplasmic reticulum chaperone BiP	Hspa5	72.35	13030.68	26
GTR1_RAT	P11167	Solute carrier family 2, facilitated glucose transporter member 1	Slc2a1	53.96	2702.7566	7
GTR3_RAT	Q07647	Solute carrier family 2, facilitated glucose transporter member 3	Slc2a3	53.58	37964.523	11
H14_RAT	P15865	Histone H1.4	Hist1h1e	21.99	1016.0143	4
H4_RAT	P62804	Histone H4	Hist1h4b; Hist1h4m; Hist4h4	11.37	2001.5176	5
HA12_RAT	P16391	RT1 class I histocompatibility antigen AA alpha chain	---	41.83	15494.76	14
HMOX1_RAT	P06762	Heme oxygenase 1	Hmox1	33.01	8042.461	12
HMOX2_RAT	P23711	Heme oxygenase 2	Hmox2	35.76	2565.247	10
HNRPF_RAT	Q794E4	Heterogeneous nuclear ribonucleoprotein F	Hnrnpf	45.73	286.0756	6
HRG1_RAT	B0BNL4	Heme transporter HRG1	Slc48a1	16.62	820.79425	8
HS90A_RAT	P82995	Heat shock protein HSP 90-alpha	Hsp90aa1	84.82	8623.396	18

HS90B_RAT	P34058	Heat shock protein HSP 90-beta	Hsp90ab1	83.28	11356.891	27
HSP72_RAT	P14659	Heat shock-related 70 kDa protein 2	Hspa2	69.64	14908.165	16
HSP7C_RAT	P63018	Heat shock cognate 71 kDa protein	Hspa8	70.87	41142.43	29
ICAM1_RAT	Q00238	Intercellular adhesion molecule 1 (ICAM1)	Icam1	60.14	3709.9238	12
IF4A2_RAT	Q5RKI1	Eukaryotic initiation factor 4A-II	Eif4a2	46.40	547.23773	6
IKIP_RAT	Q5EAJ6	Inhibitor kappa-B kinase-interacting protein	Ikbip	42.36	1422.366	9
ILRL1_RAT	Q62611	Interleukin 1 receptor-like 1	Il1rl1	64.41	9368.721	16
IMB1_RAT	P52296	Importin subunit beta-1	Kpnb1	97.12	663.5588	12
IMMT_RAT	Q3KR86	MICOS complex subunit Mic60	Immt	67.18	11029.786	21
IMPA3_RAT	D4AD37	Inositol monophosphatase 3	Impad1	38.57	5946.5176	7
IR3IP_RAT	P85007	Immediate early response 3-interacting protein 1	Ier3ip1	9.00	43136.105	2
IRK2_RAT	Q64273	Inward rectifier potassium channel 2	Kcnj2	48.20	453.43683	3
ITAD_RAT	Q9QYE7	Integrin alpha D	Itgad	126.60	915.6053	12
ITB1_RAT	P49134	Integrin beta 1	Itgb1	88.50	10606.656	20
ITFG3_RAT	Q5M7W6	Protein FAM234A	Fam234a	60.64	364.1404	6
ITM2B_RAT	Q5XIE8	Integral membrane protein 2B	Itm2b	30.31	1399.8596	6
ITM2C_RAT	Q5PQL7	Integral membrane protein 2C	Itm2c	30.48	8972.389	10
ITPR2_RAT	P29995	Inositol 1,4,5-trisphosphate receptor type 2	Itpr2	307.06	1859.219	59
ITPR3_RAT	Q63269	Inositol 1,4,5-trisphosphate receptor type 3	Itpr3	304.29	268.3315	38
K1C10_RAT	Q6IFW6	Keratin, type I cytoskeletal 10	Krt10	56.51	3125.5767	13
K1C14_RAT	Q6IFV1	Keratin, type I cytoskeletal 14	Krt14	52.68	1947.469	9
K1C15_RAT	Q6IFV3	Keratin, type I cytoskeletal 15	Krt15	48.87	2066.3047	9
K1C17_RAT	Q6IFU8	Keratin, type I cytoskeletal 17	Krt17	48.12	1520.085	9
K1C18_RAT	Q5BJY9	Keratin, type I cytoskeletal 18	Krt18	47.76	1424.4766	7
K1C42_RAT	Q6IFU7	Keratin, type I cytoskeletal 42	Krt42	50.21	1341.0945	10
K22E_RAT	Q6IG02	Keratin, type II cytoskeletal 2 epidermal	Krt2	69.13	1619.1815	8
K2C1_RAT	Q6IMF3	Keratin, type II cytoskeletal 1	Krt1	64.83	6467.131	15
K2C1B_RAT	Q6IG01	Keratin, type II cytoskeletal 1B	Krt77	57.26	2745.867	9
K2C4_RAT	Q6IG00	Keratin, type II cytoskeletal 4	Krt4	57.67	779.46643	13
K2C6A_RAT	Q4FZU2	Keratin, type II cytoskeletal 6A	Krt6a	59.25	5880.4443	11
K2C7_RAT	Q6IG12	Keratin, type II cytoskeletal 7	Krt7	50.71	3154.8394	12
K2C73_RAT	Q6IG03	Keratin, type II cytoskeletal 73	Krt73	60.39	5609.4053	11
K2C75_RAT	Q6IG05	Keratin, type II cytoskeletal 75	Krt75	59.03	3936.6782	12
K2C8_RAT	Q10758	Keratin, type II cytoskeletal 8	Krt8	54.02	3248.8926	12
KAP2_RAT	P12368	cAMP-dependent protein kinase type II-alpha regulatory subunit	Prkar2a	45.48	450.8543	5
KC1G1_RAT	Q62761	Casein kinase I isoform gamma-1	Csnk1g1	45.13	1386.6904	13
KC1G2_RAT	Q62762	Casein kinase I isoform gamma-2	Csnk1g2	47.64	1022.759	10
KC1G3_RAT	Q62763	Casein kinase I isoform gamma-3	Csnk1g3	51.43	2977.0906	14

KPYM_RAT	P11980	Pyruvate kinase PKM	Pkm	57.82	3335.2876	14
LAMP1_RAT	P14562	Lysosome-associated membrane glycoprotein 1 (Lamp1)	Lamp1	43.97	2130.0125	7
LAMP2_RAT	P17046	Lysosome-associated membrane glycoprotein 2 (Lamp2)	Lamp2	45.16	1748.5774	4
LAP2_RAT	Q62733	Lamina-associated polypeptide 2 isoform beta	Tmpo	50.28	3172.5151	12
LAT_RAT	O70601	Linker for activation of T-cells family member 1 (LAT)	Lat	26.19	4592.621	7
LAT1_RAT	Q63016	Large neutral amino acids transporter small subunit 1	Slc7a5	55.90	1773.999	7
LBR_RAT	O08984	Lamin-B receptor	Lbr	70.72	2114.675	11
LDHA_RAT	P04642	L-lactate dehydrogenase A chain	Ldha	36.45	1576.633	9
LDLR_RAT	P35952	Low-density lipoprotein receptor	Ldlr	96.62	1431.886	14
LETM1_RAT	Q5XIN6	Mitochondrial proton/calcium exchanger protein	Letm1	83.06	826.77905	13
LEUK_RAT	P13838	Leukosialin	Spn	38.43	1175.2317	5
LMAN1_RAT	Q62902	Lectin mannose-binding 1	Lman1	57.96	6617.7812	13
LMBD1_RAT	Q6AZ61	Probable lysosomal cobalamin transporter	Lmbrd1	61.02	826.9542	1
LMF2_RAT	A1L1J9	Lipase maturation factor 2	Lmf2	80.25	1244.6265	7
LPPRC_RAT	Q5SGE0	Leucine-rich PPR motif-containing protein mitochondrial	Lrpprc	156.65	1723.363	33
LRC59_RAT	Q5RJR8	Leucine-rich repeat-containing protein 59	Lrrc59	34.87	9316.835	11
LTOR1_RAT	Q6P791	Ragulator complex protein LAMTOR1	Lamtor1	17.72	20131.82	11
LTOR3_RAT	Q5U204	Ragulator complex protein LAMTOR3	Lamtor3	13.58	18707.53	4
LYN_RAT	Q07014	Tyrosine-protein kinase Lyn	Lyn	58.66	62104.484	33
M0R402_RAT	M0R402	Protein disulfide-isomerase TMX3	Tmx3	52.10	759.8056	10
M0RAJ8_RAT	M0RAJ8	40S ribosomal protein S2	---	31.41	2199.025	4
M0RBJ0_RAT	M0RBJ0	Guanine nucleotide-binding protein gamma-2	Gng2	4.88	51315.395	5
M0RC99_RAT	M0RC99	Ras-related protein Rab-5a	Rab5a	23.63	7851.471	7
M0RCA3_RAT	M0RCA3	Zinc finger protein-like 1	Zfpl1	34.25	2976.191	6
M0RCF3_RAT	M0RCF3	Integrin beta 3	Itgb3	87.08	4581.119	21
M0RCS2_RAT	M0RCS2	Transducin beta-like protein 2	Tbl2	17.40	1252.123	13
M0RD75_RAT	M0RD75	40S ribosomal protein S6	Rps6	28.42	2664.179	7
M0RDI5_RAT	M0RDI5	Mitochondrial calcium uniporter	Mcu	39.76	1066.463	7
MAVS_RAT	Q66HG9	Mitochondrial antiviral-signaling protein	Mavs	53.81	1582.96	8
MBOA5_RAT	Q5FVN0	Lysophospholipid acyltransferase 5	Lpcat3	56.02	4474.037	8
MDHM_RAT	P04636	Malate dehydrogenase mitochondrial	Mdh2	35.68	1374.79	9
MGAT2_RAT	Q09326	Alpha-1,6-mannosyl-glycoprotein 2-beta-N-acetylglucosaminyltransferase	Mgat2	51.11	1614.162	11
MIRO2_RAT	Q7TSA0	Mitochondrial Rho GTPase 2	Rhot2	69.15	564.2921	9
MLEC_RAT	Q5FVQ4	Malectin	Mlec	32.42	17085.227	13
MMGT1_RAT	B5DF51	Membrane magnesium transporter 1	Mmgt1	14.68	4324.251	2
MOGS_RAT	O88941	Mannosyl-oligosaccharide glucosidase	Mogs	91.87	726.93286	16
MOSC2_RAT	O88994	Mitochondrial amidoxime reducing component 2	Marc2	38.25	1441.2881	8
MOT1_RAT	P53987	Monocarboxylate transporter 1	Slc16a1	53.24	9277.2	7

MRLCA_RAT	P13832	Myosin regulatory light chain RLC-A	Rlc-a	19.90	2851.8682	7
MSMO1_RAT	O35532	Methylsterol monooxygenase 1	Msmo1	34.96	3108.441	3
MYH10_RAT	Q9JLT0	Myosin heavy chain 10	Myh10	228.97	502.0399	26
MYL6_RAT	Q64119	Myosin light polypeptide 6	Myl6	16.98	12424.145	9
NB5R3_RAT	P20070	NADH-cytochrome b5 reductase 3	Cyb5r3	34.18	2120.4146	7
NCLN_RAT	Q5XIA1	Nicalin	Ncln	62.99	6718.4053	13
NCPR_RAT	P00388	NADPH--cytochrome P450 reductase	Por	76.96	9740.773	30
NCUG1_RAT	Q68FV6	Glycosylated lysosomal membrane protein	Glmp	43.87	517.7008	2
NDUAA_RAT	Q561S0	NADH dehydrogenase [ubiquinone] 1 alpha subcomplex subunit 10	Ndufa10	40.49	412.8998	6
NDUF5_RAT	B2GV71	NADH dehydrogenase [ubiquinone] 1 alpha subcomplex assembly factor 5	Ndufaf5	38.23	310.12567	3
NDUS1_RAT	Q66HF1	NADH-ubiquinone oxidoreductase 75 kDa subunit mitochondrial	Ndufs1	79.41	1264.7273	19
NDUS2_RAT	Q641Y2	NADH dehydrogenase [ubiquinone] iron-sulfur protein 2	Ndufs2	52.56	838.103	9
NDUV2_RAT	P19234	NADH dehydrogenase [ubiquinone] flavoprotein 2 mitochondrial	Ndufv2	27.38	2760.076	8
NICA_RAT	Q8CGU6	Nicastrin	Ncstn	78.40	7369.522	12
NPTN_RAT	P97546	Neuroplastin	Nptn	43.93	624.8021	6
NUCB1_RAT	Q63083	Nucleobindin-1	Nucb1	53.51	2050.4395	16
NUCB2_RAT	Q9JI85	Nucleobindin-2	Nucb2	50.09	4984.8174	17
NUCL_RAT	P13383	Nucleolin	Ncl	77.15	3949.7266	21
O89035_RAT	O89035	Solute carrier family 25 member 10	Slc25a10	31.46	1959.6011	10
OCAD1_RAT	Q5XIG4	OCIA domain-containing protein 1	Ociad1	27.66	2874.1665	6
ODO1_RAT	Q5XI78	2-oxoglutarate dehydrogenase complex component E1	Ogdh	116.30	462.29266	18
ODO2_RAT	Q01205	2-oxoglutarate dehydrogenase complex component E2	Dlst	48.93	1009.41	8
ODP2_RAT	P08461	Pyruvate dehydrogenase component E2	Dlat	67.17	1237.4315	15
ODPB_RAT	P49432	Pyruvate dehydrogenase component E1 beta	Pdhb	38.98	3007.9316	11
OGFD3_RAT	Q5M843	2-oxoglutarate and iron-dependent oxygenase domain-containing protein 3	Ogfod3	35.39	1258.4572	6
OS9_RAT	Q5RKH6	Protein OS-9	Os9	75.37	1507.2655	18
OST48_RAT	Q641Y0	Oligosaccharyl transferase 48 kDa subunit	Ddost	48.90	22532.98	17
P2RX4_RAT	P51577	P2X purinoceptor 4	P2rx4	43.50	2251.637	9
P4K2B_RAT	Q5XIL2	Phosphatidylinositol 4-kinase type 2-beta	Pi4k2b	54.46	1195.6467	11
PABP1_RAT	Q9EPH8	Polyadenylate-binding protein 1	Pabpc1	70.70	2639.031	20
PBIP1_RAT	A2VD12	Pre-B-cell leukemia transcription factor-interacting protein 1	Pbxip1	80.28	925.7483	12
PCAT2_RAT	P0C1Q3	Lysophosphatidylcholine acyltransferase 2	Lpcat2	59.83	698.8402	7
PDE3B_RAT	Q63085	Cyclic GMP-inhibited phosphodiesterase B	Pde3b	123.11	821.0266	20
PDIA1_RAT	P04785	Protein disulfide-isomerase	P4hb	56.95	3330.268	18
PDIA3_RAT	P11598	Protein disulfide-isomerase A3	Pdia3	56.62	17165.145	28
PECA1_RAT	Q3SWT0	Platelet endothelial cell adhesion molecule (Pecam1)	Pecam1	76.19	2114.614	16
PGFRB_RAT	Q05030	Platelet-derived growth factor receptor beta	Pdgfrb	122.83	184.1114	6
PGRC1_RAT	P70580	Membrane-associated progesterone receptor component 1	Pgrmc1	21.60	8160.259	11

PGRC2_RAT	Q5XIU9	Membrane-associated progesterone receptor component 2	Pgrmc2	23.40	13782.562	10
PHB_RAT	P67779	Prohibitin	Phb	29.82	39984.395	17
PHB2_RAT	Q5XIH7	Prohibitin 2	Phb2	33.31	23869.629	17
PHOCN_RAT	Q9QYW3	MOB-like protein phocein	Mob4	26.03	2233.6565	6
PIGS_RAT	Q5XI31	GPI transamidase component PIG-S	Pigs	61.72	3193.355	14
PLF4_RAT	P06765	Platelet factor 4	Pf4	11.29	1582.359	2
PLP2_RAT	Q6P742	Proteolipid protein 2	Plp2	16.56	7078.4824	3
PLS1_RAT	P58195	Phospholipid scramblase 1	Plscr1	36.71	11390.705	6
PLS3_RAT	Q6QBQ4	Phospholipid scramblase 3	Plscr3	31.65	754.14386	4
PORED_RAT	Q5RJM1	3-oxo-5-alpha-steroid 4-dehydrogenase 3	Srd5a3	38.09	2307.13	5
PP1A_RAT	P62138	Serine/threonine-protein phosphatase PP1-alpha catalytic subunit	Ppp1ca	37.51	741.3635	10
PPIA_RAT	P10111	Peptidyl-prolyl cis-trans isomerase A	Ppia	17.87	813.9749	11
PPIB_RAT	P24368	Peptidyl-prolyl cis-trans isomerase B	Ppib	23.80	322.5537	5
PRAF3_RAT	Q9ES40	PRA1 family protein 3	Arl6ip5	21.55	2357.312	8
PRDX1_RAT	Q63716	Peroxiredoxin-1	Prdx1	22.11	2065.4648	8
PRDX3_RAT	Q9Z0V6	Thioredoxin-dependent peroxide reductase mitochondrial	Prdx3	28.30	917.41174	6
PROF1_RAT	P62963	Profilin-1	Pfn1	14.96	1022.584	4
PSN1_RAT	P97887	Presenilin-1	Psen1	52.79	409.77258	4
PSN2_RAT	O88777	Presenilin-2	Psen2	50.05	459.7473	4
PTN1_RAT	P20417	Protein-tyrosine phosphatase 1B	Ptpn1	49.67	6730.742	15
PTPRC_RAT	P04157	Receptor-type tyrosine-protein phosphatase C (CD45)	Ptprc	143.27	2727.253	34
Q0R3W9_RAT	Q0R3W9	GTPase, IMAP family member 1	Gimap1	33.31	6332.2266	12
Q2I6B1_RAT	Q2I6B1	V-type proton ATPase subunit A2	Atp6v0a2	98.04	1081.5837	17
Q2I6B2_RAT	Q2I6B2	V-type proton ATPase subunit A1	Atp6v0a1	95.59	10632.083	29
Q2IBC7_RAT	Q2IBC7	Hepatocyte growth factor receptor	Met	153.96	82.4271	1
Q32PX6_RAT	Q32PX6	Rho-related GTP-binding protein RhoG	Rhog	21.31	27716	14
Q3B7V5_RAT	Q3B7V5	Ras-related protein Rab-2b	Rab2b	24.09	12735.811	11
Q3KRC3_RAT	Q3KRC3	Signal recognition particle receptor alpha	Srpra	69.66	2062.8618	11
Q3MHS9_RAT	Q3MHS9	Chaperonin-containing TCP1 subunit 6A	Cct6a	58.02	915.759	10
Q497A2_RAT	Q497A2	Solute carrier family 35 member B2	Slc35b2	47.30	956.06506	5
Q4KM97_RAT	Q4KM97	Tyrosine-protein kinase BLK	Blk	56.65	491.4534	6
Q4QQS6_RAT	Q4QQS6	Dolichyl-phosphate beta-glucosyltransferase	Alg5	36.86	1178.263	8
Q4QQV0_RAT	Q4QQV0	Tubulin beta-6	Tubb6	50.06	3483.87	8
Q4V7D1_RAT	Q4V7D1	Translocon-associated protein subunit alpha	Ssr1	32.21	14023.68	5
Q4V7D9_RAT	Q4V7D9	Acid sphingomyelinase-like phosphodiesterase 3B	Smpdl3b	51.65	9802.894	12
Q4V8N6_RAT	Q4V8N6	Solute carrier organic anion transporter family member 2A1	Slco4a1	77.69	705.52014	9
Q52KJ9_RAT	Q52KJ9	Thioredoxin-related transmembrane protein 1	Tmx1	31.44	2166.8906	6
Q5BJZ3_RAT	Q5BJZ3	Nicotinamide nucleotide transhydrogenase	Nnt	113.87	429.1828	18

Q5EAP4_RAT	Q5EAP4	Guanine nucleotide binding protein alpha-14	Gna14	41.44	3949.9775	14
Q5FVC4_RAT	Q5FVC4	DnaJ (Hsp40) homolog subfamily B member 12	Dnajb12	42.52	1014.84045	9
Q5M7T6_RAT	Q5M7T6	V-type proton ATPase subunit D 1	Atp6v0d1	40.30	9359.584	17
Q5RK10_RAT	Q5RK10	60S ribosomal protein L13a, isoform	Rpl13a	23.45	2532.1545	9
Q5RKJ9_RAT	Q5RKJ9	Ras-related protein Rab-10	Rab10	22.54	28772.68	7
Q5U1Y2_RAT	Q5U1Y2	Ras-related C3 botulinum toxin substrate 2 (Rac2)	Rac2	21.44	26561.215	9
Q5U1Z9_RAT	Q5U1Z9	Metaxin 2	Mtx2	29.72	2446.838	5
Q5U2Z8_RAT	Q5U2Z8	Elongation of very long chain fatty acids protein 1	Elovl1	32.64	784.149	1
Q5U3Z7_RAT	Q5U3Z7	Serine hydroxymethyltransferase	Shmt2	55.77	639.08356	9
Q5VLR5_RAT	Q5VLR5	Endoplasmic reticulum resident protein 44	Erp44	46.88	4631.925	11
Q5XI04_RAT	Q5XI04	Erythrocyte band 7 integral membrane protein	Stom	31.38	1374.209	8
Q5XI34_RAT	Q5XI34	Serine/threonine-protein phosphatase 2A 65 kDa regulatory subunit A alpha isoform	Ppp2r1a	65.32	853.00256	14
Q5XI36_RAT	Q5XI36	CD97	Adgre5	91.14	990.31195	11
Q5XI86_RAT	Q5XI86	Peptidyl-tRNA hydrolase 2	Ptrh2	19.53	16233.149	10
Q5XIH3_RAT	Q5XIH3	NADH dehydrogenase [ubiquinone] flavoprotein 1	Ndufv1	50.73	1457.5854	10
Q5XIP2_RAT	Q5XIP2	GPI-anchor transamidase	Pigk	44.89	2760.516	12
Q5XIU4_RAT	Q5XIU4	B-cell receptor-associated protein 29	Bcap29	28.23	662.509	4
Q63116_RAT	Q63116	Mast/stem cell growth factor receptor	Kit	109.34	1779.2214	24
Q63483_RAT	Q63483	Ras-related protein Rab-38	Rab38	23.78	4574.048	6
Q642E3_RAT	Q642E3	CDK5 regulatory subunit-associated protein 3, isoform	Cdk5rap3	57.14	4572.61	16
Q66H18_RAT	Q66H18	Synaptophysin-like 1	Sypl1	28.63	3762.5981	5
Q6AXX5_RAT	Q6AXX5	Retinol dehydrogenase 11	Rdh11	35.12	1461.48	6
Q6AY18_RAT	Q6AY18	GTP-binding protein SAR1a	Sar1a	22.40	1340.813	4
Q6AYD3_RAT	Q6AYD3	Proliferation-associated protein 2G4	Pa2g4	43.66	899.6481	10
Q6AYI1_RAT	Q6AYI1	Probable ATP-dependent RNA helicase DDX5	Ddx5	69.24	1868.138	13
Q6AYM8_RAT	Q6AYM8	Glycosylphosphatidylinositol anchor attachment 1	Gpaa1	67.97	564.3983	7
Q6AYQ9_RAT	Q6AYQ9	Peptidyl-prolyl cis-trans isomerase C	Ppic	23.01	3430.5244	7
Q6IFU9_RAT	Q6IFU9	keratin 16	Krt16	50.78	773.24097	7
Q6IFV6_RAT	Q6IFV6	Keratin 35	Krt35	50.68	1185.366	5
Q6IFZ5_RAT	Q6IFZ5	Keratin, type II cytoskeletal 2 oral	Krt76	61.76	711.88745	12
Q6IN24_RAT	Q6IN24	Galectin-8	Lgals8	36.07	1860.6	10
Q6IRF8_RAT	Q6IRF8	V-type proton ATPase subunit S1	Atp6ap1	51.10	1346.6022	5
Q6MGA3_RAT	Q6MGA3	Antigen peptide transporter 2	Tap2	77.75	14208.09	22
Q6MGB4_RAT	Q6MGB4	H2-K region expressed gene 4, rat orthologue	Slc39a7	49.50	511.51898	4
Q6P3V8_RAT	Q6P3V8	Eukaryotic initiation factor 4A-I	Eif4a1	46.15	1732.11	8
Q6P3V9_RAT	Q6P3V9	60S ribosomal protein L4	Rpl4	47.30	2003.3816	18
Q6P503_RAT	Q6P503	V-type proton ATPase subunit D	Atp6v1d	28.31	1400.0286	7
Q6P685_RAT	Q6P685	Eukaryotic translation initiation factor 2 subunit 2	Eif2s2	38.24	336.0998	8

Q6P9V1_RAT	Q6P9V1	CD81 molecule	Cd81	25.91	3985.6934	4
Q6PCU4_RAT	Q6PCU4	Emerin	Emd	29.59	2569.4375	12
Q6PDV6_RAT	Q6PDV6	40S ribosomal protein S14	LOC100911847	16.27	2446.334	3
Q76GL9_RAT	Q76GL9	Neutral amino acid transporter A	Slc1a4	55.90	2930.5596	7
Q78P65_RAT	Q78P65	Rap2A-like protein	Rap2a	11.95	26551.25	6
Q7TP42_RAT	Q7TP42	Translocation protein SEC62	Sec62	67.93	1620.406	8
Q7TQ86_RAT	Q7TQ86	Ac1158	Rsl1d1	60.96	802.25543	6
Q8SEZ5_RAT	Q8SEZ5	Cytochrome c oxidase subunit 2	Mt-co2	25.93	5020.634	4
Q99JC6_RAT	Q99JC6	Tapasin	Tapbp	50.05	7855.967	11
Q9Z1J7_RAT	Q9Z1J7	Amino acid transporter	Slc1a5	56.76	1423.9875	6
QCR1_RAT	Q68FY0	Cytochrome b-c1 complex subunit 1 mitochondrial	Uqcrc1	52.85	8080.3516	15
QCR2_RAT	P32551	Cytochrome b-c1 complex subunit 2 mitochondrial	Uqcrc2	48.40	7736.858	15
QCR6_RAT	Q5M9I5	Cytochrome b-c1 complex subunit 6 mitochondrial	Uqcrh	10.42	13177.475	4
R9PXR4_RAT	R9PXR4	Mitochondrial import receptor subunit TOM70	Tomm70	61.94	6584.302	21
RAB14_RAT	P61107	Ras-related protein Rab-14	Rab14	23.93	40040.32	16
RAB15_RAT	P35289	Ras-related protein Rab-15	Rab15	24.28	16062.471	5
RAB18_RAT	Q5EB77	Ras-related protein Rab-18	Rab18	22.98	5795.3335	11
RAB1A_RAT	Q6NYB7	Ras-related protein Rab-1a	Rab1A	22.68	65807.23	13
RAB21_RAT	Q6AXT5	Ras-related protein Rab-21	Rab21	24.16	4898.142	9
RAB2A_RAT	P05712	Ras-related protein Rab-2a	Rab2a	23.54	30861.914	15
RAB35_RAT	Q5U316	Ras-related protein Rab-35	Rab35	23.03	9651.083	9
RAB3A_RAT	P63012	Ras-related protein Rab-3a	Rab3a	24.97	4949.498	5
RAB3D_RAT	Q63942	Ras-related protein Rab-3d	Rab3d	24.29	5246.023	7
RAB43_RAT	Q53B90	Ras-related protein Rab-43	Rab43	23.23	4229.5757	6
RAB4B_RAT	P51146	Ras-related protein Rab-4b	Rab4b	23.63	8660.258	10
RAB6A_RAT	Q9WVB1	Ras-related protein Rab-6a	Rab6a	23.59	15014.664	10
RAB7A_RAT	P09527	Ras-related protein Rab-7a	Rab7a	23.50	49333.57	17
RAB8A_RAT	P35280	Ras-related protein Rab-8a	Rab8a	23.67	25169.18	13
RAB8B_RAT	P70550	Ras-related protein Rab-8b	Rab8b	23.60	26870.184	10
RAB9A_RAT	Q99P75	Ras-related protein Rab-9a	Rab9a	22.90	1927.4304	7
RAC1_RAT	Q6RUV5	Ras-related C3 botulinum toxin substrate 1 (Rac1)	Rac1	21.45	12083.035	8
RALA_RAT	P63322	Ras-related protein Ral-A	Rala	23.55	13274.745	8
RAP1A_RAT	P62836	Ras-related protein Rap-1a	Rap1a	20.99	29839.578	13
RAP1B_RAT	Q62636	Ras-related protein Rap-1b	Rap1b	20.80	61852.535	16
RAP2B_RAT	P61227	Ras-related protein Rap-2b	Rap2b	20.50	33133.664	11
RASH_RAT	P20171	GTPase HRas	Hras	21.30	7927.7456	9
RASK_RAT	P08644	GTPase KRas	Kras	21.66	9070.234	12
RASN_RAT	Q04970	GTPase NRas	Nras	21.24	10836.549	12

RB11B_RAT	O35509	Ras-related protein Rab-11b	Rab11b	24.49	20963.8	13
RB27A_RAT	P23640	Ras-related protein Rab-27a	Rab27a	25.07	19213.395	12
RB27B_RAT	Q99P74	Ras-related protein Rab-27b	Rab27b	24.62	18974.406	14
RENR_RAT	Q6AXS4	Renin receptor	Atp6ap2	39.08	1127.7795	9
RER1_RAT	Q498C8	Protein RER1	Rer1	22.99	3561.753	6
RGS19_RAT	O70521	Regulator of G-protein signaling 19	Rgs19	24.74	4484.95	7
RHBL4_RAT	Q4V8F3	Rhomboid-related protein 4	Rhbdd1	35.88	757.19183	4
RHOA_RAT	P61589	Transforming protein RhoA	Rhoa	21.78	47836.855	12
RHOB_RAT	P62747	Rho-related GTP-binding protein RhoB	Rhob	22.12	20034.904	6
RL10_RAT	Q6PDV7	60S ribosomal protein L10	Rpl10	24.60	3478.3306	8
RL10A_RAT	P62907	60S ribosomal protein L10a	Rpl10a	24.83	3409.09	6
RL11_RAT	P62914	60S ribosomal protein L11	Rpl11	20.25	3160.7769	4
RL12_RAT	P23358	60S ribosomal protein L12	Rpl12	17.85	8159.423	6
RL13A_RAT	P35427	60S ribosomal protein L13a	Rpl13a	23.48	2532.1545	9
RL14_RAT	Q63507	60S ribosomal protein L14	Rpl14	23.34	4102.905	4
RL17_RAT	P24049	60S ribosomal protein L17	Rpl17	21.40	1644.8086	3
RL18_RAT	P12001	60S ribosomal protein L18	Rpl18	21.66	5652.0596	5
RL18A_RAT	P62718	60S ribosomal protein L18a	Rpl18a	20.73	3227.7095	7
RL19_RAT	P84100	60S ribosomal protein L19	Rpl19	23.47	4241.6533	4
RL23_RAT	P62832	60S ribosomal protein L23	Rpl23	14.87	3088.36	4
RL24_RAT	P83732	60S ribosomal protein L24	Rpl24	17.78	1627.648	2
RL3_RAT	P21531	60S ribosomal protein L3	Rpl3	46.14	1587.5591	13
RL30_RAT	P62890	60S ribosomal protein L30	Rpl30	12.78	11503.277	5
RL40_RAT	P62986	Ubiquitin-60S ribosomal protein L40	Uba52	14.73	35746.945	6
RL7_RAT	P05426	60S ribosomal protein L7	Rpl7	30.33	5262.581	9
RLA0_RAT	P19945	60S acidic ribosomal protein P0	Rplp0	34.22	7699.7544	9
RLA1_RAT	P19944	60S acidic ribosomal protein P1	Rplp1	11.50	55449.68	2
RMD3_RAT	Q66H15	Regulator of microtubule dynamics protein 3	Rmdn3	52.31	2174.5369	8
RPN2_RAT	P25235	Dolichyl-diphosphooligosaccharide protein glycosyltransferase subunit 2	Rpn2	69.08	42825.945	25
RRAGA_RAT	Q63486	Ras-related GTP-binding protein A (Rag A)	Rraga	36.57	1977.4419	7
RS11_RAT	P62282	40S ribosomal protein S11	Rps11	18.43	2837.272	6
RS13_RAT	P62278	40S ribosomal protein S13	Rps13	17.22	6866.343	7
RS15A_RAT	P62246	40S ribosomal protein S15a	Rps15a	14.84	1937.288	5
RS16_RAT	P62250	40S ribosomal protein S16	Rps16	16.45	3674.629	7
RS27A_RAT	P62982	Ubiquitin-40S ribosomal protein S27a	Rps27a	17.95	37445.46	8
RS28_RAT	P62859	40S ribosomal protein S28	Rps28	7.84	3012.5073	2
RS3_RAT	P62909	40S ribosomal protein S3	Rps3	26.67	4638.574	12
RS3A_RAT	P49242	40S ribosomal protein S3a	Rps3a	29.95	3326.7974	12

RS4X_RAT	P62703	40S ribosomal protein S4, X isoform	Rps4x	29.60	1144.8865	10
RS7_RAT	P62083	40S ribosomal protein S7	Rps7	22.13	3930.561	5
RS8_RAT	P62243	40S ribosomal protein S8	Rps8	24.21	8867.166	10
RSSA_RAT	P38983	40S ribosomal protein SA	Rpsa	32.82	2309.031	7
RTN4_RAT	Q9JK11	Reticulon-4	Rtn4	126.39	1037.768	14
S38A2_RAT	Q9JHE5	Sodium-coupled neutral amino acid transporter 2	Slc38a2	55.55	4436.6104	5
S61A1_RAT	P61621	SEC61 alpha-1	Sec61a1	52.27	5403.4395	9
SAC1_RAT	Q9ES21	Phosphatidylinositide phosphatase SAC1	Sacm1l	67.04	1569.263	19
SAM50_RAT	Q6AXV4	Sorting and assembly machinery component 50 homolog	Samm50	51.96	1186.929	14
SAP_RAT	P10960	Prosaposin	Psap	61.12	276.0468	9
SARAF_RAT	Q6AYN2	Store-operated calcium entry-associated regulatory factor (SARAF)	Saraf	35.89	972.8738	4
SC11A_RAT	P42667	SEC11 homolog A	Sec11a	20.60	12103.855	10
SC11C_RAT	Q9WTR7	SEC11 homolog C	Sec11c	21.65	4794.677	6
SC22B_RAT	Q4KM74	Vesicle-trafficking protein SEC22b	Sec22b	24.74	34632.867	14
SCFD1_RAT	Q62991	Syntaxin-binding protein 1-like 2	Scfd1	72.26	1780.61	11
SCRB2_RAT	P27615	Lysosome membrane protein 2 (CD36)	Scarb2	54.09	2124.119	9
SELT_RAT	Q1H5H1	Thioredoxin reductase-like selenoprotein T	Selenot	22.29	4981.218	5
SFXN1_RAT	Q63965	Sideroflexin-1	Sfxn1	35.55	12449.84	10
SGPL1_RAT	Q8CHN6	Sphingosine-1-phosphate lyase 1	Sgpl1	63.76	1971.3015	8
SIA4C_RAT	P61131	Beta-galactoside alpha-2,3-sialyltransferase 4	St3gal4	38.04	1940.3485	11
SNAA_RAT	P54921	Alpha-soluble NSF attachment protein (SNAP-alpha)	Napa	33.19	20788.06	18
SND1_RAT	Q66X93	Staphylococcal nuclease domain-containing protein 1	Snd1	101.95	557.89636	20
SNG1_RAT	Q62876	Synaptogyrin-1	Syngr1	25.67	1934.197	2
SNG2_RAT	O54980	Synaptogyrin-2	Syngr2	24.71	1547.865	2
SNP23_RAT	O70377	Synaptosomal-associated protein 23 (SNAP-23)	Snap23	23.24	44422.52	12
SO4A1_RAT	Q99N01	Organic anion-transporting polypeptide E	Slco4a1	77.63	725.4213	10
SPNS1_RAT	Q2YDU8	Protein spinster homolog 1	Spns1	56.80	857.2124	5
SQSTM_RAT	O08623	Sequestosome-1	Sqstm1	47.68	660.8219	7
SRPRB_RAT	Q4FZX7	Signal recognition particle receptor beta	Srprb	29.57	5558.2905	13
SSRD_RAT	Q07984	Translocon-associated protein subunit delta	Ssr4	18.98	36080.1	8
STIM1_RAT	P84903	Stromal interaction molecule 1 (STIM1)	Stim1	77.45	3978.3716	21
STML2_RAT	Q4FZT0	Stomatin-like protein 2	Stoml2	38.41	4983.7925	13
STRN3_RAT	P58405	Striatin-3	Strn3	87.11	330.0479	8
STX12_RAT	G3V7P1	Syntaxin-12	Stx12	31.19	24137.426	11
STX3_RAT	Q08849	Syntaxin-3	Stx3	33.26	3255.2964	8
STX5_RAT	Q08851	Syntaxin-5	Stx5	39.75	1804.198	10
STX6_RAT	Q63635	Syntaxin-6	Stx6	29.06	1157.439	6
STX7_RAT	O70257	Syntaxin-7	Stx7	29.85	13255.42	9

STX8_RAT	Q9Z2Q7	Syntaxin-8	Stx8	26.91	2008.0415	6
STXB2_RAT	Q62753	Syntaxin-binding protein 2	Stxbp2	66.70	3765.574	24
STXB5_RAT	Q9WU70	Syntaxin-binding protein 5	Stxbp5	127.66	825.01843	14
SYVC_RAT	Q04462	Valine--tRNA ligase	Vars	140.37	315.0938	21
T106B_RAT	Q6AYA5	Transmembrane protein 106 B	Tmem106b	31.15	766.6193	5
T120A_RAT	Q5HZE2	Transmembrane protein 120 A	Tmem120a	40.66	2023.4355	8
TAP1_RAT	P36370	Antigen peptide transporter 1	Tap1	79.15	7183.5654	22
TAXB1_RAT	Q66HA4	Tax1-binding protein 1 homolog	Tax1bp1	93.20	1382.8915	20
TBA1B_RAT	Q6P9V9	Tubulin alpha-1B	Tuba1b	50.15	21971.31	18
TBA1C_RAT	Q6AYZ1	Tubulin alpha-1C	Tuba1c	49.94	19631.385	17
TBA4A_RAT	Q5XIF6	Tubulin alpha-4A	Tuba4a	49.92	9072.143	16
TBA8_RAT	Q6AY56	Tubulin alpha-8	Tuba8	50.04	5823.075	14
TBB4B_RAT	Q6P9T8	Tubulin beta-4B	Tubb4b	49.80	825.013	6
TBB5_RAT	P69897	Tubulin beta-5	Tubb5	49.67	10868.01	17
TCPB_RAT	Q5XIM9	T-complex protein 1 beta	Cct2	57.46	490.99457	8
TCPE_RAT	Q68FQ0	T-complex protein 1 epsilon	Cct5	59.54	848.6412	9
TECR_RAT	Q64232	Very-long-chain enoyl-CoA reductase	Tecr	36.12	2042.097	13
TERA_RAT	P46462	Transitional endoplasmic reticulum ATPase	Vcp	89.35	9863.038	37
TGON3_RAT	P19814	Trans-Golgi network integral membrane protein TGN38	Ttgn1	38.31	1215.6049	6
THY1_RAT	P01830	Thy-1 membrane glycoprotein	Thy1	18.17	46873.016	8
TKT_RAT	P50137	Transketolase	Tkt	67.64	1239.818	14
TM109_RAT	Q6AYQ4	Transmembrane protein 109	Tmem109	26.24	851.1362	1
TM165_RAT	Q4V899	Transmembrane protein 165	Tmem165	34.81	17560.244	6
TM168_RAT	Q5PQM0	Transmembrane protein 168	Tmem168	79.77	3365.133	12
TM38B_RAT	Q68FV1	Trimeric intracellular cation channel type B (TRIC-B)	Tmem38b	32.45	2604.358	6
TM55A_RAT	Q4V888	Type 2 phosphatidylinositol 4,5-bisphosphate 4-phosphatase	Pip4p2	28.02	5976.5205	8
TM7S3_RAT	Q5FVF4	Transmembrane 7 superfamily member 3	Tm7sf3	63.59	1565.8905	6
TM9S1_RAT	Q66HF2	Transmembrane 9 superfamily member 1	Tm9sf1	66.92	840.3792	14
TM9S2_RAT	Q66HG5	Transmembrane 9 superfamily member 2	Tm9sf2	75.59	5912.821	13
TM9S4_RAT	Q4KLL4	Transmembrane 9 superfamily member 4	Tm9sf4	74.68	4822.549	19
TMCO1_RAT	Q5I0H4	Calcium load-activated calcium channel (CLAC channel)	Tmco1	21.18	2068.694	3
TMED1_RAT	Q5BK85	Transmembrane emp24 domain-containing protein 1	Tmed1	25.21	1818.857	5
TMED2_RAT	Q63524	Transmembrane emp24 domain-containing protein 2	Tmed2	22.73	10404.914	7
TMED5_RAT	Q6AXN3	Transmembrane emp24 domain-containing protein 5	Tmed5	26.14	4953.7896	8
TMED7_RAT	D3ZTX0	Transmembrane emp24 domain-containing protein 7	Tmed7	25.48	13459.775	9
TMED9_RAT	Q5I0E7	Transmembrane emp24 domain-containing protein 9	Tmed9	27.03	23465.781	9
TMEDA_RAT	Q63584	Transmembrane emp24 domain-containing protein 10	Tmed10	24.86	35308.4	12
TMM43_RAT	Q5XIP9	Transmembrane protein 43 (Protein LUMA)	Tmem43	44.77	969.2106	9

TMX2_RAT	Q5XIK2	Thioredoxin-related transmembrane protein 2	Tmx2	33.87	2550.555	7
TOIP1_RAT	Q5PQX1	Torsin-1A-interacting protein 1	Tor1aip1	65.65	2439.1387	13
TOM22_RAT	Q75Q41	Mitochondrial import receptor subunit TOM22 homolog	Tomm22	15.49	5316.167	5
TPM3_RAT	Q63610	Tropomyosin alpha-3	Tpm3	29.01	158.7291	3
TPM4_RAT	P09495	Tropomyosin alpha-4	Tpm4	28.51	372.3383	7
TRAM1_RAT	Q5XI41	Translocating chain-associated membrane protein 1	Tram1	43.03	493.56726	3
TRPV2_RAT	Q9WUD2	Transient receptor potential cation channel subfamily V member 2 (TRPV2)	Trpv2	86.71	2500.0945	16
TRY1_RAT	P00762	Anionic trypsin-1	Prss1	25.96	2475.5454	1
TXTP_RAT	P32089	Citrate transport protein	Slc25a1	33.84	951.4356	7
UBXN4_RAT	Q5HZY0	Erasin	Ubxn4	56.39	1303.3049	8
UCRI_RAT	P20788	Cytochrome b-c1 complex subunit Rieske	Uqcrfs1	29.45	3786.0586	7
UFL1_RAT	B2GV24	E3 UFM1-protein ligase 1	Ufl1	89.59	2064.419	22
VAMP3_RAT	P63025	VAMP3	Vamp3	11.48	23798.4	6
VAMP5_RAT	Q9Z2J5	VAMP5	Vamp5	11.50	7016.9023	2
VAMP7_RAT	Q9JHW5	VAMP7	Vamp7	24.78	1283.94	6
VAPA_RAT	Q9Z270	VAMP-associated protein A	Vapa	27.84	20269	12
VAPB_RAT	Q9Z269	VAMP-associated protein B	Vapb	26.92	19280.703	9
VATB2_RAT	P62815	V-type proton ATPase subunit B	Atp6v1b2	56.55	5370.288	20
VATE1_RAT	Q6PCU2	V-type proton ATPase subunit E 1	Atp6v1e1	26.13	3807.996	7
VATF_RAT	P50408	V-type proton ATPase subunit F	Atp6v1f	13.37	5433.4697	6
VDAC1_RAT	Q9Z2L0	Voltage-dependent anion-selective channel protein 1	Vdac1	30.76	31697.959	16
VDAC2_RAT	P81155	Voltage-dependent anion-selective channel protein 2	Vdac2	31.75	30921.11	15
VDAC3_RAT	Q9R1Z0	Voltage-dependent anion-selective channel protein 3	Vdac3	30.80	21432.629	16
VEZA_RAT	Q5XI52	Vezatin	Vezt	88.69	599.8607	7
VGFR2_RAT	O08775	Vascular endothelial growth factor receptor 2	Kdr	150.39	151.32126	2
VKOR1_RAT	Q6TEK4	Vitamin K epoxide reductase complex subunit 1	Vkorc1	17.78	3224.8171	1
VMAT2_RAT	Q01827	Vesicular amine transporter 2	Slc18a2	55.69	1077.7045	3
VMP1_RAT	Q91ZQ0	Vacuole membrane protein 1	Vmp1	45.90	1842.7786	2
VWA5A_RAT	Q75WE7	Von Willebrand factor A domain-containing protein 5A	Vwa5a	91.48	857.0407	12
YES_RAT	F1LM93	Tyrosine-protein kinase Yes	Yes1	60.61	13521.72	22
YIF1B_RAT	Q6PEC3	Protein YIF1B	Yif1b	28.42	2773.6765	2
YIPF4_RAT	Q5M7T4	Protein YIPF4	Yipf4	27.28	1942.5706	2
YIPF5_RAT	Q5XID0	Protein YIPF5	Yipf5	27.90	1374.8135	2
YMEL1_RAT	Q925S8	ATP-dependent zinc metalloprotease YME1L1	Yme1l1	79.87	1352.321	18
ZNT1_RAT	Q62720	Zinc transporter 1	Slc30a1	55.14	290.3024	4
ZW10_RAT	Q4V8C2	Centromere/kinetochore protein zw10 homolog	Zw10	87.97	548.4421	16

Header line description of worksheets	
**Method**	Protein identification with which Method
**Protein ID**	Protein identification of UniProt
**Protein accession**	Protein accession number of UniProt
**Protein description**	Protein functional description
**Gene name**	Indicates the name of the gene that code for the protein sequence
**MW [kDa]**	Protein molecular weight ,unit [kDa]
**Score AVG**	A simple rule to be used to judge whether a result is significant or not
**Peptides AVG**	Number of peptides whitch spectrum hit
