# Supplementary material for: Proteomic Analysis of Lipid Rafts from RBL-2H3 Mast Cells
Source: Int J Mol Sci. 2019 Aug 11;20(16):3904. doi: 10.3390/ijms20163904 (PMC6720779; doi:10.3390/ijms20163904)
Supplement: Supplementary file 1 [file ijms-20-03904-s001.zip › Table S3.pdf]

Supplementary Table 3. Detailed annotation of proteins identified in Methods I and II

Method	Protein ID	Protein accession	Protein description	Gene name
Met I; Met II	1433B_RAT	P35213	14-3-3 protein beta/alpha	Ywhab
Met I; Met II	1433E_RAT	P62260	14-3-3 protein epsilon	Ywhae
Met I; Met II	1433F_RAT	P68511	14-3-3 protein eta	Ywhah
Met I; Met II	1433G_RAT	P61983	14-3-3 protein gamma	Ywhag
Met I; Met II	1433T_RAT	P68255	14-3-3 protein theta	Ywhaq
Met I; Met II	1433Z_RAT	P63102	14-3-3 protein zeta/delta	Ywhaz
Met I; Met II	4F2_RAT	Q794F9	4F2 cell-surface antigen heavy chain	Slc3a2
Met I	6PGD_RAT	P85968	6-phosphogluconate dehydrogenase decarboxylating	Pgd
Met I	6PGL_RAT	P85971	6-phosphogluconolactonase	Pgls
Met II	A0JN29_RAT	A0JN29	Lunapark ER junction formation factor	Lnpk
Met I; Met II	A0JN30_RAT	A0JN30	Canopy 2 homolog	Cnpy2
Met II	A1L1J8_RAT	A1L1J8	Ras-related protein Rab-5b	Rab5b
Met II	A1L1L6_RAT	A1L1L6	Mitochondrial Rho GTPase 1	Rhot1
Met I; Met II	A3RLA8_RAT	A3RLA8	Low affinity immunoglobulin gamma Fc region receptor II	Fcgr2b
Met I	AATC_RAT	P13221	Aspartate aminotransferase cytoplasmic	Got1
Met I; Met II	AATM_RAT	P00507	Aspartate aminotransferase mitochondrial	Got2
Met II	ABCB6_RAT	O70595	ATP-binding cassette sub-family B member 6	Abcb6
Met II	ABCB8_RAT	Q5RKI8	ATP-binding cassette sub-family B member 8	Abcb8
Met I	ABCF1_RAT	Q6MG08	ATP-binding cassette sub-family F member 1	Abcf1
Met II	ABHGA_RAT	Q6MG55	Abhydrolase domain containing 16A	Abhd16a
Met I	ACON_RAT	Q9ER34	Aconitate hydratase mitochondrial	Aco2
Met II	ACSL3_RAT	Q63151	Long chain fatty acid CoA ligase 3	Acsl3
Met II	ACSL5_RAT	O88813	Long chain fatty acid CoA ligase 5	Acsl5
Met I; Met II	ACTB_RAT	P60711	Actin cytoplasmic 1	Actb
Met I	ACTH_RAT	P63269	Actin gamma-enteric smooth muscle	Actg2
Met I	ADA_RAT	Q920P6	Adenosine deaminase	Ada
Met II	ADA15_RAT	Q9QYV0	Disintegrin and metalloproteinase domain-containing protein 15	Adam15
Met I; Met II	ADT1_RAT	Q05962	ADP/ATP translocase 1	Slc25a4
Met I; Met II	ADT2_RAT	Q09073	ADP/ATP translocase 2	Slc25a5
Met II	AIFM1_RAT	Q9JM53	Apoptosis-inducing factor 1	Aifm1
Met I	AK1A1_RAT	P51635	Alcohol dehydrogenase [NADP(+)]	Akr1a1
Met II	AKAP1_RAT	O88884	A-kinase anchor protein 1, mitochondrial	Akap1
Met II	AL5AP_RAT	P20291	Arachidonate 5-lipoxygenase-activating protein	Alox5ap

Met I; Met II	ALDOA_RAT	P05065	Fructose-bisphosphate aldolase A	Aldoa
Met I	ALDR_RAT	P07943	Aldose reductase	Akr1b1
Met I	AN32B_RAT	Q9EST6	Acidic leucine-rich nuclear phosphoprotein 32 family member B	Anp32b
Met I	AN32E_RAT	Q5XIE0	Acidic leucine-rich nuclear phosphoprotein 32 family member E	Anp32e
Met I; Met II	ANXA1_RAT	P07150	Annexin A1	Anxa1
Met I	ANXA2_RAT	Q07936	Annexin A2	Anxa2
Met I	ANXA5_RAT	P14668	Annexin A5	Anxa5
Met II	APAF_RAT	Q9EPV5	Apoptotic protease-activating factor 1	Apaf1
Met I; Met II	ARL8B_RAT	Q66HA6	ADP-ribosylation factor-like protein 8B	Arl8b
Met II	ARM10_RAT	B1WBW4	Armadillo repeat-containing protein 10	Armc10
Met II	ARMX3_RAT	Q5XID7	Armadillo repeat-containing X-linked protein 3	Armcx3
Met I	ASNS_RAT	P49088	Glutamine-dependent asparagine synthetase	Asns
Met I; Met II	AT1A1_RAT	P06685	Sodium/potassium-transporting ATPase subunit alpha-1	Atp1a1
Met I	AT1A2_RAT	P06686	Sodium/potassium-transporting ATPase subunit alpha-2	Atp1a2
Met I	AT1A3_RAT	P06687	Sodium/potassium-transporting ATPase subunit alpha-3	Atp1a3
Met I; Met II	AT1A4_RAT	Q64541	Sodium/potassium-transporting ATPase subunit alpha-4	Atp1a4
Met I; Met II	AT1B3_RAT	Q63377	Sodium/potassium-transporting ATPase subunit beta-3	Atp1b3
Met II	AT2A1_RAT	Q64578	Sarcoplasmic/endoplasmic reticulum calcium ATPase 1 (SERCA1)	Atp2a1
Met II	AT5F1_RAT	P19511	ATP synthase F(0) complex subunit B1 mitochondrial	Atp5pb
Met II	ATAD1_RAT	Q505J9	ATPase family AAA domain-containing protein 1	Atad1
Met II	ATAD3_RAT	Q3KRE0	ATPase family AAA domain-containing protein 3	Atad3
Met II	ATP4A_RAT	P09626	Potassium-transporting ATPase alpha chain 1	Atp4a
Met I; Met II	ATP5H_RAT	P31399	ATP synthase subunit D mitochondrial	Atp5pd
Met I; Met II	ATP5I_RAT	P29419	ATP synthase subunit E mitochondrial	Atp5me
Met I; Met II	ATP5J_RAT	P21571	ATP synthase-coupling factor 6 mitochondrial	Atp5pf
Met I; Met II	ATPA_RAT	P15999	ATP synthase subunit alpha mitochondrial	Atp5f1a
Met I	ATPB_RAT	P10719	ATP synthase subunit beta mitochondrial	Atp5f1b
Met II	ATPG_RAT	P35435	ATP synthase subunit gamma mitochondrial	Atp5f1c
Met II	ATPK_RAT	D3ZAF6	ATP synthase subunit F mitochondrial	Atp5mf
Met I; Met II	ATPO_RAT	Q06647	ATP synthase subunit O mitochondrial	Atp5po
Met II	ATRAP_RAT	Q642A2	Type-1 angiotensin II receptor-associated protein	Agtrap
Met II	AUP1_RAT	A1L134	Ancient ubiquitous protein 1	Aup1
Met II	B0BN02_RAT	B0BN02	Metaxin 1	Mtx1
Met II	B0BN30_RAT	B0BN30	Mitochondrial carrier homolog 1	Mtch1
Met II	B0BN52_RAT	B0BN52	Mitochondrial carrier homolog 2	Mtch2
Met I; Met II	B0BN81_RAT	B0BN81	Ribosomal protein S5	Rps5
Met I; Met II	B0BNG3_RAT	B0BNG3	Vesicular integral-membrane protein VIP36	Lman2
Met I	B0BNJ1_RAT	B0BNJ1	Sorcin	Sri

Met II	B0BNK1_RAT	B0BNK1	Ras-related protein Rab-5c	Rab5c
Met I	B0K010_RAT	B0K010	Thioredoxin domain-containing 17	Txndc17
Met I	B0K031_RAT	B0K031	60S ribosomal protein L7, isoform	Rpl7
Met II	B1WBY5_RAT	B1WBY5	DnaJ homolog subfamily C member 11	Dnajc11
Met II	B1WBY7_RAT	B1WBY7	Erlin-1	Erlin1
Met II	B1WC50_RAT	B1WC50	RNA-binding protein EWS-like	Ewsr1
Met II	B1WC61_RAT	B1WC61	Acyl-CoA dehydrogenase family member 9	Acad9
Met II	B1WC67_RAT	B1WC67	Solute carrier family 25 member 24	Slc25a24
Met I; Met II	B2GUV5_RAT	B2GUV5	V-type proton ATPase subunit G 1	Atp6v1g1
Met II	B2GUX5_RAT	B2GUX5	5'-nucleotidase	Nt5c3a
Met I; Met II	B2GUZ9_RAT	B2GUZ9	Fam49b protein	Fam49b
Met I	B2GV72_RAT	B2GV72	Carbonyl reductase 3	Cbr3
Met I; Met II	B2GV96_RAT	B2GV96	Coiled-coil domain containing 115	Ccdc115
Met I	B2GVB9_RAT	B2GVB9	Fermt3 protein	Fermt3
Met I; Met II	B2MG_RAT	P07151	Beta-2-microglobulin	B2m
Met I; Met II	B2RYC9_RAT	B2RYC9	Glucosylceramidase	Gba
Met II	B2RYD7_RAT	B2RYD7	STT3B, catalytic subunit of the oligosaccharyltransferase complex	Stt3b
Met II	B2RYF6_RAT	B2RYF6	Cleft lip and palate associated transmembrane protein 1	Clptm1
Met II	B2RYQ2_RAT	B2RYQ2	Serine/threonine-protein phosphatase 2A activator	Ptpa
Met I	B2RZ27_RAT	B2RZ27	SH3 domain-binding glutamic acid-rich-like protein 3	Sh3bgrl3
Met II	B2RZB3_RAT	B2RZB3	Eva-1 homolog B	Eva1b
Met I; Met II	B2RZD1_RAT	B2RZD1	Protein transport protein Sec61 subunit beta	Sec61b
Met II	B3A2_RAT	P23347	Anion exchange protein 2	Slc4a2
Met II	B4F7C2_RAT	B4F7C2	Tubulin beta-4A	Tubb4a
Met II	B4F7C9_RAT	B4F7C9	STT3A, catalytic subunit of the oligosaccharyltransferase complex	Stt3a
Met II	B4GN1_RAT	Q10468	Beta-1,4 N-acetylgalactosaminyltransferase 1	B4galnt1
Met II	B5DEL8_RAT	B5DEL8	NADH dehydrogenase (Ubiquinone) iron-sulfur protein 5	Ndufs5
Met I	B5DEN5_RAT	B5DEN5	Eukaryotic translation elongation factor 1 beta 2	Eef1b2
Met II	B5DEZ3_RAT	B5DEZ3	Coiled-coil domain containing 126	Ccdc126
Met II	B5DFJ2_RAT	B5DFJ2	Protein FAM3A	Fam3a
Met II	B6RK61_RAT	B6RK61	Myosin heavy chain 7B	Myh7b
Met II	BAF_RAT	Q9R1T1	Barrier-to-autointegration factor	Banf1
Met I	BAG6_RAT	Q6MG49	Large proline-rich protein BAG6	Bag6
Met I; Met II	BASI_RAT	P26453	Basigin	Bsg
Met I; Met II	BASP1_RAT	Q05175	Brain acid soluble protein 1	Basp1
Met II	BET1_RAT	Q62896	Bet1 golgi vesicular membrane trafficking protein	Bet1
Met II	BET1L_RAT	O35152	BET1-like protein	Bet1l
Met II	C1GLC_RAT	Q499P3	C1GALT1-specific chaperone 1	C1galt1c1

Met II	C1GLT_RAT	Q9JJ05	Glycoprotein-N-acetylgalactosamine 3-beta-galactosyltransferase 1	C1galt1
Met I; Met II	C1QBP_RAT	O35796	Complement component 1 Q subcomponent-binding protein mitochondrial	C1qbp
Met II	CADM3_RAT	Q1WIM3	Cell adhesion molecule 3	Cadm3
Met I	CAH2_RAT	P27139	Carbonic anhydrase 2	Ca2
Met II	CAH8_RAT	Q5PPN4	Carbonic anhydrase-related protein	Ca8
Met I	CALM_RAT	P0DP29	Calmodulin-1	Calm1
Met I; Met II	CALR_RAT	P18418	Calreticulin	Calr
Met I; Met II	CALX_RAT	P35565	Calnexin	Canx
Met I	CAP1_RAT	Q08163	Adenylyl cyclase-associated protein 1	Cap1
Met I	CAPG_RAT	Q6AYC4	Macrophage-capping protein	Capg
Met I	CAPZB_RAT	Q5XI32	F-actin-capping protein subunit beta	Capzb
Met I	CAZA2_RAT	Q3T1K5	F-actin-capping protein subunit alpha-2	Capza2
Met II	CBPA3_RAT	P21961	Mast cell carboxypeptidase A	Cpa3
Met II	CCD47_RAT	Q5U2X6	Coiled-coil domain-containing protein 47	Ccdc47
Met II	CD38_RAT	Q64244	ADP-ribosyl cyclase/cyclic ADP-ribose hydrolase 1	Cd38
Met I; Met II	CD48_RAT	P10252	CD48 antigen	Cd48
Met I	CDC37_RAT	Q63692	Hsp90 co-chaperone Cdc37	Cdc37
Met I; Met II	CDC42_RAT	Q8CFN2	Cell division control protein 42 homolog (CDC42)	Cdc42
Met I	CDV3_RAT	Q5XIM5	Protein CDV3 homolog	Cdv3
Met I; Met II	CH10_RAT	P26772	10 kDa heat shock protein mitochondrial	Hspe1
Met II	CH60_RAT	P63039	60 kDa heat shock protein	Hspd1
Met II	CHPT1_RAT	Q66H21	Cholinephosphotransferase 1	Chpt1
Met II	CISD1_RAT	B0K020	CDGSH iron-sulfur domain-containing protein 1	Cisd1
Met I; Met II	CISY_RAT	Q8VHF5	Citrate synthase mitochondrial	Cs
Met II	CK5P3_RAT	Q9JLH7	CDK5 regulatory subunit-associated protein 3	Cdk5rap3
Met II	CL2DB_RAT	Q0H8B9	C-type lectin domain family 2 member D11	Clec2d11
Met II	CLCN5_RAT	P51796	Chloride intracellular channel protein 5	Clcn5
Met I; Met II	CLH1_RAT	P11442	Clathrin heavy chain 1	Cltc
Met I	CLIC1_RAT	Q6MG61	Chloride intracellular channel protein 1	Clic1
Met II	CMA1_RAT	P50339	Chymase 1	Cma1
Met I	CNBP_RAT	P62634	Cellular nucleic acid-binding protein	Cnbp
Met I; Met II	CNTN1_RAT	Q63198	Contactin 1	Cntn1
Met I; Met II	COF1_RAT	P45592	Cofilin-1	Cfl1
Met II	COMT_RAT	P22734	Catechol O-methyltransferase	Comt
Met II	COPB_RAT	P23514	Coatomer subunit beta 1	Copb1
Met II	COPB2_RAT	O35142	Coatomer subunit beta 2	Copb2
Met II	COPD_RAT	Q66H80	Coatomer subunit delta	Arcn1
Met II	COPG1_RAT	Q4AEF8	Coatomer subunit gamma 1	Copg1

Met I	COR1A_RAT	Q91ZN1	Coronin-1A	Coro1a
Met I	COTL1_RAT	B0BNA5	Coactosin-like protein	Cotl1
Met I; Met II	COX41_RAT	P10888	Cytochrome c oxidase subunit 4 isoform 1 mitochondrial	Cox4i1
Met I; Met II	COX5A_RAT	P11240	Cytochrome c oxidase subunit 5A mitochondrial	Cox5a
Met I; Met II	COX5B_RAT	P12075	Cytochrome c oxidase subunit 5B mitochondrial	Cox5b
Met I; Met II	CP20A_RAT	Q6P7D4	Cytochrome P450 20A1	Cyp20a1
Met II	CP51A_RAT	Q64654	Lanosterol 14-alpha demethylase	Cyp51a1
Met II	CR1L_RAT	Q63135	Complement component receptor 1-like protein	Cr1l
Met I; Met II	CX6C2_RAT	P11951	Cytochrome c oxidase subunit 6C2	Cox6c2
Met II	CX7A2_RAT	P35171	Cytochrome c oxidase subunit 7A2	Cox7a2
Met II	CYB5B_RAT	P04166	Cytochrome b5 type B	Cyb5b
Met I	CYBP_RAT	Q6AYK6	Calcyclin-binding protein	Cacybp
Met I	CYC_RAT	P62898	Cytochrome c somatic	Cycs
Met II	D3Z9L0_RAT	D3Z9L0	Acylglycerol kinase	Agk
Met II	D3ZAS9_RAT	D3ZAS9	DDRGK domain containing 1	Ddrgk1
Met II	D3ZAY7_RAT	D3ZAY7	Band 4.1-like protein 2	Epb41l2
Met II	D3ZCZ9_RAT	D3ZCZ9	NADH dehydrogenase [ubiquinone] iron-sulfur protein 6	LOC100912599
Met I; Met II	D3ZD09_RAT	D3ZD09	Cytochrome c oxidase subunit 6B1	Cox6b1
Met II	D3ZD11_RAT	D3ZD11	Signal peptidase complex subunit 2	Spcs2
Met II	D3ZD83_RAT	D3ZD83	Major facilitator superfamily domain-containing protein 10	Mfsd10
Met II	D3ZDR2_RAT	D3ZDR2	Charged multivesicular body protein 6	Chmp6
Met II	D3ZF11_RAT	D3ZF11	Ragulator complex protein LAMTOR5	Lamtor5
Met II	D3ZF12_RAT	D3ZF12	Signal peptidase complex subunit 3	Spcs3
Met II	D3ZF54_RAT	D3ZF54	Anoctamin-10	Ano10
Met II	D3ZF97_RAT	D3ZF97	Endoplasmic reticulum lectin 1	Erlec1
Met II	D3ZFK5_RAT	D3ZFK5	Signal peptidase complex subunit 1	Spcs1
Met II	D3ZG43_RAT	D3ZG43	NADH dehydrogenase (ubiquinone) iron-sulfur protein 3	Ndufs3
Met I; Met II	D3ZGM7_RAT	D3ZGM7	Peptidase inhibitor 16	Pi16
Met II	D3ZGV2_RAT	D3ZGV2	Peroxisomal membrane protein PEX16	Pex16
Met II	D3ZHA7_RAT	D3ZHA7	Myosin light chain 6B	RGD1560334_predicted
Met II	D3ZIE9_RAT	D3ZIE9	Delta-1-pyrroline-5-carboxylate synthase	Aldh18a1
Met II	D3ZIJ5_RAT	D3ZIJ5	Dolichyl-phosphate mannosyltransferase subunit 3	Dpm3
Met II	D3ZIP8_RAT	D3ZIP8	Endonuclease domain containing 1	---
Met II	D3ZJ32_RAT	D3ZJ32	Extended synaptotagmin 2	Esyt2
Met II	D3ZJS3_RAT	D3ZJS3	Translocase of outer mitochondrial membrane 6	Tomm6
Met II	D3ZJX5_RAT	D3ZJX5	Mitochondrial import inner membrane translocase subunit TIM50	Timm50
Met II	D3ZK56_RAT	D3ZK56	Ras-related protein Rap-2c	Rap2c
Met II	D3ZKE6_RAT	D3ZKE6	Sarcolemmal membrane-associated protein	Slmap

Met II	D3ZL85_RAT	D3ZL85	Cytochrome c heme lyase	Hccs
Met II	D3ZLC4_RAT	D3ZLC4	Slc2a6 protein	Slc2a6
Met II	D3ZLT7_RAT	D3ZLT7	Uncharacterized protein KIAA2013	RGD1305350
Met II	D3ZM19_RAT	D3ZM19	Protein EVI2B	Evi2b
Met II	D3ZMM8_RAT	D3ZMM8	Solute carrier family 7 member 6	Slc7a6
Met II	D3ZMR1_RAT	D3ZMR1	Translocase of outer mitochondrial membrane 7	Tomm7
Met I; Met II	D3ZN21_RAT	D3ZN21	ATP-dependent RNA helicase DDX3Y	Ddx3y
Met II	D3ZNG3_RAT	D3ZNG3	Signal peptide peptidase-like 2A	Sppl2a
Met I	D3ZNQ6_RAT	D3ZNQ6	NEDD8-conjugating enzyme Ubc12	Ube2m
Met II	D3ZNS8_RAT	D3ZNS8	Aladin	Aaas
Met II	D3ZP13_RAT	D3ZP13	Sulfhydryl oxidase 2	Qsox2
Met II	D3ZP98_RAT	D3ZP98	Minor histocompatibility antigen H13	Hm13
Met I	D3ZPP2_RAT	D3ZPP2	ADP-ribosylation factor-like protein 8A	Arl8a
Met I; Met II	D3ZPR0_RAT	D3ZPR0	Exportin-2	Cse1l
Met II	D3ZQG9_RAT	D3ZQG9	RT1 class I locus CE4	RT1-CE4
Met I; Met II	D3ZRN3_RAT	D3ZRN3	Beta-actin-like protein 2	Actbl2
Met I; Met II	D3ZSA9_RAT	D3ZSA9	Nodal modulator 1	Nomo1
Met II	D3ZSY5_RAT	D3ZSY5	keratin 84	Krt84
Met II	D3ZTJ0_RAT	D3ZTJ0	Protein XRP2	Rp2
Met II	D3ZU83_RAT	D3ZU83	Endoplasmic reticulum-Golgi intermediate compartment 3	Ergic3
Met II	D3ZUD8_RAT	D3ZUD8	Transmembrane 9 superfamily member 3	Tm9sf3
Met II	D3ZUQ7_RAT	D3ZUQ7	Pantothenate kinase 3	Pank3
Met II	D3ZUX5_RAT	D3ZUX5	MICOS complex subunit MIC-19	Chchd3
Met II	D3ZUZ4_RAT	D3ZUZ4	Glycosaminoglycan xylosylkinase	Fam20b
Met I; Met II	D3ZV75_RAT	D3ZV75	Major facilitator superfamily domain-containing protein 1	Mfsd1
Met II	D3ZWS0_RAT	D3ZWS0	Protein scribble homolog	Scrib
Met II	D3ZWZ1_RAT	D3ZWZ1	Integrin alpha X	Itgax
Met II	D3ZXF9_RAT	D3ZXF9	39S ribosomal protein L12, mitochondrial	Mrpl12
Met II	D3ZYM4_RAT	D3ZYM4	Macrophage stimulating 1 receptor	Mst1r
Met I	D3ZYS7_RAT	D3ZYS7	Ras GTPase-activating protein 1	G3bp1
Met I; Met II	D3ZYW2_RAT	Q499R8	Heterogeneous nuclear ribonucleoprotein H	Hnrnph1
Met I; Met II	D3ZZR9_RAT	D3ZZR9	Peptidylprolyl isomerase	Fkbp2
Met II	D3ZZS8_RAT	D3ZZS8	V-type proton ATPase subunit B, kidney isoform	Atp6v1b1
Met II	D4A017_RAT	D4A017	Transmembrane protein 87 A	Tmem87a
Met II	D4A0E2_RAT	D4A0E2	NSF attachment protein gamma	Napg
Met II	D4A0G7_RAT	D4A0G7	Ras-related protein Rab-37	Rab37
Met II	D4A0T0_RAT	D4A0T0	NADH dehydrogenase 1 beta subcomplex subunit 10	Ndufb10
Met II	D4A0T8_RAT	D4A0T8	Dehydrogenase/reductase SDR family member 7	Dhrs7

Met II	D4A0W1_RAT	D4A0W1	ER membrane protein complex subunit 4	Emc4
Met I	D4A0Y9_RAT	D4A0Y9	Phosphatidylcholine translocator ABCB4	Abcb1b
Met I; Met II	D4A133_RAT	D4A133	V-type proton ATPase catalytic subunit A	Atp6v1a
Met I; Met II	D4A1D8_RAT	D4A1D8	Surfeit 4	Surf4
Met II	D4A2H2_RAT	D4A2H2	Serine palmitoyltransferase 1	Sptlc1
Met II	D4A2Z6_RAT	D4A2Z6	Translocation protein SEC63 homolog	Sec63
Met II	D4A376_RAT	D4A376	Ras-related protein Rab-12	Rab12
Met I	D4A3S8_RAT	D4A3S8	NOL1/NOP2/Sun domain family member 2	Nsun2
Met II	D4A416_RAT	D4A416	Cleft lip and palate transmembrane protein 1-like protein	Clptm1l
Met II	D4A465_RAT	D4A465	Ragulator complex protein LAMTOR2	Lamtor2
Met II	D4A4J3_RAT	D4A4J3	Alpha-mannosidase 2x	Man2a2
Met II	D4A4T0_RAT	D4A4T0	STIP1 homology and U box-containing protein 1	Stub1
Met II	D4A533_RAT	D4A533	Transmembrane anterior posterior transformation protein 1	Tapt1
Met II	D4A5K6_RAT	D4A5K6	Zinc metallopeptidase STE24	Zmpste24
Met I	D4A6A2_RAT	D4A6A2	Heterogeneous nuclear ribonucleoprotein A3-like	LOC100911361
Met I	D4A720_RAT	D4A720	Serine/arginine-rich splicing factor 7 isoform 2	Srsf7
Met II	D4A781_RAT	D4A781	Importin 5	Ipo5
Met I	D4A7U1_RAT	D4A7U1	Zyxin	Zyx
Met I	D4A7U6_RAT	D4A7U6	U6 snRNA-associated Sm-like protein LSm3	Lsm3
Met II	D4A7Y9_RAT	D4A7Y9	Essential MCU regulator, mitochondrial	Smdt1
Met II	D4A994_RAT	D4A994	ER membrane protein complex subunit 1	Emc1
Met I; Met II	D4A9L2_RAT	D4A9L2	Serine/arginine-rich splicing factor 1	Srsf1
Met I; Met II	D4A9X7_RAT	D4A9X7	Hypermethylated in cancer 2 protein	Hic2
Met II	D4AA91_RAT	D4AA91	Ceramide synthase 5	Cers5
Met I; Met II	D4AAE9_RAT	D4AAE9	CDGSH iron-sulfur domain-containing protein 2	Cisd2
Met II	D4AAL4_RAT	D4AAL4	Adhesion G protein-coupled receptor L3	Adgrl3
Met II	D4ABI7_RAT	D4ABI7	Very-long-chain (3R)-3-hydroxyacyl-CoA dehydratase	Hacd3
Met II	D4ABM3_RAT	D4ABM3	Dishevelled associated activator of morphogenesis 1	Daam1
Met II	D4ABY2_RAT	D4ABY2	Coatomer subunit gamma 2	Copg2
Met II	D4ACG2_RAT	D4ACG2	Acetolactate synthase-like protein	Ilvbl
Met I	D4ADD7_RAT	D4ADD7	Glutaredoxin 5	Glrx5
Met II	D4ADE8_RAT	D4ADE8	ATP-dependent RNA helicase DDX3X	Ddx3x
Met I; Met II	D4ADS9_RAT	D4ADS9	EFR3 homolog A	Efr3a
Met II	D4ADV4_RAT	Q80ZA5	Sodium-driven chloride bicarbonate exchanger	Slc4a10
Met I; Met II	D4AEG2_RAT	D4AEG2	Ras-related protein Rab-32	Rab32
Met I	D4AEP0_RAT	D4AEP0	Adenylosuccinate synthetase isozyme 2	Adss
Met II	DAD1_RAT	P61805	Dolichyl-diphosphooligosaccharide protein glycosyltransferase subunit DAD1	Dad1
Met II	DHC24_RAT	Q5BQE6	Delta(24)-sterol reductase	Dhcr24

Met II	DHCR7_RAT	Q9Z2Z8	7-dehydrocholesterol reductase	Dhcr7
Met II	DHE3_RAT	P10860	Glutamate dehydrogenase 1	Glud1
Met II	DHPR_RAT	P11348	Dihydropteridine reductase	Qdpr
Met I; Met II	DHSA_RAT	Q920L2	Succinate dehydrogenase [ubiquinone] flavoprotein subunit mitochondrial	Sdha
Met II	DHSB_RAT	P21913	Succinate dehydrogenase [ubiquinone] iron-sulfur subunit mitochondrial	Sdhb
Met I	DOPD_RAT	P80254	D-dopachrome decarboxylase	Ddt
Met I	DUT_RAT	P70583	Deoxyuridine 5'-triphosphate nucleotidohydrolase	Dut
Met I	DX39B_RAT	Q63413	Spliceosome RNA helicase Ddx39b	Ddx39b
Met II	E2AK3_RAT	Q9Z1Z1	Eukaryotic translation initiation factor 2-alpha kinase 3	Eif2ak3
Met I	E9PSV5_RAT	E9PSV5	Phosphoserine aminotransferase	Psat1
Met II	E9PT10_RAT	Q861Q1	MHC class I antigen	RT1-CE4
Met I; Met II	E9PT29_RAT	E9PT29	ATP-dependent RNA helicase DDX17	Ddx17
Met I	E9PT65_RAT	E9PT65	Radixin	Rdx
Met II	E9PTI1_RAT	E9PTI1	V-type proton ATPase subunit H	Atp6v1h
Met II	E9PTU4_RAT	E9PTU4	Myosin heavy chain 11	Myh11
Met I; Met II	E9PTW1_RAT	E9PTW1	Secretory carrier-associated membrane protein	Scamp3
Met I; Met II	E9PTX9_RAT	E9PTX9	Solute carrier family 12 member 2	Slc12a2
Met II	E9PU37_RAT	E9PU37	Palmitoyltransferase	Zdhhc13
Met II	E9PU77_RAT	E9PU77	Synaptotagmin-like protein 3	Sytl3
Met I	E9PU78_RAT	E9PU78	Isoform Bcl-X(S) of Bcl-2-like protein 1	---
Met II	ECHA_RAT	Q64428	Trifunctional enzyme subunit alpha	Hadha
Met I	EF1A1_RAT	P62630	Elongation factor 1-alpha 1	Eef1a1
Met I; Met II	EF1G_RAT	Q68FR6	Elongation factor 1-gamma	Eef1g
Met I; Met II	EF2_RAT	P05197	Elongation factor 2	Eef2
Met II	EMC2_RAT	B0BNG0	ER membrane protein complex subunit 2	Emc2
Met II	EMC3_RAT	Q5U2V8	ER membrane protein complex subunit 3	Emc3
Met II	EMC8_RAT	Q5FVL2	ER membrane protein complex subunit 8	Emc8
Met I; Met II	ENOA_RAT	P04764	Alpha-enolase	Eno1
Met I	ENOB_RAT	P15429	Beta-enolase	Eno3
Met I; Met II	ENPL_RAT	Q66HD0	Endoplasmin	Hsp90b1
Met II	ENPP3_RAT	P97675	Ectonucleotide pyrophosphatase/phosphodiesterase family member 3	Enpp3
Met II	ERLN2_RAT	B5DEH2	Erlin-2	Erlin2
Met I	ERO1A_RAT	Q8R4A1	ERO1-like protein alpha	Ero1a
Met I	ERP29_RAT	P52555	Endoplasmic reticulum resident protein 29	Erp29
Met II	ESYT1_RAT	Q9Z1X1	Extended synaptotagmin 1	Esyt1
Met I	EZRI_RAT	P31977	Ezrin	Ezr
Met I	F10A1_RAT	P50503	Hsc70-interacting protein	St13
Met I	F1LM33_RAT	F1LM33	Leucine-rich PPR motif-containing protein mitochondrial isoform	Lrpprc

Met I; Met II	F1LM66_RAT	F1LM66	116 kDa U5 small nuclear ribonucleoprotein component	Eftud2
Met I	F1LM82_RAT	F1LM82	Heterogeneous nuclear ribonucleoproteins A2/B1	Hnrnpa2b1
Met II	F1LMG7_RAT	F1LMG7	Leucine-rich repeat-containing protein 7	Lrrc7
Met II	F1LMK0_RAT	F1LMK0	A-kinase anchoring protein 1 isoform 2	Akap1
Met I; Met II	F1LMM5_RAT	F1LMM5	Arachidonate 5-lipoxygenase	Alox5
Met I	F1LMP7_RAT	F1LMP7	Granulins	Grn
Met II	F1LN88_RAT	F1LN88	Aldehyde dehydrogenase	Aldh2
Met II	F1LNC4_RAT	F1LNC4	Vesicle transport through interaction with t-SNAREs homolog 1B	Vti1b
Met II	F1LNF0_RAT	F1LNF0	Myosin heavy chain 14	Myh14
Met II	F1LNT8_RAT	F1LNT8	VAMP8	Vamp8
Met II	F1LNX2_RAT	F1LNX2	Calcium release-activated calcium channel protein 1 (Orai1)	Orai1
Met II	F1LP82_RAT	F1LP82	Ras-related protein Rab-2a	Rab2a
Met II	F1LPK9_RAT	F1LPK9	Phosphatidylinositol glycan anchor biosynthesis class U protein	Pigu
Met I	F1LPV0_RAT	F1LPV0	Asparagine--tRNA ligase, cytoplasmic	Nars
Met I; Met II	F1LQ81_RAT	F1LQ81	Vesicle-fusing ATPase	Nsf
Met II	F1LQ92_RAT	F1LQ92	Sel-1 homolog 1	Sel1l
Met I	F1LQJ7_RAT	F1LQJ7	Phosphoenolpyruvate carboxykinase [GTP], mitochondrial	Pck2
Met II	F1LQR8_RAT	F1LQR8	Inositol 1,4,5-trisphosphate receptor type 2	Itpr2
Met I; Met II	F1LRK1_RAT	F1LRK1	Sodium/potassium-transporting ATPase subunit alpha	Atp4a
Met II	F1LRV9_RAT	F1LRV9	Myosin heavy chain 1	Myh1
Met II	F1LSI5_RAT	F1LSI5	Ras GTPase-activating protein 3	Rasa3
Met I	F1LSN4_RAT	F1LSN4	Fibroblast growth factor receptor	Fgfr3
Met II	F1LSV4_RAT	Q3B7D2	Serine palmitoyltransferase 2	Sptlc2
Met II	F1LT70_RAT	F1LT70	Pantothenate kinase 2	Pank2
Met II	F1LU48_RAT	F1LU48	Endoplasmic reticulum-golgi intermediate compartment 1	Ergic1
Met II	F1LVC3_RAT	F1LVC3	Ras-related protein Rab-6b	Rab6b
Met I	F1LVV4_RAT	F1LVV4	Protein RCC2	Rcc2
Met II	F1LW77_RAT	F1LW77	Ras-related protein Rab-33b	Rab33b
Met II	F1LX07_RAT	F1LX07	Solute carrier family 25 member 12	Slc25a12
Met I; Met II	F1LZW6_RAT	F1LZW6	Solute carrier family 25 member 13	Slc25a13
Met II	F1LZX9_RAT	F1LZX9	Integrin alpha 5	Itgav
Met I; Met II	F1M0G0_RAT	F1M0G0	Regulator of G-protein signaling 17	Rgs17
Met I; Met II	F1M0J3_RAT	F1M0J3	Sodium bicarbonate cotransporter 3, isophorm 4	Slc4a7
Met II	F1M1U0_RAT	F1M1U0	Matrix remodeling associated 7	Mxra7
Met I	F1M2K3_RAT	F1M2K3	Small ubiquitin-related modifier	Sumo2
Met I	F1M2P8_RAT	F1M2P8	Protein kinase C alpha	Prkca
Met II	F1M391_RAT	F1M391	Stimulator of interferon genes protein	Tmem173
Met I	F1M4T3_RAT	B2RYQ5	Enhancer of rudimentary homolog	Erh

Met II	F1M5Z5_RAT	F1M5Z5	Anoctamin-6	Ano6
Met II	F1M6W2_RAT	F1M6W2	Endoplasmic reticulum metallopeptidase 1	Ermp1
Met II	F1M779_RAT	F1M779	Clathrin heavy chain	Cltc
Met II	F1M790_RAT	F1M790	Prostaglandin F2 receptor negative regulator	Ptgfrn
Met I; Met II	F1M7T6_RAT	F1M7T6	Translocon-associated protein subunit gamma	Ssr3
Met II	F1M8F6_RAT	F1M8F6	Myosin heavy chain 8	Myh8
Met I	F1M8X9_RAT	F1M8X9	Golgi brefeldin A-resistant guanine nucleotide exchange factor 1	Gbf1
Met II	F1MA50_RAT	F1MA50	transmembrane protein 123	Tmem123
Met II	F1MA89_RAT	F1MA89	Cyclin Y	Ccny
Met II	F1MAS0_RAT	F1MAS0	keratin 32	Krt32
Met II	F6T9E7_RAT	F6T9E7	P-selectin glycoprotein ligand 1	Selplg
Met II	F7EP67_RAT	Q6IN10	Endothelin-converting enzyme 1 Isoform	Ece1
Met I	F7EPH4_RAT	F7EPH4	Inorganic Pyrophosphatase	Ppa1
Met I	F7EWC1_RAT	F7EWC1	Vasodilator-stimulated phosphoprotein	Vasp
Met I; Met II	F7F4S8_RAT	B2RYB8	Integrin beta 2	Itgb2
Met II	F7F744_RAT	Q6S395	Plectin	Plec
Met I; Met II	F7FFV2_RAT	F7FFV2	keratin 5	Krt5
Met II	F7FKI5_RAT	F7FKI5	Pyruvate dehydrogenase component E1 alpha	Pdha1
Met II	F7FNS3_RAT	F7FNS3	Derlin	Derl1
Met I	F7FP91_RAT	Q95II0	MHC class Ib antigen	RT1-CE5
Met II	FACR1_RAT	Q66H50	Fatty acyl-CoA reductase 1	Far1
Met II	FADS2_RAT	Q9Z122	Acyl-CoA 6-desaturase	Fads2
Met II	FAF2_RAT	Q5BK32	FAS-associated factor 2	Faf2
Met II	FAM3C_RAT	Q810F4	Protein FAM3C	Fam3c
Met I; Met II	FCERB_RAT	P13386	High affinity immunoglobulin epsilon receptor subunit beta	Ms4a2
Met II	FCERG_RAT	P20411	High affinity immunoglobulin epsilon receptor subunit gamma	Fcer1g
Met I	FEN1_RAT	Q5XIP6	Flap endonuclease 1	Fen1
Met II	FGOP2_RAT	Q6TA25	FGFR1 oncogene partner 2 homolog	Fgfr1op2
Met I; Met II	FGR_RAT	Q6P6U0	Tyrosine-protein kinase Fgr	Fgr
Met II	FIS1_RAT	P84817	Mitochondrial fission 1 protein	Fis1
Met I	FKB1A_RAT	Q62658	Peptidyl-prolyl cis-trans isomerase FKBP1A	Fkbp1a
Met II	FKBP8_RAT	Q3B7U9	Peptidyl-prolyl cis-trans isomerase FKBP8	Fkbp8
Met II	FLOT1_RAT	Q9Z1E1	Flotillin-1	Flot1
Met II	FLOT2_RAT	Q9Z2S9	Flotillin-2	Flot2
Met I	FPPS_RAT	P05369	Farnesyl pyrophosphate synthase	Fdps
Met II	FRK_RAT	Q62662	Tyrosine-protein kinase FRK	Frk
Met II	FUT8_RAT	Q6EV76	Alpha-(1,6)-fucosyltransferase	Fut8
Met I; Met II	G3P_RAT	P04797	Glyceraldehyde-3-phosphate dehydrogenase	Gapdh

Met II	G3PT_RAT	Q9ESV6	Glyceraldehyde-3-phosphate dehydrogenase testis-specific	Gapdhs
Met II	G3V608_RAT	G3V608	Interleukin-3 receptor subunit beta	Csf2rb
Met II	G3V637_RAT	G3V637	Syntaxin-binding protein 2, isoform	Stxbp2
Met II	G3V667_RAT	G3V667	Integrin alpha 6	Itga6
Met I; Met II	G3V679_RAT	G3V679	Transferrin receptor protein 1	Tfrc
Met I; Met II	G3V6D3_RAT	G3V6D3	ATP synthase subunit beta	Atp5f1b
Met II	G3V6E1_RAT	G3V6E1	Myosin heavy chain 2	Myh2
Met I	G3V6G5_RAT	G3V6G5	F-box-like/WD repeat-containing protein TBL1X	Tbl1x
Met II	G3V6H0_RAT	G3V6H0	Ras-related protein Rab-1b	LOC100363782
Met II	G3V6H5_RAT	G3V6H5	Solute carrier family 25 member 11	Slc25a11
Met II	G3V6N2_RAT	G3V6N2	Transmembrane emp24 domain-containing protein 4	Tmed4
Met I; Met II	G3V6P7_RAT	G3V6P7	Myosin heavy chain 9	Myh9
Met I; Met II	G3V6P8_RAT	G3V6P8	Guanine nucleotide-binding protein gamma-12	Gng12
Met II	G3V6R4_RAT	G3V6R4	Leucine-rich repeat-containing protein 1	Lrrc1
Met II	G3V6T1_RAT	G3V6T1	Coatomer subunit alpha	Copa
Met I	G3V6T7_RAT	G3V6T7	Protein disulfide-isomerase A4	Pdia4
Met I; Met II	G3V6W2_RAT	G3V6W2	Prolactin regulatory element-binding protein	Preb
Met II	G3V712_RAT	G3V712	keratin 7	Krt7
Met I; Met II	G3V741_RAT	G3V741	Solute carrier family 25 member 3	Slc25a3
Met II	G3V784_RAT	G3V784	ADP-dependent glucokinase	Adpgk
Met II	G3V7A5_RAT	G3V7A5	Low density lipoprotein receptor isoform	Ldlr
Met II	G3V7I0_RAT	G3V7I0	Peroxiredoxin-3	Prdx3
Met II	G3V7I3_RAT	G3V7I3	Manganese-transporting ATPase 13A1	Atp13a1
Met I	G3V7J7_RAT	G3V7J7	Eukaryotic translation initiation factor 5A-2	Eif5a2
Met II	G3V7L8_RAT	G3V7L8	V-type proton ATPase subunit E 1 isoform	Atp6v1e1
Met II	G3V7M2_RAT	G3V7M2	Integrin beta 7	Itgb7
Met II	G3V7N0_RAT	G3V7N0	Heparan sulfate 2-O-sulfotransferase 1	Hs2st1
Met II	G3V7N6_RAT	G3V7N6	Lysophospholipid acyltransferase 7	Mboat7l1
Met I; Met II	G3V7Q7_RAT	G3V7Q7	Ras GTPase-activating-like protein IQGAP1	Iqgap1
Met II	G3V7U6_RAT	G3V7U6	Calpain 5	Capn5
Met II	G3V7Y9_RAT	P28494	Alpha-mannosidase 2	Man2a1
Met I	G3V852_RAT	G3V852	Talin-1	Tln1
Met II	G3V878_RAT	G3V878	Signal peptidase complex catalytic subunit SEC11	Sec11c
Met II	G3V887_RAT	G3V887	V-type proton ATPase subunit A	Tcirg1
Met I	G3V8A5_RAT	G3V8A5	Vacuolar protein sorting-associated protein 35	Vps35
Met I	G3V8C4_RAT	G3V8C4	Chloride intracellular channel protein 4	Clic4
Met II	G3V8F5_RAT	G3V8F5	Mitochondrial import receptor subunit TOM40 homolog	Tomm40
Met I; Met II	G3V8G5_RAT	G3V8G5	Golgi apparatus protein 1	Glg1

Met II	G3V8I4_RAT	G3V8I4	Syntaxin-4A	Stx4
Met II	G3V8L7_RAT	G3V8L7	Integrin alpha M	Itgam
Met II	G3V8Q1_RAT	G3V8Q1	Coatomer subunit epsilon	Cope
Met II	G3V8V4_RAT	G3V8V4	Ceramide synthase 2	Cers2
Met II	G3V8Z1_RAT	G3V8Z1	Linker for activation of T-cells family member 2 (NTAL)	Lat2
Met I; Met II	G3V908_RAT	G3V908	keratin, type II Kb15	Kb15
Met I; Met II	G3V936_RAT	G3V936	Citrate synthase	Cs
Met II	G3V964_RAT	G3V964	Neurotrimin	Ntm
Met I	G3V993_RAT	G3V993	SAM domain, SH3 domain and nuclear localization signals, 1 (SAMNS1)	Samsn1
Met I	G3V9A3_RAT	G3V9A3	14-3-3 protein sigma	Sfn
Met II	G3V9N0_RAT	G3V9N0	Polyadenylate-binding protein 4	Pabpc4
Met II	G3V9N9_RAT	G3V9N9	alpha-1,2-Mannosidase	Man1a1
Met II	G3V9S3_RAT	G3V9S3	Tyrosine-protein phosphatase non-receptor type 13	Ptpn13
Met I; Met II	G3V9U7_RAT	G3V9U7	Sarcoplasmic/endoplasmic reticulum calcium ATPase 3 (SERCA3)	Atp2a3
Met II	G3V9W2_RAT	G3V9W2	Tyrosine-protein kinase JAK1	Jak1
Met II	G3V9X8_RAT	G3V9X8	Magnesium transporter protein 1	Magt1
Met II	G3V9Y2_RAT	G3V9Y2	Phospholipid phosphatase 1	Plpp1
Met II	GALT1_RAT	Q10473	Polypeptide N-acetylgalactosaminyltransferase 1	Galnt1
Met II	GALT7_RAT	Q9R0C5	Polypeptide N-acetylgalactosaminyltransferase 7	Galnt7
Met I; Met II	GBB1_RAT	P54311	Transducin beta-1	Gnb1
Met I; Met II	GBB2_RAT	P54313	Transducin beta-2	Gnb2
Met II	GBB4_RAT	O35353	Transducin beta-4	Gnb4
Met I	GDIA_RAT	P50398	Rab GDP dissociation inhibitor alpha	Gdi1
Met I	GDIB_RAT	P50399	Rab GDP dissociation inhibitor beta	Gdi2
Met I	GDIR1_RAT	Q5XI73	Rho GDP dissociation inhibitor 1	Arhgdia
Met I	GELS_RAT	Q68FP1	Gelsolin	Gsn
Met I; Met II	GFAP_RAT	P47819	Glial fibrillary acidic protein	Gfap
Met II	GHITM_RAT	Q5XIA8	Growth hormone-inducible transmembrane protein	Ghitm
Met II	GL8D1_RAT	Q6AYF6	Glycosyltransferase 8 domain-containing protein 1	Glt8d1
Met I	GLOD4_RAT	Q5I0D1	Glyoxalase domain-containing protein 4	Glod4
Met II	GNA11_RAT	Q9JID2	Guanine nucleotide-binding protein alpha-11	Gna11
Met I; Met II	GNAI2_RAT	P04897	Guanine nucleotide-binding protein G(i) subunit alpha-2	Gnai2
Met I; Met II	GNAI3_RAT	P08753	Guanine nucleotide-binding protein G(k) subunit alpha	Gnai3
Met II	GNAQ_RAT	P82471	Guanine nucleotide-binding protein G(q) subunit alpha	Gnaq
Met I	GNAS1_RAT	Q63803	Guanine nucleotide-binding protein G(s) subunit alpha isoforms XLas	Gnas
Met I; Met II	GNAS2_RAT	P63095	Guanine nucleotide-binding protein G(s) subunit alpha	Gnas
Met I; Met II	GNAZ_RAT	P19627	Guanine nucleotide-binding protein G(z) subunit alpha	Gnaz
Met II	GOGA2_RAT	Q62839	Golgin subfamily A member 2	Golga2

Met II	GOGA7_RAT	Q6AYQ1	Golgin subfamily A member 7	Golga7
Met II	GOLI4_RAT	Q5BJK8	Golgi integral membrane protein 4	Golim4
Met II	GOSR2_RAT	O35165	Golgi SNAP receptor complex member 2	Gosr2
Met I; Met II	GPDM_RAT	P35571	Glycerol-3-phosphate dehydrogenase mitochondrial	Gpd2
Met II	GPSM3_RAT	Q6MG88	G-protein-signaling modulator 3	Gpsm3
Met I; Met II	GRP75_RAT	P48721	Mortalin	Hspa9
Met I; Met II	GRP78_RAT	P06761	Endoplasmic reticulum chaperone BiP	Hspa5
Met I; Met II	GTR1_RAT	P11167	Solute carrier family 2, facilitated glucose transporter member 1	Slc2a1
Met I; Met II	GTR3_RAT	Q07647	Solute carrier family 2, facilitated glucose transporter member 3	Slc2a3
Met I; Met II	H14_RAT	P15865	Histone H1.4	Hist1h1e
Met I; Met II	H4_RAT	P62804	Histone H4	Hist1h4b; Hist1h4m; Hist4h4
Met II	HA12_RAT	P16391	RT1 class I histocompatibility antigen AA alpha chain	---
Met I	HAP28_RAT	Q62785	28 kDa heat- and acid-stable phosphoprotein	Pdap1
Met I	HMCS1_RAT	P17425	Hydroxymethylglutaryl-CoA synthase cytoplasmic	Hmgcs1
Met II	HMOX1_RAT	P06762	Heme oxygenase 1	Hmox1
Met II	HMOX2_RAT	P23711	Heme oxygenase 2	Hmox2
Met II	HNRPF_RAT	Q794E4	Heterogeneous nuclear ribonucleoprotein F	Hnrnpf
Met II	HRG1_RAT	B0BNL4	Heme transporter HRG1	Slc48a1
Met I; Met II	HS90A_RAT	P82995	Heat shock protein HSP 90-alpha	Hsp90aa1
Met I; Met II	HS90B_RAT	P34058	Heat shock protein HSP 90-beta	Hsp90ab1
Met I; Met II	HSP72_RAT	P14659	Heat shock-related 70 kDa protein 2	Hspa2
Met I	HSP74_RAT	O88600	Heat shock 70 kDa protein 4	Hspa4
Met II	HSP7C_RAT	P63018	Heat shock cognate 71 kDa protein	Hspa8
Met I	HXK1_RAT	P05708	Hexokinase-1	Hk1
Met I	HXK2_RAT	P27881	Hexokinase-2	Hk2
Met II	ICAM1_RAT	Q00238	Intercellular adhesion molecule 1 (ICAM1)	Icam1
Met I	ICK_RAT	Q62726	Serine/threonine-protein kinase ICK	Ick
Met I; Met II	IF4A2_RAT	Q5RKI1	Eukaryotic initiation factor 4A-II	Eif4a2
Met I	IF4A3_RAT	Q3B8Q2	Eukaryotic initiation factor 4A-III	Eif4a3
Met I	IF5A1_RAT	Q3T1J1	Eukaryotic translation initiation factor 5A-1	Eif5a
Met I	IF6_RAT	Q3KRD8	Eukaryotic translation initiation factor 6	Eif6
Met II	IKIP_RAT	Q5EAJ6	Inhibitor kappa-B kinase-interacting protein	Ikbip
Met I	ILEUA_RAT	Q4G075	Leukocyte elastase inhibitor	Serpinb1a
Met I; Met II	ILRL1_RAT	Q62611	Interleukin 1 receptor-like 1	Il1rl1
Met I; Met II	IMB1_RAT	P52296	Importin subunit beta-1	Kpnb1
Met I; Met II	IMMT_RAT	Q3KR86	MICOS complex subunit Mic60	Immt
Met II	IMPA3_RAT	D4AD37	Inositol monophosphatase 3	Impad1
Met II	IR3IP_RAT	P85007	Immediate early response 3-interacting protein 1	Ier3ip1

Met II	IRK2_RAT	Q64273	Inward rectifier potassium channel 2	Kcnj2
Met II	ITAD_RAT	Q9QYE7	Integrin alpha D	Itgad
Met II	ITB1_RAT	P49134	Integrin beta 1	Itgb1
Met I; Met II	ITFG3_RAT	Q5M7W6	Protein FAM234A	Fam234a
Met II	ITM2B_RAT	Q5XIE8	Integral membrane protein 2B	Itm2b
Met I; Met II	ITM2C_RAT	Q5PQL7	Integral membrane protein 2C	Itm2c
Met II	ITPR2_RAT	P29995	Inositol 1,4,5-trisphosphate receptor type 2	Itpr2
Met II	ITPR3_RAT	Q63269	Inositol 1,4,5-trisphosphate receptor type 3	Itpr3
Met I; Met II	K1C10_RAT	Q6IFW6	Keratin, type I cytoskeletal 10	Krt10
Met I; Met II	K1C14_RAT	Q6IFV1	Keratin, type I cytoskeletal 14	Krt14
Met I; Met II	K1C15_RAT	Q6IFV3	Keratin, type I cytoskeletal 15	Krt15
Met I; Met II	K1C17_RAT	Q6IFU8	Keratin, type I cytoskeletal 17	Krt17
Met II	K1C18_RAT	Q5BJY9	Keratin, type I cytoskeletal 18	Krt18
Met II	K1C42_RAT	Q6IFU7	Keratin, type I cytoskeletal 42	Krt42
Met II	K22E_RAT	Q6IG02	Keratin, type II cytoskeletal 2 epidermal	Krt2
Met I; Met II	K2C1_RAT	Q6IMF3	Keratin, type II cytoskeletal 1	Krt1
Met I; Met II	K2C1B_RAT	Q6IG01	Keratin, type II cytoskeletal 1B	Krt77
Met II	K2C4_RAT	Q6IG00	Keratin, type II cytoskeletal 4	Krt4
Met I	K2C5_RAT	Q6P6Q2	Keratin, type II cytoskeletal 5	Krt5
Met I; Met II	K2C6A_RAT	Q4FZU2	Keratin, type II cytoskeletal 6A	Krt6a
Met II	K2C7_RAT	Q6IG12	Keratin, type II cytoskeletal 7	Krt7
Met I; Met II	K2C73_RAT	Q6IG03	Keratin, type II cytoskeletal 73	Krt73
Met I; Met II	K2C75_RAT	Q6IG05	Keratin, type II cytoskeletal 75	Krt75
Met I; Met II	K2C8_RAT	Q10758	Keratin, type II cytoskeletal 8	Krt8
Met I	KAP0_RAT	P09456	cAMP-dependent protein kinase type I-alpha regulatory subunit	Prkar1a
Met II	KAP2_RAT	P12368	cAMP-dependent protein kinase type II-alpha regulatory subunit	Prkar2a
Met II	KC1G1_RAT	Q62761	Casein kinase I isoform gamma-1	Csnk1g1
Met II	KC1G2_RAT	Q62762	Casein kinase I isoform gamma-2	Csnk1g2
Met II	KC1G3_RAT	Q62763	Casein kinase I isoform gamma-3	Csnk1g3
Met I	KCY_RAT	Q4KM73	UMP-CMP kinase	Cmpk1
Met I; Met II	KPYM_RAT	P11980	Pyruvate kinase PKM	Pkm
Met I	KPYR_RAT	P12928	Pyruvate kinase PKLR	Pklr
Met II	LAMP1_RAT	P14562	Lysosome-associated membrane glycoprotein 1 (Lamp1)	Lamp1
Met II	LAMP2_RAT	P17046	Lysosome-associated membrane glycoprotein 2 (Lamp2)	Lamp2
Met I; Met II	LAP2_RAT	Q62733	Lamina-associated polypeptide 2 isoform beta	Tmpo
Met I; Met II	LAT_RAT	O70601	Linker for activation of T-cells family member 1 (LAT)	Lat
Met II	LAT1_RAT	Q63016	Large neutral amino acids transporter small subunit 1	Slc7a5
Met II	LBR_RAT	O08984	Lamin-B receptor	Lbr

Met I	LCK_RAT	Q01621	Tyrosine-protein kinase LCK	Lck
Met I; Met II	LDHA_RAT	P04642	L-lactate dehydrogenase A chain	Ldha
Met II	LDLR_RAT	P35952	Low-density lipoprotein receptor	Ldlr
Met I	LEG1_RAT	P11762	Galectin-1	Lgals1
Met II	LETM1_RAT	Q5XIN6	Mitochondrial proton/calcium exchanger protein	Letm1
Met II	LEUK_RAT	P13838	Leukosialin	Spn
Met II	LMAN1_RAT	Q62902	Lectin mannose-binding 1	Lman1
Met II	LMBD1_RAT	Q6AZ61	Probable lysosomal cobalamin transporter	Lmbrd1
Met II	LMF2_RAT	A1L1J9	Lipase maturation factor 2	Lmf2
Met I; Met II	LPPRC_RAT	Q5SGE0	Leucine-rich PPR motif-containing protein mitochondrial	Lrpprc
Met I; Met II	LRC59_RAT	Q5RJR8	Leucine-rich repeat-containing protein 59	Lrrc59
Met I; Met II	LTOR1_RAT	Q6P791	Ragulator complex protein LAMTOR1	Lamtor1
Met II	LTOR3_RAT	Q5U204	Ragulator complex protein LAMTOR3	Lamtor3
Met I; Met II	LYN_RAT	Q07014	Tyrosine-protein kinase Lyn	Lyn
Met II	M0R402_RAT	M0R402	Protein disulfide-isomerase TMX3	Tmx3
Met I	M0R5K9_RAT	M0R5K9	40S ribosomal protein S18	---
Met I	M0R866_RAT	M0R866	Uncharacterized protein	---
Met I	M0R9L0_RAT	M0R9L0	Nascent polypeptide-associated complex subunit alpha	Naca
Met II	M0RAJ8_RAT	M0RAJ8	40S ribosomal protein S2	---
Met I	M0RAU0_RAT	M0RAU0	Advanced glycosylation end product-specific receptor	Ager
Met II	M0RBJ0_RAT	M0RBJ0	Guanine nucleotide-binding protein gamma-2	Gng2
Met II	M0RC99_RAT	M0RC99	Ras-related protein Rab-5a	Rab5a
Met II	M0RCA3_RAT	M0RCA3	Zinc finger protein-like 1	Zfpl1
Met II	M0RCF3_RAT	M0RCF3	Integrin beta 3	Itgb3
Met II	M0RCS2_RAT	M0RCS2	Transducin beta-like protein 2	Tbl2
Met II	M0RD75_RAT	M0RD75	40S ribosomal protein S6	Rps6
Met II	M0RDI5_RAT	M0RDI5	Mitochondrial calcium uniporter	Mcu
Met I	M0RDJ4_RAT	M0RDJ4	Glia maturation factor beta	Gmfb
Met I	MAK_RAT	P20793	Serine/threonine-protein kinase MAK	Mak
Met I	MANF_RAT	P0C5H9	Mesencephalic astrocyte-derived neurotrophic factor	Manf
Met I	MARE1_RAT	Q66HR2	Microtubule-associated protein RP/EB family member 1	Mapre1
Met II	MAVS_RAT	Q66HG9	Mitochondrial antiviral-signaling protein	Mavs
Met II	MBOA5_RAT	Q5FVN0	Lysophospholipid acyltransferase 5	Lpcat3
Met I	MDHC_RAT	O88989	Malate dehydrogenase cytoplasmic	Mdh1
Met I; Met II	MDHM_RAT	P04636	Malate dehydrogenase mitochondrial	Mdh2
Met II	MGAT2_RAT	Q09326	Alpha-1,6-mannosyl-glycoprotein 2-beta-N-acetylglucosaminyltransferase	Mgat2
Met II	MIRO2_RAT	Q7TSA0	Mitochondrial Rho GTPase 2	Rhot2
Met I	MK03_RAT	P21708	Mitogen-activated protein kinase 3	Mapk3

Met II	MLEC_RAT	Q5FVQ4	Malectin	Mlec
Met II	MMGT1_RAT	B5DF51	Membrane magnesium transporter 1	Mmgt1
Met II	MOGS_RAT	O88941	Mannosyl-oligosaccharide glucosidase	Mogs
Met II	MOSC2_RAT	O88994	Mitochondrial amidoxime reducing component 2	Marc2
Met I; Met II	MOT1_RAT	P53987	Monocarboxylate transporter 1	Slc16a1
Met I	MPRD_RAT	Q6AY20	Cation-dependent mannose-6-phosphate receptor	M6pr
Met II	MRLCA_RAT	P13832	Myosin regulatory light chain RLC-A	Rlc-a
Met II	MSMO1_RAT	O35532	Methylsterol monooxygenase 1	Msmo1
Met I	MTPN_RAT	P62775	Myotrophin	Mtpn
Met II	MYH10_RAT	Q9JLT0	Myosin heavy chain 10	Myh10
Met I; Met II	MYL6_RAT	Q64119	Myosin light polypeptide 6	Myl6
Met II	NB5R3_RAT	P20070	NADH-cytochrome b5 reductase 3	Cyb5r3
Met I	NCF2_RAT	A7E3N2	Neutrophil cytosol factor 2	Ncf2
Met II	NCLN_RAT	Q5XIA1	Nicalin	Ncln
Met I; Met II	NCPR_RAT	P00388	NADPH--cytochrome P450 reductase	Por
Met II	NCUG1_RAT	Q68FV6	Glycosylated lysosomal membrane protein	Glmp
Met I	NDKA_RAT	Q05982	Nucleoside diphosphate kinase A	Nme1
Met I	NDKB_RAT	P19804	Nucleoside diphosphate kinase B	Nme2
Met II	NDUAA_RAT	Q561S0	NADH dehydrogenase [ubiquinone] 1 alpha subcomplex subunit 10	Ndufa10
Met II	NDUF5_RAT	B2GV71	NADH dehydrogenase [ubiquinone] 1 alpha subcomplex assembly factor 5	Ndufaf5
Met I; Met II	NDUS1_RAT	Q66HF1	NADH-ubiquinone oxidoreductase 75 kDa subunit mitochondrial	Ndufs1
Met II	NDUS2_RAT	Q641Y2	NADH dehydrogenase [ubiquinone] iron-sulfur protein 2	Ndufs2
Met I; Met II	NDUV2_RAT	P19234	NADH dehydrogenase [ubiquinone] flavoprotein 2 mitochondrial	Ndufv2
Met I	NEDD4_RAT	Q62940	E3 ubiquitin-protein ligase NEDD4	Nedd4
Met I	NHRF1_RAT	Q9JJ19	Na(+)/H(+) exchange regulatory cofactor NHE-RF1	Slc9a3r1
Met I; Met II	NICA_RAT	Q8CGU6	Nicastrin	Ncstn
Met I	NP1L4_RAT	Q5U2Z3	Nucleosome assembly protein 1-like 4	Nap1l4
Met I	NPM_RAT	P13084	Nucleophosmin	Npm1
Met II	NPTN_RAT	P97546	Neuroplastin	Nptn
Met I	NQO1_RAT	P05982	NAD(P)H dehydrogenase [quinone] 1	Nqo1
Met I	NSDHL_RAT	Q5PPL3	Sterol-4-alpha-carboxylate 3-dehydrogenase decarboxylating	Nsdhl
Met I	NSF1C_RAT	O35987	NSFL1 cofactor p47	Nsfl1c
Met I	NTF2_RAT	P61972	Nuclear transport factor 2	Nutf2
Met II	NUCB1_RAT	Q63083	Nucleobindin-1	Nucb1
Met II	NUCB2_RAT	Q9JI85	Nucleobindin-2	Nucb2
Met I; Met II	NUCL_RAT	P13383	Nucleolin	Ncl
Met II	O89035_RAT	O89035	Solute carrier family 25 member 10	Slc25a10
Met II	OCAD1_RAT	Q5XIG4	OCIA domain-containing protein 1	Ociad1

Met II	ODO1_RAT	Q5XI78	2-oxoglutarate dehydrogenase complex component E1	Ogdh
Met II	ODO2_RAT	Q01205	2-oxoglutarate dehydrogenase complex component E2	Dlst
Met I; Met II	ODP2_RAT	P08461	Pyruvate dehydrogenase component E2	Dlat
Met II	ODPB_RAT	P49432	Pyruvate dehydrogenase component E1 beta	Pdhb
Met II	OGFD3_RAT	Q5M843	2-oxoglutarate and iron-dependent oxygenase domain-containing protein 3	Ogfod3
Met I	ORN_RAT	Q5U1X1	Oligoribonuclease mitochondrial	Rexo2
Met II	OS9_RAT	Q5RKH6	Protein OS-9	Os9
Met II	OST48_RAT	Q641Y0	Oligosaccharyl transferase 48 kDa subunit	Ddost
Met I	OTUB1_RAT	B2RYG6	Ubiquitin thioesterase OTUB1	Otub1
Met II	P2RX4_RAT	P51577	P2X purinoceptor 4	P2rx4
Met II	P4K2B_RAT	Q5XIL2	Phosphatidylinositol 4-kinase type 2-beta	Pi4k2b
Met II	PABP1_RAT	Q9EPH8	Polyadenylate-binding protein 1	Pabpc1
Met I	PAIRB_RAT	Q6AXS5	Plasminogen activator inhibitor 1 RNA-binding protein	Serbp1
Met I	PARK7_RAT	O88767	Protein/nucleic acid deglycase DJ-1	Park7
Met II	PBIP1_RAT	A2VD12	Pre-B-cell leukemia transcription factor-interacting protein 1	Pbxip1
Met II	PCAT2_RAT	P0C1Q3	Lysophosphatidylcholine acyltransferase 2	Lpcat2
Met I	PCNA_RAT	P04961	Proliferating cell nuclear antigen	Pcna
Met II	PDE3B_RAT	Q63085	Cyclic GMP-inhibited phosphodiesterase B	Pde3b
Met I; Met II	PDIA1_RAT	P04785	Protein disulfide-isomerase	P4hb
Met I; Met II	PDIA3_RAT	P11598	Protein disulfide-isomerase A3	Pdia3
Met I	PDIA6_RAT	Q63081	Protein disulfide-isomerase A6	Pdia6
Met I	PEBP1_RAT	P31044	Phosphatidylethanolamine-binding protein 1	Pebp1
Met II	PECA1_RAT	Q3SWT0	Platelet endothelial cell adhesion molecule (Pecam1)	Pecam1
Met I	PGAM1_RAT	P25113	Phosphoglycerate mutase 1	Pgam1
Met II	PGFRB_RAT	Q05030	Platelet-derived growth factor receptor beta	Pdgfrb
Met I	PGH2_RAT	P35355	Prostaglandin G/H synthase 2	Ptgs2
Met I	PGK1_RAT	P16617	Phosphoglycerate kinase 1	Pgk1
Met I; Met II	PGRC1_RAT	P70580	Membrane-associated progesterone receptor component 1	Pgrmc1
Met II	PGRC2_RAT	Q5XIU9	Membrane-associated progesterone receptor component 2	Pgrmc2
Met I; Met II	PHB_RAT	P67779	Prohibitin	Phb
Met I; Met II	PHB2_RAT	Q5XIH7	Prohibitin 2	Phb2
Met II	PHOCN_RAT	Q9QYW3	MOB-like protein phocein	Mob4
Met II	PIGS_RAT	Q5XI31	GPI transamidase component PIG-S	Pigs
Met II	PLF4_RAT	P06765	Platelet factor 4	Pf4
Met II	PLP2_RAT	Q6P742	Proteolipid protein 2	Plp2
Met I; Met II	PLS1_RAT	P58195	Phospholipid scramblase 1	Plscr1
Met II	PLS3_RAT	Q6QBQ4	Phospholipid scramblase 3	Plscr3
Met II	PORED_RAT	Q5RJM1	3-oxo-5-alpha-steroid 4-dehydrogenase 3	Srd5a3

Met II	PP1A_RAT	P62138	Serine/threonine-protein phosphatase PP1-alpha catalytic subunit	Ppp1ca
Met I	PP2AA_RAT	P63331	Serine/threonine-protein phosphatase 2A catalytic subunit alpha isoform	Ppp2ca
Met I; Met II	PPIA_RAT	P10111	Peptidyl-prolyl cis-trans isomerase A	Ppia
Met I; Met II	PPIB_RAT	P24368	Peptidyl-prolyl cis-trans isomerase B	Ppib
Met I; Met II	PRAF3_RAT	Q9ES40	PRA1 family protein 3	Arl6ip5
Met I; Met II	PRDX1_RAT	Q63716	Peroxiredoxin-1	Prdx1
Met I	PRDX2_RAT	P35704	Peroxiredoxin-2	Prdx2
Met II	PRDX3_RAT	Q9Z0V6	Thioredoxin-dependent peroxide reductase mitochondrial	Prdx3
Met I; Met II	PROF1_RAT	P62963	Profilin-1	Pfn1
Met II	PSN1_RAT	P97887	Presenilin-1	Psen1
Met II	PSN2_RAT	O88777	Presenilin-2	Psen2
Met II	PTN1_RAT	P20417	Protein-tyrosine phosphatase 1B	Ptpn1
Met II	PTPRC_RAT	P04157	Receptor-type tyrosine-protein phosphatase C (CD45)	Ptprc
Met I	PUR9_RAT	O35567	Bifunctional purine biosynthesis protein PURH	Atic
Met I	PYGL_RAT	P09811	Glycogen phosphorylase liver form	Pygl
Met II	Q0R3W9_RAT	Q0R3W9	GTPase, IMAP family member 1	Gimap1
Met II	Q2I6B1_RAT	Q2I6B1	V-type proton ATPase subunit A2	Atp6v0a2
Met I; Met II	Q2I6B2_RAT	Q2I6B2	V-type proton ATPase subunit A1	Atp6v0a1
Met II	Q2IBC7_RAT	Q2IBC7	Hepatocyte growth factor receptor	Met
Met I; Met II	Q32PX6_RAT	Q32PX6	Rho-related GTP-binding protein RhoG	Rhog
Met II	Q3B7V5_RAT	Q3B7V5	Ras-related protein Rab-2b	Rab2b
Met II	Q3KRC3_RAT	Q3KRC3	Signal recognition particle receptor alpha	Srpra
Met II	Q3MHS9_RAT	Q3MHS9	Chaperonin-containing TCP1 subunit 6A	Cct6a
Met II	Q497A2_RAT	Q497A2	Solute carrier family 35 member B2	Slc35b2
Met I	Q4G002_RAT	Q4G002	Histocompatibility 2 T region locus 24	RT1-T24-4
Met I; Met II	Q4KM97_RAT	Q4KM97	Tyrosine-protein kinase BLK	Blk
Met II	Q4QQS6_RAT	Q4QQS6	Dolichyl-phosphate beta-glucosyltransferase	Alg5
Met II	Q4QQV0_RAT	Q4QQV0	Tubulin beta-6	Tubb6
Met I; Met II	Q4V7D1_RAT	Q4V7D1	Translocon-associated protein subunit alpha	Ssr1
Met I; Met II	Q4V7D9_RAT	Q4V7D9	Acid sphingomyelinase-like phosphodiesterase 3B	Smpdl3b
Met II	Q4V8N6_RAT	Q4V8N6	Solute carrier organic anion transporter family member 2A1	Slco4a1
Met II	Q52KJ9_RAT	Q52KJ9	Thioredoxin-related transmembrane protein 1	Tmx1
Met II	Q5BJZ3_RAT	Q5BJZ3	Nicotinamide nucleotide transhydrogenase	Nnt
Met II	Q5EAP4_RAT	Q5EAP4	Guanine nucleotide binding protein alpha-14	Gna14
Met II	Q5FVC4_RAT	Q5FVC4	DnaJ (Hsp40) homolog subfamily B member 12	Dnajb12
Met I; Met II	Q5M7T6_RAT	Q5M7T6	V-type proton ATPase subunit D 1	Atp6v0d1
Met I	Q5M860_RAT	Q5M860	Rho GDP dissociation inhibitor 2	Arhgdib
Met I	Q5RJK5_RAT	Q5RJK5	Chromobox 3	Cbx3

Met II	Q5RK10_RAT	Q5RK10	60S ribosomal protein L13a, isoform	Rpl13a
Met II	Q5RKJ9_RAT	Q5RKJ9	Ras-related protein Rab-10	Rab10
Met I; Met II	Q5U1Y2_RAT	Q5U1Y2	Ras-related C3 botulinum toxin substrate 2 (Rac2)	Rac2
Met II	Q5U1Z9_RAT	Q5U1Z9	Metaxin 2	Mtx2
Met II	Q5U2Z8_RAT	Q5U2Z8	Elongation of very long chain fatty acids protein 1	Elovl1
Met I	Q5U362_RAT	Q5U362	Annexin A4	Anxa4
Met I; Met II	Q5U3Z7_RAT	Q5U3Z7	Serine hydroxymethyltransferase	Shmt2
Met II	Q5VLR5_RAT	Q5VLR5	Endoplasmic reticulum resident protein 44	Erp44
Met II	Q5XI04_RAT	Q5XI04	Erythrocyte band 7 integral membrane protein	Stom
Met I; Met II	Q5XI34_RAT	Q5XI34	Serine/threonine-protein phosphatase 2A 65 kDa regulatory subunit A alpha isoform	Ppp2r1a
Met II	Q5XI36_RAT	Q5XI36	CD97	Adgre5
Met I	Q5XI38_RAT	Q5XI38	Lymphocyte cytosolic protein 1	Lcp1
Met I	Q5XI77_RAT	Q5XI77	Annexin A11	Anxa11
Met II	Q5XI86_RAT	Q5XI86	Peptidyl-tRNA hydrolase 2	Ptrh2
Met II	Q5XIH3_RAT	Q5XIH3	NADH dehydrogenase [ubiquinone] flavoprotein 1	Ndufv1
Met I	Q5XIM7_RAT	Q5XIM7	Lysine--tRNA ligase	Kars
Met II	Q5XIP2_RAT	Q5XIP2	GPI-anchor transamidase	Pigk
Met II	Q5XIU4_RAT	Q5XIU4	B-cell receptor-associated protein 29	Bcap29
Met I	Q5XIV1_RAT	Q5XIV1	Phosphoglycerate kinase 2	Pgk2
Met I; Met II	Q63116_RAT	Q63116	Mast/stem cell growth factor receptor	Kit
Met II	Q63483_RAT	Q63483	Ras-related protein Rab-38	Rab38
Met II	Q642E3_RAT	Q642E3	CDK5 regulatory subunit-associated protein 3, isoform	Cdk5rap3
Met II	Q66H18_RAT	Q66H18	Synaptophysin-like 1	Sypl1
Met I	Q68FX4_RAT	Q68FX4	Hematopoietic cell-specific LYN substrate 1 (hcls1)	Hcls1
Met II	Q6AXX5_RAT	Q6AXX5	Retinol dehydrogenase 11	Rdh11
Met II	Q6AY18_RAT	Q6AY18	GTP-binding protein SAR1a	Sar1a
Met I; Met II	Q6AYD3_RAT	Q6AYD3	Proliferation-associated protein 2G4	Pa2g4
Met I; Met II	Q6AYI1_RAT	Q6AYI1	Probable ATP-dependent RNA helicase DDX5	Ddx5
Met II	Q6AYM8_RAT	Q6AYM8	Glycosylphosphatidylinositol anchor attachment 1	Gpaa1
Met II	Q6AYQ9_RAT	Q6AYQ9	Peptidyl-prolyl cis-trans isomerase C	Ppic
Met I; Met II	Q6IFU9_RAT	Q6IFU9	keratin 16	Krt16
Met II	Q6IFV6_RAT	Q6IFV6	Keratin 35	Krt35
Met II	Q6IFZ5_RAT	Q6IFZ5	Keratin, type II cytoskeletal 2 oral	Krt76
Met II	Q6IN24_RAT	Q6IN24	Galectin-8	Lgals8
Met I	Q6IN37_RAT	Q6IN37	GM2 ganglioside activator	Gm2a
Met II	Q6IRF8_RAT	Q6IRF8	V-type proton ATPase subunit S1	Atp6ap1
Met I; Met II	Q6MGA3_RAT	Q6MGA3	Antigen peptide transporter 2	Tap2
Met II	Q6MGB4_RAT	Q6MGB4	H2-K region expressed gene 4, rat orthologue	Slc39a7

Met I; Met II	Q6P3V8_RAT	Q6P3V8	Eukaryotic initiation factor 4A-I	Eif4a1
Met II	Q6P3V9_RAT	Q6P3V9	60S ribosomal protein L4	Rpl4
Met II	Q6P503_RAT	Q6P503	V-type proton ATPase subunit D	Atp6v1d
Met I; Met II	Q6P685_RAT	Q6P685	Eukaryotic translation initiation factor 2 subunit 2	Eif2s2
Met I	Q6P6T6_RAT	Q6P6T6	Cathepsin D	Ctsd
Met II	Q6P9V1_RAT	Q6P9V1	CD81 molecule	Cd81
Met II	Q6PCU4_RAT	Q6PCU4	Emerin	Emd
Met II	Q6PDV6_RAT	Q6PDV6	40S ribosomal protein S14	LOC100911847
Met I	Q6TXF3_RAT	Q6TXF3	Acyl-CoA-binding protein	Dbi
Met I; Met II	Q76GL9_RAT	Q76GL9	Neutral amino acid transporter A	Slc1a4
Met I; Met II	Q78P65_RAT	Q78P65	Rap2A-like protein	Rap2a
Met I	Q7TP15_RAT	Q7TP15	S-methyl-5'-thioadenosine phosphorylase	Mtap
Met II	Q7TP42_RAT	Q7TP42	Translocation protein SEC62	Sec62
Met II	Q7TQ86_RAT	Q7TQ86	Ac1158	Rsl1d1
Met II	Q8SEZ5_RAT	Q8SEZ5	Cytochrome c oxidase subunit 2	Mt-co2
Met II	Q99JC6_RAT	Q99JC6	Tapasin	Tapbp
Met I	Q9JKB7_RAT	Q9JKB7	Guanine deaminase	Gda
Met I	Q9QX80_RAT	Q9QX80	Heterogeneous nuclear ribonucleoprotein A/B	Hnrnpab
Met II	Q9Z1J7_RAT	Q9Z1J7	Amino acid transporter	Slc1a5
Met I; Met II	QCR1_RAT	Q68FY0	Cytochrome b-c1 complex subunit 1 mitochondrial	Uqcrc1
Met I; Met II	QCR2_RAT	P32551	Cytochrome b-c1 complex subunit 2 mitochondrial	Uqcrc2
Met I; Met II	QCR6_RAT	Q5M9I5	Cytochrome b-c1 complex subunit 6 mitochondrial	Uqcrh
Met II	R9PXR4_RAT	R9PXR4	Mitochondrial import receptor subunit TOM70	Tomm70
Met I	R9PXR7_RAT	B2GV92	Prostaglandin E synthase 3	Ptges3
Met I	R9PXU4_RAT	R9PXU4	Thioredoxin reductase 1 cytoplasmic	Txnrd1
Met II	RAB14_RAT	P61107	Ras-related protein Rab-14	Rab14
Met I; Met II	RAB15_RAT	P35289	Ras-related protein Rab-15	Rab15
Met II	RAB18_RAT	Q5EB77	Ras-related protein Rab-18	Rab18
Met I; Met II	RAB1A_RAT	Q6NYB7	Ras-related protein Rab-1a	Rab1A
Met II	RAB21_RAT	Q6AXT5	Ras-related protein Rab-21	Rab21
Met I; Met II	RAB2A_RAT	P05712	Ras-related protein Rab-2a	Rab2a
Met I; Met II	RAB35_RAT	Q5U316	Ras-related protein Rab-35	Rab35
Met II	RAB3A_RAT	P63012	Ras-related protein Rab-3a	Rab3a
Met II	RAB3D_RAT	Q63942	Ras-related protein Rab-3d	Rab3d
Met II	RAB43_RAT	Q53B90	Ras-related protein Rab-43	Rab43
Met I; Met II	RAB4B_RAT	P51146	Ras-related protein Rab-4b	Rab4b
Met II	RAB6A_RAT	Q9WVB1	Ras-related protein Rab-6a	Rab6a
Met I; Met II	RAB7A_RAT	P09527	Ras-related protein Rab-7a	Rab7a

Met II	RAB8A_RAT	P35280	Ras-related protein Rab-8a	Rab8a
Met I; Met II	RAB8B_RAT	P70550	Ras-related protein Rab-8b	Rab8b
Met II	RAB9A_RAT	Q99P75	Ras-related protein Rab-9a	Rab9a
Met I; Met II	RAC1_RAT	Q6RUV5	Ras-related C3 botulinum toxin substrate 1 (Rac1)	Rac1
Met I	RACK1_RAT	P63245	Receptor of activated protein C kinase 1 (RACK1)	Rack1
Met II	RALA_RAT	P63322	Ras-related protein Ral-A	Rala
Met I	RAN_RAT	P62828	GTP-binding nuclear protein Ran	Ran
Met I; Met II	RAP1A_RAT	P62836	Ras-related protein Rap-1a	Rap1a
Met I; Met II	RAP1B_RAT	Q62636	Ras-related protein Rap-1b	Rap1b
Met I; Met II	RAP2B_RAT	P61227	Ras-related protein Rap-2b	Rap2b
Met II	RASH_RAT	P20171	GTPase HRas	Hras
Met II	RASK_RAT	P08644	GTPase KRas	Kras
Met II	RASN_RAT	Q04970	GTPase NRas	Nras
Met I; Met II	RB11B_RAT	O35509	Ras-related protein Rab-11b	Rab11b
Met II	RB27A_RAT	P23640	Ras-related protein Rab-27a	Rab27a
Met II	RB27B_RAT	Q99P74	Ras-related protein Rab-27b	Rab27b
Met II	RENR_RAT	Q6AXS4	Renin receptor	Atp6ap2
Met II	RER1_RAT	Q498C8	Protein RER1	Rer1
Met I; Met II	RGS19_RAT	O70521	Regulator of G-protein signaling 19	Rgs19
Met II	RHBL4_RAT	Q4V8F3	Rhomboid-related protein 4	Rhbdd1
Met I; Met II	RHOA_RAT	P61589	Transforming protein RhoA	Rhoa
Met II	RHOB_RAT	P62747	Rho-related GTP-binding protein RhoB	Rhob
Met II	RL10_RAT	Q6PDV7	60S ribosomal protein L10	Rpl10
Met I; Met II	RL10A_RAT	P62907	60S ribosomal protein L10a	Rpl10a
Met II	RL11_RAT	P62914	60S ribosomal protein L11	Rpl11
Met II	RL12_RAT	P23358	60S ribosomal protein L12	Rpl12
Met II	RL13A_RAT	P35427	60S ribosomal protein L13a	Rpl13a
Met II	RL14_RAT	Q63507	60S ribosomal protein L14	Rpl14
Met II	RL17_RAT	P24049	60S ribosomal protein L17	Rpl17
Met II	RL18_RAT	P12001	60S ribosomal protein L18	Rpl18
Met II	RL18A_RAT	P62718	60S ribosomal protein L18a	Rpl18a
Met II	RL19_RAT	P84100	60S ribosomal protein L19	Rpl19
Met II	RL23_RAT	P62832	60S ribosomal protein L23	Rpl23
Met II	RL24_RAT	P83732	60S ribosomal protein L24	Rpl24
Met II	RL3_RAT	P21531	60S ribosomal protein L3	Rpl3
Met I; Met II	RL30_RAT	P62890	60S ribosomal protein L30	Rpl30
Met I; Met II	RL40_RAT	P62986	Ubiquitin-60S ribosomal protein L40	Uba52
Met I	RL5_RAT	P09895	60S ribosomal protein L5	Rpl5

Met I; Met II	RL7_RAT	P05426	60S ribosomal protein L7	Rpl7
Met II	RLA0_RAT	P19945	60S acidic ribosomal protein P0	Rplp0
Met I; Met II	RLA1_RAT	P19944	60S acidic ribosomal protein P1	Rplp1
Met I	RLA2_RAT	P02401	60S acidic ribosomal protein P2	Rplp2
Met II	RMD3_RAT	Q66H15	Regulator of microtubule dynamics protein 3	Rmdn3
Met I	ROA3_RAT	Q6URK4	Heterogeneous nuclear ribonucleoprotein A3	Hnrnpa3
Met I	RPN1_RAT	P07153	Dolichyl-diphosphooligosaccharide protein glycosyltransferase subunit 1	Rpn1
Met I; Met II	RPN2_RAT	P25235	Dolichyl-diphosphooligosaccharide protein glycosyltransferase subunit 2	Rpn2
Met II	RRAGA_RAT	Q63486	Ras-related GTP-binding protein A (Rag A)	Rraga
Met II	RS11_RAT	P62282	40S ribosomal protein S11	Rps11
Met II	RS13_RAT	P62278	40S ribosomal protein S13	Rps13
Met II	RS15A_RAT	P62246	40S ribosomal protein S15a	Rps15a
Met II	RS16_RAT	P62250	40S ribosomal protein S16	Rps16
Met I	RS19_RAT	P17074	40S ribosomal protein S19	Rps19
Met II	RS27A_RAT	P62982	Ubiquitin-40S ribosomal protein S27a	Rps27a
Met I; Met II	RS28_RAT	P62859	40S ribosomal protein S28	Rps28
Met I; Met II	RS3_RAT	P62909	40S ribosomal protein S3	Rps3
Met II	RS3A_RAT	P49242	40S ribosomal protein S3a	Rps3a
Met II	RS4X_RAT	P62703	40S ribosomal protein S4, X isoform	Rps4x
Met I; Met II	RS7_RAT	P62083	40S ribosomal protein S7	Rps7
Met I; Met II	RS8_RAT	P62243	40S ribosomal protein S8	Rps8
Met I; Met II	RSSA_RAT	P38983	40S ribosomal protein SA	Rpsa
Met I; Met II	RTN4_RAT	Q9JK11	Reticulon-4	Rtn4
Met I; Met II	S38A2_RAT	Q9JHE5	Sodium-coupled neutral amino acid transporter 2	Slc38a2
Met II	S61A1_RAT	P61621	SEC61 alpha-1	Sec61a1
Met II	SAC1_RAT	Q9ES21	Phosphatidylinositide phosphatase SAC1	Sacm1l
Met I	SAE1_RAT	Q6AXQ0	SUMO-activating enzyme subunit 1	Sae1
Met II	SAM50_RAT	Q6AXV4	Sorting and assembly machinery component 50 homolog	Samm50
Met I; Met II	SAP_RAT	P10960	Prosaposin	Psap
Met II	SARAF_RAT	Q6AYN2	Store-operated calcium entry-associated regulatory factor (SARAF)	Saraf
Met II	SC11A_RAT	P42667	SEC11 homolog A	Sec11a
Met II	SC11C_RAT	Q9WTR7	SEC11 homolog C	Sec11c
Met I; Met II	SC22B_RAT	Q4KM74	Vesicle-trafficking protein SEC22b	Sec22b
Met II	SCFD1_RAT	Q62991	Syntaxin-binding protein 1-like 2	Scfd1
Met II	SCRB2_RAT	P27615	Lysosome membrane protein 2 (CD36)	Scarb2
Met II	SELT_RAT	Q1H5H1	Thioredoxin reductase-like selenoprotein T	Selenot
Met I	SEPT2_RAT	Q91Y81	Septin-2	Sept2
Met I	SERA_RAT	O08651	D-3-phosphoglycerate dehydrogenase	Phgdh

Met I	SET_RAT	Q63945	Protein SET	Set
Met I; Met II	SFXN1_RAT	Q63965	Sideroflexin-1	Sfxn1
Met II	SGPL1_RAT	Q8CHN6	Sphingosine-1-phosphate lyase 1	Sgpl1
Met II	SIA4C_RAT	P61131	Beta-galactoside alpha-2,3-sialyltransferase 4	St3gal4
Met I; Met II	SNAA_RAT	P54921	Alpha-soluble NSF attachment protein (SNAP-alpha)	Napa
Met I; Met II	SND1_RAT	Q66X93	Staphylococcal nuclease domain-containing protein 1	Snd1
Met II	SNG1_RAT	Q62876	Synaptogyrin-1	Syngr1
Met II	SNG2_RAT	O54980	Synaptogyrin-2	Syngr2
Met II	SNP23_RAT	O70377	Synaptosomal-associated protein 23 (SNAP-23)	Snap23
Met II	SO4A1_RAT	Q99N01	Organic anion-transporting polypeptide E	Slco4a1
Met I	SODC_RAT	P07632	Superoxide dismutase [Cu-Zn]	Sod1
Met II	SPNS1_RAT	Q2YDU8	Protein spinster homolog 1	Spns1
Met II	SQSTM_RAT	O08623	Sequestosome-1	Sqstm1
Met II	SRPRB_RAT	Q4FZX7	Signal recognition particle receptor beta	Srprb
Met I	SRSF2_RAT	Q6PDU1	Serine/arginine-rich splicing factor 2	Srsf2
Met II	SSRD_RAT	Q07984	Translocon-associated protein subunit delta	Ssr4
Met II	STIM1_RAT	P84903	Stromal interaction molecule 1 (STIM1)	Stim1
Met II	STML2_RAT	Q4FZT0	Stomatin-like protein 2	Stoml2
Met II	STRN3_RAT	P58405	Striatin-3	Strn3
Met I; Met II	STX12_RAT	G3V7P1	Syntaxin-12	Stx12
Met II	STX3_RAT	Q08849	Syntaxin-3	Stx3
Met II	STX5_RAT	Q08851	Syntaxin-5	Stx5
Met II	STX6_RAT	Q63635	Syntaxin-6	Stx6
Met II	STX7_RAT	O70257	Syntaxin-7	Stx7
Met II	STX8_RAT	Q9Z2Q7	Syntaxin-8	Stx8
Met II	STXB2_RAT	Q62753	Syntaxin-binding protein 2	Stxbp2
Met II	STXB5_RAT	Q9WU70	Syntaxin-binding protein 5	Stxbp5
Met I	SYAC_RAT	P50475	Alanine--tRNA ligase cytoplasmic	Aars
Met I	SYG_RAT	Q5I0G4	Glycine--tRNA ligase	Gars
Met I	SYSC_RAT	Q6P799	Serine--tRNA ligase cytoplasmic	Sars
Met II	SYVC_RAT	Q04462	Valine--tRNA ligase	Vars
Met I	SYYC_RAT	Q4KM49	Tyrosine--tRNA ligase cytoplasmic	Yars
Met II	T106B_RAT	Q6AYA5	Transmembrane protein 106 B	Tmem106b
Met II	T120A_RAT	Q5HZE2	Transmembrane protein 120 A	Tmem120a
Met I	TAGL2_RAT	Q5XFX0	Transgelin-2	Tagln2
Met II	TAP1_RAT	P36370	Antigen peptide transporter 1	Tap1
Met II	TAXB1_RAT	Q66HA4	Tax1-binding protein 1 homolog	Tax1bp1
Met I	TBA1A_RAT	P68370	Tubulin alpha-1A	Tuba1a

Met II	TBA1B_RAT	Q6P9V9	Tubulin alpha-1B	Tuba1b
Met I; Met II	TBA1C_RAT	Q6AYZ1	Tubulin alpha-1C	Tuba1c
Met I; Met II	TBA4A_RAT	Q5XIF6	Tubulin alpha-4A	Tuba4a
Met II	TBA8_RAT	Q6AY56	Tubulin alpha-8	Tuba8
Met I	TBB2A_RAT	P85108	Tubulin beta-2A	Tubb2a
Met I	TBB2B_RAT	Q3KRE8	Tubulin beta-2B	Tubb2b
Met I	TBB3_RAT	Q4QRB4	Tubulin beta-3	Tubb3
Met I; Met II	TBB4B_RAT	Q6P9T8	Tubulin beta-4B	Tubb4b
Met I; Met II	TBB5_RAT	P69897	Tubulin beta-5	Tubb5
Met I	TCP4_RAT	Q63396	Activated RNA polymerase II transcriptional coactivator p15	Sub1
Met II	TCPB_RAT	Q5XIM9	T-complex protein 1 beta	Cct2
Met II	TCPE_RAT	Q68FQ0	T-complex protein 1 epsilon	Cct5
Met II	TECR_RAT	Q64232	Very-long-chain enoyl-CoA reductase	Tecr
Met I; Met II	TERA_RAT	P46462	Transitional endoplasmic reticulum ATPase	Vcp
Met II	TGON3_RAT	P19814	Trans-Golgi network integral membrane protein TGN38	Ttgn1
Met I	THIO_RAT	P11232	Thioredoxin	Txn
Met I; Met II	THY1_RAT	P01830	Thy-1 membrane glycoprotein	Thy1
Met I; Met II	TKT_RAT	P50137	Transketolase	Tkt
Met II	TM109_RAT	Q6AYQ4	Transmembrane protein 109	Tmem109
Met I; Met II	TM165_RAT	Q4V899	Transmembrane protein 165	Tmem165
Met II	TM168_RAT	Q5PQM0	Transmembrane protein 168	Tmem168
Met II	TM38B_RAT	Q68FV1	Trimeric intracellular cation channel type B (TRIC-B)	Tmem38b
Met II	TM55A_RAT	Q4V888	Type 2 phosphatidylinositol 4,5-bisphosphate 4-phosphatase	Pip4p2
Met II	TM7S3_RAT	Q5FVF4	Transmembrane 7 superfamily member 3	Tm7sf3
Met II	TM9S1_RAT	Q66HF2	Transmembrane 9 superfamily member 1	Tm9sf1
Met II	TM9S2_RAT	Q66HG5	Transmembrane 9 superfamily member 2	Tm9sf2
Met II	TM9S4_RAT	Q4KLL4	Transmembrane 9 superfamily member 4	Tm9sf4
Met II	TMCO1_RAT	Q5I0H4	Calcium load-activated calcium channel (CLAC channel)	Tmco1
Met II	TMED1_RAT	Q5BK85	Transmembrane emp24 domain-containing protein 1	Tmed1
Met II	TMED2_RAT	Q63524	Transmembrane emp24 domain-containing protein 2	Tmed2
Met II	TMED5_RAT	Q6AXN3	Transmembrane emp24 domain-containing protein 5	Tmed5
Met I; Met II	TMED7_RAT	D3ZTX0	Transmembrane emp24 domain-containing protein 7	Tmed7
Met I; Met II	TMED9_RAT	Q5I0E7	Transmembrane emp24 domain-containing protein 9	Tmed9
Met I; Met II	TMEDA_RAT	Q63584	Transmembrane emp24 domain-containing protein 10	Tmed10
Met II	TMM43_RAT	Q5XIP9	Transmembrane protein 43 (Protein LUMA)	Tmem43
Met II	TMX2_RAT	Q5XIK2	Thioredoxin-related transmembrane protein 2	Tmx2
Met II	TOIP1_RAT	Q5PQX1	Torsin-1A-interacting protein 1	Tor1aip1
Met II	TOM22_RAT	Q75Q41	Mitochondrial import receptor subunit TOM22 homolog	Tomm22

Met I	TPIS_RAT	P48500	Triosephosphate isomerase	Tpi1
Met I; Met II	TPM3_RAT	Q63610	Tropomyosin alpha-3	Tpm3
Met I; Met II	TPM4_RAT	P09495	Tropomyosin alpha-4	Tpm4
Met II	TRAM1_RAT	Q5XI41	Translocating chain-associated membrane protein 1	Tram1
Met I; Met II	TRPV2_RAT	Q9WUD2	Transient receptor potential cation channel subfamily V member 2 (TRPV2)	Trpv2
Met I; Met II	TRY1_RAT	P00762	Anionic trypsin-1	Prss1
Met I	TSH3_RAT	D3ZKB9	Teashirt homolog 3	Tshz3
Met I	TXNL1_RAT	Q920J4	Thioredoxin-like protein 1	Txnl1
Met II	TXTP_RAT	P32089	Citrate transport protein	Slc25a1
Met I	TYB4_RAT	P62329	Thymosin beta-4	Tmsb4x
Met I	UBA1_RAT	Q5U300	Ubiquitin-like modifier-activating enzyme 1	Uba1
Met I	UBE2N_RAT	Q9EQX9	Ubiquitin-conjugating enzyme E2 N	Ube2n
Met II	UBXN4_RAT	Q5HZY0	Erasin	Ubxn4
Met II	UCRI_RAT	P20788	Cytochrome b-c1 complex subunit Rieske	Uqcrfs1
Met II	UFL1_RAT	B2GV24	E3 UFM1-protein ligase 1	Ufl1
Met I; Met II	VAMP3_RAT	P63025	VAMP3	Vamp3
Met II	VAMP5_RAT	Q9Z2J5	VAMP5	Vamp5
Met II	VAMP7_RAT	Q9JHW5	VAMP7	Vamp7
Met II	VAPA_RAT	Q9Z270	VAMP-associated protein A	Vapa
Met II	VAPB_RAT	Q9Z269	VAMP-associated protein B	Vapb
Met I	VAT1_RAT	Q3MIE4	Synaptic vesicle membrane protein VAT-1 homolog	Vat1
Met I; Met II	VATB2_RAT	P62815	V-type proton ATPase subunit B	Atp6v1b2
Met II	VATE1_RAT	Q6PCU2	V-type proton ATPase subunit E 1	Atp6v1e1
Met II	VATF_RAT	P50408	V-type proton ATPase subunit F	Atp6v1f
Met I; Met II	VDAC1_RAT	Q9Z2L0	Voltage-dependent anion-selective channel protein 1	Vdac1
Met I; Met II	VDAC2_RAT	P81155	Voltage-dependent anion-selective channel protein 2	Vdac2
Met II	VDAC3_RAT	Q9R1Z0	Voltage-dependent anion-selective channel protein 3	Vdac3
Met II	VEZA_RAT	Q5XI52	Vezatin	Vezt
Met II	VGFR2_RAT	O08775	Vascular endothelial growth factor receptor 2	Kdr
Met I	VIME_RAT	P31000	Vimentin	Vim
Met II	VKOR1_RAT	Q6TEK4	Vitamin K epoxide reductase complex subunit 1	Vkorc1
Met II	VMAT2_RAT	Q01827	Vesicular amine transporter 2	Slc18a2
Met II	VMP1_RAT	Q91ZQ0	Vacuole membrane protein 1	Vmp1
Met I; Met II	VWA5A_RAT	Q75WE7	Von Willebrand factor A domain-containing protein 5A	Vwa5a
Met I	YBOX3_RAT	Q62764	Y-box-binding protein 3	Ybx3
Met II	YES_RAT	F1LM93	Tyrosine-protein kinase Yes	Yes1
Met II	YIF1B_RAT	Q6PEC3	Protein YIF1B	Yif1b
Met II	YIPF4_RAT	Q5M7T4	Protein YIPF4	Yipf4

Met II	YIPF5_RAT	Q5XID0	Protein YIPF5	Yipf5
Met I; Met II	YMEL1_RAT	Q925S8	ATP-dependent zinc metalloprotease YME1L1	Yme1l1
Met II	ZNT1_RAT	Q62720	Zinc transporter 1	Slc30a1
Met II	ZW10_RAT	Q4V8C2	Centromere/kinetochore protein zw10 homolog	Zw10

Header line description of worksheets	
**Method**	Protein identification with which Method
**Protein ID**	Protein identification of UniProt
**Protein accession**	Protein accession number of UniProt
**Protein description**	Protein functional description
**Gene name**	Indicates the name of the gene that code for the protein sequence
**MW [kDa]**	Protein molecular weight ,unit [kDa]
**Score AVG**	A simple rule to be used to judge whether a result is significant or not
**Peptides AVG**	Number of peptides whitch spectrum hit
