# Supplementary material for: Proteomic Analysis of Lipid Rafts from RBL-2H3 Mast Cells
Source: Int J Mol Sci. 2019 Aug 11;20(16):3904. doi: 10.3390/ijms20163904 (PMC6720779; doi:10.3390/ijms20163904)
Supplement: Supplementary file 1 [file ijms-20-03904-s001.zip › Table S4.pdf]

Supplementary Table 4. Mast cell lipid raft proteins absent from RafProtV2 database

Method	Protein ID	Protein accession	Protein description	Gene name
Met II	AL5AP_RAT	P20291	Arachidonate 5-lipoxygenase-activating protein	Alox5ap
Met I	ASNS_RAT	P49088	Glutamine-dependent asparagine synthetase	Asns
Met II	B1WC50_RAT	B1WC50	RNA-binding protein EWS-like	Ewsr1
Met II	B4GN1_RAT	Q10468	Beta-1,4 N-acetylgalactosaminyltransferase 1	B4galnt1
Met I	B5DEN5_RAT	B5DEN5	Eukaryotic translation elongation factor 1 beta 2	Eef1b2
Met II	B5DEZ3_RAT	B5DEZ3	Coiled-coil domain containing 126	Ccdc126
Met II	B5DFJ2_RAT	B5DFJ2	Protein FAM3A	Fam3a
Met II	C1GLC_RAT	Q499P3	C1GALT1-specific chaperone 1	C1galt1c1
Met II	CAH8_RAT	Q5PPN4	Carbonic anhydrase-related protein	Ca8
Met II	CBPA3_RAT	P21961	Mast cell carboxypeptidase A	Cpa3
Met II	CHPT1_RAT	Q66H21	Cholinephosphotransferase 1	Chpt1
Met II	CL2DB_RAT	Q0H8B9	C-type lectin domain family 2 member D11	Clec2d11
Met II	CMA1_RAT	P50339	Chymase 1	Cma1
Met I	CNBP_RAT	P62634	Cellular nucleic acid-binding protein	Cnbp
Met II	COPD_RAT	Q66H80	Coatomer subunit delta	Arcn1
Met II	D3ZF97_RAT	D3ZF97	Endoplasmic reticulum lectin 1	Erlec1
Met II	D3ZGV2_RAT	D3ZGV2	Peroxisomal membrane protein PEX16	Pex16
Met II	D3ZMM8_RAT	D3ZMM8	Solute carrier family 7 member 6	Slc7a6
Met I	D3ZNQ6_RAT	D3ZNQ6	NEDD8-conjugating enzyme Ubc12	Ube2m
Met II	D3ZNS8_RAT	D3ZNS8	Aladin	Aaas
Met II	D3ZQG9_RAT	D3ZQG9	RT1 class I locus CE4	RT1-CE4
Met II	D3ZSY5_RAT	D3ZSY5	keratin 84	Krt84
Met II	D3ZU83_RAT	D3ZU83	Endoplasmic reticulum-Golgi intermediate compartment 3	Ergic3
Met II	D3ZUQ7_RAT	D3ZUQ7	Pantothenate kinase 3	Pank3
Met II	D3ZWZ1_RAT	D3ZWZ1	Integrin alpha X	Itgax
Met II	D3ZYM4_RAT	D3ZYM4	Macrophage stimulating 1 receptor	Mst1r
Met I	D4A3S8_RAT	D4A3S8	NOL1/NOP2/Sun domain family member 2	Nsun2
Met II	D4A533_RAT	D4A533	Transmembrane anterior posterior transformation protein 1	Tapt1
Met I	D4A6A2_RAT	D4A6A2	Heterogeneous nuclear ribonucleoprotein A3-like	LOC100911361
Met I	D4A720_RAT	D4A720	Serine/arginine-rich splicing factor 7 isoform 2	Srsf7
Met I	D4A7U6_RAT	D4A7U6	U6 snRNA-associated Sm-like protein LSm3	Lsm3
Met I; Met II	D4A9X7_RAT	D4A9X7	Hypermethylated in cancer 2 protein	Hic2

Met I	D4ADD7_RAT	D4ADD7	Glutaredoxin 5	Glrx5
Met I	DOPD_RAT	P80254	D-dopachrome decarboxylase	Ddt
Met II	E2AK3_RAT	Q9Z1Z1	Eukaryotic translation initiation factor 2-alpha kinase 3	Eif2ak3
Met II	E9PT10_RAT	Q861Q1	MHC class I antigen	RT1-CE4
Met II	E9PU77_RAT	E9PU77	Synaptotagmin-like protein 3	Sytl3
Met I	E9PU78_RAT	E9PU78	Isoform Bcl-X(S) of Bcl-2-like protein 1	---
Met I	F1LM33_RAT	F1LM33	Leucine-rich PPR motif-containing protein mitochondrial isoform	Lrpprc
Met II	F1LMK0_RAT	F1LMK0	A-kinase anchoring protein 1 isoform 2	Akap1
Met II	F1LRV9_RAT	F1LRV9	Myosin heavy chain 1	Myh1
Met II	F1LT70_RAT	F1LT70	Pantothenate kinase 2	Pank2
Met II	F1M8F6_RAT	F1M8F6	Myosin heavy chain 8	Myh8
Met II	F1MAS0_RAT	F1MAS0	keratin 32	Krt32
Met II	F6T9E7_RAT	F6T9E7	P-selectin glycoprotein ligand 1	Selplg
Met II	F7EP67_RAT	Q6IN10	Endothelin-converting enzyme 1 Isoform	Ece1
Met I	F7FP91_RAT	Q95II0	MHC class Ib antigen	RT1-CE5
Met II	FADS2_RAT	Q9Z122	Acyl-CoA 6-desaturase	Fads2
Met I; Met II	FCERB_RAT	P13386	High affinity immunoglobulin epsilon receptor subunit beta	Ms4a2
Met II	FGOP2_RAT	Q6TA25	FGFR1 oncogene partner 2 homolog	Fgfr1op2
Met II	G3V608_RAT	G3V608	Interleukin-3 receptor subunit beta	Csf2rb
Met II	G3V637_RAT	G3V637	Syntaxin-binding protein 2, isoform	Stxbp2
Met II	G3V6E1_RAT	G3V6E1	Myosin heavy chain 2	Myh2
Met I	G3V6G5_RAT	G3V6G5	F-box-like/WD repeat-containing protein TBL1X	Tbl1x
Met II	G3V712_RAT	G3V712	keratin 7	Krt7
Met II	G3V7A5_RAT	G3V7A5	Low density lipoprotein receptor isoform	Ldlr
Met II	G3V7L8_RAT	G3V7L8	V-type proton ATPase subunit E 1 isoform	Atp6v1e1
Met II	G3V7M2_RAT	G3V7M2	Integrin beta 7	Itgb7
Met II	G3V8I4_RAT	G3V8I4	Syntaxin-4A	Stx4
Met I; Met II	G3V908_RAT	G3V908	keratin, type II Kb15	Kb15
Met I; Met II	G3V936_RAT	G3V936	Citrate synthase	Cs
Met I	G3V993_RAT	G3V993	SAM domain, SH3 domain and nuclear localization signals, 1 (SAMNS1)	Samsn1
Met I	HMCS1_RAT	P17425	Hydroxymethylglutaryl-CoA synthase cytoplasmic	Hmgcs1
Met II	HRG1_RAT	B0BNL4	Heme transporter HRG1	Slc48a1
Met I	ICK_RAT	Q62726	Serine/threonine-protein kinase ICK	Ick
Met II	IRK2_RAT	Q64273	Inward rectifier potassium channel 2	Kcnj2
Met II	ITAD_RAT	Q9QYE7	Integrin alpha D	Itgad
Met I	M0R866_RAT	M0R866	Uncharacterized protein	---
Met II	M0RCA3_RAT	M0RCA3	Zinc finger protein-like 1	Zfpl1

Met I	M0RDJ4_RAT	M0RDJ4	Glia maturation factor beta	Gmfb
Met II	NCUG1_RAT	Q68FV6	Glycosylated lysosomal membrane protein	Glmp
Met II	NDUF5_RAT	B2GV71	NADH dehydrogenase [ubiquinone] 1 alpha subcomplex assembly factor 5	Ndufaf5
Met I	NQO1_RAT	P05982	NAD(P)H dehydrogenase [quinone] 1	Nqo1
Met II	PCAT2_RAT	P0C1Q3	Lysophosphatidylcholine acyltransferase 2	Lpcat2
Met II	PDE3B_RAT	Q63085	Cyclic GMP-inhibited phosphodiesterase B	Pde3b
Met II	PHOCN_RAT	Q9QYW3	MOB-like protein phocein	Mob4
Met II	PORED_RAT	Q5RJM1	3-oxo-5-alpha-steroid 4-dehydrogenase 3	Srd5a3
Met I	Q4G002_RAT	Q4G002	Histocompatibility 2 T region locus 24	RT1-T24-4
Met II	Q5FVC4_RAT	Q5FVC4	DnaJ (Hsp40) homolog subfamily B member 12	Dnajb12
Met II	Q5RK10_RAT	Q5RK10	60S ribosomal protein L13a, isoform	Rpl13a
Met I; Met II	Q63116_RAT	Q63116	Mast/stem cell growth factor receptor	Kit
Met II	Q63483_RAT	Q63483	Ras-related protein Rab-38	Rab38
Met II	Q642E3_RAT	Q642E3	CDK5 regulatory subunit-associated protein 3, isoform	Cdk5rap3
Met I	Q7TP15_RAT	Q7TP15	S-methyl-5'-thioadenosine phosphorylase	Mtap
Met II	Q7TQ86_RAT	Q7TQ86	Ac1158	Rsl1d1
Met I	R9PXU4_RAT	R9PXU4	Thioredoxin reductase 1 cytoplasmic	Txnrd1
Met II	RAB43_RAT	Q53B90	Ras-related protein Rab-43	Rab43
Met II	SIA4C_RAT	P61131	Beta-galactoside alpha-2,3-sialyltransferase 4	St3gal4
Met II	SO4A1_RAT	Q99N01	Organic anion-transporting polypeptide E	Slco4a1
Met II	STRN3_RAT	P58405	Striatin-3	Strn3
Met II	TM7S3_RAT	Q5FVF4	Transmembrane 7 superfamily member 3	Tm7sf3
Met I; Met II	TRY1_RAT	P00762	Anionic trypsin-1	Prss1
Met II	VEZA_RAT	Q5XI52	Vezatin	Vezt
Met II	VGFR2_RAT	O08775	Vascular endothelial growth factor receptor 2	Kdr

Header line description of worksheet "RafProtV2 Analysis"	
**Method**	Protein identification by which Method
**Protein ID**	Protein identification of UniProt
**Protein accession**	Protein accession number of UniProt
**Protein description**	Protein functional description
**Gene name**	Indicates the name of the gene that code for the protein sequence
