# Supplementary material for: Proteomic Analysis of Lipid Rafts from RBL-2H3 Mast Cells
Source: Int J Mol Sci. 2019 Aug 11;20(16):3904. doi: 10.3390/ijms20163904 (PMC6720779; doi:10.3390/ijms20163904)
Supplement: Supplementary file 1 [file ijms-20-03904-s001.zip › Table S6.pdf]

Supplementary Table 6. Enriched GO terms from mast cell lipid raft proteome analysis using DAVID Bioinformatic Resources

Annotation Cluster Enrichment Score: 150.54					

ID Term	GO Term	Fold Enriched	Count	*p* -value	FDR
GO:1903561	EXTRACELLULAR VESICLE	3.60	487	6.97E-169	1.08E-165
Annotated proteins					

Protein Accession	Protein Identification
D3ZAF6	ATP synthase subunit F mitochondrial
Q6P503	V-type proton ATPase subunit D
B5DEH2	Erlin-2
F1M2P8	Protein kinase C alpha
P50408	V-type proton ATPase subunit F
Q63716	Peroxiredoxin-1
P26772	10 kDa heat shock protein mitochondrial
Q5U204	Ragulator complex protein LAMTOR3
P0DP29	Calmodulin-1
G3V852	Talin-1
D3ZTX0	Transmembrane emp24 domain-containing protein 7
P50503	Hsc70-interacting protein
P06686	Sodium/potassium-transporting ATPase subunit alpha-2
P06687	Sodium/potassium-transporting ATPase subunit alpha-3
O08623	Sequestosome-1
P06685	Sodium/potassium-transporting ATPase subunit alpha-1
P07632	Superoxide dismutase [Cu-Zn]
Q66H18	Synaptophysin-like 1
G3V6H0	Ras-related protein Rab-1b
P27139	Carbonic anhydrase 2
Q7TP15	S-methyl-5'-thioadenosine phosphorylase
Q9ES40	PRA1 family protein 3
P61589	Transforming protein RhoA
G3V7U6	Calpain 5
P68511	14-3-3 protein eta
Q01205	2-oxoglutarate dehydrogenase complex component E2
P67779	Prohibitin
Q5FVF4	Transmembrane 7 superfamily member 3
Q5BJY9	Keratin, type I cytoskeletal 18
F1M5Z5	Anoctamin-6

D3ZRN3	Beta-actin-like protein 2
O08651	D-3-phosphoglycerate dehydrogenase
O88989	Malate dehydrogenase cytoplasmic
P11762	Galectin-1
P27615	Lysosome membrane protein 2 (CD36)
Q6AYK6	Calcyclin-binding protein
P06761	Endoplasmic reticulum chaperone BiP
Q9QYV0	Disintegrin and metalloproteinase domain-containing protein 15
P63102	14-3-3 protein zeta/delta
B0K020	CDGSH iron-sulfur domain-containing protein 1
Q9EST6	Acidic leucine-rich nuclear phosphoprotein 32 family member B
Q78P65	Rap2A-like protein
P07943	Aldose reductase
P32089	Citrate transport protein
M0RCF3	Integrin beta 3
Q5XI34	Serine/threonine-protein phosphatase 2A 65 kDa regulatory subunit A alpha isoform
Q5XI32	F-actin-capping protein subunit beta
P83732	60S ribosomal protein L24
Q5XIV1	Phosphoglycerate kinase 2
P52555	Endoplasmic reticulum resident protein 29
O88994	Mitochondrial amidoxime reducing component 2
Q5XI38	Lymphocyte cytosolic protein 1
P04764	Alpha-enolase
Q5XI36	CD97
B0K010	Thioredoxin domain-containing 17
Q10758	Keratin, type II cytoskeletal 8
Q07936	Annexin A2
P05197	Elongation factor 2
Q9R1Z0	Voltage-dependent anion-selective channel protein 3
P85968	6-phosphogluconate dehydrogenase decarboxylating
P63322	Ras-related protein Ral-A
Q63396	Activated RNA polymerase II transcriptional coactivator p15
P54313	Transducin beta-2
Q6AYQ9	Peptidyl-prolyl cis-trans isomerase C
F1MA89	Cyclin Y
P54311	Transducin beta-1
Q6PDU1	Serine/arginine-rich splicing factor 2
Q6AYQ4	Transmembrane protein 109
O70257	Syntaxin-7

O88767	Protein/nucleic acid deglycase DJ-1
D4AEP0	Adenylosuccinate synthetase isozyme 2
P51577	P2X purinoceptor 4
D3ZUX5	MICOS complex subunit MIC-19
P35213	14-3-3 protein beta/alpha
P34058	Heat shock protein HSP 90-beta
Q9EQX9	Ubiquitin-conjugating enzyme E2 N
G3V7P1	Syntaxin-12
P15865	Histone H1.4
Q91ZN1	Coronin-1A
P04785	Protein disulfide-isomerase
G3V9Y2	Phospholipid phosphatase 1
G3V7Q7	Ras GTPase-activating-like protein IQGAP1
Q63016	Large neutral amino acids transporter small subunit 1
Q5EAP4	Guanine nucleotide binding protein alpha-14
R9PXR4	Mitochondrial import receptor subunit TOM70
P63331	Serine/threonine-protein phosphatase 2A catalytic subunit alpha isoform
P85971	6-phosphogluconolactonase
D3ZSY5	keratin 84
Q64244	ADP-ribosyl cyclase/cyclic ADP-ribose hydrolase 1
P21531	60S ribosomal protein L3
P81155	Voltage-dependent anion-selective channel protein 2
P31000	Vimentin
P12368	cAMP-dependent protein kinase type II-alpha regulatory subunit
F7EPH4	Inorganic Pyrophosphatase
Q5I0E7	Transmembrane emp24 domain-containing protein 9
A1L1J8	Ras-related protein Rab-5b
P38983	40S ribosomal protein SA
B0K031	60S ribosomal protein L7, isoform
F1LN88	Aldehyde dehydrogenase
F7FFV2	keratin 5
Q05982	Nucleoside diphosphate kinase A
P21708	Mitogen-activated protein kinase 3
P04797	Glyceraldehyde-3-phosphate dehydrogenase
O35567	Bifunctional purine biosynthesis protein PURH
P04897	Guanine nucleotide-binding protein G(i) subunit alpha-2
Q8VHF5	Citrate synthase mitochondrial
G3V936	Citrate synthase
Q08849	Syntaxin-3

Q8CFN2	Cell division control protein 42 homolog (CDC42)
Q9EPV5	Apoptotic protease-activating factor 1
Q5I0D1	Glyoxalase domain-containing protein 4
Q53B90	Ras-related protein Rab-43
P58195	Phospholipid scramblase 1
Q9JHW5	VAMP7
Q9JI85	Nucleobindin-2
P01830	Thy-1 membrane glycoprotein
O70377	Synaptosomal-associated protein 23 (SNAP-23)
Q68FP1	Gelsolin
D4A133	V-type proton ATPase catalytic subunit A
P21913	Succinate dehydrogenase [ubiquinone] iron-sulfur subunit mitochondrial
Q07014	Tyrosine-protein kinase Lyn
D3ZK56	Ras-related protein Rap-2c
Q07984	Translocon-associated protein subunit delta
Q5XI77	Annexin A11
P31977	Ezrin
Q5XI73	Rho GDP dissociation inhibitor 1
P07151	Beta-2-microglobulin
P15999	ATP synthase subunit alpha mitochondrial
P07150	Annexin A1
Q6AYQ1	Golgin subfamily A member 7
P60711	Actin cytoplasmic 1
O35353	Transducin beta-4
B2RYB8	Integrin beta 2
F1LW77	Ras-related protein Rab-33b
Q9Z2S9	Flotillin-2
Q5U362	Annexin A4
Q68FR6	Elongation factor 1-gamma
Q9Z2L0	Voltage-dependent anion-selective channel protein 1
B2RYC9	Glucosylceramidase
P51635	Alcohol dehydrogenase [NADP(+)]
E9PTU4	Myosin heavy chain 11
F1LM82	Heterogeneous nuclear ribonucleoproteins A2/B1
P11442	Clathrin heavy chain 1
B0BN81	Ribosomal protein S5
P12075	Cytochrome c oxidase subunit 5B mitochondrial
Q05030	Platelet-derived growth factor receptor beta
G3V7L8	V-type proton ATPase subunit E 1 isoform

Q62662	Tyrosine-protein kinase FRK
P61107	Ras-related protein Rab-14
F1LPV0	Asparagine--tRNA ligase, cytoplasmic
P61972	Nuclear transport factor 2
Q6PCU2	V-type proton ATPase subunit E 1
Q5PQL7	Integral membrane protein 2C
M0R9L0	Nascent polypeptide-associated complex subunit alpha
P49134	Integrin beta 1
P50475	Alanine--tRNA ligase cytoplasmic
G3V712	keratin 7
Q68FQ0	T-complex protein 1 epsilon
Q810F4	Protein FAM3C
P31044	Phosphatidylethanolamine-binding protein 1
Q4QQV0	Tubulin beta-6
F1LQ81	Vesicle-fusing ATPase
Q3B7V5	Ras-related protein Rab-2b
D4A0T0	NADH dehydrogenase 1 beta subcomplex subunit 10
G3V7M2	Integrin beta 7
G3V6P8	Guanine nucleotide-binding protein gamma-12
G3V6P7	Myosin heavy chain 9
Q04970	GTPase NRas
D3ZNG3	Signal peptide peptidase-like 2A
Q9Z1J7	Amino acid transporter
E9PTW1	Secretory carrier-associated membrane protein
R9PXU4	Thioredoxin reductase 1 cytoplasmic
D4ADE8	ATP-dependent RNA helicase DDX3X
O88600	Heat shock 70 kDa protein 4
G3V9N9	alpha-1,2-Mannosidase
Q794F9	4F2 cell-surface antigen heavy chain
Q6P791	Ragulator complex protein LAMTOR1
F1LNT8	VAMP8
P61227	Ras-related protein Rap-2b
Q6P799	Serine--tRNA ligase cytoplasmic
P62703	40S ribosomal protein S4, X isoform
P62909	40S ribosomal protein S3
Q6AYC4	Macrophage-capping protein
Q63377	Sodium/potassium-transporting ATPase subunit beta-3
P62907	60S ribosomal protein L10a
M0RBJ0	Guanine nucleotide-binding protein gamma-2

Q63942	Ras-related protein Rab-3d
P48721	Mortalin
Q63584	Transmembrane emp24 domain-containing protein 10
P10960	Prosaposin
Q6EV76	Alpha-(1,6)-fucosyltransferase
Q5XIM9	T-complex protein 1 beta
F1M779	Clathrin heavy chain
P05065	Fructose-bisphosphate aldolase A
P32551	Cytochrome b-c1 complex subunit 2 mitochondrial
B0BN52	Mitochondrial carrier homolog 2
E9PTX9	Solute carrier family 12 member 2
Q5XFX0	Transgelin-2
Q7TSA0	Mitochondrial Rho GTPase 2
P61805	Dolichyl-diphosphooligosaccharide protein glycosyltransferase subunit DAD1
G3V908	keratin, type II Kb15
P09811	Glycogen phosphorylase liver form
P53987	Monocarboxylate transporter 1
G3V6N2	Transmembrane emp24 domain-containing protein 4
Q62902	Lectin mannose-binding 1
P28494	Alpha-mannosidase 2
P61131	Beta-galactoside alpha-2,3-sialyltransferase 4
Q6PDV6	40S ribosomal protein S14
P50399	Rab GDP dissociation inhibitor beta
Q6AYD3	Proliferation-associated protein 2G4
Q9WVB1	Ras-related protein Rab-6a
Q05175	Brain acid soluble protein 1
B2RYG6	Ubiquitin thioesterase OTUB1
P62278	40S ribosomal protein S13
Q3T1K5	F-actin-capping protein subunit alpha-2
P62138	Serine/threonine-protein phosphatase PP1-alpha catalytic subunit
P68370	Tubulin alpha-1A
Q5U316	Ras-related protein Rab-35
P11167	Solute carrier family 2, facilitated glucose transporter member 1
P31399	ATP synthase subunit D mitochondrial
D4A720	Serine/arginine-rich splicing factor 7 isoform 2
G3V8L7	Integrin alpha M
P04642	L-lactate dehydrogenase A chain
O35509	Ras-related protein Rab-11b
P62828	GTP-binding nuclear protein Ran

P70583	Deoxyuridine 5'-triphosphate nucleotidohydrolase
Q6AYI1	Probable ATP-dependent RNA helicase DDX5
P70580	Membrane-associated progesterone receptor component 1
P23358	60S ribosomal protein L12
Q3T1J1	Eukaryotic translation initiation factor 5A-1
F7EWC1	Vasodilator-stimulated phosphoprotein
Q66HA6	ADP-ribosylation factor-like protein 8B
P11598	Protein disulfide-isomerase A3
P62282	40S ribosomal protein S11
Q6P3V9	60S ribosomal protein L4
G3V6T1	Coatomer subunit alpha
Q6P3V8	Eukaryotic initiation factor 4A-I
Q66HA4	Tax1-binding protein 1 homolog
F1LP82	Ras-related protein Rab-2a
Q62636	Ras-related protein Rap-1b
Q3MIE4	Synaptic vesicle membrane protein VAT-1 homolog
Q6P742	Proteolipid protein 2
P04636	Malate dehydrogenase mitochondrial
P62914	60S ribosomal protein L11
Q5U300	Ubiquitin-like modifier-activating enzyme 1
G3V7I0	Peroxiredoxin-3
P62815	V-type proton ATPase subunit B
Q9JID2	Guanine nucleotide-binding protein alpha-11
Q63198	Contactin 1
P11980	Pyruvate kinase PKM
Q9Z2J5	VAMP5
P10719	ATP synthase subunit beta mitochondrial
D3ZAY7	Band 4.1-like protein 2
G3V741	Solute carrier family 25 member 3
O88941	Mannosyl-oligosaccharide glucosidase
O54980	Synaptogyrin-2
Q6P6T6	Cathepsin D
Q4V7D9	Acid sphingomyelinase-like phosphodiesterase 3B
Q6S395	Plectin
F1LMP7	Granulins
Q5XI04	Erythrocyte band 7 integral membrane protein
F1LQJ7	Phosphoenolpyruvate carboxykinase [GTP], mitochondrial
Q9R1T1	Barrier-to-autointegration factor
P22734	Catechol O-methyltransferase

P62804	Histone H4
F1LM93	Tyrosine-protein kinase Yes
Q63083	Nucleobindin-1
Q9JKB7	Guanine deaminase
P12928	Pyruvate kinase PKLR
Q63081	Protein disulfide-isomerase A6
G3V9S3	Tyrosine-protein phosphatase non-receptor type 13
P13832	Myosin regulatory light chain RLC-A
B1WBY5	DnaJ homolog subfamily C member 11
Q62940	E3 ubiquitin-protein ligase NEDD4
Q62658	Peptidyl-prolyl cis-trans isomerase FKBP1A
P68255	14-3-3 protein theta
Q63803	Guanine nucleotide-binding protein G(s) subunit alpha isoforms XLas
Q6P6U0	Tyrosine-protein kinase Fgr
Q62753	Syntaxin-binding protein 2
D4A5K6	Zinc metallopeptidase STE24
P13838	Leukosialin
Q06647	ATP synthase subunit O mitochondrial
G3V637	Syntaxin-binding protein 2, isoform
P62859	40S ribosomal protein S28
Q6IN24	Galectin-8
G3V8I4	Syntaxin-4A
Q6IFV6	Keratin 35
P09895	60S ribosomal protein L5
Q63507	60S ribosomal protein L14
P10111	Peptidyl-prolyl cis-trans isomerase A
G3V679	Transferrin receptor protein 1
P62260	14-3-3 protein epsilon
Q5XIE8	Integral membrane protein 2B
B2GUZ9	Fam49b protein
Q6IG03	Keratin, type II cytoskeletal 73
Q6IG02	Keratin, type II cytoskeletal 2 epidermal
Q6IG01	Keratin, type II cytoskeletal 1B
Q9Z0V6	Thioredoxin-dependent peroxide reductase mitochondrial
Q3SWT0	Platelet endothelial cell adhesion molecule (Pecam1)
Q6IN10	Endothelin-converting enzyme 1 Isoform
Q6IG05	Keratin, type II cytoskeletal 75
Q6IFW6	Keratin, type I cytoskeletal 10
P09495	Tropomyosin alpha-4

Q5XIF6	Tubulin alpha-4A
D3ZWS0	Protein scribble homolog
P62963	Profilin-1
Q66HD0	Endoplasmin
P62250	40S ribosomal protein S16
Q91Y81	Septin-2
Q99P74	Ras-related protein Rab-27b
P70550	Ras-related protein Rab-8b
Q99P75	Ras-related protein Rab-9a
Q6IG12	Keratin, type II cytoskeletal 7
P35704	Peroxiredoxin-2
Q2I6B2	V-type proton ATPase subunit A1
G3V8G5	Golgi apparatus protein 1
P62630	Elongation factor 1-alpha 1
P62775	Myotrophin
Q6P6Q2	Keratin, type II cytoskeletal 5
P62246	40S ribosomal protein S15a
D3ZNQ6	NEDD8-conjugating enzyme Ubc12
P62832	60S ribosomal protein L23
P05712	Ras-related protein Rab-2a
P62836	Ras-related protein Rap-1a
P62243	40S ribosomal protein S8
E9PSV5	Phosphoserine aminotransferase
Q9JLT0	Myosin heavy chain 10
P82995	Heat shock protein HSP 90-alpha
P42667	SEC11 homolog A
G3V8F5	Mitochondrial import receptor subunit TOM40 homolog
Q6IN37	GM2 ganglioside activator
P80254	D-dopachrome decarboxylase
P13383	Nucleolin
Q63610	Tropomyosin alpha-3
Q66X93	Staphylococcal nuclease domain-containing protein 1
Q6IFZ5	Keratin, type II cytoskeletal 2 oral
Q5VLR5	Endoplasmic reticulum resident protein 44
P62986	Ubiquitin-60S ribosomal protein L40
Q6RUV5	Ras-related C3 botulinum toxin substrate 1 (Rac1)
P62982	Ubiquitin-40S ribosomal protein S27a
P82471	Guanine nucleotide-binding protein G(q) subunit alpha
B4F7C2	Tubulin beta-4A

E9PT65	Radixin
Q9EPH8	Polyadenylate-binding protein 1
Q6MG61	Chloride intracellular channel protein 1
P19944	60S acidic ribosomal protein P1
Q6TXF3	Acyl-CoA-binding protein
P19945	60S acidic ribosomal protein P0
B2RZ27	SH3 domain-binding glutamic acid-rich-like protein 3
B2RYQ2	Serine/threonine-protein phosphatase 2A activator
P11240	Cytochrome c oxidase subunit 5A mitochondrial
P11232	Thioredoxin
P05982	NAD(P)H dehydrogenase [quinone] 1
P10252	CD48 antigen
P69897	Tubulin beta-5
P62890	60S ribosomal protein L30
P63095	Guanine nucleotide-binding protein G(s) subunit alpha
P52296	Importin subunit beta-1
D3ZTJ0	Protein XRP2
Q4QRB4	Tubulin beta-3
Q6P9V9	Tubulin alpha-1B
B2GUV5	V-type proton ATPase subunit G 1
P18418	Calreticulin
Q6P9V1	CD81 molecule
P13221	Aspartate aminotransferase cytoplasmic
Q09073	ADP/ATP translocase 2
Q6AYA5	Transmembrane protein 106 B
P54921	Alpha-soluble NSF attachment protein (SNAP-alpha)
P25113	Phosphoglycerate mutase 1
Q3KRD8	Eukaryotic translation initiation factor 6
G3V8C4	Chloride intracellular channel protein 4
P61983	14-3-3 protein gamma
P49242	40S ribosomal protein S3a
B0BNA5	Coactosin-like protein
P02401	60S acidic ribosomal protein P2
P50137	Transketolase
P09527	Ras-related protein Rab-7a
P10888	Cytochrome c oxidase subunit 4 isoform 1 mitochondrial
Q66HG5	Transmembrane 9 superfamily member 2
Q5U3Z7	Serine hydroxymethyltransferase
P48500	Triosephosphate isomerase

P23640	Ras-related protein Rab-27a
P62747	Rho-related GTP-binding protein RhoB
O70595	ATP-binding cassette sub-family B member 6
B2GV92	Prostaglandin E synthase 3
P62083	40S ribosomal protein S7
Q6IFU9	keratin 16
Q6P9T8	Tubulin beta-4B
Q6IFU8	Keratin, type I cytoskeletal 17
P04157	Receptor-type tyrosine-protein phosphatase C (CD45)
Q6IFV3	Keratin, type I cytoskeletal 15
Q6IFV1	Keratin, type I cytoskeletal 14
P20070	NADH-cytochrome b5 reductase 3
Q5XIH7	Prohibitin 2
G3V8A5	Vacuolar protein sorting-associated protein 35
P11348	Dihydropteridine reductase
D4A465	Ragulator complex protein LAMTOR2
Q5I0G4	Glycine--tRNA ligase
Q5U1Y2	Ras-related C3 botulinum toxin substrate 2 (Rac2)
P85007	Immediate early response 3-interacting protein 1
Q00238	Intercellular adhesion molecule 1 (ICAM1)
Q5M843	2-oxoglutarate and iron-dependent oxygenase domain-containing protein 3
Q5EB77	Ras-related protein Rab-18
Q6AY18	GTP-binding protein SAR1a
D3ZIP8	Endonuclease domain containing 1
P19804	Nucleoside diphosphate kinase B
D4ADS9	EFR3 homolog A
B2GVB9	Fermt3 protein
B0BNG3	Vesicular integral-membrane protein VIP36
Q6AXT5	Ras-related protein Rab-21
Q6NYB7	Ras-related protein Rab-1a
D3ZPR0	Exportin-2
P19511	ATP synthase F(0) complex subunit B1 mitochondrial
G3V8Z1	Linker for activation of T-cells family member 2 (NTAL)
Q76GL9	Neutral amino acid transporter A
F1LNF0	Myosin heavy chain 14
P17074	40S ribosomal protein S19
Q5M7T6	V-type proton ATPase subunit D 1
P63245	Receptor of activated protein C kinase 1 (RACK1)
Q8SEZ5	Cytochrome c oxidase subunit 2

Q4KM73	UMP-CMP kinase
A1L134	Ancient ubiquitous protein 1
P46462	Transitional endoplasmic reticulum ATPase
P35171	Cytochrome c oxidase subunit 7A2
P04961	Proliferating cell nuclear antigen
Q32PX6	Rho-related GTP-binding protein RhoG
Q3KRC3	Signal recognition particle receptor alpha
Q6AXS5	Plasminogen activator inhibitor 1 RNA-binding protein
Q6AXS4	Renin receptor
P05426	60S ribosomal protein L7
D4A4T0	STIP1 homology and U box-containing protein 1
D4A0E2	NSF attachment protein gamma
P16617	Phosphoglycerate kinase 1
P45592	Cofilin-1
Q01621	Tyrosine-protein kinase LCK
F1LZX9	Integrin alpha 5
F1MAS0	keratin 32
Q9JJ19	Na(+)/H(+) exchange regulatory cofactor NHE-RF1
P08753	Guanine nucleotide-binding protein G(k) subunit alpha
D3ZPP2	ADP-ribosylation factor-like protein 8A
P63269	Actin gamma-enteric smooth muscle
P15429	Beta-enolase
Q63692	Hsp90 co-chaperone Cdc37
P97675	Ectonucleotide pyrophosphatase/phosphodiesterase family member 3
Q5XIA8	Growth hormone-inducible transmembrane protein
Q5M7W6	Protein FAM234A
D4A9L2	Serine/arginine-rich splicing factor 1
P35289	Ras-related protein Rab-15
D3ZZR9	Peptidylprolyl isomerase
P63018	Heat shock cognate 71 kDa protein
P63012	Ras-related protein Rab-3a
P35565	Calnexin
Q9R0C5	Polypeptide N-acetylgalactosaminyltransferase 7
B0BNK1	Ras-related protein Rab-5c
P17046	Lysosome-associated membrane glycoprotein 2 (Lamp2)
G3V6D3	ATP synthase subunit beta
D3ZDR2	Charged multivesicular body protein 6
E9PTI1	V-type proton ATPase subunit H
P85108	Tubulin beta-2A

D3ZZS8	V-type proton ATPase subunit B, kidney isoform
P35435	ATP synthase subunit gamma mitochondrial
D3ZGM7	Peptidase inhibitor 16
Q08163	Adenylyl cyclase-associated protein 1
P26453	Basigin
Q5RKJ9	Ras-related protein Rab-10
Q5M860	Rho GDP dissociation inhibitor 2
Q8CGU6	Nicastrin
P63039	60 kDa heat shock protein
P14659	Heat shock-related 70 kDa protein 2
Q6IRF8	V-type proton ATPase subunit S1
P00507	Aspartate aminotransferase mitochondrial
Q3MHS9	Chaperonin-containing TCP1 subunit 6A
M0RC99	Ras-related protein Rab-5a
Q9JK11	Reticulon-4
P14562	Lysosome-associated membrane glycoprotein 1 (Lamp1)
Q6AXV4	Sorting and assembly machinery component 50 homolog
P14668	Annexin A5
P49432	Pyruvate dehydrogenase component E1 beta
Q4G075	Leukocyte elastase inhibitor
Q9Z1E1	Flotillin-1
Q6IMF3	Keratin, type II cytoskeletal 1
Q920J4	Thioredoxin-like protein 1
P24368	Peptidyl-prolyl cis-trans isomerase B
Q499P3	C1GALT1-specific chaperone 1
B0BNJ1	Sorcin
P19627	Guanine nucleotide-binding protein G(z) subunit alpha
G3V9A3	14-3-3 protein sigma
P35280	Ras-related protein Rab-8a

ID Term	GO Term	Fold Enriched	Count	*p* -value	FDR
**GO:0070062**	**EXTRACELLULAR EXOSOME**	**3.60**	**485**	**2.38E-167**	**3.69E-64**
**Annotated proteins**					
**Protein Accession**	**Protein Identification**				
D3ZAF6	ATP synthase subunit F mitochondrial				
Q6P503	V-type proton ATPase subunit D				
B5DEH2	Erlin-2				

F1M2P8	Protein kinase C alpha
P50408	V-type proton ATPase subunit F
Q63716	Peroxiredoxin-1
P26772	10 kDa heat shock protein mitochondrial
Q5U204	Ragulator complex protein LAMTOR3
P0DP29	Calmodulin-1
G3V852	Talin-1
D3ZTX0	Transmembrane emp24 domain-containing protein 7
P50503	Hsc70-interacting protein
O08623	Sequestosome-1
P06685	Sodium/potassium-transporting ATPase subunit alpha-1
P07632	Superoxide dismutase [Cu-Zn]
Q66H18	Synaptophysin-like 1
G3V6H0	Ras-related protein Rab-1b
P27139	Carbonic anhydrase 2
Q7TP15	S-methyl-5'-thioadenosine phosphorylase
Q9ES40	PRA1 family protein 3
P61589	Transforming protein RhoA
G3V7U6	Calpain 5
P68511	14-3-3 protein eta
Q01205	2-oxoglutarate dehydrogenase complex component E2
P67779	Prohibitin
Q5FVF4	Transmembrane 7 superfamily member 3
Q5BJY9	Keratin, type I cytoskeletal 18
F1M5Z5	Anoctamin-6
D3ZRN3	Beta-actin-like protein 2
O08651	D-3-phosphoglycerate dehydrogenase
O88989	Malate dehydrogenase cytoplasmic
P11762	Galectin-1
P27615	Lysosome membrane protein 2 (CD36)
Q6AYK6	Calcyclin-binding protein
P06761	Endoplasmic reticulum chaperone BiP
Q9QYV0	Disintegrin and metalloproteinase domain-containing protein 15
P63102	14-3-3 protein zeta/delta
B0K020	CDGSH iron-sulfur domain-containing protein 1
Q9EST6	Acidic leucine-rich nuclear phosphoprotein 32 family member B
Q78P65	Rap2A-like protein
P07943	Aldose reductase
P32089	Citrate transport protein

M0RCF3	Integrin beta 3
Q5XI34	Serine/threonine-protein phosphatase 2A 65 kDa regulatory subunit A alpha isoform
Q5XI32	F-actin-capping protein subunit beta
P83732	60S ribosomal protein L24
Q5XIV1	Phosphoglycerate kinase 2
P52555	Endoplasmic reticulum resident protein 29
O88994	Mitochondrial amidoxime reducing component 2
Q5XI38	Lymphocyte cytosolic protein 1
P04764	Alpha-enolase
Q5XI36	CD97
B0K010	Thioredoxin domain-containing 17
Q10758	Keratin, type II cytoskeletal 8
Q07936	Annexin A2
P05197	Elongation factor 2
Q9R1Z0	Voltage-dependent anion-selective channel protein 3
P85968	6-phosphogluconate dehydrogenase decarboxylating
P63322	Ras-related protein Ral-A
Q63396	Activated RNA polymerase II transcriptional coactivator p15
P54313	Transducin beta-2
Q6AYQ9	Peptidyl-prolyl cis-trans isomerase C
F1MA89	Cyclin Y
P54311	Transducin beta-1
Q6PDU1	Serine/arginine-rich splicing factor 2
Q6AYQ4	Transmembrane protein 109
O70257	Syntaxin-7
O88767	Protein/nucleic acid deglycase DJ-1
D4AEP0	Adenylosuccinate synthetase isozyme 2
P51577	P2X purinoceptor 4
D3ZUX5	MICOS complex subunit MIC-19
P35213	14-3-3 protein beta/alpha
P34058	Heat shock protein HSP 90-beta
Q9EQX9	Ubiquitin-conjugating enzyme E2 N
G3V7P1	Syntaxin-12
P15865	Histone H1.4
Q91ZN1	Coronin-1A
P04785	Protein disulfide-isomerase
G3V9Y2	Phospholipid phosphatase 1
G3V7Q7	Ras GTPase-activating-like protein IQGAP1
Q63016	Large neutral amino acids transporter small subunit 1

Q5EAP4	Guanine nucleotide binding protein alpha-14
R9PXR4	Mitochondrial import receptor subunit TOM70
P63331	Serine/threonine-protein phosphatase 2A catalytic subunit alpha isoform
P85971	6-phosphogluconolactonase
D3ZSY5	keratin 84
Q64244	ADP-ribosyl cyclase/cyclic ADP-ribose hydrolase 1
P21531	60S ribosomal protein L3
P81155	Voltage-dependent anion-selective channel protein 2
P31000	Vimentin
P12368	cAMP-dependent protein kinase type II-alpha regulatory subunit
F7EPH4	Inorganic Pyrophosphatase
Q5I0E7	Transmembrane emp24 domain-containing protein 9
A1L1J8	Ras-related protein Rab-5b
P38983	40S ribosomal protein SA
B0K031	60S ribosomal protein L7, isoform
F1LN88	Aldehyde dehydrogenase
F7FFV2	keratin 5
Q05982	Nucleoside diphosphate kinase A
P21708	Mitogen-activated protein kinase 3
P04797	Glyceraldehyde-3-phosphate dehydrogenase
O35567	Bifunctional purine biosynthesis protein PURH
P04897	Guanine nucleotide-binding protein G(i) subunit alpha-2
Q8VHF5	Citrate synthase mitochondrial
G3V936	Citrate synthase
Q08849	Syntaxin-3
Q8CFN2	Cell division control protein 42 homolog (CDC42)
Q9EPV5	Apoptotic protease-activating factor 1
Q5I0D1	Glyoxalase domain-containing protein 4
Q53B90	Ras-related protein Rab-43
P58195	Phospholipid scramblase 1
Q9JHW5	VAMP7
Q9JI85	Nucleobindin-2
P01830	Thy-1 membrane glycoprotein
O70377	Synaptosomal-associated protein 23 (SNAP-23)
Q68FP1	Gelsolin
D4A133	V-type proton ATPase catalytic subunit A
P21913	Succinate dehydrogenase [ubiquinone] iron-sulfur subunit mitochondrial
Q07014	Tyrosine-protein kinase Lyn
D3ZK56	Ras-related protein Rap-2c

Q07984	Translocon-associated protein subunit delta
Q5XI77	Annexin A11
P31977	Ezrin
Q5XI73	Rho GDP dissociation inhibitor 1
P07151	Beta-2-microglobulin
P15999	ATP synthase subunit alpha mitochondrial
P07150	Annexin A1
Q6AYQ1	Golgin subfamily A member 7
P60711	Actin cytoplasmic 1
O35353	Transducin beta-4
B2RYB8	Integrin beta 2
F1LW77	Ras-related protein Rab-33b
Q9Z2S9	Flotillin-2
Q5U362	Annexin A4
Q68FR6	Elongation factor 1-gamma
Q9Z2L0	Voltage-dependent anion-selective channel protein 1
B2RYC9	Glucosylceramidase
P51635	Alcohol dehydrogenase [NADP(+)]
E9PTU4	Myosin heavy chain 11
F1LM82	Heterogeneous nuclear ribonucleoproteins A2/B1
P11442	Clathrin heavy chain 1
B0BN81	Ribosomal protein S5
P12075	Cytochrome c oxidase subunit 5B mitochondrial
Q05030	Platelet-derived growth factor receptor beta
G3V7L8	V-type proton ATPase subunit E 1 isoform
Q62662	Tyrosine-protein kinase FRK
P61107	Ras-related protein Rab-14
F1LPV0	Asparagine--tRNA ligase, cytoplasmic
P61972	Nuclear transport factor 2
Q6PCU2	V-type proton ATPase subunit E 1
Q5PQL7	Integral membrane protein 2C
M0R9L0	Nascent polypeptide-associated complex subunit alpha
P49134	Integrin beta 1
P50475	Alanine--tRNA ligase cytoplasmic
G3V712	keratin 7
Q68FQ0	T-complex protein 1 epsilon
Q810F4	Protein FAM3C
P31044	Phosphatidylethanolamine-binding protein 1
Q4QQV0	Tubulin beta-6

F1LQ81	Vesicle-fusing ATPase
Q3B7V5	Ras-related protein Rab-2b
D4A0T0	NADH dehydrogenase 1 beta subcomplex subunit 10
G3V7M2	Integrin beta 7
G3V6P8	Guanine nucleotide-binding protein gamma-12
G3V6P7	Myosin heavy chain 9
Q04970	GTPase NRas
D3ZNG3	Signal peptide peptidase-like 2A
Q9Z1J7	Amino acid transporter
E9PTW1	Secretory carrier-associated membrane protein
R9PXU4	Thioredoxin reductase 1 cytoplasmic
D4ADE8	ATP-dependent RNA helicase DDX3X
O88600	Heat shock 70 kDa protein 4
G3V9N9	alpha-1,2-Mannosidase
Q794F9	4F2 cell-surface antigen heavy chain
Q6P791	Ragulator complex protein LAMTOR1
F1LNT8	VAMP8
P61227	Ras-related protein Rap-2b
Q6P799	Serine--tRNA ligase cytoplasmic
P62703	40S ribosomal protein S4, X isoform
P62909	40S ribosomal protein S3
Q6AYC4	Macrophage-capping protein
Q63377	Sodium/potassium-transporting ATPase subunit beta-3
P62907	60S ribosomal protein L10a
M0RBJ0	Guanine nucleotide-binding protein gamma-2
Q63942	Ras-related protein Rab-3d
P48721	Mortalin
Q63584	Transmembrane emp24 domain-containing protein 10
P10960	Prosaposin
Q6EV76	Alpha-(1,6)-fucosyltransferase
Q5XIM9	T-complex protein 1 beta
F1M779	Clathrin heavy chain
P05065	Fructose-bisphosphate aldolase A
P32551	Cytochrome b-c1 complex subunit 2 mitochondrial
B0BN52	Mitochondrial carrier homolog 2
E9PTX9	Solute carrier family 12 member 2
Q5XFX0	Transgelin-2
Q7TSA0	Mitochondrial Rho GTPase 2
P61805	Dolichyl-diphosphooligosaccharide protein glycosyltransferase subunit DAD1

G3V908	keratin, type II Kb15
P09811	Glycogen phosphorylase liver form
P53987	Monocarboxylate transporter 1
G3V6N2	Transmembrane emp24 domain-containing protein 4
P28494	Alpha-mannosidase 2
Q62902	Lectin mannose-binding 1
P61131	Beta-galactoside alpha-2,3-sialyltransferase 4
Q6PDV6	40S ribosomal protein S14
P50399	Rab GDP dissociation inhibitor beta
Q6AYD3	Proliferation-associated protein 2G4
Q9WVB1	Ras-related protein Rab-6a
Q05175	Brain acid soluble protein 1
B2RYG6	Ubiquitin thioesterase OTUB1
P62278	40S ribosomal protein S13
Q3T1K5	F-actin-capping protein subunit alpha-2
P62138	Serine/threonine-protein phosphatase PP1-alpha catalytic subunit
P68370	Tubulin alpha-1A
Q5U316	Ras-related protein Rab-35
P11167	Solute carrier family 2, facilitated glucose transporter member 1
P31399	ATP synthase subunit D mitochondrial
D4A720	Serine/arginine-rich splicing factor 7 isoform 2
G3V8L7	Integrin alpha M
P04642	L-lactate dehydrogenase A chain
O35509	Ras-related protein Rab-11b
P62828	GTP-binding nuclear protein Ran
P70583	Deoxyuridine 5'-triphosphate nucleotidohydrolase
Q6AYI1	Probable ATP-dependent RNA helicase DDX5
P70580	Membrane-associated progesterone receptor component 1
P23358	60S ribosomal protein L12
Q3T1J1	Eukaryotic translation initiation factor 5A-1
F7EWC1	Vasodilator-stimulated phosphoprotein
Q66HA6	ADP-ribosylation factor-like protein 8B
P11598	Protein disulfide-isomerase A3
P62282	40S ribosomal protein S11
Q6P3V9	60S ribosomal protein L4
G3V6T1	Coatomer subunit alpha
Q6P3V8	Eukaryotic initiation factor 4A-I
Q66HA4	Tax1-binding protein 1 homolog
F1LP82	Ras-related protein Rab-2a

Q62636	Ras-related protein Rap-1b
Q3MIE4	Synaptic vesicle membrane protein VAT-1 homolog
Q6P742	Proteolipid protein 2
P04636	Malate dehydrogenase mitochondrial
P62914	60S ribosomal protein L11
Q5U300	Ubiquitin-like modifier-activating enzyme 1
G3V7I0	Peroxiredoxin-3
P62815	V-type proton ATPase subunit B
Q9JID2	Guanine nucleotide-binding protein alpha-11
Q63198	Contactin 1
P11980	Pyruvate kinase PKM
Q9Z2J5	VAMP5
P10719	ATP synthase subunit beta mitochondrial
D3ZAY7	Band 4.1-like protein 2
G3V741	Solute carrier family 25 member 3
O88941	Mannosyl-oligosaccharide glucosidase
O54980	Synaptogyrin-2
Q6P6T6	Cathepsin D
Q4V7D9	Acid sphingomyelinase-like phosphodiesterase 3B
Q6S395	Plectin
F1LMP7	Granulins
Q5XI04	Erythrocyte band 7 integral membrane protein
F1LQJ7	Phosphoenolpyruvate carboxykinase [GTP], mitochondrial
Q9R1T1	Barrier-to-autointegration factor
P22734	Catechol O-methyltransferase
P62804	Histone H4
F1LM93	Tyrosine-protein kinase Yes
Q63083	Nucleobindin-1
Q9JKB7	Guanine deaminase
P12928	Pyruvate kinase PKLR
Q63081	Protein disulfide-isomerase A6
G3V9S3	Tyrosine-protein phosphatase non-receptor type 13
P13832	Myosin regulatory light chain RLC-A
B1WBY5	DnaJ homolog subfamily C member 11
Q62940	E3 ubiquitin-protein ligase NEDD4
Q62658	Peptidyl-prolyl cis-trans isomerase FKBP1A
P68255	14-3-3 protein theta
Q63803	Guanine nucleotide-binding protein G(s) subunit alpha isoforms XLas
Q6P6U0	Tyrosine-protein kinase Fgr

Q62753	Syntaxin-binding protein 2
D4A5K6	Zinc metallopeptidase STE24
P13838	Leukosialin
Q06647	ATP synthase subunit O mitochondrial
G3V637	Syntaxin-binding protein 2, isoform
P62859	40S ribosomal protein S28
Q6IN24	Galectin-8
G3V8I4	Syntaxin-4A
Q6IFV6	Keratin 35
P09895	60S ribosomal protein L5
Q63507	60S ribosomal protein L14
P10111	Peptidyl-prolyl cis-trans isomerase A
G3V679	Transferrin receptor protein 1
P62260	14-3-3 protein epsilon
Q5XIE8	Integral membrane protein 2B
B2GUZ9	Fam49b protein
Q6IG03	Keratin, type II cytoskeletal 73
Q6IG02	Keratin, type II cytoskeletal 2 epidermal
Q6IG01	Keratin, type II cytoskeletal 1B
Q9Z0V6	Thioredoxin-dependent peroxide reductase mitochondrial
Q3SWT0	Platelet endothelial cell adhesion molecule (Pecam1)
Q6IN10	Endothelin-converting enzyme 1 Isoform
Q6IG05	Keratin, type II cytoskeletal 75
Q6IFW6	Keratin, type I cytoskeletal 10
P09495	Tropomyosin alpha-4
Q5XIF6	Tubulin alpha-4A
D3ZWS0	Protein scribble homolog
P62963	Profilin-1
Q66HD0	Endoplasmin
P62250	40S ribosomal protein S16
Q91Y81	Septin-2
Q99P74	Ras-related protein Rab-27b
P70550	Ras-related protein Rab-8b
Q99P75	Ras-related protein Rab-9a
Q6IG12	Keratin, type II cytoskeletal 7
P35704	Peroxiredoxin-2
Q2I6B2	V-type proton ATPase subunit A1
G3V8G5	Golgi apparatus protein 1
P62630	Elongation factor 1-alpha 1

P62775	Myotrophin
Q6P6Q2	Keratin, type II cytoskeletal 5
P62246	40S ribosomal protein S15a
D3ZNQ6	NEDD8-conjugating enzyme Ubc12
P62832	60S ribosomal protein L23
P05712	Ras-related protein Rab-2a
P62836	Ras-related protein Rap-1a
P62243	40S ribosomal protein S8
E9PSV5	Phosphoserine aminotransferase
Q9JLT0	Myosin heavy chain 10
P82995	Heat shock protein HSP 90-alpha
P42667	SEC11 homolog A
G3V8F5	Mitochondrial import receptor subunit TOM40 homolog
Q6IN37	GM2 ganglioside activator
P80254	D-dopachrome decarboxylase
P13383	Nucleolin
Q63610	Tropomyosin alpha-3
Q66X93	Staphylococcal nuclease domain-containing protein 1
Q6IFZ5	Keratin, type II cytoskeletal 2 oral
Q5VLR5	Endoplasmic reticulum resident protein 44
P62986	Ubiquitin-60S ribosomal protein L40
Q6RUV5	Ras-related C3 botulinum toxin substrate 1 (Rac1)
P62982	Ubiquitin-40S ribosomal protein S27a
P82471	Guanine nucleotide-binding protein G(q) subunit alpha
B4F7C2	Tubulin beta-4A
E9PT65	Radixin
Q9EPH8	Polyadenylate-binding protein 1
Q6MG61	Chloride intracellular channel protein 1
P19944	60S acidic ribosomal protein P1
Q6TXF3	Acyl-CoA-binding protein
P19945	60S acidic ribosomal protein P0
B2RZ27	SH3 domain-binding glutamic acid-rich-like protein 3
B2RYQ2	Serine/threonine-protein phosphatase 2A activator
P11240	Cytochrome c oxidase subunit 5A mitochondrial
P11232	Thioredoxin
P05982	NAD(P)H dehydrogenase [quinone] 1
P10252	CD48 antigen
P69897	Tubulin beta-5
P62890	60S ribosomal protein L30

P63095	Guanine nucleotide-binding protein G(s) subunit alpha
P52296	Importin subunit beta-1
D3ZTJ0	Protein XRP2
Q4QRB4	Tubulin beta-3
Q6P9V9	Tubulin alpha-1B
B2GUV5	V-type proton ATPase subunit G 1
P18418	Calreticulin
Q6P9V1	CD81 molecule
P13221	Aspartate aminotransferase cytoplasmic
Q09073	ADP/ATP translocase 2
Q6AYA5	Transmembrane protein 106 B
P54921	Alpha-soluble NSF attachment protein (SNAP-alpha)
P25113	Phosphoglycerate mutase 1
Q3KRD8	Eukaryotic translation initiation factor 6
G3V8C4	Chloride intracellular channel protein 4
P61983	14-3-3 protein gamma
P49242	40S ribosomal protein S3a
B0BNA5	Coactosin-like protein
P02401	60S acidic ribosomal protein P2
P50137	Transketolase
P09527	Ras-related protein Rab-7a
P10888	Cytochrome c oxidase subunit 4 isoform 1 mitochondrial
Q66HG5	Transmembrane 9 superfamily member 2
Q5U3Z7	Serine hydroxymethyltransferase
P48500	Triosephosphate isomerase
P23640	Ras-related protein Rab-27a
P62747	Rho-related GTP-binding protein RhoB
O70595	ATP-binding cassette sub-family B member 6
B2GV92	Prostaglandin E synthase 3
P62083	40S ribosomal protein S7
Q6IFU9	keratin 16
Q6P9T8	Tubulin beta-4B
Q6IFU8	Keratin, type I cytoskeletal 17
P04157	Receptor-type tyrosine-protein phosphatase C (CD45)
Q6IFV3	Keratin, type I cytoskeletal 15
Q6IFV1	Keratin, type I cytoskeletal 14
P20070	NADH-cytochrome b5 reductase 3
Q5XIH7	Prohibitin 2
G3V8A5	Vacuolar protein sorting-associated protein 35

P11348	Dihydropteridine reductase
D4A465	Ragulator complex protein LAMTOR2
Q5I0G4	Glycine--tRNA ligase
Q5U1Y2	Ras-related C3 botulinum toxin substrate 2 (Rac2)
P85007	Immediate early response 3-interacting protein 1
Q00238	Intercellular adhesion molecule 1 (ICAM1)
Q5M843	2-oxoglutarate and iron-dependent oxygenase domain-containing protein 3
Q5EB77	Ras-related protein Rab-18
Q6AY18	GTP-binding protein SAR1a
D3ZIP8	Endonuclease domain containing 1
P19804	Nucleoside diphosphate kinase B
D4ADS9	EFR3 homolog A
B2GVB9	Fermt3 protein
B0BNG3	Vesicular integral-membrane protein VIP36
Q6AXT5	Ras-related protein Rab-21
Q6NYB7	Ras-related protein Rab-1a
D3ZPR0	Exportin-2
P19511	ATP synthase F(0) complex subunit B1 mitochondrial
G3V8Z1	Linker for activation of T-cells family member 2 (NTAL)
Q76GL9	Neutral amino acid transporter A
F1LNF0	Myosin heavy chain 14
P17074	40S ribosomal protein S19
Q5M7T6	V-type proton ATPase subunit D 1
P63245	Receptor of activated protein C kinase 1 (RACK1)
Q8SEZ5	Cytochrome c oxidase subunit 2
Q4KM73	UMP-CMP kinase
A1L134	Ancient ubiquitous protein 1
P46462	Transitional endoplasmic reticulum ATPase
P35171	Cytochrome c oxidase subunit 7A2
P04961	Proliferating cell nuclear antigen
Q32PX6	Rho-related GTP-binding protein RhoG
Q3KRC3	Signal recognition particle receptor alpha
Q6AXS5	Plasminogen activator inhibitor 1 RNA-binding protein
Q6AXS4	Renin receptor
P05426	60S ribosomal protein L7
D4A4T0	STIP1 homology and U box-containing protein 1
D4A0E2	NSF attachment protein gamma
P16617	Phosphoglycerate kinase 1
P45592	Cofilin-1

Q01621	Tyrosine-protein kinase LCK
F1LZX9	Integrin alpha 5
F1MAS0	keratin 32
Q9JJ19	Na(+)/H(+) exchange regulatory cofactor NHE-RF1
P08753	Guanine nucleotide-binding protein G(k) subunit alpha
D3ZPP2	ADP-ribosylation factor-like protein 8A
P63269	Actin gamma-enteric smooth muscle
P15429	Beta-enolase
Q63692	Hsp90 co-chaperone Cdc37
P97675	Ectonucleotide pyrophosphatase/phosphodiesterase family member 3
Q5XIA8	Growth hormone-inducible transmembrane protein
Q5M7W6	Protein FAM234A
D4A9L2	Serine/arginine-rich splicing factor 1
P35289	Ras-related protein Rab-15
D3ZZR9	Peptidylprolyl isomerase
P63018	Heat shock cognate 71 kDa protein
P35565	Calnexin
Q9R0C5	Polypeptide N-acetylgalactosaminyltransferase 7
B0BNK1	Ras-related protein Rab-5c
P17046	Lysosome-associated membrane glycoprotein 2 (Lamp2)
G3V6D3	ATP synthase subunit beta
D3ZDR2	Charged multivesicular body protein 6
E9PTI1	V-type proton ATPase subunit H
P85108	Tubulin beta-2A
D3ZZS8	V-type proton ATPase subunit B, kidney isoform
P35435	ATP synthase subunit gamma mitochondrial
D3ZGM7	Peptidase inhibitor 16
Q08163	Adenylyl cyclase-associated protein 1
P26453	Basigin
Q5RKJ9	Ras-related protein Rab-10
Q5M860	Rho GDP dissociation inhibitor 2
Q8CGU6	Nicastrin
P63039	60 kDa heat shock protein
P14659	Heat shock-related 70 kDa protein 2
Q6IRF8	V-type proton ATPase subunit S1
P00507	Aspartate aminotransferase mitochondrial
Q3MHS9	Chaperonin-containing TCP1 subunit 6A
M0RC99	Ras-related protein Rab-5a
Q9JK11	Reticulon-4

P14562	Lysosome-associated membrane glycoprotein 1 (Lamp1)
Q6AXV4	Sorting and assembly machinery component 50 homolog
P14668	Annexin A5
P49432	Pyruvate dehydrogenase component E1 beta
Q4G075	Leukocyte elastase inhibitor
Q9Z1E1	Flotillin-1
Q6IMF3	Keratin, type II cytoskeletal 1
Q920J4	Thioredoxin-like protein 1
P24368	Peptidyl-prolyl cis-trans isomerase B
Q499P3	C1GALT1-specific chaperone 1
B0BNJ1	Sorcin
P19627	Guanine nucleotide-binding protein G(z) subunit alpha
G3V9A3	14-3-3 protein sigma
P35280	Ras-related protein Rab-8a

ID Term	GO Term	Fold Enriched	Count	*p* -value	FDR
**GO:0031988**	**MEMBRANE-BOUNDED VESICLE**	**3.18**	**538**	**2.78E-166**	**4.30E-163**
**Annotated proteins**					
**Protein Accession**	**Protein Identification**				
D3ZAF6	ATP synthase subunit F mitochondrial				
Q6P503	V-type proton ATPase subunit D				
B5DEH2	Erlin-2				
F1M2P8	Protein kinase C alpha				
P21961	Mast cell carboxypeptidase A				
P50408	V-type proton ATPase subunit F				
Q62876	Synaptogyrin-1				
Q63716	Peroxiredoxin-1				
P26772	10 kDa heat shock protein mitochondrial				
Q5U204	Ragulator complex protein LAMTOR3				
P0DP29	Calmodulin-1				
G3V852	Talin-1				
D3ZTX0	Transmembrane emp24 domain-containing protein 7				
P50503	Hsc70-interacting protein				
O08623	Sequestosome-1				
P06685	Sodium/potassium-transporting ATPase subunit alpha-1				
Q9ESV6	Glyceraldehyde-3-phosphate dehydrogenase testis-specific				
Q5XI52	Vezatin				

P07632	Superoxide dismutase [Cu-Zn]
Q66H18	Synaptophysin-like 1
G3V6H0	Ras-related protein Rab-1b
P27139	Carbonic anhydrase 2
Q7TP15	S-methyl-5'-thioadenosine phosphorylase
Q9ES40	PRA1 family protein 3
P61589	Transforming protein RhoA
G3V7U6	Calpain 5
P68511	14-3-3 protein eta
Q01205	2-oxoglutarate dehydrogenase complex component E2
P67779	Prohibitin
Q5FVF4	Transmembrane 7 superfamily member 3
Q5BJY9	Keratin, type I cytoskeletal 18
F1M5Z5	Anoctamin-6
D3ZRN3	Beta-actin-like protein 2
Q62991	Syntaxin-binding protein 1-like 2
O08651	D-3-phosphoglycerate dehydrogenase
O88989	Malate dehydrogenase cytoplasmic
P11762	Galectin-1
P27615	Lysosome membrane protein 2 (CD36)
Q6AYK6	Calcyclin-binding protein
P06761	Endoplasmic reticulum chaperone BiP
Q9QYV0	Disintegrin and metalloproteinase domain-containing protein 15
P63102	14-3-3 protein zeta/delta
B0K020	CDGSH iron-sulfur domain-containing protein 1
Q9EST6	Acidic leucine-rich nuclear phosphoprotein 32 family member B
Q78P65	Rap2A-like protein
P07943	Aldose reductase
P32089	Citrate transport protein
M0RCF3	Integrin beta 3
Q5XI34	Serine/threonine-protein phosphatase 2A 65 kDa regulatory subunit A alpha isoform
Q5XI32	F-actin-capping protein subunit beta
P83732	60S ribosomal protein L24
Q5XIV1	Phosphoglycerate kinase 2
P52555	Endoplasmic reticulum resident protein 29
O88994	Mitochondrial amidoxime reducing component 2
Q5XI38	Lymphocyte cytosolic protein 1
P04764	Alpha-enolase
Q5XI36	CD97

B0K010	Thioredoxin domain-containing 17
Q10758	Keratin, type II cytoskeletal 8
P06765	Platelet factor 4
Q07936	Annexin A2
P05197	Elongation factor 2
Q9R1Z0	Voltage-dependent anion-selective channel protein 3
P85968	6-phosphogluconate dehydrogenase decarboxylating
P63322	Ras-related protein Ral-A
D3ZD83	Major facilitator superfamily domain-containing protein 10
Q63396	Activated RNA polymerase II transcriptional coactivator p15
P54313	Transducin beta-2
Q6AYQ9	Peptidyl-prolyl cis-trans isomerase C
F1MA89	Cyclin Y
P54311	Transducin beta-1
Q6PDU1	Serine/arginine-rich splicing factor 2
Q6AYQ4	Transmembrane protein 109
O70257	Syntaxin-7
O88767	Protein/nucleic acid deglycase DJ-1
D4AEP0	Adenylosuccinate synthetase isozyme 2
P51577	P2X purinoceptor 4
P35213	14-3-3 protein beta/alpha
D3ZUX5	MICOS complex subunit MIC-19
P34058	Heat shock protein HSP 90-beta
O08775	Vascular endothelial growth factor receptor 2
Q66H80	Coatomer subunit delta
Q9EQX9	Ubiquitin-conjugating enzyme E2 N
G3V7P1	Syntaxin-12
P15865	Histone H1.4
Q920P6	Adenosine deaminase
Q91ZN1	Coronin-1A
P04785	Protein disulfide-isomerase
G3V9Y2	Phospholipid phosphatase 1
G3V7Q7	Ras GTPase-activating-like protein IQGAP1
Q63016	Large neutral amino acids transporter small subunit 1
O35165	Golgi SNAP receptor complex member 2
Q5EAP4	Guanine nucleotide binding protein alpha-14
R9PXR4	Mitochondrial import receptor subunit TOM70
P63331	Serine/threonine-protein phosphatase 2A catalytic subunit alpha isoform
Q63116	Mast/stem cell growth factor receptor

P85971	6-phosphogluconolactonase
D3ZSY5	keratin 84
Q64244	ADP-ribosyl cyclase/cyclic ADP-ribose hydrolase 1
P81155	Voltage-dependent anion-selective channel protein 2
P21531	60S ribosomal protein L3
P31000	Vimentin
P12368	cAMP-dependent protein kinase type II-alpha regulatory subunit
Q9WU70	Syntaxin-binding protein 5
F7EPH4	Inorganic Pyrophosphatase
Q5I0E7	Transmembrane emp24 domain-containing protein 9
A1L1J8	Ras-related protein Rab-5b
P38983	40S ribosomal protein SA
B0K031	60S ribosomal protein L7, isoform
F1LN88	Aldehyde dehydrogenase
F7FFV2	keratin 5
Q05982	Nucleoside diphosphate kinase A
F1LQR8	Inositol 1,4,5-trisphosphate receptor type 2
P21708	Mitogen-activated protein kinase 3
P04797	Glyceraldehyde-3-phosphate dehydrogenase
O35567	Bifunctional purine biosynthesis protein PURH
P04897	Guanine nucleotide-binding protein G(i) subunit alpha-2
G3V936	Citrate synthase
Q8VHF5	Citrate synthase mitochondrial
Q08849	Syntaxin-3
Q8CFN2	Cell division control protein 42 homolog (CDC42)
Q9EPV5	Apoptotic protease-activating factor 1
Q5I0D1	Glyoxalase domain-containing protein 4
Q53B90	Ras-related protein Rab-43
P58195	Phospholipid scramblase 1
Q9JHW5	VAMP7
Q9JI85	Nucleobindin-2
P01830	Thy-1 membrane glycoprotein
O70377	Synaptosomal-associated protein 23 (SNAP-23)
Q68FP1	Gelsolin
D4A133	V-type proton ATPase catalytic subunit A
P21913	Succinate dehydrogenase [ubiquinone] iron-sulfur subunit mitochondrial
P51146	Ras-related protein Rab-4b
Q07014	Tyrosine-protein kinase Lyn
D3ZK56	Ras-related protein Rap-2c

Q07984	Translocon-associated protein subunit delta
Q5XI77	Annexin A11
P31977	Ezrin
Q5XI73	Rho GDP dissociation inhibitor 1
P07151	Beta-2-microglobulin
P15999	ATP synthase subunit alpha mitochondrial
P07150	Annexin A1
Q6AYQ1	Golgin subfamily A member 7
P60711	Actin cytoplasmic 1
O35353	Transducin beta-4
B2RYB8	Integrin beta 2
F1LW77	Ras-related protein Rab-33b
Q9Z2S9	Flotillin-2
P07153	Dolichyl-diphosphooligosaccharide protein glycosyltransferase subunit 1
Q5U362	Annexin A4
Q9Z2L0	Voltage-dependent anion-selective channel protein 1
Q68FR6	Elongation factor 1-gamma
B2RYC9	Glucosylceramidase
P51635	Alcohol dehydrogenase [NADP(+)]
E9PTU4	Myosin heavy chain 11
F1LM82	Heterogeneous nuclear ribonucleoproteins A2/B1
P11442	Clathrin heavy chain 1
B0BN81	Ribosomal protein S5
P12075	Cytochrome c oxidase subunit 5B mitochondrial
Q05030	Platelet-derived growth factor receptor beta
G3V7L8	V-type proton ATPase subunit E 1 isoform
Q62662	Tyrosine-protein kinase FRK
P61107	Ras-related protein Rab-14
F1LPV0	Asparagine--tRNA ligase, cytoplasmic
P61972	Nuclear transport factor 2
Q6PCU2	V-type proton ATPase subunit E 1
Q5PQL7	Integral membrane protein 2C
M0R9L0	Nascent polypeptide-associated complex subunit alpha
P49134	Integrin beta 1
P50475	Alanine--tRNA ligase cytoplasmic
G3V712	keratin 7
Q68FQ0	T-complex protein 1 epsilon
Q810F4	Protein FAM3C
G3V8Q1	Coatomer subunit epsilon

P31044	Phosphatidylethanolamine-binding protein 1
E9PU77	Synaptotagmin-like protein 3
Q4QQV0	Tubulin beta-6
F1LQ81	Vesicle-fusing ATPase
Q63269	Inositol 1,4,5-trisphosphate receptor type 3
Q3B7V5	Ras-related protein Rab-2b
D4A0T0	NADH dehydrogenase 1 beta subcomplex subunit 10
G3V7M2	Integrin beta 7
G3V6P8	Guanine nucleotide-binding protein gamma-12
G3V6P7	Myosin heavy chain 9
Q04970	GTPase NRas
D3ZNG3	Signal peptide peptidase-like 2A
Q9Z1J7	Amino acid transporter
Q9WUD2	Transient receptor potential cation channel subfamily V member 2 (TRPV2)
E9PTW1	Secretory carrier-associated membrane protein
R9PXU4	Thioredoxin reductase 1 cytoplasmic
F1LSN4	Fibroblast growth factor receptor
D4ADE8	ATP-dependent RNA helicase DDX3X
O88600	Heat shock 70 kDa protein 4
G3V9N9	alpha-1,2-Mannosidase
Q794F9	4F2 cell-surface antigen heavy chain
Q6P791	Ragulator complex protein LAMTOR1
F1LNT8	VAMP8
P61227	Ras-related protein Rap-2b
Q6P799	Serine--tRNA ligase cytoplasmic
P62703	40S ribosomal protein S4, X isoform
P62909	40S ribosomal protein S3
P62907	60S ribosomal protein L10a
Q6AYC4	Macrophage-capping protein
Q63377	Sodium/potassium-transporting ATPase subunit beta-3
M0RBJ0	Guanine nucleotide-binding protein gamma-2
Q63942	Ras-related protein Rab-3d
P48721	Mortalin
Q63584	Transmembrane emp24 domain-containing protein 10
P10960	Prosaposin
Q5PQM0	Transmembrane protein 168
Q6EV76	Alpha-(1,6)-fucosyltransferase
Q5XIM9	T-complex protein 1 beta
F1M779	Clathrin heavy chain

P05065	Fructose-bisphosphate aldolase A
P32551	Cytochrome b-c1 complex subunit 2 mitochondrial
B0BN52	Mitochondrial carrier homolog 2
E9PTX9	Solute carrier family 12 member 2
Q5XFX0	Transgelin-2
Q7TSA0	Mitochondrial Rho GTPase 2
P61805	Dolichyl-diphosphooligosaccharide protein glycosyltransferase subunit DAD1
G3V908	keratin, type II Kb15
P53987	Monocarboxylate transporter 1
P09811	Glycogen phosphorylase liver form
G3V6N2	Transmembrane emp24 domain-containing protein 4
Q62902	Lectin mannose-binding 1
P28494	Alpha-mannosidase 2
P61131	Beta-galactoside alpha-2,3-sialyltransferase 4
Q6PDV6	40S ribosomal protein S14
P50399	Rab GDP dissociation inhibitor beta
Q6AYD3	Proliferation-associated protein 2G4
Q9WVB1	Ras-related protein Rab-6a
Q05175	Brain acid soluble protein 1
Q62839	Golgin subfamily A member 2
B2RYG6	Ubiquitin thioesterase OTUB1
P62278	40S ribosomal protein S13
Q3T1K5	F-actin-capping protein subunit alpha-2
P62138	Serine/threonine-protein phosphatase PP1-alpha catalytic subunit
P68370	Tubulin alpha-1A
Q5U316	Ras-related protein Rab-35
P11167	Solute carrier family 2, facilitated glucose transporter member 1
P31399	ATP synthase subunit D mitochondrial
D4A720	Serine/arginine-rich splicing factor 7 isoform 2
G3V8L7	Integrin alpha M
P04642	L-lactate dehydrogenase A chain
O35509	Ras-related protein Rab-11b
P62828	GTP-binding nuclear protein Ran
Q6AYI1	Probable ATP-dependent RNA helicase DDX5
P70583	Deoxyuridine 5'-triphosphate nucleotidohydrolase
P70580	Membrane-associated progesterone receptor component 1
P23358	60S ribosomal protein L12
D4ABY2	Coatomer subunit gamma 2
Q3T1J1	Eukaryotic translation initiation factor 5A-1

D4AEG2	Ras-related protein Rab-32
F7EWC1	Vasodilator-stimulated phosphoprotein
Q66HA6	ADP-ribosylation factor-like protein 8B
G3V6T7	Protein disulfide-isomerase A4
P11598	Protein disulfide-isomerase A3
P62282	40S ribosomal protein S11
Q6P3V9	60S ribosomal protein L4
G3V6T1	Coatomer subunit alpha
Q6P3V8	Eukaryotic initiation factor 4A-I
Q66HA4	Tax1-binding protein 1 homolog
F1LP82	Ras-related protein Rab-2a
Q62636	Ras-related protein Rap-1b
Q6P742	Proteolipid protein 2
Q3MIE4	Synaptic vesicle membrane protein VAT-1 homolog
P04636	Malate dehydrogenase mitochondrial
P62914	60S ribosomal protein L11
Q5U300	Ubiquitin-like modifier-activating enzyme 1
G3V7I0	Peroxiredoxin-3
P62815	V-type proton ATPase subunit B
Q9JID2	Guanine nucleotide-binding protein alpha-11
Q63198	Contactin 1
P11980	Pyruvate kinase PKM
Q9Z2J5	VAMP5
P10719	ATP synthase subunit beta mitochondrial
D3ZAY7	Band 4.1-like protein 2
G3V741	Solute carrier family 25 member 3
O88941	Mannosyl-oligosaccharide glucosidase
O54980	Synaptogyrin-2
Q6P6T6	Cathepsin D
Q4V7D9	Acid sphingomyelinase-like phosphodiesterase 3B
Q6S395	Plectin
F1LMP7	Granulins
Q5XI04	Erythrocyte band 7 integral membrane protein
F1LQJ7	Phosphoenolpyruvate carboxykinase [GTP], mitochondrial
Q9R1T1	Barrier-to-autointegration factor
P22734	Catechol O-methyltransferase
P62804	Histone H4
Q63483	Ras-related protein Rab-38
F1LM93	Tyrosine-protein kinase Yes

Q63083	Nucleobindin-1
Q9JKB7	Guanine deaminase
P12928	Pyruvate kinase PKLR
Q63081	Protein disulfide-isomerase A6
P13832	Myosin regulatory light chain RLC-A
G3V9S3	Tyrosine-protein phosphatase non-receptor type 13
B1WBY5	DnaJ homolog subfamily C member 11
Q62940	E3 ubiquitin-protein ligase NEDD4
Q62658	Peptidyl-prolyl cis-trans isomerase FKBP1A
P68255	14-3-3 protein theta
Q63803	Guanine nucleotide-binding protein G(s) subunit alpha isoforms XLas
Q6P6U0	Tyrosine-protein kinase Fgr
Q62753	Syntaxin-binding protein 2
D4A5K6	Zinc metallopeptidase STE24
P13838	Leukosialin
Q06647	ATP synthase subunit O mitochondrial
G3V637	Syntaxin-binding protein 2, isoform
P62859	40S ribosomal protein S28
Q6IN24	Galectin-8
Q6PEC3	Protein YIF1B
G3V8I4	Syntaxin-4A
Q6IFV6	Keratin 35
P09895	60S ribosomal protein L5
Q63507	60S ribosomal protein L14
P10111	Peptidyl-prolyl cis-trans isomerase A
Q5XIE0	Acidic leucine-rich nuclear phosphoprotein 32 family member E
G3V679	Transferrin receptor protein 1
P29995	Inositol 1,4,5-trisphosphate receptor type 2
P62260	14-3-3 protein epsilon
Q5XIE8	Integral membrane protein 2B
B2GUZ9	Fam49b protein
Q6IG03	Keratin, type II cytoskeletal 73
Q6IG02	Keratin, type II cytoskeletal 2 epidermal
Q6IG01	Keratin, type II cytoskeletal 1B
Q9Z0V6	Thioredoxin-dependent peroxide reductase mitochondrial
Q3SWT0	Platelet endothelial cell adhesion molecule (Pecam1)
Q6IN10	Endothelin-converting enzyme 1 Isoform
Q6IG05	Keratin, type II cytoskeletal 75
Q6IFW6	Keratin, type I cytoskeletal 10

P09495	Tropomyosin alpha-4
Q5XIF6	Tubulin alpha-4A
D3ZWS0	Protein scribble homolog
P62963	Profilin-1
Q66HD0	Endoplasmin
P62250	40S ribosomal protein S16
Q91Y81	Septin-2
P70550	Ras-related protein Rab-8b
Q99P74	Ras-related protein Rab-27b
Q99P75	Ras-related protein Rab-9a
Q6IG12	Keratin, type II cytoskeletal 7
P35704	Peroxiredoxin-2
Q2I6B2	V-type proton ATPase subunit A1
G3V8G5	Golgi apparatus protein 1
Q2I6B1	V-type proton ATPase subunit A2
P62630	Elongation factor 1-alpha 1
P62775	Myotrophin
Q6P6Q2	Keratin, type II cytoskeletal 5
P62246	40S ribosomal protein S15a
D3ZNQ6	NEDD8-conjugating enzyme Ubc12
P62832	60S ribosomal protein L23
P05712	Ras-related protein Rab-2a
P62836	Ras-related protein Rap-1a
E9PSV5	Phosphoserine aminotransferase
P62243	40S ribosomal protein S8
Q01827	Vesicular amine transporter 2
Q9JLT0	Myosin heavy chain 10
P82995	Heat shock protein HSP 90-alpha
P42667	SEC11 homolog A
G3V8F5	Mitochondrial import receptor subunit TOM40 homolog
Q6IN37	GM2 ganglioside activator
Q5XID0	Protein YIPF5
P80254	D-dopachrome decarboxylase
P13383	Nucleolin
Q63610	Tropomyosin alpha-3
Q66X93	Staphylococcal nuclease domain-containing protein 1
Q6IFZ5	Keratin, type II cytoskeletal 2 oral
Q5VLR5	Endoplasmic reticulum resident protein 44
P62986	Ubiquitin-60S ribosomal protein L40

Q6RUV5	Ras-related C3 botulinum toxin substrate 1 (Rac1)
P62982	Ubiquitin-40S ribosomal protein S27a
P82471	Guanine nucleotide-binding protein G(q) subunit alpha
B4F7C2	Tubulin beta-4A
E9PT65	Radixin
Q9EPH8	Polyadenylate-binding protein 1
Q6MG61	Chloride intracellular channel protein 1
P19944	60S acidic ribosomal protein P1
Q6TXF3	Acyl-CoA-binding protein
P19945	60S acidic ribosomal protein P0
B2RZ27	SH3 domain-binding glutamic acid-rich-like protein 3
B2RYQ2	Serine/threonine-protein phosphatase 2A activator
P11240	Cytochrome c oxidase subunit 5A mitochondrial
E9PU37	Palmitoyltransferase
P11232	Thioredoxin
P05982	NAD(P)H dehydrogenase [quinone] 1
P10252	CD48 antigen
P69897	Tubulin beta-5
P62890	60S ribosomal protein L30
P63095	Guanine nucleotide-binding protein G(s) subunit alpha
P52296	Importin subunit beta-1
D3ZTJ0	Protein XRP2
Q4QRB4	Tubulin beta-3
Q6P9V9	Tubulin alpha-1B
B2GUV5	V-type proton ATPase subunit G 1
P18418	Calreticulin
Q6P9V1	CD81 molecule
Q63635	Syntaxin-6
P13221	Aspartate aminotransferase cytoplasmic
Q09073	ADP/ATP translocase 2
Q6AYA5	Transmembrane protein 106 B
A7E3N2	Neutrophil cytosol factor 2
P54921	Alpha-soluble NSF attachment protein (SNAP-alpha)
Q3KRD8	Eukaryotic translation initiation factor 6
P25113	Phosphoglycerate mutase 1
G3V8C4	Chloride intracellular channel protein 4
P61983	14-3-3 protein gamma
P49242	40S ribosomal protein S3a
B0BNA5	Coactosin-like protein

P02401	60S acidic ribosomal protein P2
P50137	Transketolase
P09527	Ras-related protein Rab-7a
P10888	Cytochrome c oxidase subunit 4 isoform 1 mitochondrial
Q66HG5	Transmembrane 9 superfamily member 2
Q5U3Z7	Serine hydroxymethyltransferase
P48500	Triosephosphate isomerase
Q63524	Transmembrane emp24 domain-containing protein 2
P23640	Ras-related protein Rab-27a
P62747	Rho-related GTP-binding protein RhoB
O70595	ATP-binding cassette sub-family B member 6
B2GV92	Prostaglandin E synthase 3
Q4AEF8	Coatomer subunit gamma 1
P62083	40S ribosomal protein S7
B2GV96	Coiled-coil domain containing 115
Q6IFU9	keratin 16
Q6P9T8	Tubulin beta-4B
Q6IFU8	Keratin, type I cytoskeletal 17
P04157	Receptor-type tyrosine-protein phosphatase C (CD45)
Q6IFV3	Keratin, type I cytoskeletal 15
Q6IFV1	Keratin, type I cytoskeletal 14
P20070	NADH-cytochrome b5 reductase 3
Q5XIH7	Prohibitin 2
G3V8A5	Vacuolar protein sorting-associated protein 35
D3ZP98	Minor histocompatibility antigen H13
P11348	Dihydropteridine reductase
D4A465	Ragulator complex protein LAMTOR2
Q5I0G4	Glycine--tRNA ligase
Q5U1Y2	Ras-related C3 botulinum toxin substrate 2 (Rac2)
P85007	Immediate early response 3-interacting protein 1
Q642A2	Type-1 angiotensin II receptor-associated protein
Q6AZ61	Probable lysosomal cobalamin transporter
Q00238	Intercellular adhesion molecule 1 (ICAM1)
Q5M843	2-oxoglutarate and iron-dependent oxygenase domain-containing protein 3
Q5EB77	Ras-related protein Rab-18
Q6AY18	GTP-binding protein SAR1a
D3ZIP8	Endonuclease domain containing 1
P19804	Nucleoside diphosphate kinase B
D4ADS9	EFR3 homolog A

B2GVB9	Fermt3 protein
B0BNG3	Vesicular integral-membrane protein VIP36
Q6AXT5	Ras-related protein Rab-21
D4A376	Ras-related protein Rab-12
Q6NYB7	Ras-related protein Rab-1a
D3ZPR0	Exportin-2
P19511	ATP synthase F(0) complex subunit B1 mitochondrial
G3V8Z1	Linker for activation of T-cells family member 2 (NTAL)
P19814	Trans-Golgi network integral membrane protein TGN38
Q76GL9	Neutral amino acid transporter A
F1LNF0	Myosin heavy chain 14
P17074	40S ribosomal protein S19
Q5M7T6	V-type proton ATPase subunit D 1
P63245	Receptor of activated protein C kinase 1 (RACK1)
Q8SEZ5	Cytochrome c oxidase subunit 2
Q4KM73	UMP-CMP kinase
A1L134	Ancient ubiquitous protein 1
Q4KM74	Vesicle-trafficking protein SEC22b
P46462	Transitional endoplasmic reticulum ATPase
P35171	Cytochrome c oxidase subunit 7A2
P04961	Proliferating cell nuclear antigen
Q32PX6	Rho-related GTP-binding protein RhoG
Q3KRC3	Signal recognition particle receptor alpha
Q6AXS5	Plasminogen activator inhibitor 1 RNA-binding protein
Q6AXS4	Renin receptor
P05426	60S ribosomal protein L7
D4A4T0	STIP1 homology and U box-containing protein 1
D4A0E2	NSF attachment protein gamma
P16617	Phosphoglycerate kinase 1
P45592	Cofilin-1
Q01621	Tyrosine-protein kinase LCK
F1LZX9	Integrin alpha 5
F1MAS0	keratin 32
O35142	Coatomer subunit beta 2
Q9JJ19	Na(+)/H(+) exchange regulatory cofactor NHE-RF1
P08753	Guanine nucleotide-binding protein G(k) subunit alpha
D3ZPP2	ADP-ribosylation factor-like protein 8A
P63269	Actin gamma-enteric smooth muscle
P15429	Beta-enolase

Q63692	Hsp90 co-chaperone Cdc37
P97675	Ectonucleotide pyrophosphatase/phosphodiesterase family member 3
Q5XIA8	Growth hormone-inducible transmembrane protein
D4A0G7	Ras-related protein Rab-37
Q5M7W6	Protein FAM234A
D4A9L2	Serine/arginine-rich splicing factor 1
P35289	Ras-related protein Rab-15
D3ZZR9	Peptidylprolyl isomerase
P63018	Heat shock cognate 71 kDa protein
P63012	Ras-related protein Rab-3a
P35565	Calnexin
Q9R0C5	Polypeptide N-acetylgalactosaminyltransferase 7
B0BNK1	Ras-related protein Rab-5c
P17046	Lysosome-associated membrane glycoprotein 2 (Lamp2)
O70521	Regulator of G-protein signaling 19
F1LNC4	Vesicle transport through interaction with t-SNAREs homolog 1B
G3V6D3	ATP synthase subunit beta
D3ZDR2	Charged multivesicular body protein 6
E9PTI1	V-type proton ATPase subunit H
P85108	Tubulin beta-2A
D3ZZS8	V-type proton ATPase subunit B, kidney isoform
P35435	ATP synthase subunit gamma mitochondrial
D3ZGM7	Peptidase inhibitor 16
Q08163	Adenylyl cyclase-associated protein 1
P26453	Basigin
Q5RKJ9	Ras-related protein Rab-10
Q07647	Solute carrier family 2, facilitated glucose transporter member 3
Q5M860	Rho GDP dissociation inhibitor 2
Q8CGU6	Nicastrin
P63039	60 kDa heat shock protein
P14659	Heat shock-related 70 kDa protein 2
Q6IRF8	V-type proton ATPase subunit S1
P00507	Aspartate aminotransferase mitochondrial
Q3MHS9	Chaperonin-containing TCP1 subunit 6A
M0RC99	Ras-related protein Rab-5a
Q9JK11	Reticulon-4
P14562	Lysosome-associated membrane glycoprotein 1 (Lamp1)
Q6AXV4	Sorting and assembly machinery component 50 homolog
P14668	Annexin A5

P49432	Pyruvate dehydrogenase component E1 beta
Q4G075	Leukocyte elastase inhibitor
Q9Z1E1	Flotillin-1
Q6IMF3	Keratin, type II cytoskeletal 1
P63025	VAMP3
Q920J4	Thioredoxin-like protein 1
P24368	Peptidyl-prolyl cis-trans isomerase B
Q499P3	C1GALT1-specific chaperone 1
B0BNJ1	Sorcin
P19627	Guanine nucleotide-binding protein G(z) subunit alpha
G3V9A3	14-3-3 protein sigma
P23514	Coatomer subunit beta 1
P35280	Ras-related protein Rab-8a

ID Term	GO Term	Fold Enriched	Count	*p* -value	FDR
GO:0031988	EXTRACELLULAR REGION	2.43	510	4.04E-103	6.26E-100
Annotated proteins					
Protein Accession	Protein Identification				
Q10473	Polypeptide N-acetylgalactosaminyltransferase 1				
D3ZAF6	ATP synthase subunit F mitochondrial				
Q6P503	V-type proton ATPase subunit D				
B5DEH2	Erlin-2				
F1M2P8	Protein kinase C alpha				
P21961	Mast cell carboxypeptidase A				
P50408	V-type proton ATPase subunit F				
Q63716	Peroxiredoxin-1				
P26772	10 kDa heat shock protein mitochondrial				
Q5U204	Ragulator complex protein LAMTOR3				
P0DP29	Calmodulin-1				
G3V852	Talin-1				
D3ZTX0	Transmembrane emp24 domain-containing protein 7				
P50503	Hsc70-interacting protein				
P06686	Sodium/potassium-transporting ATPase subunit alpha-2				
P06687	Sodium/potassium-transporting ATPase subunit alpha-3				
O08623	Sequestosome-1				
P06685	Sodium/potassium-transporting ATPase subunit alpha-1				
P07632	Superoxide dismutase [Cu-Zn]				

Q66H18	Synaptophysin-like 1
G3V6H0	Ras-related protein Rab-1b
P27139	Carbonic anhydrase 2
Q7TP15	S-methyl-5'-thioadenosine phosphorylase
Q9ES40	PRA1 family protein 3
P61589	Transforming protein RhoA
G3V7U6	Calpain 5
P68511	14-3-3 protein eta
Q01205	2-oxoglutarate dehydrogenase complex component E2
P67779	Prohibitin
Q5FVF4	Transmembrane 7 superfamily member 3
Q5BJY9	Keratin, type I cytoskeletal 18
F1M5Z5	Anoctamin-6
D3ZRN3	Beta-actin-like protein 2
O08651	D-3-phosphoglycerate dehydrogenase
O88989	Malate dehydrogenase cytoplasmic
P11762	Galectin-1
P27615	Lysosome membrane protein 2 (CD36)
Q6AYK6	Calcyclin-binding protein
P06761	Endoplasmic reticulum chaperone BiP
Q9QYV0	Disintegrin and metalloproteinase domain-containing protein 15
P63102	14-3-3 protein zeta/delta
B0K020	CDGSH iron-sulfur domain-containing protein 1
Q9EST6	Acidic leucine-rich nuclear phosphoprotein 32 family member B
Q78P65	Rap2A-like protein
P07943	Aldose reductase
P32089	Citrate transport protein
M0RCF3	Integrin beta 3
Q5XI34	Serine/threonine-protein phosphatase 2A 65 kDa regulatory subunit A alpha isoform
Q5XI32	F-actin-capping protein subunit beta
P83732	60S ribosomal protein L24
Q5XIV1	Phosphoglycerate kinase 2
Q62611	Interleukin 1 receptor-like 1
P52555	Endoplasmic reticulum resident protein 29
O88994	Mitochondrial amidoxime reducing component 2
Q5XI38	Lymphocyte cytosolic protein 1
P04764	Alpha-enolase
Q5XI36	CD97
B0K010	Thioredoxin domain-containing 17

Q10758	Keratin, type II cytoskeletal 8
P06765	Platelet factor 4
Q07936	Annexin A2
P05197	Elongation factor 2
Q9R1Z0	Voltage-dependent anion-selective channel protein 3
P85968	6-phosphogluconate dehydrogenase decarboxylating
P63322	Ras-related protein Ral-A
Q63396	Activated RNA polymerase II transcriptional coactivator p15
P54313	Transducin beta-2
Q6AYQ9	Peptidyl-prolyl cis-trans isomerase C
F1MA89	Cyclin Y
P54311	Transducin beta-1
Q6PDU1	Serine/arginine-rich splicing factor 2
Q6AYQ4	Transmembrane protein 109
O70257	Syntaxin-7
O88767	Protein/nucleic acid deglycase DJ-1
D4AEP0	Adenylosuccinate synthetase isozyme 2
P51577	P2X purinoceptor 4
D3ZUX5	MICOS complex subunit MIC-19
P35213	14-3-3 protein beta/alpha
P34058	Heat shock protein HSP 90-beta
Q9EQX9	Ubiquitin-conjugating enzyme E2 N
G3V7P1	Syntaxin-12
P15865	Histone H1.4
Q920P6	Adenosine deaminase
Q91ZN1	Coronin-1A
P04785	Protein disulfide-isomerase
G3V9Y2	Phospholipid phosphatase 1
G3V7Q7	Ras GTPase-activating-like protein IQGAP1
Q63016	Large neutral amino acids transporter small subunit 1
Q5EAP4	Guanine nucleotide binding protein alpha-14
R9PXR4	Mitochondrial import receptor subunit TOM70
P63331	Serine/threonine-protein phosphatase 2A catalytic subunit alpha isoform
Q63116	Mast/stem cell growth factor receptor
P85971	6-phosphogluconolactonase
D3ZSY5	keratin 84
Q64244	ADP-ribosyl cyclase/cyclic ADP-ribose hydrolase 1
P21531	60S ribosomal protein L3
P81155	Voltage-dependent anion-selective channel protein 2

G3V964	Neurotrimin
P31000	Vimentin
P12368	cAMP-dependent protein kinase type II-alpha regulatory subunit
F7EPH4	Inorganic Pyrophosphatase
Q5I0E7	Transmembrane emp24 domain-containing protein 9
A1L1J8	Ras-related protein Rab-5b
P38983	40S ribosomal protein SA
B0K031	60S ribosomal protein L7, isoform
F1LN88	Aldehyde dehydrogenase
F7FFV2	keratin 5
Q05982	Nucleoside diphosphate kinase A
P21708	Mitogen-activated protein kinase 3
P04797	Glyceraldehyde-3-phosphate dehydrogenase
O35567	Bifunctional purine biosynthesis protein PURH
P04897	Guanine nucleotide-binding protein G(i) subunit alpha-2
Q8VHF5	Citrate synthase mitochondrial
G3V936	Citrate synthase
Q08849	Syntaxin-3
Q8CFN2	Cell division control protein 42 homolog (CDC42)
Q9EPV5	Apoptotic protease-activating factor 1
Q5I0D1	Glyoxalase domain-containing protein 4
Q53B90	Ras-related protein Rab-43
P58195	Phospholipid scramblase 1
Q9JHW5	VAMP7
Q9JI85	Nucleobindin-2
P01830	Thy-1 membrane glycoprotein
O70377	Synaptosomal-associated protein 23 (SNAP-23)
Q68FP1	Gelsolin
D4A133	V-type proton ATPase catalytic subunit A
P21913	Succinate dehydrogenase [ubiquinone] iron-sulfur subunit mitochondrial
Q07014	Tyrosine-protein kinase Lyn
D3ZK56	Ras-related protein Rap-2c
Q07984	Translocon-associated protein subunit delta
Q5XI77	Annexin A11
P31977	Ezrin
Q5XI73	Rho GDP dissociation inhibitor 1
P07151	Beta-2-microglobulin
P00762	Anionic trypsin-1
P15999	ATP synthase subunit alpha mitochondrial

P07150	Annexin A1
Q6AYQ1	Golgin subfamily A member 7
P60711	Actin cytoplasmic 1
O35353	Transducin beta-4
B2RYB8	Integrin beta 2
F1LW77	Ras-related protein Rab-33b
Q9Z2S9	Flotillin-2
Q5U362	Annexin A4
Q68FR6	Elongation factor 1-gamma
Q9Z2L0	Voltage-dependent anion-selective channel protein 1
B2RYC9	Glucosylceramidase
P51635	Alcohol dehydrogenase [NADP(+)]
E9PTU4	Myosin heavy chain 11
F1LM82	Heterogeneous nuclear ribonucleoproteins A2/B1
P11442	Clathrin heavy chain 1
B0BN81	Ribosomal protein S5
P12075	Cytochrome c oxidase subunit 5B mitochondrial
Q05030	Platelet-derived growth factor receptor beta
G3V7L8	V-type proton ATPase subunit E 1 isoform
Q62662	Tyrosine-protein kinase FRK
P61107	Ras-related protein Rab-14
F1LPV0	Asparagine--tRNA ligase, cytoplasmic
P61972	Nuclear transport factor 2
Q6PCU2	V-type proton ATPase subunit E 1
Q5PQL7	Integral membrane protein 2C
M0R9L0	Nascent polypeptide-associated complex subunit alpha
P49134	Integrin beta 1
P50475	Alanine--tRNA ligase cytoplasmic
G3V712	keratin 7
Q68FQ0	T-complex protein 1 epsilon
Q810F4	Protein FAM3C
P31044	Phosphatidylethanolamine-binding protein 1
Q4QQV0	Tubulin beta-6
F1LQ81	Vesicle-fusing ATPase
Q3B7V5	Ras-related protein Rab-2b
D4A0T0	NADH dehydrogenase 1 beta subcomplex subunit 10
G3V7M2	Integrin beta 7
G3V6P8	Guanine nucleotide-binding protein gamma-12
G3V6P7	Myosin heavy chain 9

Q04970	GTPase NRas
D3ZNG3	Signal peptide peptidase-like 2A
Q9Z1J7	Amino acid transporter
E9PTW1	Secretory carrier-associated membrane protein
R9PXU4	Thioredoxin reductase 1 cytoplasmic
D4ADE8	ATP-dependent RNA helicase DDX3X
O88600	Heat shock 70 kDa protein 4
G3V9N9	alpha-1,2-Mannosidase
Q794F9	4F2 cell-surface antigen heavy chain
Q6P791	Ragulator complex protein LAMTOR1
F1LNT8	VAMP8
P61227	Ras-related protein Rap-2b
Q6P799	Serine--tRNA ligase cytoplasmic
P62703	40S ribosomal protein S4, X isoform
P62909	40S ribosomal protein S3
O35796	Complement component 1 Q subcomponent-binding protein mitochondrial
Q6AYC4	Macrophage-capping protein
Q63377	Sodium/potassium-transporting ATPase subunit beta-3
P62907	60S ribosomal protein L10a
M0RBJ0	Guanine nucleotide-binding protein gamma-2
Q63942	Ras-related protein Rab-3d
P48721	Mortalin
Q63584	Transmembrane emp24 domain-containing protein 10
P10960	Prosaposin
Q6EV76	Alpha-(1,6)-fucosyltransferase
Q5XIM9	T-complex protein 1 beta
F1M779	Clathrin heavy chain
P05065	Fructose-bisphosphate aldolase A
P32551	Cytochrome b-c1 complex subunit 2 mitochondrial
B0BN52	Mitochondrial carrier homolog 2
E9PTX9	Solute carrier family 12 member 2
Q5XFX0	Transgelin-2
F1LRK1	Sodium/potassium-transporting ATPase subunit alpha
Q7TSA0	Mitochondrial Rho GTPase 2
P61805	Dolichyl-diphosphooligosaccharide protein glycosyltransferase subunit DAD1
G3V908	keratin, type II Kb15
P53987	Monocarboxylate transporter 1
P09811	Glycogen phosphorylase liver form
G3V6N2	Transmembrane emp24 domain-containing protein 4

Q62902	Lectin mannose-binding 1
P28494	Alpha-mannosidase 2
P61131	Beta-galactoside alpha-2,3-sialyltransferase 4
Q6PDV6	40S ribosomal protein S14
P50399	Rab GDP dissociation inhibitor beta
Q6AYD3	Proliferation-associated protein 2G4
Q9WVB1	Ras-related protein Rab-6a
Q05175	Brain acid soluble protein 1
B2RYG6	Ubiquitin thioesterase OTUB1
P62278	40S ribosomal protein S13
Q3T1K5	F-actin-capping protein subunit alpha-2
P62138	Serine/threonine-protein phosphatase PP1-alpha catalytic subunit
P68370	Tubulin alpha-1A
Q5U316	Ras-related protein Rab-35
P11167	Solute carrier family 2, facilitated glucose transporter member 1
P31399	ATP synthase subunit D mitochondrial
D4A720	Serine/arginine-rich splicing factor 7 isoform 2
G3V8L7	Integrin alpha M
P04642	L-lactate dehydrogenase A chain
O35509	Ras-related protein Rab-11b
P62828	GTP-binding nuclear protein Ran
P70583	Deoxyuridine 5'-triphosphate nucleotidohydrolase
Q6AYI1	Probable ATP-dependent RNA helicase DDX5
P70580	Membrane-associated progesterone receptor component 1
P23358	60S ribosomal protein L12
Q3T1J1	Eukaryotic translation initiation factor 5A-1
F7EWC1	Vasodilator-stimulated phosphoprotein
Q66HA6	ADP-ribosylation factor-like protein 8B
P11598	Protein disulfide-isomerase A3
P62282	40S ribosomal protein S11
Q6P3V9	60S ribosomal protein L4
G3V6T1	Coatomer subunit alpha
Q6P3V8	Eukaryotic initiation factor 4A-I
Q66HA4	Tax1-binding protein 1 homolog
F1LP82	Ras-related protein Rab-2a
Q62636	Ras-related protein Rap-1b
Q3MIE4	Synaptic vesicle membrane protein VAT-1 homolog
Q6P742	Proteolipid protein 2
P04636	Malate dehydrogenase mitochondrial

P62914	60S ribosomal protein L11
P50339	Chymase 1
Q5U300	Ubiquitin-like modifier-activating enzyme 1
G3V7I0	Peroxiredoxin-3
P62815	V-type proton ATPase subunit B
Q9JID2	Guanine nucleotide-binding protein alpha-11
Q63198	Contactin 1
P11980	Pyruvate kinase PKM
Q9Z2J5	VAMP5
P10719	ATP synthase subunit beta mitochondrial
D3ZAY7	Band 4.1-like protein 2
G3V741	Solute carrier family 25 member 3
O88941	Mannosyl-oligosaccharide glucosidase
O54980	Synaptogyrin-2
Q6P6T6	Cathepsin D
Q4V7D9	Acid sphingomyelinase-like phosphodiesterase 3B
Q6S395	Plectin
F1LMP7	Granulins
Q5XI04	Erythrocyte band 7 integral membrane protein
F1LQJ7	Phosphoenolpyruvate carboxykinase [GTP], mitochondrial
Q9R1T1	Barrier-to-autointegration factor
P22734	Catechol O-methyltransferase
P62804	Histone H4
F1LM93	Tyrosine-protein kinase Yes
Q63083	Nucleobindin-1
Q9JKB7	Guanine deaminase
P12928	Pyruvate kinase PKLR
Q63081	Protein disulfide-isomerase A6
G3V9S3	Tyrosine-protein phosphatase non-receptor type 13
P13832	Myosin regulatory light chain RLC-A
B1WBY5	DnaJ homolog subfamily C member 11
Q62940	E3 ubiquitin-protein ligase NEDD4
Q62658	Peptidyl-prolyl cis-trans isomerase FKBP1A
P68255	14-3-3 protein theta
Q63803	Guanine nucleotide-binding protein G(s) subunit alpha isoforms XLas
Q6P6U0	Tyrosine-protein kinase Fgr
Q62753	Syntaxin-binding protein 2
D4A5K6	Zinc metallopeptidase STE24
P13838	Leukosialin

Q06647	ATP synthase subunit O mitochondrial
G3V637	Syntaxin-binding protein 2, isoform
P62859	40S ribosomal protein S28
Q6IN24	Galectin-8
G3V8I4	Syntaxin-4A
Q6IFV6	Keratin 35
P09895	60S ribosomal protein L5
Q63507	60S ribosomal protein L14
P10111	Peptidyl-prolyl cis-trans isomerase A
G3V679	Transferrin receptor protein 1
P62260	14-3-3 protein epsilon
Q5XIE8	Integral membrane protein 2B
B2GUZ9	Fam49b protein
Q6IG03	Keratin, type II cytoskeletal 73
Q6IG02	Keratin, type II cytoskeletal 2 epidermal
Q6IG01	Keratin, type II cytoskeletal 1B
Q9Z0V6	Thioredoxin-dependent peroxide reductase mitochondrial
Q3SWT0	Platelet endothelial cell adhesion molecule (Pecam1)
Q6IN10	Endothelin-converting enzyme 1 Isoform
Q6IG05	Keratin, type II cytoskeletal 75
Q6IFW6	Keratin, type I cytoskeletal 10
P09495	Tropomyosin alpha-4
Q5XIF6	Tubulin alpha-4A
D3ZWS0	Protein scribble homolog
P62963	Profilin-1
Q66HD0	Endoplasmin
P62250	40S ribosomal protein S16
Q91Y81	Septin-2
P70550	Ras-related protein Rab-8b
Q99P74	Ras-related protein Rab-27b
Q99P75	Ras-related protein Rab-9a
Q6IG12	Keratin, type II cytoskeletal 7
Q2IBC7	Hepatocyte growth factor receptor
P35704	Peroxiredoxin-2
Q2I6B2	V-type proton ATPase subunit A1
G3V8G5	Golgi apparatus protein 1
P62630	Elongation factor 1-alpha 1
P62775	Myotrophin
F1LMM5	Arachidonate 5-lipoxygenase

Q6P6Q2	Keratin, type II cytoskeletal 5
P62246	40S ribosomal protein S15a
D3ZNQ6	NEDD8-conjugating enzyme Ubc12
P62832	60S ribosomal protein L23
P05712	Ras-related protein Rab-2a
P62836	Ras-related protein Rap-1a
P62243	40S ribosomal protein S8
E9PSV5	Phosphoserine aminotransferase
Q9JLT0	Myosin heavy chain 10
P82995	Heat shock protein HSP 90-alpha
P42667	SEC11 homolog A
G3V8F5	Mitochondrial import receptor subunit TOM40 homolog
Q6IN37	GM2 ganglioside activator
P80254	D-dopachrome decarboxylase
P13383	Nucleolin
G3V667	Integrin alpha 6
Q63610	Tropomyosin alpha-3
Q66X93	Staphylococcal nuclease domain-containing protein 1
Q6IFZ5	Keratin, type II cytoskeletal 2 oral
Q5VLR5	Endoplasmic reticulum resident protein 44
P62986	Ubiquitin-60S ribosomal protein L40
Q6RUV5	Ras-related C3 botulinum toxin substrate 1 (Rac1)
P62982	Ubiquitin-40S ribosomal protein S27a
P82471	Guanine nucleotide-binding protein G(q) subunit alpha
B4F7C2	Tubulin beta-4A
E9PT65	Radixin
Q9EPH8	Polyadenylate-binding protein 1
Q6MG61	Chloride intracellular channel protein 1
P19944	60S acidic ribosomal protein P1
Q6TXF3	Acyl-CoA-binding protein
P19945	60S acidic ribosomal protein P0
B2RZ27	SH3 domain-binding glutamic acid-rich-like protein 3
B2RYQ2	Serine/threonine-protein phosphatase 2A activator
P11240	Cytochrome c oxidase subunit 5A mitochondrial
P11232	Thioredoxin
P05982	NAD(P)H dehydrogenase [quinone] 1
P10252	CD48 antigen
P69897	Tubulin beta-5
P62890	60S ribosomal protein L30

P63095	Guanine nucleotide-binding protein G(s) subunit alpha
P52296	Importin subunit beta-1
D3ZTJ0	Protein XRP2
Q4QRB4	Tubulin beta-3
Q6P9V9	Tubulin alpha-1B
B2GUV5	V-type proton ATPase subunit G 1
B2GV72	Carbonyl reductase 3
P18418	Calreticulin
Q6P9V1	CD81 molecule
P13221	Aspartate aminotransferase cytoplasmic
Q09073	ADP/ATP translocase 2
Q6AYA5	Transmembrane protein 106 B
P54921	Alpha-soluble NSF attachment protein (SNAP-alpha)
P25113	Phosphoglycerate mutase 1
Q3KRD8	Eukaryotic translation initiation factor 6
P09626	Potassium-transporting ATPase alpha chain 1
G3V8C4	Chloride intracellular channel protein 4
P61983	14-3-3 protein gamma
P49242	40S ribosomal protein S3a
B0BNA5	Coactosin-like protein
P02401	60S acidic ribosomal protein P2
P50137	Transketolase
P09527	Ras-related protein Rab-7a
P10888	Cytochrome c oxidase subunit 4 isoform 1 mitochondrial
Q66HG5	Transmembrane 9 superfamily member 2
Q5U3Z7	Serine hydroxymethyltransferase
P48500	Triosephosphate isomerase
P23640	Ras-related protein Rab-27a
P62747	Rho-related GTP-binding protein RhoB
O70595	ATP-binding cassette sub-family B member 6
B2GV92	Prostaglandin E synthase 3
M0RAU0	Advanced glycosylation end product-specific receptor
P62083	40S ribosomal protein S7
Q6IFU9	keratin 16
Q6P9T8	Tubulin beta-4B
Q6IFU8	Keratin, type I cytoskeletal 17
P04157	Receptor-type tyrosine-protein phosphatase C (CD45)
G3V7A5	Low density lipoprotein receptor isoform
Q6IFV3	Keratin, type I cytoskeletal 15

Q6IFV1	Keratin, type I cytoskeletal 14
P20070	NADH-cytochrome b5 reductase 3
Q5XIH7	Prohibitin 2
G3V8A5	Vacuolar protein sorting-associated protein 35
P11348	Dihydropteridine reductase
D4A465	Ragulator complex protein LAMTOR2
Q5I0G4	Glycine--tRNA ligase
Q5U1Y2	Ras-related C3 botulinum toxin substrate 2 (Rac2)
P85007	Immediate early response 3-interacting protein 1
Q00238	Intercellular adhesion molecule 1 (ICAM1)
Q5M843	2-oxoglutarate and iron-dependent oxygenase domain-containing protein 3
Q5EB77	Ras-related protein Rab-18
Q6AY18	GTP-binding protein SAR1a
D3ZIP8	Endonuclease domain containing 1
P19804	Nucleoside diphosphate kinase B
D4ADS9	EFR3 homolog A
B2GVB9	Fermt3 protein
B0BNG3	Vesicular integral-membrane protein VIP36
Q6AXT5	Ras-related protein Rab-21
Q6NYB7	Ras-related protein Rab-1a
D3ZPR0	Exportin-2
P19511	ATP synthase F(0) complex subunit B1 mitochondrial
G3V8Z1	Linker for activation of T-cells family member 2 (NTAL)
Q76GL9	Neutral amino acid transporter A
F1LNF0	Myosin heavy chain 14
P17074	40S ribosomal protein S19
Q5M7T6	V-type proton ATPase subunit D 1
P63245	Receptor of activated protein C kinase 1 (RACK1)
Q8SEZ5	Cytochrome c oxidase subunit 2
Q4KM73	UMP-CMP kinase
A1L134	Ancient ubiquitous protein 1
P46462	Transitional endoplasmic reticulum ATPase
P35171	Cytochrome c oxidase subunit 7A2
P04961	Proliferating cell nuclear antigen
Q32PX6	Rho-related GTP-binding protein RhoG
Q3KRC3	Signal recognition particle receptor alpha
Q6AXS5	Plasminogen activator inhibitor 1 RNA-binding protein
Q6AXS4	Renin receptor
P05426	60S ribosomal protein L7

D4A4T0	STIP1 homology and U box-containing protein 1
D4A0E2	NSF attachment protein gamma
P16617	Phosphoglycerate kinase 1
P45592	Cofilin-1
Q01621	Tyrosine-protein kinase LCK
F1LZX9	Integrin alpha 5
F1MAS0	keratin 32
Q9JJ19	Na(+)/H(+) exchange regulatory cofactor NHE-RF1
P08753	Guanine nucleotide-binding protein G(k) subunit alpha
D3ZPP2	ADP-ribosylation factor-like protein 8A
P63269	Actin gamma-enteric smooth muscle
P15429	Beta-enolase
Q63692	Hsp90 co-chaperone Cdc37
P97675	Ectonucleotide pyrophosphatase/phosphodiesterase family member 3
Q5XIA8	Growth hormone-inducible transmembrane protein
P0C5H9	Mesencephalic astrocyte-derived neurotrophic factor
Q5M7W6	Protein FAM234A
D4A9L2	Serine/arginine-rich splicing factor 1
P35289	Ras-related protein Rab-15
Q4FZX7	Signal recognition particle receptor beta
D3ZZR9	Peptidylprolyl isomerase
P63018	Heat shock cognate 71 kDa protein
P63012	Ras-related protein Rab-3a
P35565	Calnexin
Q9R0C5	Polypeptide N-acetylgalactosaminyltransferase 7
B0BNK1	Ras-related protein Rab-5c
P17046	Lysosome-associated membrane glycoprotein 2 (Lamp2)
G3V6D3	ATP synthase subunit beta
D3ZDR2	Charged multivesicular body protein 6
E9PTI1	V-type proton ATPase subunit H
P85108	Tubulin beta-2A
D3ZZS8	V-type proton ATPase subunit B, kidney isoform
P35435	ATP synthase subunit gamma mitochondrial
D3ZGM7	Peptidase inhibitor 16
Q08163	Adenylyl cyclase-associated protein 1
P26453	Basigin
Q5RKJ9	Ras-related protein Rab-10
Q5M860	Rho GDP dissociation inhibitor 2
Q8CGU6	Nicastrin

P63039	60 kDa heat shock protein
P14659	Heat shock-related 70 kDa protein 2
Q6IRF8	V-type proton ATPase subunit S1
P00507	Aspartate aminotransferase mitochondrial
Q3MHS9	Chaperonin-containing TCP1 subunit 6A
M0RC99	Ras-related protein Rab-5a
P35952	Low-density lipoprotein receptor
Q9JK11	Reticulon-4
P14562	Lysosome-associated membrane glycoprotein 1 (Lamp1)
Q6AXV4	Sorting and assembly machinery component 50 homolog
P14668	Annexin A5
P49432	Pyruvate dehydrogenase component E1 beta
D3ZP13	Sulfhydryl oxidase 2
Q4G075	Leukocyte elastase inhibitor
Q9Z1E1	Flotillin-1
Q6IMF3	Keratin, type II cytoskeletal 1
Q920J4	Thioredoxin-like protein 1
P24368	Peptidyl-prolyl cis-trans isomerase B
Q499P3	C1GALT1-specific chaperone 1
B0BNJ1	Sorcin
P19627	Guanine nucleotide-binding protein G(z) subunit alpha
G3V9A3	14-3-3 protein sigma
P35280	Ras-related protein Rab-8a

Annotation Cluster Enrichment Score: 26.01

ID Term	GO Term	Fold Enriched	Count	*p* -value	FDR
GO:0045184	ESTABLISHMENT OF PROTEIN LOCALIZATION	2.41	221	2.22E-36	4.35E-33

Annotated proteins

Protein Accession	Protein Identification
Q5BK85	Transmembrane emp24 domain-containing protein 1
F1M2P8	Protein kinase C alpha
Q62876	Synaptogyrin-1
Q62726	Serine/threonine-protein kinase ICK
Q5XIU4	B-cell receptor-associated protein 29
Q63716	Peroxiredoxin-1
G3V8I4	Syntaxin-4A

F1LMK0	A-kinase anchoring protein 1 isoform 2
B0BN02	Metaxin 1
P10111	Peptidyl-prolyl cis-trans isomerase A
D3ZTX0	Transmembrane emp24 domain-containing protein 7
G3V679	Transferrin receptor protein 1
P29995	Inositol 1,4,5-trisphosphate receptor type 2
P62260	14-3-3 protein epsilon
G3V6H0	Ras-related protein Rab-1b
P20417	Protein-tyrosine phosphatase 1B
P68511	14-3-3 protein eta
Q66HD0	Endoplasmin
P70550	Ras-related protein Rab-8b
Q5BJY9	Keratin, type I cytoskeletal 18
Q62991	Syntaxin-binding protein 1-like 2
Q99P75	Ras-related protein Rab-9a
Q62896	Bet1 golgi vesicular membrane trafficking protein
O88884	A-kinase anchor protein 1, mitochondrial
Q5XI41	Translocating chain-associated membrane protein 1
P27615	Lysosome membrane protein 2 (CD36)
G3V8G5	Golgi apparatus protein 1
P63102	14-3-3 protein zeta/delta
Q9EST6	Acidic leucine-rich nuclear phosphoprotein 32 family member B
Q78P65	Rap2A-like protein
D3ZUZ4	Glycosaminoglycan xylosylkinase
P05712	Ras-related protein Rab-2a
P62836	Ras-related protein Rap-1a
Q01827	Vesicular amine transporter 2
P97887	Presenilin-1
P36370	Antigen peptide transporter 1
P82995	Heat shock protein HSP 90-alpha
P52555	Endoplasmic reticulum resident protein 29
Q62611	Interleukin 1 receptor-like 1
G3V8F5	Mitochondrial import receptor subunit TOM40 homolog
Q5XI38	Lymphocyte cytosolic protein 1
Q5XID0	Protein YIPF5
Q5RKH6	Protein OS-9
P13383	Nucleolin
P05708	Hexokinase-1
P13386	High affinity immunoglobulin epsilon receptor subunit beta
M0RDI5	Mitochondrial calcium uniporter
Q07936	Annexin A2
P82471	Guanine nucleotide-binding protein G(q) subunit alpha
E9PT65	Radixin
Q498C8	Protein RER1

D4A2Z6	Translocation protein SEC63 homolog
P11232	Thioredoxin
O88767	Protein/nucleic acid deglycase DJ-1
O70257	Syntaxin-7
P35213	14-3-3 protein beta/alpha
P34058	Heat shock protein HSP 90-beta
F7FNS3	Derlin
D3ZJX5	Mitochondrial import inner membrane translocase subunit TIM50
Q66H80	Coatomer subunit delta
G3V7P1	Syntaxin-12
P10860	Glutamate dehydrogenase 1
P63095	Guanine nucleotide-binding protein G(s) subunit alpha
P52296	Importin subunit beta-1
O35165	Golgi SNAP receptor complex member 2
R9PXR4	Mitochondrial import receptor subunit TOM70
P63331	Serine/threonine-protein phosphatase 2A catalytic subunit alpha isoform
D4A1D8	Surfeit 4
D3ZGV2	Peroxisomal membrane protein PEX16
Q64244	ADP-ribosyl cyclase/cyclic ADP-ribose hydrolase 1
P18418	Calreticulin
O88777	Presenilin-2
Q63635	Syntaxin-6
Q9WU70	Syntaxin-binding protein 5
Q5I0E7	Transmembrane emp24 domain-containing protein 9
Q9Z2Q7	Syntaxin-8
P54921	Alpha-soluble NSF attachment protein (SNAP-alpha)
F1M7T6	Translocon-associated protein subunit gamma
P61983	14-3-3 protein gamma
F1LQR8	Inositol 1,4,5-trisphosphate receptor type 2
P21708	Mitogen-activated protein kinase 3
Q08849	Syntaxin-3
P09527	Ras-related protein Rab-7a
Q8CFN2	Cell division control protein 42 homolog (CDC42)
Q08851	Syntaxin-5
Q63151	Long chain fatty acid CoA ligase 3
Q9JHW5	VAMP7
Q63524	Transmembrane emp24 domain-containing protein 2
O70377	Synaptosomal-associated protein 23 (SNAP-23)
Q1H5H1	Thioredoxin reductase-like selenoprotein T
Q66HG9	Mitochondrial antiviral-signaling protein
P23640	Ras-related protein Rab-27a
Q4V8F3	Rhomboid-related protein 4
P51146	Ras-related protein Rab-4b
Q07014	Tyrosine-protein kinase Lyn

P62747	Rho-related GTP-binding protein RhoB
Q4AEF8	Coatomer subunit gamma 1
P31977	Ezrin
P07150	Annexin A1
Q6AYQ1	Golgin subfamily A member 7
F1LW77	Ras-related protein Rab-33b
Q6MG49	Large proline-rich protein BAG6
Q9Z2S9	Flotillin-2
Q5XIH7	Prohibitin 2
G3V8A5	Vacuolar protein sorting-associated protein 35
D3ZP98	Minor histocompatibility antigen H13
F1LVV4	Protein RCC2
Q5U362	Annexin A4
Q5U1Y2	Ras-related C3 botulinum toxin substrate 2 (Rac2)
B2RZD1	Protein transport protein Sec61 subunit beta
P11442	Clathrin heavy chain 1
Q6AXQ0	SUMO-activating enzyme subunit 1
Q6AY20	Cation-dependent mannose-6-phosphate receptor
D3ZMR1	Translocase of outer mitochondrial membrane 7
Q5EB77	Ras-related protein Rab-18
P61107	Ras-related protein Rab-14
Q6AY18	GTP-binding protein SAR1a
D4ADS9	EFR3 homolog A
Q6PCU4	Emerin
P61972	Nuclear transport factor 2
P49134	Integrin beta 1
F1M391	Stimulator of interferon genes protein
Q68FQ0	T-complex protein 1 epsilon
D4A781	Importin 5
P35355	Prostaglandin G/H synthase 2
E9PU78	Isoform Bcl-X(S) of Bcl-2-like protein 1
E9PU77	Synaptotagmin-like protein 3
Q6NYB7	Ras-related protein Rab-1a
D4A376	Ras-related protein Rab-12
Q6AXT5	Ras-related protein Rab-21
D3ZPR0	Exportin-2
F1LQ81	Vesicle-fusing ATPase
Q63269	Inositol 1,4,5-trisphosphate receptor type 3
P63245	Receptor of activated protein C kinase 1 (RACK1)
G3V6P7	Myosin heavy chain 9
A1L134	Ancient ubiquitous protein 1
P46462	Transitional endoplasmic reticulum ATPase
Q04970	GTPase NRas
Q4KM74	Vesicle-trafficking protein SEC22b

Q32PX6	Rho-related GTP-binding protein RhoG
D3ZD11	Signal peptidase complex subunit 2
E9PTW1	Secretory carrier-associated membrane protein
Q3KRC3	Signal recognition particle receptor alpha
O88600	Heat shock 70 kDa protein 4
D4A0E2	NSF attachment protein gamma
Q6MGA3	Antigen peptide transporter 2
P45592	Cofilin-1
Q6AXN3	Transmembrane emp24 domain-containing protein 5
F1LNT8	VAMP8
O35142	Coatomer subunit beta 2
Q4V8C2	Centromere/kinetochore protein zw10 homolog
Q9JJ19	Na(+)/H(+) exchange regulatory cofactor NHE-RF1
Q63584	Transmembrane emp24 domain-containing protein 10
P48721	Mortalin
Q63942	Ras-related protein Rab-3d
D3ZWZ1	Integrin alpha X
Q5XIM9	T-complex protein 1 beta
F1M779	Clathrin heavy chain
B0BN52	Mitochondrial carrier homolog 2
P61621	SEC61 alpha-1
Q5BK32	FAS-associated factor 2
P50398	Rab GDP dissociation inhibitor alpha
P53987	Monocarboxylate transporter 1
Q62902	Lectin mannose-binding 1
P50399	Rab GDP dissociation inhibitor beta
O35152	BET1-like protein
Q9WVB1	Ras-related protein Rab-6a
G3V6W2	Prolactin regulatory element-binding protein
P35289	Ras-related protein Rab-15
P63018	Heat shock cognate 71 kDa protein
P63012	Ras-related protein Rab-3a
Q7TP42	Translocation protein SEC62
Q5U316	Ras-related protein Rab-35
Q75Q41	Mitochondrial import receptor subunit TOM22 homolog
D3ZF97	Endoplasmic reticulum lectin 1
P17046	Lysosome-associated membrane glycoprotein 2 (Lamp2)
O70521	Regulator of G-protein signaling 19
O35509	Ras-related protein Rab-11b
F1LNC4	Vesicle transport through interaction with t-SNAREs homolog 1B
P62828	GTP-binding nuclear protein Ran
D4ABY2	Coatomer subunit gamma 2
Q3T1J1	Eukaryotic translation initiation factor 5A-1
P26453	Basigin

Q5RKJ9	Ras-related protein Rab-10
G3V6T1	Coatomer subunit alpha
F1LP82	Ras-related protein Rab-2a
P62914	60S ribosomal protein L11
Q4KLL4	Transmembrane 9 superfamily member 4
Q9Z2J5	VAMP5
P63039	60 kDa heat shock protein
Q3MHS9	Chaperonin-containing TCP1 subunit 6A
O54980	Synaptogyrin-2
M0RC99	Ras-related protein Rab-5a
Q5XI04	Erythrocyte band 7 integral membrane protein
Q68FX4	Hematopoietic cell-specific LYN substrate 1 (hcls1)
F1LQ92	Sel-1 homolog 1
P84817	Mitochondrial fission 1 protein
Q4V7D1	Translocon-associated protein subunit alpha
P14562	Lysosome-associated membrane glycoprotein 1 (Lamp1)
F1LQJ7	Phosphoenolpyruvate carboxykinase [GTP], mitochondrial
Q6AXV4	Sorting and assembly machinery component 50 homolog
P27881	Hexokinase-2
P14668	Annexin A5
Q63083	Nucleobindin-1
Q63085	Cyclic GMP-inhibited phosphodiesterase B
Q5U1Z9	Metaxin 2
Q9Z1E1	Flotillin-1
P63025	VAMP3
D3ZFK5	Signal peptidase complex subunit 1
P13832	Myosin regulatory light chain RLC-A
Q4KM97	Tyrosine-protein kinase BLK
Q62940	E3 ubiquitin-protein ligase NEDD4
B0BNJ1	Sorcin
P68255	14-3-3 protein theta
G3V9A3	14-3-3 protein sigma
Q6P6U0	Tyrosine-protein kinase Fgr
Q63803	Guanine nucleotide-binding protein G(s) subunit alpha isoforms XLas
P23514	Coatomer subunit beta 1
Q62753	Syntaxin-binding protein 2
P35280	Ras-related protein Rab-8a
G3V637	Syntaxin-binding protein 2, isoform

ID Term	GO Term	Fold Enriched	Count	*p* -value	FDR
**GO:0008104**	**PROTEIN LOCALIZATION**	**2.20**	**256**	**3.09E-36**	**6.05E-33**
**Annotated proteins**					

Protein Accession	Protein Identification
Q5BK85	Transmembrane emp24 domain-containing protein 1
Q6P503	V-type proton ATPase subunit D
F1M2P8	Protein kinase C alpha
Q62876	Synaptogyrin-1
Q62726	Serine/threonine-protein kinase ICK
Q5XIU4	B-cell receptor-associated protein 29
Q63716	Peroxiredoxin-1
G3V8I4	Syntaxin-4A
Q5U204	Ragulator complex protein LAMTOR3
F1LMK0	A-kinase anchoring protein 1 isoform 2
G3V852	Talin-1
B0BN02	Metaxin 1
P10111	Peptidyl-prolyl cis-trans isomerase A
P13084	Nucleophosmin
D3ZTX0	Transmembrane emp24 domain-containing protein 7
G3V679	Transferrin receptor protein 1
P29995	Inositol 1,4,5-trisphosphate receptor type 2
P62260	14-3-3 protein epsilon
F1M8X9	Golgi brefeldin A-resistant guanine nucleotide exchange factor 1
Q9Z270	VAMP-associated protein A
G3V6H0	Ras-related protein Rab-1b
D3ZWS0	Protein scribble homolog
P20417	Protein-tyrosine phosphatase 1B
P68511	14-3-3 protein eta
Q66HD0	Endoplasmin
P20411	High affinity immunoglobulin epsilon receptor subunit gamma
Q5BJY9	Keratin, type I cytoskeletal 18
P70550	Ras-related protein Rab-8b
Q62896	Bet1 golgi vesicular membrane trafficking protein
Q99P75	Ras-related protein Rab-9a
Q62991	Syntaxin-binding protein 1-like 2
O88884	A-kinase anchor protein 1, mitochondrial
Q5XI41	Translocating chain-associated membrane protein 1
P27615	Lysosome membrane protein 2 (CD36)
G3V8G5	Golgi apparatus protein 1
P11762	Galectin-1
P06761	Endoplasmic reticulum chaperone BiP
P63102	14-3-3 protein zeta/delta
Q9EST6	Acidic leucine-rich nuclear phosphoprotein 32 family member B
Q78P65	Rap2A-like protein
D3ZUZ4	Glycosaminoglycan xylosylkinase
P05712	Ras-related protein Rab-2a
P62836	Ras-related protein Rap-1a

Q1WIM3	Cell adhesion molecule 3
Q01827	Vesicular amine transporter 2
P97887	Presenilin-1
P36370	Antigen peptide transporter 1
P82995	Heat shock protein HSP 90-alpha
P52555	Endoplasmic reticulum resident protein 29
Q62611	Interleukin 1 receptor-like 1
G3V8F5	Mitochondrial import receptor subunit TOM40 homolog
Q5XI38	Lymphocyte cytosolic protein 1
Q5XID0	Protein YIPF5
Q5RKH6	Protein OS-9
P13383	Nucleolin
P05708	Hexokinase-1
P13386	High affinity immunoglobulin epsilon receptor subunit beta
M0RDI5	Mitochondrial calcium uniporter
Q6RUV5	Ras-related C3 botulinum toxin substrate 1 (Rac1)
Q07936	Annexin A2
Q5XID7	Armadillo repeat-containing X-linked protein 3
P82471	Guanine nucleotide-binding protein G(q) subunit alpha
E9PT65	Radixin
Q498C8	Protein RER1
D4A2Z6	Translocation protein SEC63 homolog
P11232	Thioredoxin
O88767	Protein/nucleic acid deglycase DJ-1
O70257	Syntaxin-7
P62329	Thymosin beta-4
P35213	14-3-3 protein beta/alpha
P34058	Heat shock protein HSP 90-beta
F7FNS3	Derlin
Q642E3	CDK5 regulatory subunit-associated protein 3, isoform
D3ZJX5	Mitochondrial import inner membrane translocase subunit TIM50
Q66H80	Coatomer subunit delta
G3V7P1	Syntaxin-12
P10860	Glutamate dehydrogenase 1
P63095	Guanine nucleotide-binding protein G(s) subunit alpha
G3V7Q7	Ras GTPase-activating-like protein IQGAP1
P52296	Importin subunit beta-1
O35165	Golgi SNAP receptor complex member 2
R9PXR4	Mitochondrial import receptor subunit TOM70
P63331	Serine/threonine-protein phosphatase 2A catalytic subunit alpha isoform
D4A1D8	Surfeit 4
D3ZGV2	Peroxisomal membrane protein PEX16
Q5PQX1	Torsin-1A-interacting protein 1
Q64244	ADP-ribosyl cyclase/cyclic ADP-ribose hydrolase 1

P18418	Calreticulin
Q6P9V1	CD81 molecule
Q63635	Syntaxin-6
O88777	Presenilin-2
Q9WU70	Syntaxin-binding protein 5
Q5I0E7	Transmembrane emp24 domain-containing protein 9
Q9Z2Q7	Syntaxin-8
P54921	Alpha-soluble NSF attachment protein (SNAP-alpha)
F1M7T6	Translocon-associated protein subunit gamma
P61983	14-3-3 protein gamma
F1LQR8	Inositol 1,4,5-trisphosphate receptor type 2
P21708	Mitogen-activated protein kinase 3
Q08849	Syntaxin-3
Q8CFN2	Cell division control protein 42 homolog (CDC42)
P09527	Ras-related protein Rab-7a
Q08851	Syntaxin-5
Q63151	Long chain fatty acid CoA ligase 3
Q9JHW5	VAMP7
Q63524	Transmembrane emp24 domain-containing protein 2
O70377	Synaptosomal-associated protein 23 (SNAP-23)
Q1H5H1	Thioredoxin reductase-like selenoprotein T
Q66HG9	Mitochondrial antiviral-signaling protein
Q68FP1	Gelsolin
P23640	Ras-related protein Rab-27a
Q4V8F3	Rhomboid-related protein 4
P51146	Ras-related protein Rab-4b
Q07014	Tyrosine-protein kinase Lyn
P62747	Rho-related GTP-binding protein RhoB
Q4AEF8	Coatomer subunit gamma 1
M0RAU0	Advanced glycosylation end product-specific receptor
P31977	Ezrin
P07150	Annexin A1
Q6AYQ1	Golgin subfamily A member 7
B2RYB8	Integrin beta 2
F1LW77	Ras-related protein Rab-33b
Q6MG49	Large proline-rich protein BAG6
Q9Z2S9	Flotillin-2
Q5XIH7	Prohibitin 2
G3V8A5	Vacuolar protein sorting-associated protein 35
Q5BQE6	Delta(24)-sterol reductase
D3ZP98	Minor histocompatibility antigen H13
F1LVV4	Protein RCC2
D4A465	Ragulator complex protein LAMTOR2
Q5U362	Annexin A4

Q5U1Y2	Ras-related C3 botulinum toxin substrate 2 (Rac2)
B2RZD1	Protein transport protein Sec61 subunit beta
P11442	Clathrin heavy chain 1
Q6AXQ0	SUMO-activating enzyme subunit 1
Q6AY20	Cation-dependent mannose-6-phosphate receptor
D3ZMR1	Translocase of outer mitochondrial membrane 7
Q5EB77	Ras-related protein Rab-18
P61107	Ras-related protein Rab-14
Q6AY18	GTP-binding protein SAR1a
D4ADS9	EFR3 homolog A
Q6PCU4	Emerin
P61972	Nuclear transport factor 2
P49134	Integrin beta 1
P97546	Neuroplastin
F1M391	Stimulator of interferon genes protein
Q68FQ0	T-complex protein 1 epsilon
D4A781	Importin 5
P35355	Prostaglandin G/H synthase 2
E9PU78	Isoform Bcl-X(S) of Bcl-2-like protein 1
E9PU77	Synaptotagmin-like protein 3
Q6NYB7	Ras-related protein Rab-1a
D4A376	Ras-related protein Rab-12
Q6AXT5	Ras-related protein Rab-21
Q9JLH7	CDK5 regulatory subunit-associated protein 3
D3ZPR0	Exportin-2
F1LQ81	Vesicle-fusing ATPase
Q63269	Inositol 1,4,5-trisphosphate receptor type 3
G3V7M2	Integrin beta 7
P63245	Receptor of activated protein C kinase 1 (RACK1)
A1L134	Ancient ubiquitous protein 1
G3V6P7	Myosin heavy chain 9
P46462	Transitional endoplasmic reticulum ATPase
Q04970	GTPase NRas
Q4KM74	Vesicle-trafficking protein SEC22b
Q32PX6	Rho-related GTP-binding protein RhoG
D3ZD11	Signal peptidase complex subunit 2
E9PTW1	Secretory carrier-associated membrane protein
Q3KRC3	Signal recognition particle receptor alpha
D4ADE8	ATP-dependent RNA helicase DDX3X
O88600	Heat shock 70 kDa protein 4
D4A0E2	NSF attachment protein gamma
Q6MGA3	Antigen peptide transporter 2
P45592	Cofilin-1
Q6AXN3	Transmembrane emp24 domain-containing protein 5

F1LNT8	VAMP8
Q6P791	Ragulator complex protein LAMTOR1
O35142	Coatomer subunit beta 2
Q63377	Sodium/potassium-transporting ATPase subunit beta-3
Q4V8C2	Centromere/kinetochore protein zw10 homolog
D3ZF11	Ragulator complex protein LAMTOR5
Q9JJ19	Na(+)/H(+) exchange regulatory cofactor NHE-RF1
Q63584	Transmembrane emp24 domain-containing protein 10
P48721	Mortalin
Q63942	Ras-related protein Rab-3d
D3ZWZ1	Integrin alpha X
Q5XIM9	T-complex protein 1 beta
F1M779	Clathrin heavy chain
B0BN52	Mitochondrial carrier homolog 2
P61621	SEC61 alpha-1
Q5BK32	FAS-associated factor 2
P50398	Rab GDP dissociation inhibitor alpha
P53987	Monocarboxylate transporter 1
Q62902	Lectin mannose-binding 1
P50399	Rab GDP dissociation inhibitor beta
O35152	BET1-like protein
Q9WVB1	Ras-related protein Rab-6a
G3V6W2	Prolactin regulatory element-binding protein
P35289	Ras-related protein Rab-15
P63018	Heat shock cognate 71 kDa protein
P63012	Ras-related protein Rab-3a
Q7TP42	Translocation protein SEC62
Q5U316	Ras-related protein Rab-35
Q75Q41	Mitochondrial import receptor subunit TOM22 homolog
D3ZF97	Endoplasmic reticulum lectin 1
Q9Z1Z1	Eukaryotic translation initiation factor 2-alpha kinase 3
P17046	Lysosome-associated membrane glycoprotein 2 (Lamp2)
O70521	Regulator of G-protein signaling 19
O35509	Ras-related protein Rab-11b
Q66HR2	Microtubule-associated protein RP/EB family member 1
F1LNC4	Vesicle transport through interaction with t-SNAREs homolog 1B
P62828	GTP-binding nuclear protein Ran
D4ABY2	Coatomer subunit gamma 2
Q3T1J1	Eukaryotic translation initiation factor 5A-1
P26453	Basigin
Q5RKJ9	Ras-related protein Rab-10
G3V6T1	Coatomer subunit alpha
F1LP82	Ras-related protein Rab-2a
Q5XIP2	GPI-anchor transamidase

P62914	60S ribosomal protein L11
Q4KLL4	Transmembrane 9 superfamily member 4
Q9Z2J5	VAMP5
P63039	60 kDa heat shock protein
Q3MHS9	Chaperonin-containing TCP1 subunit 6A
O54980	Synaptogyrin-2
M0RC99	Ras-related protein Rab-5a
Q5XI04	Erythrocyte band 7 integral membrane protein
Q68FX4	Hematopoietic cell-specific LYN substrate 1 (hcls1)
F1LQ92	Sel-1 homolog 1
P84817	Mitochondrial fission 1 protein
Q4V7D1	Translocon-associated protein subunit alpha
P14562	Lysosome-associated membrane glycoprotein 1 (Lamp1)
F1LQJ7	Phosphoenolpyruvate carboxykinase [GTP], mitochondrial
Q63486	Ras-related GTP-binding protein A (Rag A)
Q63483	Ras-related protein Rab-38
Q6AXV4	Sorting and assembly machinery component 50 homolog
P27881	Hexokinase-2
P14668	Annexin A5
Q63083	Nucleobindin-1
Q63085	Cyclic GMP-inhibited phosphodiesterase B
Q5U1Z9	Metaxin 2
Q9Z1E1	Flotillin-1
P63025	VAMP3
D3ZFK5	Signal peptidase complex subunit 1
P13832	Myosin regulatory light chain RLC-A
Q4KM97	Tyrosine-protein kinase BLK
Q62940	E3 ubiquitin-protein ligase NEDD4
B0BNJ1	Sorcin
P68255	14-3-3 protein theta
G3V9A3	14-3-3 protein sigma
Q63803	Guanine nucleotide-binding protein G(s) subunit alpha isoforms XLas
Q6P6U0	Tyrosine-protein kinase Fgr
F1M2K3	Small ubiquitin-related modifier
P23514	Coatomer subunit beta 1
Q62753	Syntaxin-binding protein 2
P35280	Ras-related protein Rab-8a
G3V637	Syntaxin-binding protein 2, isoform

ID Term	GO Term	Fold Enriched	Count	*p* -value	FDR
**GO:0033036**	**MACROMOLECULE LOCALIZATION**	**2.08**	**279**	**6.90E-35**	**1.35E-31**
**Annotated proteins**					

Protein Accession	Protein Identification
Q5BK85	Transmembrane emp24 domain-containing protein 1
Q6P503	V-type proton ATPase subunit D
F1M2P8	Protein kinase C alpha
Q62876	Synaptogyrin-1
Q62726	Serine/threonine-protein kinase ICK
Q5XIU4	B-cell receptor-associated protein 29
Q63716	Peroxiredoxin-1
G3V8I4	Syntaxin-4A
Q5U204	Ragulator complex protein LAMTOR3
F1LMK0	A-kinase anchoring protein 1 isoform 2
G3V852	Talin-1
B0BN02	Metaxin 1
P13084	Nucleophosmin
P10111	Peptidyl-prolyl cis-trans isomerase A
D3ZTX0	Transmembrane emp24 domain-containing protein 7
G3V679	Transferrin receptor protein 1
P29995	Inositol 1,4,5-trisphosphate receptor type 2
Q4FZT0	Stomatin-like protein 2
P62260	14-3-3 protein epsilon
F1M8X9	Golgi brefeldin A-resistant guanine nucleotide exchange factor 1
Q9Z270	VAMP-associated protein A
Q10468	Beta-1,4 N-acetylgalactosaminyltransferase 1
G3V6H0	Ras-related protein Rab-1b
D3ZWS0	Protein scribble homolog
P20417	Protein-tyrosine phosphatase 1B
P68511	14-3-3 protein eta
Q66HD0	Endoplasmin
P20411	High affinity immunoglobulin epsilon receptor subunit gamma
Q5BJY9	Keratin, type I cytoskeletal 18
P70550	Ras-related protein Rab-8b
Q62896	Bet1 golgi vesicular membrane trafficking protein
Q99P75	Ras-related protein Rab-9a
Q62991	Syntaxin-binding protein 1-like 2
O88884	A-kinase anchor protein 1, mitochondrial
Q5XI41	Translocating chain-associated membrane protein 1
P27615	Lysosome membrane protein 2 (CD36)
G3V8G5	Golgi apparatus protein 1
P11762	Galectin-1
P06761	Endoplasmic reticulum chaperone BiP
P63102	14-3-3 protein zeta/delta
Q9EST6	Acidic leucine-rich nuclear phosphoprotein 32 family member B
Q78P65	Rap2A-like protein
D3ZUZ4	Glycosaminoglycan xylosylkinase

D4A6A2	Heterogeneous nuclear ribonucleoprotein A3-like
P05712	Ras-related protein Rab-2a
M0RCF3	Integrin beta 3
P62836	Ras-related protein Rap-1a
Q1WIM3	Cell adhesion molecule 3
Q01827	Vesicular amine transporter 2
Q3B8Q2	Eukaryotic initiation factor 4A-III
Q63413	Spliceosome RNA helicase Ddx39b
P97887	Presenilin-1
P36370	Antigen peptide transporter 1
P82995	Heat shock protein HSP 90-alpha
P52555	Endoplasmic reticulum resident protein 29
Q62611	Interleukin 1 receptor-like 1
G3V8F5	Mitochondrial import receptor subunit TOM40 homolog
Q5XI38	Lymphocyte cytosolic protein 1
Q6IN37	GM2 ganglioside activator
Q5XID0	Protein YIPF5
Q5RKH6	Protein OS-9
P13383	Nucleolin
P05708	Hexokinase-1
P13386	High affinity immunoglobulin epsilon receptor subunit beta
M0RDI5	Mitochondrial calcium uniporter
Q6RUV5	Ras-related C3 botulinum toxin substrate 1 (Rac1)
Q07936	Annexin A2
Q5XID7	Armadillo repeat-containing X-linked protein 3
P82471	Guanine nucleotide-binding protein G(q) subunit alpha
E9PT65	Radixin
Q498C8	Protein RER1
D4A2Z6	Translocation protein SEC63 homolog
P11232	Thioredoxin
O88767	Protein/nucleic acid deglycase DJ-1
O70257	Syntaxin-7
P62329	Thymosin beta-4
P35213	14-3-3 protein beta/alpha
P34058	Heat shock protein HSP 90-beta
F7FNS3	Derlin
Q642E3	CDK5 regulatory subunit-associated protein 3, isoform
D3ZJX5	Mitochondrial import inner membrane translocase subunit TIM50
Q66H80	Coatomer subunit delta
G3V7P1	Syntaxin-12
P10860	Glutamate dehydrogenase 1
P63095	Guanine nucleotide-binding protein G(s) subunit alpha
G3V7Q7	Ras GTPase-activating-like protein IQGAP1
P52296	Importin subunit beta-1

O35165	Golgi SNAP receptor complex member 2
P63331	Serine/threonine-protein phosphatase 2A catalytic subunit alpha isoform
R9PXR4	Mitochondrial import receptor subunit TOM70
D4A1D8	Surfeit 4
D3ZGV2	Peroxisomal membrane protein PEX16
Q5PQX1	Torsin-1A-interacting protein 1
Q64244	ADP-ribosyl cyclase/cyclic ADP-ribose hydrolase 1
P18418	Calreticulin
Q6P9V1	CD81 molecule
Q63635	Syntaxin-6
O88777	Presenilin-2
Q9WU70	Syntaxin-binding protein 5
Q5I0E7	Transmembrane emp24 domain-containing protein 9
Q9Z2Q7	Syntaxin-8
F1LM33	Leucine-rich PPR motif-containing protein mitochondrial isoform
P38983	40S ribosomal protein SA
P54921	Alpha-soluble NSF attachment protein (SNAP-alpha)
F1M7T6	Translocon-associated protein subunit gamma
P61983	14-3-3 protein gamma
F1LQR8	Inositol 1,4,5-trisphosphate receptor type 2
P21708	Mitogen-activated protein kinase 3
Q08849	Syntaxin-3
Q8CFN2	Cell division control protein 42 homolog (CDC42)
P09527	Ras-related protein Rab-7a
Q08851	Syntaxin-5
Q63151	Long chain fatty acid CoA ligase 3
P58195	Phospholipid scramblase 1
Q9JHW5	VAMP7
Q63524	Transmembrane emp24 domain-containing protein 2
Q9JI85	Nucleobindin-2
Q5SGE0	Leucine-rich PPR motif-containing protein mitochondrial
Q66HG9	Mitochondrial antiviral-signaling protein
O70377	Synaptosomal-associated protein 23 (SNAP-23)
Q1H5H1	Thioredoxin reductase-like selenoprotein T
Q68FP1	Gelsolin
P23640	Ras-related protein Rab-27a
Q4V8F3	Rhomboid-related protein 4
P51146	Ras-related protein Rab-4b
Q07014	Tyrosine-protein kinase Lyn
P62747	Rho-related GTP-binding protein RhoB
Q4AEF8	Coatomer subunit gamma 1
M0RAU0	Advanced glycosylation end product-specific receptor
P31977	Ezrin
P07150	Annexin A1

G3V7A5	Low density lipoprotein receptor isoform
Q6AYQ1	Golgin subfamily A member 7
B2RYB8	Integrin beta 2
F1LW77	Ras-related protein Rab-33b
Q6MG49	Large proline-rich protein BAG6
Q9Z2S9	Flotillin-2
Q5XIH7	Prohibitin 2
G3V8A5	Vacuolar protein sorting-associated protein 35
Q5BQE6	Delta(24)-sterol reductase
D3ZP98	Minor histocompatibility antigen H13
F1LVV4	Protein RCC2
D4A465	Ragulator complex protein LAMTOR2
Q5U362	Annexin A4
Q5U1Y2	Ras-related C3 botulinum toxin substrate 2 (Rac2)
B2RZD1	Protein transport protein Sec61 subunit beta
F1LM82	Heterogeneous nuclear ribonucleoproteins A2/B1
P11442	Clathrin heavy chain 1
Q6AXQ0	SUMO-activating enzyme subunit 1
Q6AY20	Cation-dependent mannose-6-phosphate receptor
D3ZMR1	Translocase of outer mitochondrial membrane 7
Q5EB77	Ras-related protein Rab-18
P61107	Ras-related protein Rab-14
Q6AY18	GTP-binding protein SAR1a
D4ADS9	EFR3 homolog A
Q6PCU4	Emerin
P61972	Nuclear transport factor 2
P49134	Integrin beta 1
P97546	Neuroplastin
F1M391	Stimulator of interferon genes protein
Q68FQ0	T-complex protein 1 epsilon
D4A781	Importin 5
P35355	Prostaglandin G/H synthase 2
E9PU78	Isoform Bcl-X(S) of Bcl-2-like protein 1
E9PU77	Synaptotagmin-like protein 3
Q6AXT5	Ras-related protein Rab-21
Q6NYB7	Ras-related protein Rab-1a
D4A376	Ras-related protein Rab-12
Q9JLH7	CDK5 regulatory subunit-associated protein 3
D3ZPR0	Exportin-2
F1LQ81	Vesicle-fusing ATPase
Q63269	Inositol 1,4,5-trisphosphate receptor type 3
G3V7M2	Integrin beta 7
P63245	Receptor of activated protein C kinase 1 (RACK1)
A1L134	Ancient ubiquitous protein 1

G3V6P7	Myosin heavy chain 9
Q4KM74	Vesicle-trafficking protein SEC22b
P46462	Transitional endoplasmic reticulum ATPase
Q04970	GTPase NRas
Q32PX6	Rho-related GTP-binding protein RhoG
D3ZD11	Signal peptidase complex subunit 2
E9PTW1	Secretory carrier-associated membrane protein
Q3KRC3	Signal recognition particle receptor alpha
D4ADE8	ATP-dependent RNA helicase DDX3X
O88600	Heat shock 70 kDa protein 4
D4A0E2	NSF attachment protein gamma
Q6MGA3	Antigen peptide transporter 2
P45592	Cofilin-1
Q6AXN3	Transmembrane emp24 domain-containing protein 5
F1LNT8	VAMP8
Q6P791	Ragulator complex protein LAMTOR1
F1LZX9	Integrin alpha 5
O35142	Coatomer subunit beta 2
Q63377	Sodium/potassium-transporting ATPase subunit beta-3
Q4V8C2	Centromere/kinetochore protein zw10 homolog
D3ZF11	Ragulator complex protein LAMTOR5
Q9JJ19	Na(+)/H(+) exchange regulatory cofactor NHE-RF1
Q63584	Transmembrane emp24 domain-containing protein 10
P48721	Mortalin
Q63942	Ras-related protein Rab-3d
P10960	Prosaposin
D3ZWZ1	Integrin alpha X
Q5XIM9	T-complex protein 1 beta
F1M779	Clathrin heavy chain
B0BN52	Mitochondrial carrier homolog 2
P61621	SEC61 alpha-1
Q5BK32	FAS-associated factor 2
P50398	Rab GDP dissociation inhibitor alpha
P53987	Monocarboxylate transporter 1
O88813	Long chain fatty acid CoA ligase 5
Q62902	Lectin mannose-binding 1
P50399	Rab GDP dissociation inhibitor beta
O35152	BET1-like protein
Q9WVB1	Ras-related protein Rab-6a
G3V6W2	Prolactin regulatory element-binding protein
P35289	Ras-related protein Rab-15
P63018	Heat shock cognate 71 kDa protein
P63012	Ras-related protein Rab-3a
Q7TP42	Translocation protein SEC62

Q5U316	Ras-related protein Rab-35
Q75Q41	Mitochondrial import receptor subunit TOM22 homolog
D3ZF97	Endoplasmic reticulum lectin 1
Q9Z1Z1	Eukaryotic translation initiation factor 2-alpha kinase 3
P17046	Lysosome-associated membrane glycoprotein 2 (Lamp2)
O35509	Ras-related protein Rab-11b
O70521	Regulator of G-protein signaling 19
Q66HR2	Microtubule-associated protein RP/EB family member 1
F1LNC4	Vesicle transport through interaction with t-SNAREs homolog 1B
P62828	GTP-binding nuclear protein Ran
D4ABY2	Coatomer subunit gamma 2
Q3T1J1	Eukaryotic translation initiation factor 5A-1
P26453	Basigin
Q5RKJ9	Ras-related protein Rab-10
G3V6T1	Coatomer subunit alpha
F1LP82	Ras-related protein Rab-2a
Q5XIP2	GPI-anchor transamidase
P62914	60S ribosomal protein L11
Q4KLL4	Transmembrane 9 superfamily member 4
Q9Z2J5	VAMP5
P63039	60 kDa heat shock protein
Q6URK4	Heterogeneous nuclear ribonucleoprotein A3
P00507	Aspartate aminotransferase mitochondrial
Q3MHS9	Chaperonin-containing TCP1 subunit 6A
O54980	Synaptogyrin-2
M0RC99	Ras-related protein Rab-5a
Q5XI04	Erythrocyte band 7 integral membrane protein
Q68FX4	Hematopoietic cell-specific LYN substrate 1 (hcls1)
P35952	Low-density lipoprotein receptor
Q9Z1X1	Extended synaptotagmin 1
F1LQ92	Sel-1 homolog 1
P84817	Mitochondrial fission 1 protein
Q4V7D1	Translocon-associated protein subunit alpha
P14562	Lysosome-associated membrane glycoprotein 1 (Lamp1)
F1LQJ7	Phosphoenolpyruvate carboxykinase [GTP], mitochondrial
Q63486	Ras-related GTP-binding protein A (Rag A)
Q63483	Ras-related protein Rab-38
Q6AXV4	Sorting and assembly machinery component 50 homolog
P27881	Hexokinase-2
P14668	Annexin A5
Q2YDU8	Protein spinster homolog 1
Q63083	Nucleobindin-1
Q63085	Cyclic GMP-inhibited phosphodiesterase B
Q5U1Z9	Metaxin 2

Q9Z1E1	Flotillin-1
P63025	VAMP3
D3ZFK5	Signal peptidase complex subunit 1
P13832	Myosin regulatory light chain RLC-A
Q4KM97	Tyrosine-protein kinase BLK
Q62940	E3 ubiquitin-protein ligase NEDD4
B0BNJ1	Sorcin
P68255	14-3-3 protein theta
G3V9A3	14-3-3 protein sigma
Q63803	Guanine nucleotide-binding protein G(s) subunit alpha isoforms XLas
Q6P6U0	Tyrosine-protein kinase Fgr
F1M2K3	Small ubiquitin-related modifier
P23514	Coatomer subunit beta 1
Q62753	Syntaxin-binding protein 2
P35280	Ras-related protein Rab-8a
G3V637	Syntaxin-binding protein 2, isoform

Annotation Cluster Enrichment Score: 17.93

ID Term	GO Term	Fold Enriched	Count	*p* -value	FDR
**GO:0098857**	**MEMBRANE MICRODOMAIN/RAFT**	**3.67**	**65**	**5.09E-19**	**7.88E-16**

Annotated proteins

Protein Accession	Protein Identification
D4A465	Ragulator complex protein LAMTOR2
Q9Z2L0	Voltage-dependent anion-selective channel protein 1
P63018	Heat shock cognate 71 kDa protein
B5DEH2	Erlin-2
F1M2P8	Protein kinase C alpha
Q00238	Intercellular adhesion molecule 1 (ICAM1)
Q5U204	Ragulator complex protein LAMTOR3
O88767	Protein/nucleic acid deglycase DJ-1
P11167	Solute carrier family 2, facilitated glucose transporter member 1
P10252	CD48 antigen
P17046	Lysosome-associated membrane glycoprotein 2 (Lamp2)
O70521	Regulator of G-protein signaling 19
P06686	Sodium/potassium-transporting ATPase subunit alpha-2
O08775	Vascular endothelial growth factor receptor 2
P06685	Sodium/potassium-transporting ATPase subunit alpha-1
G3V7P1	Syntaxin-12
Q4FZT0	Stomatin-like protein 2
P49134	Integrin beta 1

P09456	cAMP-dependent protein kinase type I-alpha regulatory subunit
P63095	Guanine nucleotide-binding protein G(s) subunit alpha
G3V7Q7	Ras GTPase-activating-like protein IQGAP1
Q3SWT0	Platelet endothelial cell adhesion molecule (Pecam1)
P35355	Prostaglandin G/H synthase 2
P26453	Basigin
Q62636	Ras-related protein Rap-1b
P81155	Voltage-dependent anion-selective channel protein 2
G3V8Z1	Linker for activation of T-cells family member 2 (NTAL)
P12368	cAMP-dependent protein kinase type II-alpha regulatory subunit
P08644	GTPase KRas
Q07647	Solute carrier family 2, facilitated glucose transporter member 3
Q63198	Contactin 1
P63039	60 kDa heat shock protein
A3RLA8	Low affinity immunoglobulin gamma Fc region receptor II
P21708	Mitogen-activated protein kinase 3
P04897	Guanine nucleotide-binding protein G(i) subunit alpha-2
P06762	Heme oxygenase 1
P16617	Phosphoglycerate kinase 1
Q01621	Tyrosine-protein kinase LCK
Q6P6T6	Cathepsin D
Q6P791	Ragulator complex protein LAMTOR1
M0RC99	Ras-related protein Rab-5a
Q5XI04	Erythrocyte band 7 integral membrane protein
P58195	Phospholipid scramblase 1
P35952	Low-density lipoprotein receptor
P61227	Ras-related protein Rap-2b
P01830	Thy-1 membrane glycoprotein
D3ZF11	Ragulator complex protein LAMTOR5
Q63377	Sodium/potassium-transporting ATPase subunit beta-3
Q9JJ19	Na(+)/H(+) exchange regulatory cofactor NHE-RF1
Q07014	Tyrosine-protein kinase Lyn
P08753	Guanine nucleotide-binding protein G(k) subunit alpha
P97887	Presenilin-1
P31977	Ezrin
Q9Z1E1	Flotillin-1
P05708	Hexokinase-1
P13386	High affinity immunoglobulin epsilon receptor subunit beta
P04157	Receptor-type tyrosine-protein phosphatase C (CD45)
G3V7A5	Low density lipoprotein receptor isoform
Q62940	E3 ubiquitin-protein ligase NEDD4
B2RYB8	Integrin beta 2
Q07936	Annexin A2
Q63803	Guanine nucleotide-binding protein G(s) subunit alpha isoforms XLas

P82471	Guanine nucleotide-binding protein G(q) subunit alpha
P05197	Elongation factor 2
Q9Z2S9	Flotillin-2

ID Term	GO Term	Fold Enriched	Count	*p* -value	FDR
**GO:0098589**	**MEMBRANE REGION**	**3.33**	**69**	**6.04E-18**	**9.35E-15**
**Annotated proteins**					

Protein Accession	Protein Identification
D4A465	Ragulator complex protein LAMTOR2
Q9Z2L0	Voltage-dependent anion-selective channel protein 1
P63018	Heat shock cognate 71 kDa protein
B5DEH2	Erlin-2
F1M2P8	Protein kinase C alpha
P11442	Clathrin heavy chain 1
Q00238	Intercellular adhesion molecule 1 (ICAM1)
Q5U204	Ragulator complex protein LAMTOR3
O88767	Protein/nucleic acid deglycase DJ-1
Q5U316	Ras-related protein Rab-35
P11167	Solute carrier family 2, facilitated glucose transporter member 1
P10252	CD48 antigen
P17046	Lysosome-associated membrane glycoprotein 2 (Lamp2)
O70521	Regulator of G-protein signaling 19
G3V679	Transferrin receptor protein 1
P06686	Sodium/potassium-transporting ATPase subunit alpha-2
O08775	Vascular endothelial growth factor receptor 2
P06685	Sodium/potassium-transporting ATPase subunit alpha-1
G3V7P1	Syntaxin-12
Q4FZT0	Stomatin-like protein 2
P49134	Integrin beta 1
P09456	cAMP-dependent protein kinase type I-alpha regulatory subunit
P63095	Guanine nucleotide-binding protein G(s) subunit alpha
G3V7Q7	Ras GTPase-activating-like protein IQGAP1
Q3SWT0	Platelet endothelial cell adhesion molecule (Pecam1)
P35355	Prostaglandin G/H synthase 2
Q3T1J1	Eukaryotic translation initiation factor 5A-1
P26453	Basigin
Q62636	Ras-related protein Rap-1b
P81155	Voltage-dependent anion-selective channel protein 2
G3V8Z1	Linker for activation of T-cells family member 2 (NTAL)
P12368	cAMP-dependent protein kinase type II-alpha regulatory subunit
P08644	GTPase KRas
Q07647	Solute carrier family 2, facilitated glucose transporter member 3

Q63198	Contactin 1
P63039	60 kDa heat shock protein
A3RLA8	Low affinity immunoglobulin gamma Fc region receptor II
P21708	Mitogen-activated protein kinase 3
P04897	Guanine nucleotide-binding protein G(i) subunit alpha-2
P06762	Heme oxygenase 1
P16617	Phosphoglycerate kinase 1
Q01621	Tyrosine-protein kinase LCK
Q6P6T6	Cathepsin D
Q6P791	Ragulator complex protein LAMTOR1
M0RC99	Ras-related protein Rab-5a
Q5XI04	Erythrocyte band 7 integral membrane protein
P58195	Phospholipid scramblase 1
P35952	Low-density lipoprotein receptor
P61227	Ras-related protein Rap-2b
P01830	Thy-1 membrane glycoprotein
D3ZF11	Ragulator complex protein LAMTOR5
Q63377	Sodium/potassium-transporting ATPase subunit beta-3
Q9JJ19	Na(+)/H(+) exchange regulatory cofactor NHE-RF1
Q07014	Tyrosine-protein kinase Lyn
P08753	Guanine nucleotide-binding protein G(k) subunit alpha
P97887	Presenilin-1
P31977	Ezrin
F1M779	Clathrin heavy chain
Q9Z1E1	Flotillin-1
P05708	Hexokinase-1
P13386	High affinity immunoglobulin epsilon receptor subunit beta
P04157	Receptor-type tyrosine-protein phosphatase C (CD45)
G3V7A5	Low density lipoprotein receptor isoform
Q62940	E3 ubiquitin-protein ligase NEDD4
B2RYB8	Integrin beta 2
Q07936	Annexin A2
Q63803	Guanine nucleotide-binding protein G(s) subunit alpha isoforms XLas
P82471	Guanine nucleotide-binding protein G(q) subunit alpha
P05197	Elongation factor 2
Q9Z2S9	Flotillin-2

Annotation Cluster Enrichment Score: 16.14

ID Term	GO Term	Fold Enriched	Count	*p* -value	FDR
UP_KEYWORDS	MEMBRANE	1.54	510	1.41E-33	1.92E-30

Annotated proteins	
**Protein Accession**	**Protein Identification**
Q62720	Zinc transporter 1
D4A017	Transmembrane protein 87 A
Q10473	Polypeptide N-acetylgalactosaminyltransferase 1
D3ZAF6	ATP synthase subunit F mitochondrial
Q5BK85	Transmembrane emp24 domain-containing protein 1
Q5XIU9	Membrane-associated progesterone receptor component 2
B5DEH2	Erlin-2
F1M2P8	Protein kinase C alpha
Q62876	Synaptogyrin-1
Q0H8B9	C-type lectin domain family 2 member D11
Q5XIU4	B-cell receptor-associated protein 29
Q5U204	Ragulator complex protein LAMTOR3
Q6AYM8	Glycosylphosphatidylinositol anchor attachment 1
Q8R4A1	ERO1-like protein alpha
B0BN02	Metaxin 1
D3ZTX0	Transmembrane emp24 domain-containing protein 7
Q920L2	Succinate dehydrogenase [ubiquinone] flavoprotein subunit mitochondrial
P06686	Sodium/potassium-transporting ATPase subunit alpha-2
P06687	Sodium/potassium-transporting ATPase subunit alpha-3
F1MA50	transmembrane protein 123
P06685	Sodium/potassium-transporting ATPase subunit alpha-1
Q641Y2	NADH dehydrogenase [ubiquinone] iron-sulfur protein 2
D3ZAS9	DDRGK domain containing 1
Q641Y0	Oligosaccharyl transferase 48 kDa subunit
Q66H15	Regulator of microtubule dynamics protein 3
Q5XI52	Vezatin
Q62733	Lamina-associated polypeptide 2 isoform beta
Q9Z270	VAMP-associated protein A
Q6AYN2	Store-operated calcium entry-associated regulatory factor (SARAF)
G3V6H5	Solute carrier family 25 member 11
Q66H18	Synaptophysin-like 1
Q10468	Beta-1,4 N-acetylgalactosaminyltransferase 1
G3V6H0	Ras-related protein Rab-1b
P27139	Carbonic anhydrase 2
Q66H50	Fatty acyl-CoA reductase 1
G3V9U7	Sarcoplasmic/endoplasmic reticulum calcium ATPase 3 (SERCA3)
Q9ES40	PRA1 family protein 3
P61589	Transforming protein RhoA
P20417	Protein-tyrosine phosphatase 1B
Q9Z269	VAMP-associated protein B
P67779	Prohibitin
Q5FVF4	Transmembrane 7 superfamily member 3

Q4V888	Type 2 phosphatidylinositol 4,5-bisphosphate 4-phosphatase
P21571	ATP synthase-coupling factor 6 mitochondrial
P20411	High affinity immunoglobulin epsilon receptor subunit gamma
F1M5Z5	Anoctamin-6
Q62896	Bet1 golgi vesicular membrane trafficking protein
Q62991	Syntaxin-binding protein 1-like 2
P51796	Chloride intracellular channel protein 5
O88884	A-kinase anchor protein 1, mitochondrial
Q5XI41	Translocating chain-associated membrane protein 1
P27615	Lysosome membrane protein 2 (CD36)
A1L1L6	Mitochondrial Rho GTPase 1
P06762	Heme oxygenase 1
Q9QYV0	Disintegrin and metalloproteinase domain-containing protein 15
B5DFJ2	Protein FAM3A
Q66H21	Cholinephosphotransferase 1
G3V878	Signal peptidase complex catalytic subunit SEC11
B0K020	CDGSH iron-sulfur domain-containing protein 1
D3ZYM4	Macrophage stimulating 1 receptor
P32089	Citrate transport protein
D4A416	Cleft lip and palate transmembrane protein 1-like protein
Q4V899	Transmembrane protein 165
Q861Q1	MHC class I antigen
M0RCF3	Integrin beta 3
G3V8V4	Ceramide synthase 2
Q1WIM3	Cell adhesion molecule 3
Q3KR86	MICOS complex subunit Mic60
Q5XI31	GPI transamidase component PIG-S
P97887	Presenilin-1
Q9QYW3	MOB-like protein phocein
Q62611	Interleukin 1 receptor-like 1
O88994	Mitochondrial amidoxime reducing component 2
Q5BJZ3	Nicotinamide nucleotide transhydrogenase
P04764	Alpha-enolase
Q5XI36	CD97
G3V887	V-type proton ATPase subunit A
M0RDI5	Mitochondrial calcium uniporter
Q64578	Sarcoplasmic/endoplasmic reticulum calcium ATPase 1 (SERCA1)
Q9R1Z0	Voltage-dependent anion-selective channel protein 3
G3V9X8	Magnesium transporter protein 1
D3ZD83	Major facilitator superfamily domain-containing protein 10
P63322	Ras-related protein Ral-A
D4A533	Transmembrane anterior posterior transformation protein 1
Q6AYQ4	Transmembrane protein 109
Q64541	Sodium/potassium-transporting ATPase subunit alpha-4

O70257	Syntaxin-7
Q5FVC4	DnaJ (Hsp40) homolog subfamily B member 12
O88767	Protein/nucleic acid deglycase DJ-1
O08984	Lamin-B receptor
P51577	P2X purinoceptor 4
D3ZUX5	MICOS complex subunit MIC-19
F7FNS3	Derlin
O08775	Vascular endothelial growth factor receptor 2
Q80ZA5	Sodium-driven chloride bicarbonate exchanger
Q66H80	Coatomer subunit delta
G3V7P1	Syntaxin-12
Q920P6	Adenosine deaminase
Q91ZN1	Coronin-1A
P04785	Protein disulfide-isomerase
G3V9Y2	Phospholipid phosphatase 1
Q63016	Large neutral amino acids transporter small subunit 1
O35165	Golgi SNAP receptor complex member 2
R9PXR4	Mitochondrial import receptor subunit TOM70
Q63116	Mast/stem cell growth factor receptor
Q64244	ADP-ribosyl cyclase/cyclic ADP-ribose hydrolase 1
P81155	Voltage-dependent anion-selective channel protein 2
G3V964	Neurotrimin
B0BN30	Mitochondrial carrier homolog 1
O88777	Presenilin-2
P12368	cAMP-dependent protein kinase type II-alpha regulatory subunit
Q9WU70	Syntaxin-binding protein 5
Q5I0E7	Transmembrane emp24 domain-containing protein 9
Q9Z2Q7	Syntaxin-8
F1LM33	Leucine-rich PPR motif-containing protein mitochondrial isoform
Q64654	Lanosterol 14-alpha demethylase
P38983	40S ribosomal protein SA
P08644	GTPase KRas
Q63965	Sideroflexin-1
A1L1J9	Lipase maturation factor 2
F1M7T6	Translocon-associated protein subunit gamma
F1LQR8	Inositol 1,4,5-trisphosphate receptor type 2
P04897	Guanine nucleotide-binding protein G(i) subunit alpha-2
F1LZW6	Solute carrier family 25 member 13
Q08849	Syntaxin-3
Q8CFN2	Cell division control protein 42 homolog (CDC42)
Q8CHN6	Sphingosine-1-phosphate lyase 1
F6T9E7	P-selectin glycoprotein ligand 1
Q08851	Syntaxin-5
Q53B90	Ras-related protein Rab-43

Q63151	Long chain fatty acid CoA ligase 3
P58195	Phospholipid scramblase 1
Q9JHW5	VAMP7
Q9JI85	Nucleobindin-2
D4A2H2	Serine palmitoyltransferase 1
D3ZV75	Major facilitator superfamily domain-containing protein 1
P01830	Thy-1 membrane glycoprotein
O70377	Synaptosomal-associated protein 23 (SNAP-23)
F1LNX2	Calcium release-activated calcium channel protein 1 (Orai1)
D3ZCZ9	NADH dehydrogenase [ubiquinone] iron-sulfur protein 6
P21913	Succinate dehydrogenase [ubiquinone] iron-sulfur subunit mitochondrial
P51146	Ras-related protein Rab-4b
Q07014	Tyrosine-protein kinase Lyn
D3ZIJ5	Dolichyl-phosphate mannosyltransferase subunit 3
Q07984	Translocon-associated protein subunit delta
P31977	Ezrin
Q63135	Complement component receptor 1-like protein
P15999	ATP synthase subunit alpha mitochondrial
M0RCA3	Zinc finger protein-like 1
D3ZQG9	RT1 class I locus CE4
P07150	Annexin A1
Q6AYQ1	Golgin subfamily A member 7
Q52KJ9	Thioredoxin-related transmembrane protein 1
Q64273	Inward rectifier potassium channel 2
B2RYB8	Integrin beta 2
Q5HZE2	Transmembrane protein 120 A
Q9Z2S9	Flotillin-2
P07153	Dolichyl-diphosphooligosaccharide protein glycosyltransferase subunit 1
Q9ES21	Phosphatidylinositide phosphatase SAC1
D3ZU83	Endoplasmic reticulum-Golgi intermediate compartment 3
D3ZLT7	Uncharacterized protein KIAA2013
Q9Z2L0	Voltage-dependent anion-selective channel protein 1
P25235	Dolichyl-diphosphooligosaccharide protein glycosyltransferase subunit 2
B2RZD1	Protein transport protein Sec61 subunit beta
P11442	Clathrin heavy chain 1
Q6QBQ4	Phospholipid scramblase 3
Q5PPL3	Sterol-4-alpha-carboxylate 3-dehydrogenase decarboxylating
P12075	Cytochrome c oxidase subunit 5B mitochondrial
P11951	Cytochrome c oxidase subunit 6C2
Q05030	Platelet-derived growth factor receptor beta
P61107	Ras-related protein Rab-14
Q6TEK4	Vitamin K epoxide reductase complex subunit 1
Q05962	ADP/ATP translocase 1
Q6PCU4	Emerin

P61972	Nuclear transport factor 2
Q5PQL7	Integral membrane protein 2C
P49134	Integrin beta 1
B2RYD7	STT3B, catalytic subunit of the oligosaccharyltransferase complex
E9PU78	Isoform Bcl-X(S) of Bcl-2-like protein 1
P31044	Phosphatidylethanolamine-binding protein 1
Q5RJR8	Leucine-rich repeat-containing protein 59
F1M790	Prostaglandin F2 receptor negative regulator
O35532	Methylsterol monooxygenase 1
Q6MGB4	H2-K region expressed gene 4, rat orthologue
Q5XIL2	Phosphatidylinositol 4-kinase type 2-beta
Q63269	Inositol 1,4,5-trisphosphate receptor type 3
G3V7M2	Integrin beta 7
Q6AYF6	Glycosyltransferase 8 domain-containing protein 1
G3V6P8	Guanine nucleotide-binding protein gamma-12
B1WBW4	Armadillo repeat-containing protein 10
Q04970	GTPase NRas
B2RZB3	Eva-1 homolog B
D3ZNG3	Signal peptide peptidase-like 2A
Q9Z1J7	Amino acid transporter
Q9WUD2	Transient receptor potential cation channel subfamily V member 2 (TRPV2)
E9PTW1	Secretory carrier-associated membrane protein
F1LSN4	Fibroblast growth factor receptor
G3V9N9	alpha-1,2-Mannosidase
G3V608	Interleukin-3 receptor subunit beta
Q6MGA3	Antigen peptide transporter 2
Q794F9	4F2 cell-surface antigen heavy chain
Q64232	Very-long-chain enoyl-CoA reductase
Q6P791	Ragulator complex protein LAMTOR1
F1LNT8	VAMP8
F1M6W2	Endoplasmic reticulum metallopeptidase 1
Q5U2V8	ER membrane protein complex subunit 3
G3V7N6	Lysophospholipid acyltransferase 7
P61227	Ras-related protein Rap-2b
D4A0W1	ER membrane protein complex subunit 4
P62909	40S ribosomal protein S3
O35796	Complement component 1 Q subcomponent-binding protein mitochondrial
D3ZUD8	Transmembrane 9 superfamily member 3
Q63377	Sodium/potassium-transporting ATPase subunit beta-3
Q95II0	MHC class Ib antigen
M0RBJ0	Guanine nucleotide-binding protein gamma-2
Q63942	Ras-related protein Rab-3d
Q63584	Transmembrane emp24 domain-containing protein 10
Q5PQM0	Transmembrane protein 168

Q6EV76	Alpha-(1,6)-fucosyltransferase
F1LU48	Endoplasmic reticulum-golgi intermediate compartment 1
D3ZWZ1	Integrin alpha X
B2RYF6	Cleft lip and palate associated transmembrane protein 1
F1M779	Clathrin heavy chain
P32551	Cytochrome b-c1 complex subunit 2 mitochondrial
P0C1Q3	Lysophosphatidylcholine acyltransferase 2
B0BN52	Mitochondrial carrier homolog 2
E9PTX9	Solute carrier family 12 member 2
P61621	SEC61 alpha-1
B1WC67	Solute carrier family 25 member 24
F1LRK1	Sodium/potassium-transporting ATPase subunit alpha
F1M1U0	Matrix remodeling associated 7
Q7TSA0	Mitochondrial Rho GTPase 2
P61805	Dolichyl-diphosphooligosaccharide protein glycosyltransferase subunit DAD1
P53987	Monocarboxylate transporter 1
Q5XIN6	Mitochondrial proton/calcium exchanger protein
P20788	Cytochrome b-c1 complex subunit Rieske
G3V6N2	Transmembrane emp24 domain-containing protein 4
Q62902	Lectin mannose-binding 1
O88813	Long chain fatty acid CoA ligase 5
P28494	Alpha-mannosidase 2
O89035	Solute carrier family 25 member 10
P61131	Beta-galactoside alpha-2,3-sialyltransferase 4
P50399	Rab GDP dissociation inhibitor beta
Q9WVB1	Ras-related protein Rab-6a
Q4V8N6	Solute carrier organic anion transporter family member 2A1
Q05175	Brain acid soluble protein 1
M0R402	Protein disulfide-isomerase TMX3
Q62839	Golgin subfamily A member 2
Q7TP42	Translocation protein SEC62
Q68FV6	Glycosylated lysosomal membrane protein
Q5U316	Ras-related protein Rab-35
Q68FV1	Trimeric intracellular cation channel type B (TRIC-B)
P11167	Solute carrier family 2, facilitated glucose transporter member 1
P31399	ATP synthase subunit D mitochondrial
G3V8L7	Integrin alpha M
D4A4J3	Alpha-mannosidase 2x
O35509	Ras-related protein Rab-11b
D4A0Y9	Phosphatidylcholine translocator ABCB4
P70580	Membrane-associated progesterone receptor component 1
D4ABY2	Coatomer subunit gamma 2
Q3T1J1	Eukaryotic translation initiation factor 5A-1
Q66HA6	ADP-ribosylation factor-like protein 8B

G3V6T1	Coatomer subunit alpha
F1LP82	Ras-related protein Rab-2a
Q62636	Ras-related protein Rap-1b
Q5XIP2	GPI-anchor transamidase
Q3MIE4	Synaptic vesicle membrane protein VAT-1 homolog
Q6P742	Proteolipid protein 2
D4ACG2	Acetolactate synthase-like protein
G3V7I3	Manganese-transporting ATPase 13A1
P62815	V-type proton ATPase subunit B
Q5M9I5	Cytochrome b-c1 complex subunit 6 mitochondrial
P23347	Anion exchange protein 2
Q9Z122	Acyl-CoA 6-desaturase
Q4KLL4	Transmembrane 9 superfamily member 4
Q5XIP9	Transmembrane protein 43 (Protein LUMA)
Q9JID2	Guanine nucleotide-binding protein alpha-11
Q63198	Contactin 1
Q9Z2J5	VAMP5
A3RLA8	Low affinity immunoglobulin gamma Fc region receptor II
P10719	ATP synthase subunit beta mitochondrial
Q0R3W9	GTPase, IMAP family member 1
G3V741	Solute carrier family 25 member 3
D3ZLC4	Slc2a6 protein
Q925S8	ATP-dependent zinc metalloprotease YME1L1
O88941	Mannosyl-oligosaccharide glucosidase
O54980	Synaptogyrin-2
Q3B7U9	Peptidyl-prolyl cis-trans isomerase FKBP8
Q5XI04	Erythrocyte band 7 integral membrane protein
F1LQ92	Sel-1 homolog 1
Q4V7D1	Translocon-associated protein subunit alpha
D4AA91	Ceramide synthase 5
P22734	Catechol O-methyltransferase
F1LM93	Tyrosine-protein kinase Yes
Q2YDU8	Protein spinster homolog 1
Q63083	Nucleobindin-1
Q63085	Cyclic GMP-inhibited phosphodiesterase B
Q63081	Protein disulfide-isomerase A6
Q62940	E3 ubiquitin-protein ligase NEDD4
Q63803	Guanine nucleotide-binding protein G(s) subunit alpha isoforms XLas
Q6P6U0	Tyrosine-protein kinase Fgr
Q5EAJ6	Inhibitor kappa-B kinase-interacting protein
D4A5K6	Zinc metallopeptidase STE24
P00388	NADPH--cytochrome P450 reductase
P13838	Leukosialin
Q06647	ATP synthase subunit O mitochondrial

Q505J9	ATPase family AAA domain-containing protein 1
Q6PEC3	Protein YIF1B
G3V8I4	Syntaxin-4A
Q68FY0	Cytochrome b-c1 complex subunit 1 mitochondrial
F1LMK0	A-kinase anchoring protein 1 isoform 2
G3V679	Transferrin receptor protein 1
P29995	Inositol 1,4,5-trisphosphate receptor type 2
Q4FZT0	Stomatin-like protein 2
Q5XIE8	Integral membrane protein 2B
Q9JHE5	Sodium-coupled neutral amino acid transporter 2
Q3SWT0	Platelet endothelial cell adhesion molecule (Pecam1)
Q99N01	Organic anion-transporting polypeptide E
Q6IN10	Endothelin-converting enzyme 1 Isoform
F1M0J3	Sodium bicarbonate cotransporter 3, isophorm 4
P16391	RT1 class I histocompatibility antigen AA alpha chain
Q91Y81	Septin-2
P70550	Ras-related protein Rab-8b
Q99P74	Ras-related protein Rab-27b
Q99P75	Ras-related protein Rab-9a
Q2IBC7	Hepatocyte growth factor receptor
Q2I6B2	V-type proton ATPase subunit A1
G3V8G5	Golgi apparatus protein 1
Q2I6B1	V-type proton ATPase subunit A2
Q09326	Alpha-1,6-mannosyl-glycoprotein 2-beta-N-acetylglucosaminyltransferase
F1LMM5	Arachidonate 5-lipoxygenase
P05712	Ras-related protein Rab-2a
P62836	Ras-related protein Rap-1a
P62243	40S ribosomal protein S8
Q01827	Vesicular amine transporter 2
Q5BJK8	Golgi integral membrane protein 4
P36370	Antigen peptide transporter 1
P82995	Heat shock protein HSP 90-alpha
P42667	SEC11 homolog A
Q9JM53	Apoptosis-inducing factor 1
G3V8F5	Mitochondrial import receptor subunit TOM40 homolog
Q5XID0	Protein YIPF5
P05708	Hexokinase-1
D3ZKE6	Sarcolemmal membrane-associated protein
G3V667	Integrin alpha 6
P13386	High affinity immunoglobulin epsilon receptor subunit beta
D3ZMM8	Solute carrier family 7 member 6
B4F7C9	STT3A, catalytic subunit of the oligosaccharyltransferase complex
Q5HZY0	Erasin
Q6RUV5	Ras-related C3 botulinum toxin substrate 1 (Rac1)

Q5XID7	Armadillo repeat-containing X-linked protein 3
P82471	Guanine nucleotide-binding protein G(q) subunit alpha
P58405	Striatin-3
Q6MG61	Chloride intracellular channel protein 1
Q498C8	Protein RER1
D4A2Z6	Translocation protein SEC63 homolog
F1LMG7	Leucine-rich repeat-containing protein 7
P11240	Cytochrome c oxidase subunit 5A mitochondrial
E9PU37	Palmitoyltransferase
P10252	CD48 antigen
Q3KRE0	ATPase family AAA domain-containing protein 3
P04166	Cytochrome b5 type B
Q5U2X6	Coiled-coil domain-containing protein 47
P63095	Guanine nucleotide-binding protein G(s) subunit alpha
Q5XIK2	Thioredoxin-related transmembrane protein 2
P20291	Arachidonate 5-lipoxygenase-activating protein
D4A1D8	Surfeit 4
D4A7Y9	Essential MCU regulator, mitochondrial
B2GV71	NADH dehydrogenase [ubiquinone] 1 alpha subcomplex assembly factor 5
Q5PQX1	Torsin-1A-interacting protein 1
Q6P9V1	CD81 molecule
Q63635	Syntaxin-6
Q5RJM1	3-oxo-5-alpha-steroid 4-dehydrogenase 3
Q09073	ADP/ATP translocase 2
Q6AYA5	Transmembrane protein 106 B
P54921	Alpha-soluble NSF attachment protein (SNAP-alpha)
P09626	Potassium-transporting ATPase alpha chain 1
G3V8C4	Chloride intracellular channel protein 4
Q497A2	Solute carrier family 35 member B2
P09527	Ras-related protein Rab-7a
P29419	ATP synthase subunit E mitochondrial
P10888	Cytochrome c oxidase subunit 4 isoform 1 mitochondrial
Q66HG5	Transmembrane 9 superfamily member 2
Q5U2Z8	Elongation of very long chain fatty acids protein 1
Q63524	Transmembrane emp24 domain-containing protein 2
Q5SGE0	Leucine-rich PPR motif-containing protein mitochondrial
Q66HG9	Mitochondrial antiviral-signaling protein
P23640	Ras-related protein Rab-27a
Q4V8F3	Rhomboid-related protein 4
Q6MG55	Abhydrolase domain containing 16A
P62747	Rho-related GTP-binding protein RhoB
O70595	ATP-binding cassette sub-family B member 6
Q4AEF8	Coatomer subunit gamma 1
M0RAU0	Advanced glycosylation end product-specific receptor

P20171	GTPase HRas
B5DF51	Membrane magnesium transporter 1
P04157	Receptor-type tyrosine-protein phosphatase C (CD45)
G3V7A5	Low density lipoprotein receptor isoform
Q66HF2	Transmembrane 9 superfamily member 1
Q66HF1	NADH-ubiquinone oxidoreductase 75 kDa subunit mitochondrial
P20070	NADH-cytochrome b5 reductase 3
Q5XIH7	Prohibitin 2
Q5BQE6	Delta(24)-sterol reductase
D3ZP98	Minor histocompatibility antigen H13
P85007	Immediate early response 3-interacting protein 1
Q642A2	Type-1 angiotensin II receptor-associated protein
Q4G002	Histocompatibility 2 T region locus 24
Q6AZ61	Probable lysosomal cobalamin transporter
Q00238	Intercellular adhesion molecule 1 (ICAM1)
Q5M843	2-oxoglutarate and iron-dependent oxygenase domain-containing protein 3
D3ZMR1	Translocase of outer mitochondrial membrane 7
Q6AY20	Cation-dependent mannose-6-phosphate receptor
D3ZIP8	Endonuclease domain containing 1
B0BNG3	Vesicular integral-membrane protein VIP36
P97546	Neuroplastin
F1M391	Stimulator of interferon genes protein
P35355	Prostaglandin G/H synthase 2
Q5M7T4	Protein YIPF4
Q5I0H4	Calcium load-activated calcium channel (CLAC channel)
Q6AXT5	Ras-related protein Rab-21
D4A376	Ras-related protein Rab-12
D3ZJ32	Extended synaptotagmin 2
Q6NYB7	Ras-related protein Rab-1a
P19511	ATP synthase F(0) complex subunit B1 mitochondrial
G3V8Z1	Linker for activation of T-cells family member 2 (NTAL)
P19814	Trans-Golgi network integral membrane protein TGN38
Q76GL9	Neutral amino acid transporter A
P84903	Stromal interaction molecule 1 (STIM1)
P63245	Receptor of activated protein C kinase 1 (RACK1)
Q8SEZ5	Cytochrome c oxidase subunit 2
A1L134	Ancient ubiquitous protein 1
Q4KM74	Vesicle-trafficking protein SEC22b
P35171	Cytochrome c oxidase subunit 7A2
D3ZD11	Signal peptidase complex subunit 2
Q6AXS4	Renin receptor
D3ZM19	Protein EVI2B
P45592	Cofilin-1
Q01621	Tyrosine-protein kinase LCK

Q6AXN3	Transmembrane emp24 domain-containing protein 5
F1LZX9	Integrin alpha 5
D3ZF12	Signal peptidase complex subunit 3
Q5FVQ4	Malectin
O35142	Coatomer subunit beta 2
Q9JJ19	Na(+)/H(+) exchange regulatory cofactor NHE-RF1
Q4V8C2	Centromere/kinetochore protein zw10 homolog
Q9JJ05	Glycoprotein-N-acetylgalactosamine 3-beta-galactosyltransferase 1
P08753	Guanine nucleotide-binding protein G(k) subunit alpha
D4AD37	Inositol monophosphatase 3
D4AAL4	Adhesion G protein-coupled receptor L3
P97675	Ectonucleotide pyrophosphatase/phosphodiesterase family member 3
O35152	BET1-like protein
Q5XIA8	Growth hormone-inducible transmembrane protein
Q5M7W6	Protein FAM234A
P35289	Ras-related protein Rab-15
G3V6W2	Prolactin regulatory element-binding protein
Q4FZX7	Signal recognition particle receptor beta
P63018	Heat shock cognate 71 kDa protein
Q5XIA1	Nicalin
P63012	Ras-related protein Rab-3a
Q5RKI8	ATP-binding cassette sub-family B member 8
P35565	Calnexin
F1LPK9	Phosphatidylinositol glycan anchor biosynthesis class U protein
Q9R0C5	Polypeptide N-acetylgalactosaminyltransferase 7
D4ABI7	Very-long-chain (3R)-3-hydroxyacyl-CoA dehydratase
Q75Q41	Mitochondrial import receptor subunit TOM22 homolog
Q9Z1Z1	Eukaryotic translation initiation factor 2-alpha kinase 3
P17046	Lysosome-associated membrane glycoprotein 2 (Lamp2)
O70521	Regulator of G-protein signaling 19
Q9Z2Z8	7-dehydrocholesterol reductase
F1LNC4	Vesicle transport through interaction with t-SNAREs homolog 1B
Q9QYE7	Integrin alpha D
G3V6D3	ATP synthase subunit beta
Q9WTR7	SEC11 homolog C
Q6AXX5	Retinol dehydrogenase 11
P09456	cAMP-dependent protein kinase type I-alpha regulatory subunit
F1LX07	Solute carrier family 25 member 12
P35435	ATP synthase subunit gamma mitochondrial
D4AAE9	CDGSH iron-sulfur domain-containing protein 2
Q08163	Adenylyl cyclase-associated protein 1
P26453	Basigin
Q5RKJ9	Ras-related protein Rab-10
B0BNL4	Heme transporter HRG1

Q07647	Solute carrier family 2, facilitated glucose transporter member 3
Q8CGU6	Nicastrin
O70601	Linker for activation of T-cells family member 1 (LAT)
A0JN29	Lunapark ER junction formation factor
Q6IRF8	V-type proton ATPase subunit S1
P00507	Aspartate aminotransferase mitochondrial
M0RC99	Ras-related protein Rab-5a
D3ZF54	Anoctamin-10
P35952	Low-density lipoprotein receptor
Q9Z1X1	Extended synaptotagmin 1
P84817	Mitochondrial fission 1 protein
Q9JK11	Reticulon-4
P14562	Lysosome-associated membrane glycoprotein 1 (Lamp1)
Q91ZQ0	Vacuole membrane protein 1
Q6AXV4	Sorting and assembly machinery component 50 homolog
Q6P7D4	Cytochrome P450 20A1
D3ZP13	Sulfhydryl oxidase 2
Q9Z1E1	Flotillin-1
Q6IMF3	Keratin, type II cytoskeletal 1
P63025	VAMP3
D3ZFK5	Signal peptidase complex subunit 1
Q5FVN0	Lysophospholipid acyltransferase 5
P19234	NADH dehydrogenase [ubiquinone] flavoprotein 2 mitochondrial
Q499P3	C1GALT1-specific chaperone 1
P19627	Guanine nucleotide-binding protein G(z) subunit alpha
P23514	Coatomer subunit beta 1
Q99JC6	Tapasin
P35280	Ras-related protein Rab-8a

ID Term	GO Term	Fold Enriched	Count	*p* -value	FDR
UP_KEYWORDS	TRANSMEMBRANE HELIX	1.28	357	3.48E-8	4.73E-5
Annotated proteins					
Protein Accession			Protein Identification		
Q62720			Zinc transporter 1		
D4A017			Transmembrane protein 87 A		
Q10473			Polypeptide N-acetylgalactosaminyltransferase 1		
D3ZAF6			ATP synthase subunit F mitochondrial		
Q5BK85			Transmembrane emp24 domain-containing protein 1		
Q5XIU9			Membrane-associated progesterone receptor component 2		
B5DEH2			Erlin-2		
Q62876			Synaptogyrin-1		
Q6PEC3			Protein YIF1B		

Q5XIU4	B-cell receptor-associated protein 29
Q0H8B9	C-type lectin domain family 2 member D11
G3V8I4	Syntaxin-4A
F1LMK0	A-kinase anchoring protein 1 isoform 2
Q6AYM8	Glycosylphosphatidylinositol anchor attachment 1
B0BN02	Metaxin 1
D3ZTX0	Transmembrane emp24 domain-containing protein 7
G3V679	Transferrin receptor protein 1
P06686	Sodium/potassium-transporting ATPase subunit alpha-2
F1MA50	transmembrane protein 123
P06687	Sodium/potassium-transporting ATPase subunit alpha-3
P29995	Inositol 1,4,5-trisphosphate receptor type 2
P06685	Sodium/potassium-transporting ATPase subunit alpha-1
D3ZAS9	DDRGK domain containing 1
Q641Y0	Oligosaccharyl transferase 48 kDa subunit
Q5XIE8	Integral membrane protein 2B
Q66H15	Regulator of microtubule dynamics protein 3
Q5XI52	Vezatin
Q62733	Lamina-associated polypeptide 2 isoform beta
Q9JHE5	Sodium-coupled neutral amino acid transporter 2
Q9Z270	VAMP-associated protein A
Q6AYN2	Store-operated calcium entry-associated regulatory factor (SARAF)
G3V6H5	Solute carrier family 25 member 11
Q66H18	Synaptophysin-like 1
Q10468	Beta-1,4 N-acetylgalactosaminyltransferase 1
Q3SWT0	Platelet endothelial cell adhesion molecule (Pecam1)
Q99N01	Organic anion-transporting polypeptide E
Q66H50	Fatty acyl-CoA reductase 1
Q6IN10	Endothelin-converting enzyme 1 Isoform
G3V9U7	Sarcoplasmic/endoplasmic reticulum calcium ATPase 3 (SERCA3)
F1M0J3	Sodium bicarbonate cotransporter 3, isophorm 4
Q9ES40	PRA1 family protein 3
Q9Z269	VAMP-associated protein B
Q5FVF4	Transmembrane 7 superfamily member 3
Q4V888	Type 2 phosphatidylinositol 4,5-bisphosphate 4-phosphatase
P20411	High affinity immunoglobulin epsilon receptor subunit gamma
P16391	RT1 class I histocompatibility antigen AA alpha chain
F1M5Z5	Anoctamin-6
Q62896	Bet1 golgi vesicular membrane trafficking protein
P51796	Chloride intracellular channel protein 5
O88884	A-kinase anchor protein 1, mitochondrial
Q2IBC7	Hepatocyte growth factor receptor
Q5XI41	Translocating chain-associated membrane protein 1
Q2I6B2	V-type proton ATPase subunit A1

P27615	Lysosome membrane protein 2 (CD36)
G3V8G5	Golgi apparatus protein 1
A1L1L6	Mitochondrial Rho GTPase 1
Q09326	Alpha-1,6-mannosyl-glycoprotein 2-beta-N-acetylglucosaminyltransferase
Q2I6B1	V-type proton ATPase subunit A2
Q9QYV0	Disintegrin and metalloproteinase domain-containing protein 15
B5DFJ2	Protein FAM3A
Q66H21	Cholinephosphotransferase 1
G3V878	Signal peptidase complex catalytic subunit SEC11
B0K020	CDGSH iron-sulfur domain-containing protein 1
D3ZYM4	Macrophage stimulating 1 receptor
P32089	Citrate transport protein
D4A416	Cleft lip and palate transmembrane protein 1-like protein
Q4V899	Transmembrane protein 165
Q861Q1	MHC class I antigen
M0RCF3	Integrin beta 3
G3V8V4	Ceramide synthase 2
Q1WIM3	Cell adhesion molecule 3
Q01827	Vesicular amine transporter 2
Q3KR86	MICOS complex subunit Mic60
P97887	Presenilin-1
Q5XI31	GPI transamidase component PIG-S
Q5BJK8	Golgi integral membrane protein 4
P36370	Antigen peptide transporter 1
Q62611	Interleukin 1 receptor-like 1
P42667	SEC11 homolog A
Q9JM53	Apoptosis-inducing factor 1
O88994	Mitochondrial amidoxime reducing component 2
Q5BJZ3	Nicotinamide nucleotide transhydrogenase
Q5XI36	CD97
G3V887	V-type proton ATPase subunit A
Q5XID0	Protein YIPF5
D3ZKE6	Sarcolemmal membrane-associated protein
G3V667	Integrin alpha 6
P13386	High affinity immunoglobulin epsilon receptor subunit beta
M0RDI5	Mitochondrial calcium uniporter
D3ZMM8	Solute carrier family 7 member 6
B4F7C9	STT3A, catalytic subunit of the oligosaccharyltransferase complex
Q5XID7	Armadillo repeat-containing X-linked protein 3
Q64578	Sarcoplasmic/endoplasmic reticulum calcium ATPase 1 (SERCA1)
Q6MG61	Chloride intracellular channel protein 1
G3V9X8	Magnesium transporter protein 1
Q498C8	Protein RER1
D3ZD83	Major facilitator superfamily domain-containing protein 10

D4A2Z6	Translocation protein SEC63 homolog
D4A533	Transmembrane anterior posterior transformation protein 1
Q6AYQ4	Transmembrane protein 109
Q64541	Sodium/potassium-transporting ATPase subunit alpha-4
E9PU37	Palmitoyltransferase
O08984	Lamin-B receptor
Q5FVC4	DnaJ (Hsp40) homolog subfamily B member 12
O70257	Syntaxin-7
P51577	P2X purinoceptor 4
Q3KRE0	ATPase family AAA domain-containing protein 3
P04166	Cytochrome b5 type B
F7FNS3	Derlin
O08775	Vascular endothelial growth factor receptor 2
Q80ZA5	Sodium-driven chloride bicarbonate exchanger
G3V7P1	Syntaxin-12
Q5U2X6	Coiled-coil domain-containing protein 47
G3V9Y2	Phospholipid phosphatase 1
Q5XIK2	Thioredoxin-related transmembrane protein 2
P20291	Arachidonate 5-lipoxygenase-activating protein
Q63016	Large neutral amino acids transporter small subunit 1
O35165	Golgi SNAP receptor complex member 2
R9PXR4	Mitochondrial import receptor subunit TOM70
D4A1D8	Surfeit 4
Q63116	Mast/stem cell growth factor receptor
D4A7Y9	Essential MCU regulator, mitochondrial
Q5PQX1	Torsin-1A-interacting protein 1
Q64244	ADP-ribosyl cyclase/cyclic ADP-ribose hydrolase 1
Q6P9V1	CD81 molecule
B0BN30	Mitochondrial carrier homolog 1
Q63635	Syntaxin-6
O88777	Presenilin-2
Q5RJM1	3-oxo-5-alpha-steroid 4-dehydrogenase 3
Q09073	ADP/ATP translocase 2
Q5I0E7	Transmembrane emp24 domain-containing protein 9
Q9Z2Q7	Syntaxin-8
Q6AYA5	Transmembrane protein 106 B
Q64654	Lanosterol 14-alpha demethylase
Q63965	Sideroflexin-1
A1L1J9	Lipase maturation factor 2
F1M7T6	Translocon-associated protein subunit gamma
P09626	Potassium-transporting ATPase alpha chain 1
G3V8C4	Chloride intracellular channel protein 4
F1LQR8	Inositol 1,4,5-trisphosphate receptor type 2
Q497A2	Solute carrier family 35 member B2

F1LZW6	Solute carrier family 25 member 13
Q08849	Syntaxin-3
Q8CHN6	Sphingosine-1-phosphate lyase 1
Q66HG5	Transmembrane 9 superfamily member 2
F6T9E7	P-selectin glycoprotein ligand 1
Q08851	Syntaxin-5
Q63151	Long chain fatty acid CoA ligase 3
Q5U2Z8	Elongation of very long chain fatty acids protein 1
P58195	Phospholipid scramblase 1
Q9JHW5	VAMP7
Q63524	Transmembrane emp24 domain-containing protein 2
D4A2H2	Serine palmitoyltransferase 1
D3ZV75	Major facilitator superfamily domain-containing protein 1
Q66HG9	Mitochondrial antiviral-signaling protein
F1LNX2	Calcium release-activated calcium channel protein 1 (Orai1)
Q4V8F3	Rhomboid-related protein 4
Q6MG55	Abhydrolase domain containing 16A
D3ZIJ5	Dolichyl-phosphate mannosyltransferase subunit 3
O70595	ATP-binding cassette sub-family B member 6
Q07984	Translocon-associated protein subunit delta
M0RAU0	Advanced glycosylation end product-specific receptor
B5DF51	Membrane magnesium transporter 1
Q63135	Complement component receptor 1-like protein
M0RCA3	Zinc finger protein-like 1
P04157	Receptor-type tyrosine-protein phosphatase C (CD45)
D3ZQG9	RT1 class I locus CE4
G3V7A5	Low density lipoprotein receptor isoform
Q52KJ9	Thioredoxin-related transmembrane protein 1
Q64273	Inward rectifier potassium channel 2
Q66HF2	Transmembrane 9 superfamily member 1
B2RYB8	Integrin beta 2
Q5HZE2	Transmembrane protein 120 A
Q5BQE6	Delta(24)-sterol reductase
D3ZP98	Minor histocompatibility antigen H13
D3ZU83	Endoplasmic reticulum-Golgi intermediate compartment 3
Q9ES21	Phosphatidylinositide phosphatase SAC1
P07153	Dolichyl-diphosphooligosaccharide protein glycosyltransferase subunit 1
D3ZLT7	Uncharacterized protein KIAA2013
P25235	Dolichyl-diphosphooligosaccharide protein glycosyltransferase subunit 2
P85007	Immediate early response 3-interacting protein 1
Q4G002	Histocompatibility 2 T region locus 24
Q642A2	Type-1 angiotensin II receptor-associated protein
Q6AZ61	Probable lysosomal cobalamin transporter
B2RZD1	Protein transport protein Sec61 subunit beta

Q5M843	2-oxoglutarate and iron-dependent oxygenase domain-containing protein 3
Q00238	Intercellular adhesion molecule 1 (ICAM1)
Q6QBQ4	Phospholipid scramblase 3
D3ZMR1	Translocase of outer mitochondrial membrane 7
Q5PPL3	Sterol-4-alpha-carboxylate 3-dehydrogenase decarboxylating
Q6AY20	Cation-dependent mannose-6-phosphate receptor
P11951	Cytochrome c oxidase subunit 6C2
Q05030	Platelet-derived growth factor receptor beta
Q6TEK4	Vitamin K epoxide reductase complex subunit 1
D3ZIP8	Endonuclease domain containing 1
Q05962	ADP/ATP translocase 1
Q6PCU4	Emerin
Q5PQL7	Integral membrane protein 2C
B0BNG3	Vesicular integral-membrane protein VIP36
P49134	Integrin beta 1
P97546	Neuroplastin
B2RYD7	STT3B, catalytic subunit of the oligosaccharyltransferase complex
F1M391	Stimulator of interferon genes protein
E9PU78	Isoform Bcl-X(S) of Bcl-2-like protein 1
Q5M7T4	Protein YIPF4
Q5I0H4	Calcium load-activated calcium channel (CLAC channel)
Q5RJR8	Leucine-rich repeat-containing protein 59
D3ZJ32	Extended synaptotagmin 2
G3V8Z1	Linker for activation of T-cells family member 2 (NTAL)
P19814	Trans-Golgi network integral membrane protein TGN38
F1M790	Prostaglandin F2 receptor negative regulator
Q76GL9	Neutral amino acid transporter A
O35532	Methylsterol monooxygenase 1
Q6MGB4	H2-K region expressed gene 4, rat orthologue
Q63269	Inositol 1,4,5-trisphosphate receptor type 3
G3V7M2	Integrin beta 7
Q6AYF6	Glycosyltransferase 8 domain-containing protein 1
P84903	Stromal interaction molecule 1 (STIM1)
Q8SEZ5	Cytochrome c oxidase subunit 2
A1L134	Ancient ubiquitous protein 1
Q4KM74	Vesicle-trafficking protein SEC22b
B1WBW4	Armadillo repeat-containing protein 10
P35171	Cytochrome c oxidase subunit 7A2
B2RZB3	Eva-1 homolog B
D3ZNG3	Signal peptide peptidase-like 2A
Q9WUD2	Transient receptor potential cation channel subfamily V member 2 (TRPV2)
Q9Z1J7	Amino acid transporter
D3ZD11	Signal peptidase complex subunit 2
E9PTW1	Secretory carrier-associated membrane protein

F1LSN4	Fibroblast growth factor receptor
Q6AXS4	Renin receptor
D3ZM19	Protein EVI2B
G3V9N9	alpha-1,2-Mannosidase
Q6MGA3	Antigen peptide transporter 2
G3V608	Interleukin-3 receptor subunit beta
Q794F9	4F2 cell-surface antigen heavy chain
Q6AXN3	Transmembrane emp24 domain-containing protein 5
F1LNT8	VAMP8
Q64232	Very-long-chain enoyl-CoA reductase
F1M6W2	Endoplasmic reticulum metallopeptidase 1
F1LZX9	Integrin alpha 5
D3ZF12	Signal peptidase complex subunit 3
Q5U2V8	ER membrane protein complex subunit 3
G3V7N6	Lysophospholipid acyltransferase 7
Q5FVQ4	Malectin
D4A0W1	ER membrane protein complex subunit 4
D3ZUD8	Transmembrane 9 superfamily member 3
Q63377	Sodium/potassium-transporting ATPase subunit beta-3
Q95II0	MHC class Ib antigen
Q9JJ05	Glycoprotein-N-acetylgalactosamine 3-beta-galactosyltransferase 1
Q63584	Transmembrane emp24 domain-containing protein 10
Q5PQM0	Transmembrane protein 168
Q6EV76	Alpha-(1,6)-fucosyltransferase
F1LU48	Endoplasmic reticulum-golgi intermediate compartment 1
D3ZWZ1	Integrin alpha X
B2RYF6	Cleft lip and palate associated transmembrane protein 1
P0C1Q3	Lysophosphatidylcholine acyltransferase 2
B0BN52	Mitochondrial carrier homolog 2
E9PTX9	Solute carrier family 12 member 2
P61621	SEC61 alpha-1
B1WC67	Solute carrier family 25 member 24
F1LRK1	Sodium/potassium-transporting ATPase subunit alpha
F1M1U0	Matrix remodeling associated 7
D4AD37	Inositol monophosphatase 3
Q7TSA0	Mitochondrial Rho GTPase 2
P61805	Dolichyl-diphosphooligosaccharide protein glycosyltransferase subunit DAD1
D4AAL4	Adhesion G protein-coupled receptor L3
P53987	Monocarboxylate transporter 1
Q5XIN6	Mitochondrial proton/calcium exchanger protein
P20788	Cytochrome b-c1 complex subunit Rieske
G3V6N2	Transmembrane emp24 domain-containing protein 4
P28494	Alpha-mannosidase 2
O88813	Long chain fatty acid CoA ligase 5

P97675	Ectonucleotide pyrophosphatase/phosphodiesterase family member 3
Q62902	Lectin mannose-binding 1
P61131	Beta-galactoside alpha-2,3-sialyltransferase 4
O89035	Solute carrier family 25 member 10
O35152	BET1-like protein
Q5XIA8	Growth hormone-inducible transmembrane protein
Q4V8N6	Solute carrier organic anion transporter family member 2A1
Q5M7W6	Protein FAM234A
G3V6W2	Prolactin regulatory element-binding protein
Q4FZX7	Signal recognition particle receptor beta
M0R402	Protein disulfide-isomerase TMX3
Q5XIA1	Nicalin
Q5RKI8	ATP-binding cassette sub-family B member 8
P35565	Calnexin
Q7TP42	Translocation protein SEC62
F1LPK9	Phosphatidylinositol glycan anchor biosynthesis class U protein
Q68FV6	Glycosylated lysosomal membrane protein
Q9R0C5	Polypeptide N-acetylgalactosaminyltransferase 7
D4ABI7	Very-long-chain (3R)-3-hydroxyacyl-CoA dehydratase
Q68FV1	Trimeric intracellular cation channel type B (TRIC-B)
Q75Q41	Mitochondrial import receptor subunit TOM22 homolog
Q9Z1Z1	Eukaryotic translation initiation factor 2-alpha kinase 3
P11167	Solute carrier family 2, facilitated glucose transporter member 1
G3V8L7	Integrin alpha M
D4A4J3	Alpha-mannosidase 2x
P17046	Lysosome-associated membrane glycoprotein 2 (Lamp2)
D4A0Y9	Phosphatidylcholine translocator ABCB4
F1LNC4	Vesicle transport through interaction with t-SNAREs homolog 1B
Q9Z2Z8	7-dehydrocholesterol reductase
Q9QYE7	Integrin alpha D
P70580	Membrane-associated progesterone receptor component 1
Q9WTR7	SEC11 homolog C
Q6AXX5	Retinol dehydrogenase 11
F1LX07	Solute carrier family 25 member 12
D4AAE9	CDGSH iron-sulfur domain-containing protein 2
P26453	Basigin
B0BNL4	Heme transporter HRG1
Q6P742	Proteolipid protein 2
Q5XIP2	GPI-anchor transamidase
D4ACG2	Acetolactate synthase-like protein
G3V7I3	Manganese-transporting ATPase 13A1
P23347	Anion exchange protein 2
Q4KLL4	Transmembrane 9 superfamily member 4
Q9Z122	Acyl-CoA 6-desaturase

Q5XIP9	Transmembrane protein 43 (Protein LUMA)
Q07647	Solute carrier family 2, facilitated glucose transporter member 3
Q9Z2J5	VAMP5
Q8CGU6	Nicastrin
O70601	Linker for activation of T-cells family member 1 (LAT)
A3RLA8	Low affinity immunoglobulin gamma Fc region receptor II
A0JN29	Lunapark ER junction formation factor
Q0R3W9	GTPase, IMAP family member 1
G3V741	Solute carrier family 25 member 3
Q6IRF8	V-type proton ATPase subunit S1
D3ZLC4	Slc2a6 protein
Q925S8	ATP-dependent zinc metalloprotease YME1L1
O88941	Mannosyl-oligosaccharide glucosidase
O54980	Synaptogyrin-2
Q3B7U9	Peptidyl-prolyl cis-trans isomerase FKBP8
Q5XI04	Erythrocyte band 7 integral membrane protein
D3ZF54	Anoctamin-10
P35952	Low-density lipoprotein receptor
Q9Z1X1	Extended synaptotagmin 1
F1LQ92	Sel-1 homolog 1
Q9JK11	Reticulon-4
P84817	Mitochondrial fission 1 protein
Q4V7D1	Translocon-associated protein subunit alpha
P14562	Lysosome-associated membrane glycoprotein 1 (Lamp1)
D4AA91	Ceramide synthase 5
P22734	Catechol O-methyltransferase
Q91ZQ0	Vacuole membrane protein 1
Q2YDU8	Protein spinster homolog 1
Q63085	Cyclic GMP-inhibited phosphodiesterase B
Q6P7D4	Cytochrome P450 20A1
D3ZP13	Sulfhydryl oxidase 2
P63025	VAMP3
D3ZFK5	Signal peptidase complex subunit 1
Q5FVN0	Lysophospholipid acyltransferase 5
Q499P3	C1GALT1-specific chaperone 1
Q5EAJ6	Inhibitor kappa-B kinase-interacting protein
D4A5K6	Zinc metallopeptidase STE24
Q99JC6	Tapasin
P13838	Leukosialin
P00388	NADPH--cytochrome P450 reductase

Annotation Cluster Enrichment Score: 4.17

ID Term	GO Term	Fold Enriched	Count	*p* -value	FDR
GO:0043303	MAST CELL DEGRANULATION	6.01	13	8.69E-7	1.70E-4

Annotated proteins

Protein Accession	Protein Identification
G3V8Z1	Linker for activation of T-cells family member 2 (NTAL)
P63102	14-3-3 protein zeta/delta
P13386	High affinity immunoglobulin epsilon receptor subunit beta
Q07014	Tyrosine-protein kinase Lyn
F1LNT8	VAMP8
O70601	Linker for activation of T-cells family member 1 (LAT)
Q63116	Mast/stem cell growth factor receptor
P06762	Heme oxygenase 1
Q6P6U0	Tyrosine-protein kinase Fgr
Q9JHW5	VAMP7
P63025	VAMP3
P20411	High affinity immunoglobulin epsilon receptor subunit gamma
O70377	Synaptosomal-associated protein 23 (SNAP-23)

ID Term	GO Term	Fold Enriched	Count	*p* -value	FDR
GO:0043299	LEUKOCYTE DEGRANULATION	5.47	20	5.33E-9	1.04E-5

Annotated proteins

Protein Accession	Protein Identification
Q91ZN1	Coronin-1A
Q07014	Tyrosine-protein kinase Lyn
O70601	Linker for activation of T-cells family member 1 (LAT)
Q5U1Y2	Ras-related C3 botulinum toxin substrate 2 (Rac2)
Q63116	Mast/stem cell growth factor receptor
G3V8I4	Syntaxin-4A
P06762	Heme oxygenase 1
P63025	VAMP3
G3V8Z1	Linker for activation of T-cells family member 2 (NTAL)
P63102	14-3-3 protein zeta/delta
P13386	High affinity immunoglobulin epsilon receptor subunit beta
F1LNT8	VAMP8
Q6P6U0	Tyrosine-protein kinase Fgr
Q9JHW5	VAMP7
P14562	Lysosome-associated membrane glycoprotein 1 (Lamp1)
Q62753	Syntaxin-binding protein 2
P20411	High affinity immunoglobulin epsilon receptor subunit gamma
O70377	Synaptosomal-associated protein 23 (SNAP-23)

G3V637	Syntaxin-binding protein 2, isoform
P23640	Ras-related protein Rab-27a

ID Term	GO Term	Fold Enriched	Count	*p* -value	FDR
GO:0002279	MAST CELL ACTIVATION INVOLVED IN IMMUNE RESPONSE	5.96	16	1.02E-7	1.99E-4

Annotated proteins

Protein Accession	Protein Identification
Q07014	Tyrosine-protein kinase Lyn
O70601	Linker for activation of T-cells family member 1 (LAT)
Q5U1Y2	Ras-related C3 botulinum toxin substrate 2 (Rac2)
Q63116	Mast/stem cell growth factor receptor
P06762	Heme oxygenase 1
P63025	VAMP3
G3V8Z1	Linker for activation of T-cells family member 2 (NTAL)
P63102	14-3-3 protein zeta/delta
P13386	High affinity immunoglobulin epsilon receptor subunit beta
F1LNT8	VAMP8
Q6P6U0	Tyrosine-protein kinase Fgr
Q9JHW5	VAMP7
Q62753	Syntaxin-binding protein 2
O70377	Synaptosomal-associated protein 23 (SNAP-23)
P20411	High affinity immunoglobulin epsilon receptor subunit gamma
G3V637	Syntaxin-binding protein 2, isoform

Annotation Cluster Enrichment Score: 3.10

ID Term	GO Term	Fold Enriched	Count	*p* -value	FDR
GO:0023056	POSITIVE REGULATION OF SIGNALING	1.43	112	9.53E-5	1.8E-2

Annotated proteins

Protein Accession	Protein Identification
D4A465	Ragulator complex protein LAMTOR2
F1M2P8	Protein kinase C alpha
G3V8I4	Syntaxin-4A
Q00238	Intercellular adhesion molecule 1 (ICAM1)
Q5U204	Ragulator complex protein LAMTOR3
P0DP29	Calmodulin-1

Q05030	Platelet-derived growth factor receptor beta
Q5PQL7	Integral membrane protein 2C
D3ZAS9	DDRGK domain containing 1
P49134	Integrin beta 1
P97546	Neuroplastin
Q9Z270	VAMP-associated protein A
P07632	Superoxide dismutase [Cu-Zn]
Q3SWT0	Platelet endothelial cell adhesion molecule (Pecam1)
P35355	Prostaglandin G/H synthase 2
P31044	Phosphatidylethanolamine-binding protein 1
P27139	Carbonic anhydrase 2
Q9JLH7	CDK5 regulatory subunit-associated protein 3
Q6IN10	Endothelin-converting enzyme 1 Isoform
D3ZWS0	Protein scribble homolog
Q9ES40	PRA1 family protein 3
P61589	Transforming protein RhoA
P20417	Protein-tyrosine phosphatase 1B
F1LQ81	Vesicle-fusing ATPase
Q63269	Inositol 1,4,5-trisphosphate receptor type 3
P63245	Receptor of activated protein C kinase 1 (RACK1)
P67779	Prohibitin
P70550	Ras-related protein Rab-8b
Q2IBC7	Hepatocyte growth factor receptor
P35704	Peroxiredoxin-2
P11762	Galectin-1
F1LSN4	Fibroblast growth factor receptor
P06762	Heme oxygenase 1
Q6AXS4	Renin receptor
Q01621	Tyrosine-protein kinase LCK
F1LNT8	VAMP8
Q6P791	Ragulator complex protein LAMTOR1
D3ZYM4	Macrophage stimulating 1 receptor
F1LZX9	Integrin alpha 5
P07943	Aldose reductase
P62836	Ras-related protein Rap-1a
P62909	40S ribosomal protein S3
O35796	Complement component 1 Q subcomponent-binding protein mitochondrial
M0RCF3	Integrin beta 3
D3ZF11	Ragulator complex protein LAMTOR5
Q9JJ19	Na(+)/H(+) exchange regulatory cofactor NHE-RF1
Q5XI34	Serine/threonine-protein phosphatase 2A 65 kDa regulatory subunit A alpha isoform
P10960	Prosaposin
P97887	Presenilin-1
P52555	Endoplasmic reticulum resident protein 29

B0BN52	Mitochondrial carrier homolog 2
M0RDI5	Mitochondrial calcium uniporter
P06765	Platelet factor 4
G3V6N2	Transmembrane emp24 domain-containing protein 4
Q6TXF3	Acyl-CoA-binding protein
P62138	Serine/threonine-protein phosphatase PP1-alpha catalytic subunit
B2RYQ5	Enhancer of rudimentary homolog
E9PU37	Palmitoyltransferase
O88767	Protein/nucleic acid deglycase DJ-1
Q9Z1Z1	Eukaryotic translation initiation factor 2-alpha kinase 3
P51577	P2X purinoceptor 4
P35213	14-3-3 protein beta/alpha
Q642E3	CDK5 regulatory subunit-associated protein 3, isoform
O08775	Vascular endothelial growth factor receptor 2
Q6AYI1	Probable ATP-dependent RNA helicase DDX5
P47819	Glial fibrillary acidic protein
Q9EQX9	Ubiquitin-conjugating enzyme E2 N
Q920P6	Adenosine deaminase
P10860	Glutamate dehydrogenase 1
P63095	Guanine nucleotide-binding protein G(s) subunit alpha
G3V7Q7	Ras GTPase-activating-like protein IQGAP1
Q63116	Mast/stem cell growth factor receptor
P11598	Protein disulfide-isomerase A3
Q64244	ADP-ribosyl cyclase/cyclic ADP-ribose hydrolase 1
Q62636	Ras-related protein Rap-1b
Q6P9V1	CD81 molecule
P18418	Calreticulin
O88777	Presenilin-2
P08644	GTPase KRas
O70601	Linker for activation of T-cells family member 1 (LAT)
P21708	Mitogen-activated protein kinase 3
P04897	Guanine nucleotide-binding protein G(i) subunit alpha-2
Q497A2	Solute carrier family 35 member B2
Q6IRF8	V-type proton ATPase subunit S1
Q08849	Syntaxin-3
E9PT29	ATP-dependent RNA helicase DDX17
Q8CFN2	Cell division control protein 42 homolog (CDC42)
Q9EPV5	Apoptotic protease-activating factor 1
Q3B7U9	Peptidyl-prolyl cis-trans isomerase FKBP8
Q68FX4	Hematopoietic cell-specific LYN substrate 1 (hcls1)
P58195	Phospholipid scramblase 1
Q9JHW5	VAMP7
P84817	Mitochondrial fission 1 protein
F1LQJ7	Phosphoenolpyruvate carboxykinase [GTP], mitochondrial

Q66HG9	Mitochondrial antiviral-signaling protein
Q68FP1	Gelsolin
Q63486	Ras-related GTP-binding protein A (Rag A)
Q07014	Tyrosine-protein kinase Lyn
M0RAU0	Advanced glycosylation end product-specific receptor
P20171	GTPase HRas
Q9Z1E1	Flotillin-1
P63025	VAMP3
P04157	Receptor-type tyrosine-protein phosphatase C (CD45)
Q4KM97	Tyrosine-protein kinase BLK
Q62940	E3 ubiquitin-protein ligase NEDD4
Q62658	Peptidyl-prolyl cis-trans isomerase FKBP1A
B0BNJ1	Sorcin
Q6P6U0	Tyrosine-protein kinase Fgr
Q63803	Guanine nucleotide-binding protein G(s) subunit alpha isoforms XLas
G3V8A5	Vacuolar protein sorting-associated protein 35
Q5XIH7	Prohibitin 2
P00388	NADPH--cytochrome P450 reductase

ID Term	GO Term	Fold Enriched	Count	*p* -value	FDR
**GO:0010647**	**POSITIVE REGULATION OF CELL COMMUNICATION**	**1.40**	**109**	**2.93E-4**	**5.7E-2**
**Annotated proteins**					
**Protein Accession**	**Protein Identification**				
D4A465	Ragulator complex protein LAMTOR2				
F1M2P8	Protein kinase C alpha				
G3V8I4	Syntaxin-4A				
Q00238	Intercellular adhesion molecule 1 (ICAM1)				
Q5U204	Ragulator complex protein LAMTOR3				
P0DP29	Calmodulin-1				
Q05030	Platelet-derived growth factor receptor beta				
Q5PQL7	Integral membrane protein 2C				
D3ZAS9	DDRGK domain containing 1				
P49134	Integrin beta 1				
P97546	Neuroplastin				
Q9Z270	VAMP-associated protein A				
P07632	Superoxide dismutase [Cu-Zn]				
P35355	Prostaglandin G/H synthase 2				
Q3SWT0	Platelet endothelial cell adhesion molecule (Pecam1)				
P31044	Phosphatidylethanolamine-binding protein 1				
P27139	Carbonic anhydrase 2				

Q9JLH7	CDK5 regulatory subunit-associated protein 3
Q6IN10	Endothelin-converting enzyme 1 Isoform
P61589	Transforming protein RhoA
P20417	Protein-tyrosine phosphatase 1B
Q9ES40	PRA1 family protein 3
Q63269	Inositol 1,4,5-trisphosphate receptor type 3
P63245	Receptor of activated protein C kinase 1 (RACK1)
P67779	Prohibitin
P70550	Ras-related protein Rab-8b
Q2IBC7	Hepatocyte growth factor receptor
P35704	Peroxiredoxin-2
P11762	Galectin-1
F1LSN4	Fibroblast growth factor receptor
P06762	Heme oxygenase 1
Q6AXS4	Renin receptor
Q01621	Tyrosine-protein kinase LCK
F1LNT8	VAMP8
Q6P791	Ragulator complex protein LAMTOR1
D3ZYM4	Macrophage stimulating 1 receptor
F1LZX9	Integrin alpha 5
P07943	Aldose reductase
P62836	Ras-related protein Rap-1a
P62909	40S ribosomal protein S3
O35796	Complement component 1 Q subcomponent-binding protein mitochondrial
M0RCF3	Integrin beta 3
D3ZF11	Ragulator complex protein LAMTOR5
Q9JJ19	Na(+)/H(+) exchange regulatory cofactor NHE-RF1
Q5XI34	Serine/threonine-protein phosphatase 2A 65 kDa regulatory subunit A alpha isoform
P10960	Prosaposin
P97887	Presenilin-1
P52555	Endoplasmic reticulum resident protein 29
B0BN52	Mitochondrial carrier homolog 2
M0RDI5	Mitochondrial calcium uniporter
P06765	Platelet factor 4
G3V6N2	Transmembrane emp24 domain-containing protein 4
Q6TXF3	Acyl-CoA-binding protein
P62138	Serine/threonine-protein phosphatase PP1-alpha catalytic subunit
B2RYQ5	Enhancer of rudimentary homolog
E9PU37	Palmitoyltransferase
O88767	Protein/nucleic acid deglycase DJ-1
Q9Z1Z1	Eukaryotic translation initiation factor 2-alpha kinase 3
P51577	P2X purinoceptor 4
P35213	14-3-3 protein beta/alpha
Q642E3	CDK5 regulatory subunit-associated protein 3, isoform

O08775	Vascular endothelial growth factor receptor 2
Q6AYI1	Probable ATP-dependent RNA helicase DDX5
P47819	Glial fibrillary acidic protein
Q9EQX9	Ubiquitin-conjugating enzyme E2 N
Q920P6	Adenosine deaminase
P10860	Glutamate dehydrogenase 1
P63095	Guanine nucleotide-binding protein G(s) subunit alpha
G3V7Q7	Ras GTPase-activating-like protein IQGAP1
Q63116	Mast/stem cell growth factor receptor
P11598	Protein disulfide-isomerase A3
Q64244	ADP-ribosyl cyclase/cyclic ADP-ribose hydrolase 1
Q62636	Ras-related protein Rap-1b
Q6P9V1	CD81 molecule
P18418	Calreticulin
O88777	Presenilin-2
P08644	GTPase KRas
O70601	Linker for activation of T-cells family member 1 (LAT)
P21708	Mitogen-activated protein kinase 3
P04897	Guanine nucleotide-binding protein G(i) subunit alpha-2
Q497A2	Solute carrier family 35 member B2
Q6IRF8	V-type proton ATPase subunit S1
Q08849	Syntaxin-3
E9PT29	ATP-dependent RNA helicase DDX17
Q8CFN2	Cell division control protein 42 homolog (CDC42)
Q9EPV5	Apoptotic protease-activating factor 1
Q3B7U9	Peptidyl-prolyl cis-trans isomerase FKBP8
Q68FX4	Hematopoietic cell-specific LYN substrate 1 (hcls1)
P58195	Phospholipid scramblase 1
Q9JHW5	VAMP7
P84817	Mitochondrial fission 1 protein
F1LQJ7	Phosphoenolpyruvate carboxykinase [GTP], mitochondrial
Q66HG9	Mitochondrial antiviral-signaling protein
Q68FP1	Gelsolin
Q63486	Ras-related GTP-binding protein A (Rag A)
Q07014	Tyrosine-protein kinase Lyn
M0RAU0	Advanced glycosylation end product-specific receptor
P20171	GTPase HRas
Q9Z1E1	Flotillin-1
P04157	Receptor-type tyrosine-protein phosphatase C (CD45)
Q4KM97	Tyrosine-protein kinase BLK
Q62940	E3 ubiquitin-protein ligase NEDD4
Q62658	Peptidyl-prolyl cis-trans isomerase FKBP1A
B0BNJ1	Sorcin
Q6P6U0	Tyrosine-protein kinase Fgr

Q63803	Guanine nucleotide-binding protein G(s) subunit alpha isoforms XLas
G3V8A5	Vacuolar protein sorting-associated protein 35
Q5XIH7	Prohibitin 2
P00388	NADPH--cytochrome P450 reductase

ID Term	GO Term	Fold Enriched	Count	*p* -value	FDR
**GO:1902533**	**POSITIVE REGULATION OF INTRACELLULAR SIGNAL TRANSDUCTION**	**1.65**	**78**	**1.87E-5**	**3.6E-2**
**Annotated proteins**					
**Protein Accession**	**Protein Identification**				
D4A465	Ragulator complex protein LAMTOR2				
F1M2P8	Protein kinase C alpha				
Q00238	Intercellular adhesion molecule 1 (ICAM1)				
Q5U204	Ragulator complex protein LAMTOR3				
P0DP29	Calmodulin-1				
Q05030	Platelet-derived growth factor receptor beta				
E9PU37	Palmitoyltransferase				
O88767	Protein/nucleic acid deglycase DJ-1				
Q9Z1Z1	Eukaryotic translation initiation factor 2-alpha kinase 3				
P51577	P2X purinoceptor 4				
P35213	14-3-3 protein beta/alpha				
Q642E3	CDK5 regulatory subunit-associated protein 3, isoform				
Q6AYI1	Probable ATP-dependent RNA helicase DDX5				
O08775	Vascular endothelial growth factor receptor 2				
Q9EQX9	Ubiquitin-conjugating enzyme E2 N				
D3ZAS9	DDRGK domain containing 1				
P49134	Integrin beta 1				
P97546	Neuroplastin				
Q920P6	Adenosine deaminase				
P63095	Guanine nucleotide-binding protein G(s) subunit alpha				
G3V7Q7	Ras GTPase-activating-like protein IQGAP1				
Q9Z270	VAMP-associated protein A				
P07632	Superoxide dismutase [Cu-Zn]				
Q3SWT0	Platelet endothelial cell adhesion molecule (Pecam1)				
P35355	Prostaglandin G/H synthase 2				
Q63116	Mast/stem cell growth factor receptor				
P31044	Phosphatidylethanolamine-binding protein 1				
Q9JLH7	CDK5 regulatory subunit-associated protein 3				
P18418	Calreticulin				
Q6P9V1	CD81 molecule				

Q62636	Ras-related protein Rap-1b
Q9ES40	PRA1 family protein 3
P61589	Transforming protein RhoA
P20417	Protein-tyrosine phosphatase 1B
P63245	Receptor of activated protein C kinase 1 (RACK1)
P67779	Prohibitin
P08644	GTPase KRas
Q2IBC7	Hepatocyte growth factor receptor
P35704	Peroxiredoxin-2
P21708	Mitogen-activated protein kinase 3
P04897	Guanine nucleotide-binding protein G(i) subunit alpha-2
P11762	Galectin-1
Q497A2	Solute carrier family 35 member B2
F1LSN4	Fibroblast growth factor receptor
Q6IRF8	V-type proton ATPase subunit S1
P06762	Heme oxygenase 1
Q8CFN2	Cell division control protein 42 homolog (CDC42)
Q01621	Tyrosine-protein kinase LCK
Q6P791	Ragulator complex protein LAMTOR1
D3ZYM4	Macrophage stimulating 1 receptor
Q68FX4	Hematopoietic cell-specific LYN substrate 1 (hcls1)
P07943	Aldose reductase
P58195	Phospholipid scramblase 1
F1LZX9	Integrin alpha 5
P84817	Mitochondrial fission 1 protein
P62836	Ras-related protein Rap-1a
P62909	40S ribosomal protein S3
O35796	Complement component 1 Q subcomponent-binding protein mitochondrial
M0RCF3	Integrin beta 3
Q66HG9	Mitochondrial antiviral-signaling protein
D3ZF11	Ragulator complex protein LAMTOR5
Q9JJ19	Na(+)/H(+) exchange regulatory cofactor NHE-RF1
Q63486	Ras-related GTP-binding protein A (Rag A)
Q07014	Tyrosine-protein kinase Lyn
P10960	Prosaposin
P97887	Presenilin-1
M0RAU0	Advanced glycosylation end product-specific receptor
P52555	Endoplasmic reticulum resident protein 29
P20171	GTPase HRas
B0BN52	Mitochondrial carrier homolog 2
P04157	Receptor-type tyrosine-protein phosphatase C (CD45)
Q62940	E3 ubiquitin-protein ligase NEDD4
P06765	Platelet factor 4
Q62658	Peptidyl-prolyl cis-trans isomerase FKBP1A

G3V6N2	Transmembrane emp24 domain-containing protein 4
Q63803	Guanine nucleotide-binding protein G(s) subunit alpha isoforms XLas
Q6P6U0	Tyrosine-protein kinase Fgr
Q5XIH7	Prohibitin 2

ISOLATED TERMS

ID Term	GO Term	Fold Enriched	Count	*p* -value	FDR
GO:0098796	MEMBRANE PROTEIN COMPLEX	3.46	187	6.99E-37	1.08E-32

Annotated proteins

Protein Accession	Protein Identification
D4A465	Ragulator complex protein LAMTOR2
D3ZAF6	ATP synthase subunit F mitochondrial
Q9Z2L0	Voltage-dependent anion-selective channel protein 1
Q6P503	V-type proton ATPase subunit D
P25235	Dolichyl-diphosphooligosaccharide protein glycosyltransferase subunit 2
Q4G002	Histocompatibility 2 T region locus 24
P50408	V-type proton ATPase subunit F
B2RZD1	Protein transport protein Sec61 subunit beta
G3V8I4	Syntaxin-4A
P11442	Clathrin heavy chain 1
Q68FY0	Cytochrome b-c1 complex subunit 1 mitochondrial
Q5U204	Ragulator complex protein LAMTOR3
D3ZMR1	Translocase of outer mitochondrial membrane 7
P0DP29	Calmodulin-1
P12075	Cytochrome c oxidase subunit 5B mitochondrial
Q6AYM8	Glycosylphosphatidylinositol anchor attachment 1
P11951	Cytochrome c oxidase subunit 6C2
G3V7L8	V-type proton ATPase subunit E 1 isoform
G3V679	Transferrin receptor protein 1
Q920L2	Succinate dehydrogenase [ubiquinone] flavoprotein subunit mitochondrial
P06686	Sodium/potassium-transporting ATPase subunit alpha-2
P06687	Sodium/potassium-transporting ATPase subunit alpha-3
P06685	Sodium/potassium-transporting ATPase subunit alpha-1
Q6PCU2	V-type proton ATPase subunit E 1
B0BNG0	ER membrane protein complex subunit 2
Q641Y2	NADH dehydrogenase [ubiquinone] iron-sulfur protein 2
Q4FZT0	Stomatin-like protein 2
P49134	Integrin beta 1
Q641Y0	Oligosaccharyl transferase 48 kDa subunit

B2RYD7	STT3B, catalytic subunit of the oligosaccharyltransferase complex
G3V8Q1	Coatomer subunit epsilon
Q561S0	NADH dehydrogenase [ubiquinone] 1 alpha subcomplex subunit 10
P19511	ATP synthase F(0) complex subunit B1 mitochondrial
D3ZJS3	Translocase of outer mitochondrial membrane 6
Q5M7T6	V-type proton ATPase subunit D 1
G3V7M2	Integrin beta 7
D4A0T0	NADH dehydrogenase 1 beta subcomplex subunit 10
G3V6P8	Guanine nucleotide-binding protein gamma-12
Q8SEZ5	Cytochrome c oxidase subunit 2
P21571	ATP synthase-coupling factor 6 mitochondrial
A1L134	Ancient ubiquitous protein 1
G3V6P7	Myosin heavy chain 9
P20411	High affinity immunoglobulin epsilon receptor subunit gamma
P16391	RT1 class I histocompatibility antigen AA alpha chain
P46462	Transitional endoplasmic reticulum ATPase
Q4KM74	Vesicle-trafficking protein SEC22b
F1M5Z5	Anoctamin-6
Q62991	Syntaxin-binding protein 1-like 2
Q62896	Bet1 golgi vesicular membrane trafficking protein
D3ZD11	Signal peptidase complex subunit 2
Q3KRC3	Signal recognition particle receptor alpha
Q2I6B2	V-type proton ATPase subunit A1
Q2I6B1	V-type proton ATPase subunit A2
D4A0E2	NSF attachment protein gamma
Q6MGA3	Antigen peptide transporter 2
F1LNT8	VAMP8
Q6P791	Ragulator complex protein LAMTOR1
Q5U2V8	ER membrane protein complex subunit 3
F1LZX9	Integrin alpha 5
D3ZF12	Signal peptidase complex subunit 3
Q861Q1	MHC class I antigen
D4A0W1	ER membrane protein complex subunit 4
M0RCF3	Integrin beta 3
O35142	Coatomer subunit beta 2
D3ZF11	Ragulator complex protein LAMTOR5
Q63377	Sodium/potassium-transporting ATPase subunit beta-3
Q95II0	MHC class Ib antigen
M0RBJ0	Guanine nucleotide-binding protein gamma-2
Q63584	Transmembrane emp24 domain-containing protein 10
Q3KR86	MICOS complex subunit Mic60
P08753	Guanine nucleotide-binding protein G(k) subunit alpha
P97887	Presenilin-1
Q5XI31	GPI transamidase component PIG-S

P36370	Antigen peptide transporter 1
D3ZWZ1	Integrin alpha X
G3V8F5	Mitochondrial import receptor subunit TOM40 homolog
Q6IN37	GM2 ganglioside activator
F1M779	Clathrin heavy chain
P32551	Cytochrome b-c1 complex subunit 2 mitochondrial
Q5BK32	FAS-associated factor 2
G3V887	V-type proton ATPase subunit A
Q5RKH6	Protein OS-9
G3V667	Integrin alpha 6
P13386	High affinity immunoglobulin epsilon receptor subunit beta
M0RDI5	Mitochondrial calcium uniporter
Q10758	Keratin, type II cytoskeletal 8
P61805	Dolichyl-diphosphooligosaccharide protein glycosyltransferase subunit DAD1
B4F7C9	STT3A, catalytic subunit of the oligosaccharyltransferase complex
P20788	Cytochrome b-c1 complex subunit Rieske
P82471	Guanine nucleotide-binding protein G(q) subunit alpha
Q9R1Z0	Voltage-dependent anion-selective channel protein 3
Q6MG61	Chloride intracellular channel protein 1
G3V9X8	Magnesium transporter protein 1
P54311	Transducin beta-1
B2RYQ2	Serine/threonine-protein phosphatase 2A activator
F1LPK9	Phosphatidylinositol glycan anchor biosynthesis class U protein
P11240	Cytochrome c oxidase subunit 5A mitochondrial
Q64541	Sodium/potassium-transporting ATPase subunit alpha-4
O70257	Syntaxin-7
O88767	Protein/nucleic acid deglycase DJ-1
Q75Q41	Mitochondrial import receptor subunit TOM22 homolog
P31399	ATP synthase subunit D mitochondrial
G3V8L7	Integrin alpha M
P17046	Lysosome-associated membrane glycoprotein 2 (Lamp2)
D3ZUX5	MICOS complex subunit MIC-19
F1LNC4	Vesicle transport through interaction with t-SNAREs homolog 1B
G3V6D3	ATP synthase subunit beta
Q9QYE7	Integrin alpha D
F7FNS3	Derlin
D3ZJX5	Mitochondrial import inner membrane translocase subunit TIM50
P70580	Membrane-associated progesterone receptor component 1
Q66H80	Coatomer subunit delta
G3V7P1	Syntaxin-12
D3ZDR2	Charged multivesicular body protein 6
E9PTI1	V-type proton ATPase subunit H
P63095	Guanine nucleotide-binding protein G(s) subunit alpha
D4ABY2	Coatomer subunit gamma 2

P35435	ATP synthase subunit gamma mitochondrial
D3ZZS8	V-type proton ATPase subunit B, kidney isoform
O35165	Golgi SNAP receptor complex member 2
B2GUV5	V-type proton ATPase subunit G 1
P11598	Protein disulfide-isomerase A3
D4A7Y9	Essential MCU regulator, mitochondrial
G3V6T1	Coatomer subunit alpha
P81155	Voltage-dependent anion-selective channel protein 2
P18418	Calreticulin
Q5XIP2	GPI-anchor transamidase
Q63635	Syntaxin-6
Q9WU70	Syntaxin-binding protein 5
P62815	V-type proton ATPase subunit B
Q9Z2Q7	Syntaxin-8
Q5FVL2	ER membrane protein complex subunit 8
Q5M9I5	Cytochrome b-c1 complex subunit 6 mitochondrial
F1M7T6	Translocon-associated protein subunit gamma
P54921	Alpha-soluble NSF attachment protein (SNAP-alpha)
D3ZG43	NADH dehydrogenase (ubiquinone) iron-sulfur protein 3
Q9Z2J5	VAMP5
G3V8C4	Chloride intracellular channel protein 4
P63039	60 kDa heat shock protein
P10719	ATP synthase subunit beta mitochondrial
P04897	Guanine nucleotide-binding protein G(i) subunit alpha-2
P14659	Heat shock-related 70 kDa protein 2
Q6IRF8	V-type proton ATPase subunit S1
Q08849	Syntaxin-3
P09527	Ras-related protein Rab-7a
B5DEL8	NADH dehydrogenase (Ubiquinone) iron-sulfur protein 5
P29419	ATP synthase subunit E mitochondrial
P10888	Cytochrome c oxidase subunit 4 isoform 1 mitochondrial
Q08851	Syntaxin-5
F1LQ92	Sel-1 homolog 1
Q9JHW5	VAMP7
D4A2H2	Serine palmitoyltransferase 1
O70377	Synaptosomal-associated protein 23 (SNAP-23)
D4A133	V-type proton ATPase catalytic subunit A
D3ZCZ9	NADH dehydrogenase [ubiquinone] iron-sulfur protein 6
Q63486	Ras-related GTP-binding protein A (Rag A)
P21913	Succinate dehydrogenase [ubiquinone] iron-sulfur subunit mitochondrial
Q07014	Tyrosine-protein kinase Lyn
Q6AXV4	Sorting and assembly machinery component 50 homolog
Q4AEF8	Coatomer subunit gamma 1
Q07984	Translocon-associated protein subunit delta

B5DF51	Membrane magnesium transporter 1
D4A994	ER membrane protein complex subunit 1
Q9Z1E1	Flotillin-1
Q3B7D2	Serine palmitoyltransferase 2
P63025	VAMP3
D3ZFK5	Signal peptidase complex subunit 1
P07151	Beta-2-microglobulin
P15999	ATP synthase subunit alpha mitochondrial
P19234	NADH dehydrogenase [ubiquinone] flavoprotein 2 mitochondrial
D3ZQG9	RT1 class I locus CE4
Q64273	Inward rectifier potassium channel 2
P19627	Guanine nucleotide-binding protein G(z) subunit alpha
Q66HF1	NADH-ubiquinone oxidoreductase 75 kDa subunit mitochondrial
B2RYB8	Integrin beta 2
Q5XIH3	NADH dehydrogenase [ubiquinone] flavoprotein 1
Q63803	Guanine nucleotide-binding protein G(s) subunit alpha isoforms XLas
Q9Z2S9	Flotillin-2
P23514	Coatomer subunit beta 1
Q62753	Syntaxin-binding protein 2
G3V8A5	Vacuolar protein sorting-associated protein 35
D3ZP98	Minor histocompatibility antigen H13
Q99JC6	Tapasin
Q9ES21	Phosphatidylinositide phosphatase SAC1
P07153	Dolichyl-diphosphooligosaccharide protein glycosyltransferase subunit 1
Q06647	ATP synthase subunit O mitochondrial
G3V637	Syntaxin-binding protein 2, isoform

ID Term	GO Term	Fold Enriched	Count	*p* -value	FDR
**GO:0061024**	**MEMBRANE ORGANIZATION**	**2.77**	**119**	**2.58E-24**	**5.06E-21**

Annotated proteins

Protein Accession	Protein Identification
G3V8I4	Syntaxin-4A
B2RZD1	Protein transport protein Sec61 subunit beta
P11442	Clathrin heavy chain 1
Q6QBQ4	Phospholipid scramblase 3
D3ZMR1	Translocase of outer mitochondrial membrane 7
P61107	Ras-related protein Rab-14
D4ADS9	EFR3 homolog A
Q05962	ADP/ATP translocase 1
Q6PCU4	Emerin
P61972	Nuclear transport factor 2
Q4FZT0	Stomatin-like protein 2

P49134	Integrin beta 1
Q62733	Lamina-associated polypeptide 2 isoform beta
Q9Z270	VAMP-associated protein A
F1M8X9	Golgi brefeldin A-resistant guanine nucleotide exchange factor 1
P07632	Superoxide dismutase [Cu-Zn]
E9PU78	Isoform Bcl-X(S) of Bcl-2-like protein 1
E9PU77	Synaptotagmin-like protein 3
Q6NYB7	Ras-related protein Rab-1a
F1LQ81	Vesicle-fusing ATPase
G3V7M2	Integrin beta 7
Q9Z269	VAMP-associated protein B
P63245	Receptor of activated protein C kinase 1 (RACK1)
G3V6P7	Myosin heavy chain 9
P20411	High affinity immunoglobulin epsilon receptor subunit gamma
Q4KM74	Vesicle-trafficking protein SEC22b
Q5BJY9	Keratin, type I cytoskeletal 18
F1M5Z5	Anoctamin-6
P70550	Ras-related protein Rab-8b
Q62896	Bet1 golgi vesicular membrane trafficking protein
Q32PX6	Rho-related GTP-binding protein RhoG
Q5XI41	Translocating chain-associated membrane protein 1
Q3KRC3	Signal recognition particle receptor alpha
A1L1L6	Mitochondrial Rho GTPase 1
O88600	Heat shock 70 kDa protein 4
D4A0E2	NSF attachment protein gamma
F1LNT8	VAMP8
Q78P65	Rap2A-like protein
Q63377	Sodium/potassium-transporting ATPase subunit beta-3
Q9JJ19	Na(+)/H(+) exchange regulatory cofactor NHE-RF1
Q63584	Transmembrane emp24 domain-containing protein 10
Q3KR86	MICOS complex subunit Mic60
P08753	Guanine nucleotide-binding protein G(k) subunit alpha
Q9JLT0	Myosin heavy chain 10
Q9QYW3	MOB-like protein phocein
P82995	Heat shock protein HSP 90-alpha
F1M779	Clathrin heavy chain
Q6IN37	GM2 ganglioside activator
P0C1Q3	Lysophosphatidylcholine acyltransferase 2
B0BN52	Mitochondrial carrier homolog 2
P61621	SEC61 alpha-1
P50398	Rab GDP dissociation inhibitor alpha
Q7TSA0	Mitochondrial Rho GTPase 2
Q5XIN6	Mitochondrial proton/calcium exchanger protein
Q6RUV5	Ras-related C3 botulinum toxin substrate 1 (Rac1)

Q07936	Annexin A2
E9PT65	Radixin
O35152	BET1-like protein
G3V6W2	Prolactin regulatory element-binding protein
P63018	Heat shock cognate 71 kDa protein
Q498C8	Protein RER1
D4A2Z6	Translocation protein SEC63 homolog
P63012	Ras-related protein Rab-3a
Q7TP42	Translocation protein SEC62
O70257	Syntaxin-7
Q75Q41	Mitochondrial import receptor subunit TOM22 homolog
D3ZUX5	MICOS complex subunit MIC-19
O35509	Ras-related protein Rab-11b
F1LNC4	Vesicle transport through interaction with t-SNAREs homolog 1B
D3ZJX5	Mitochondrial import inner membrane translocase subunit TIM50
G3V7P1	Syntaxin-12
D3ZDR2	Charged multivesicular body protein 6
Q91ZN1	Coronin-1A
O35165	Golgi SNAP receptor complex member 2
P26453	Basigin
D3ZGV2	Peroxisomal membrane protein PEX16
Q5RKJ9	Ras-related protein Rab-10
Q5PQX1	Torsin-1A-interacting protein 1
Q3MIE4	Synaptic vesicle membrane protein VAT-1 homolog
Q63635	Syntaxin-6
Q09073	ADP/ATP translocase 2
Q5I0E7	Transmembrane emp24 domain-containing protein 9
Q9Z2Q7	Syntaxin-8
G3V6E1	Myosin heavy chain 2
Q5XIP9	Transmembrane protein 43 (Protein LUMA)
O35987	NSFL1 cofactor p47
F1M7T6	Translocon-associated protein subunit gamma
P54921	Alpha-soluble NSF attachment protein (SNAP-alpha)
Q9Z2J5	VAMP5
A3RLA8	Low affinity immunoglobulin gamma Fc region receptor II
Q08849	Syntaxin-3
P09527	Ras-related protein Rab-7a
Q08851	Syntaxin-5
Q5XI04	Erythrocyte band 7 integral membrane protein
Q63151	Long chain fatty acid CoA ligase 3
P58195	Phospholipid scramblase 1
Q63524	Transmembrane emp24 domain-containing protein 2
Q9JHW5	VAMP7
P84817	Mitochondrial fission 1 protein

Q4V7D1	Translocon-associated protein subunit alpha
O70377	Synaptosomal-associated protein 23 (SNAP-23)
Q68FP1	Gelsolin
Q63483	Ras-related protein Rab-38
Q6AXV4	Sorting and assembly machinery component 50 homolog
P27881	Hexokinase-2
M0RAU0	Advanced glycosylation end product-specific receptor
P31977	Ezrin
Q9Z1E1	Flotillin-1
P63025	VAMP3
P13832	Myosin regulatory light chain RLC-A
P07150	Annexin A1
Q6AYQ1	Golgin subfamily A member 7
B2RYB8	Integrin beta 2
Q6MG49	Large proline-rich protein BAG6
Q9Z2S9	Flotillin-2
G3V8A5	Vacuolar protein sorting-associated protein 35
Q5BQE6	Delta(24)-sterol reductase
D4A5K6	Zinc metallopeptidase STE24
P35280	Ras-related protein Rab-8a

ID Term	GO Term	Fold Enriched	Count	*p* -value	FDR
**GO:0043227**	**MEMBRANE-BOUNDED ORGANELLE**	**1.53**	**863**	**8.75E-111**	**9.36E-109**
**Annotated proteins**					
**Protein Accession**			**Protein Identification**		
Q10473			Pol+AZ716:BB737ypeptide N-acetylgalactosaminyltransferase 1		
P50408			V-type proton ATPase subunit F		
Q62876			Synaptogyrin-1		
Q63716			Peroxiredoxin-1		
P0DP29			Calmodulin-1		
Q8R4A1			ERO1-like protein alpha		
D3ZXF9			39S ribosomal protein L12, mitochondrial		
G3V852			Talin-1		
B0BN02			Metaxin 1		
P13084			Nucleophosmin		
Q920L2			Succinate dehydrogenase [ubiquinone] flavoprotein subunit mitochondrial		
Q641Y2			NADH dehydrogenase [ubiquinone] iron-sulfur protein 2		
D3ZAS9			DDRGK domain containing 1		
Q9ESV6			Glyceraldehyde-3-phosphate dehydrogenase testis-specific		
Q641Y0			Oligosaccharyl transferase 48 kDa subunit		
Q5XI52			Vezatin		
Q66H15			Regulator of microtubule dynamics protein 3		

Q9Z270	VAMP-associated protein A
P07632	Superoxide dismutase [Cu-Zn]
Q66H18	Synaptophysin-like 1
Q10468	Beta-1,4 N-acetylgalactosaminyltransferase 1
G3V9U7	Sarcoplasmic/endoplasmic reticulum calcium ATPase 3 (SERCA3)
Q7TP15	S-methyl-5'-thioadenosine phosphorylase
P20417	Protein-tyrosine phosphatase 1B
Q01205	2-oxoglutarate dehydrogenase complex component E2
G3V7U6	Calpain 5
P68511	14-3-3 protein eta
Q9Z269	VAMP-associated protein B
Q4V888	Type 2 phosphatidylinositol 4,5-bisphosphate 4-phosphatase
P21571	ATP synthase-coupling factor 6 mitochondrial
F1M5Z5	Anoctamin-6
D3ZRN3	Beta-actin-like protein 2
P51796	Chloride intracellular channel protein 5
Q62896	Bet1 golgi vesicular membrane trafficking protein
Q5XI41	Translocating chain-associated membrane protein 1
O88989	Malate dehydrogenase cytoplasmic
Q9QX80	Heterogeneous nuclear ribonucleoprotein A/B
P27615	Lysosome membrane protein 2 (CD36)
P06762	Heme oxygenase 1
P06761	Endoplasmic reticulum chaperone BiP
Q9QYV0	Disintegrin and metalloproteinase domain-containing protein 15
G3V878	Signal peptidase complex catalytic subunit SEC11
B0K020	CDGSH iron-sulfur domain-containing protein 1
P63102	14-3-3 protein zeta/delta
Q9EST6	Acidic leucine-rich nuclear phosphoprotein 32 family member B
Q78P65	Rap2A-like protein
D3ZUZ4	Glycosaminoglycan xylosylkinase
Q4V899	Transmembrane protein 165
Q5XI34	Serine/threonine-protein phosphatase 2A 65 kDa regulatory subunit A alpha isoform
Q5XI32	F-actin-capping protein subunit beta
Q3KR86	MICOS complex subunit Mic60
Q5XI31	GPI transamidase component PIG-S
Q9QYW3	MOB-like protein phocein
P52555	Endoplasmic reticulum resident protein 29
O88994	Mitochondrial amidoxime reducing component 2
Q5XI38	Lymphocyte cytosolic protein 1
Q5XI36	CD97
G3V9W2	Tyrosine-protein kinase JAK1
G3V887	V-type proton ATPase subunit A
B0K010	Thioredoxin domain-containing 17
M0RDI5	Mitochondrial calcium uniporter

P06765	Platelet factor 4
P05197	Elongation factor 2
G3V9X8	Magnesium transporter protein 1
Q6P685	Eukaryotic translation initiation factor 2 subunit 2
D3ZD83	Major facilitator superfamily domain-containing protein 10
P63322	Ras-related protein Ral-A
Q63396	Activated RNA polymerase II transcriptional coactivator p15
P54313	Transducin beta-2
F1MA89	Cyclin Y
P54311	Transducin beta-1
O70257	Syntaxin-7
O08984	Lamin-B receptor
O88767	Protein/nucleic acid deglycase DJ-1
D4AEP0	Adenylosuccinate synthetase isozyme 2
P51577	P2X purinoceptor 4
D3ZUX5	MICOS complex subunit MIC-19
P35213	14-3-3 protein beta/alpha
F7FNS3	Derlin
O08775	Vascular endothelial growth factor receptor 2
Q9EQX9	Ubiquitin-conjugating enzyme E2 N
G3V7P1	Syntaxin-12
P15865	Histone H1.4
Q920P6	Adenosine deaminase
G3V9Y2	Phospholipid phosphatase 1
G3V7Q7	Ras GTPase-activating-like protein IQGAP1
Q5EAP4	Guanine nucleotide binding protein alpha-14
P63331	Serine/threonine-protein phosphatase 2A catalytic subunit alpha isoform
Q63116	Mast/stem cell growth factor receptor
D3ZSY5	keratin 84
Q64244	ADP-ribosyl cyclase/cyclic ADP-ribose hydrolase 1
P81155	Voltage-dependent anion-selective channel protein 2
P21531	60S ribosomal protein L3
B0BN30	Mitochondrial carrier homolog 1
P31000	Vimentin
O88777	Presenilin-2
D3ZL85	Cytochrome c heme lyase
F7EPH4	Inorganic Pyrophosphatase
Q64654	Lanosterol 14-alpha demethylase
P08644	GTPase KRas
Q63965	Sideroflexin-1
B0K031	60S ribosomal protein L7, isoform
F1LN88	Aldehyde dehydrogenase
F1M7T6	Translocon-associated protein subunit gamma
Q05982	Nucleoside diphosphate kinase A

P04897	Guanine nucleotide-binding protein G(i) subunit alpha-2
Q8VHF5	Citrate synthase mitochondrial
Q08849	Syntaxin-3
Q8CFN2	Cell division control protein 42 homolog (CDC42)
Q5XI86	Peptidyl-tRNA hydrolase 2
Q8CHN6	Sphingosine-1-phosphate lyase 1
Q08851	Syntaxin-5
Q63151	Long chain fatty acid CoA ligase 3
Q1H5H1	Thioredoxin reductase-like selenoprotein T
D3ZCZ9	NADH dehydrogenase [ubiquinone] iron-sulfur protein 6
Q07014	Tyrosine-protein kinase Lyn
Q5XI78	2-oxoglutarate dehydrogenase complex component E1
Q5XI77	Annexin A11
Q5XI73	Rho GDP dissociation inhibitor 1
P07151	Beta-2-microglobulin
P07150	Annexin A1
Q52KJ9	Thioredoxin-related transmembrane protein 1
Q64273	Inward rectifier potassium channel 2
Q4QQS6	Dolichyl-phosphate beta-glucosyltransferase
B2RYB8	Integrin beta 2
Q5HZE2	Transmembrane protein 120 A
F1LW77	Ras-related protein Rab-33b
P07153	Dolichyl-diphosphooligosaccharide protein glycosyltransferase subunit 1
F1LVV4	Protein RCC2
Q5U362	Annexin A4
B2RYC9	Glucosylceramidase
E9PTU4	Myosin heavy chain 11
P11442	Clathrin heavy chain 1
Q6QBQ4	Phospholipid scramblase 3
B0BN81	Ribosomal protein S5
P12075	Cytochrome c oxidase subunit 5B mitochondrial
B2GV24	E3 UFM1-protein ligase 1
Q05030	Platelet-derived growth factor receptor beta
G3V7L8	V-type proton ATPase subunit E 1 isoform
D4A7U6	U6 snRNA-associated Sm-like protein LSm3
Q62662	Tyrosine-protein kinase FRK
Q6TEK4	Vitamin K epoxide reductase complex subunit 1
D4A7U1	Zyxin
Q05962	ADP/ATP translocase 1
Q6PCU4	Emerin
P61972	Nuclear transport factor 2
Q6PCU2	V-type proton ATPase subunit E 1
Q5PQL7	Integral membrane protein 2C
P50475	Alanine--tRNA ligase cytoplasmic

B2RYD7	STT3B, catalytic subunit of the oligosaccharyltransferase complex
Q810F4	Protein FAM3C
E9PU78	Isoform Bcl-X(S) of Bcl-2-like protein 1
P31044	Phosphatidylethanolamine-binding protein 1
E9PU77	Synaptotagmin-like protein 3
Q5RJR8	Leucine-rich repeat-containing protein 59
Q9JLH7	CDK5 regulatory subunit-associated protein 3
Q4QQV0	Tubulin beta-6
Q6MGB4	H2-K region expressed gene 4, rat orthologue
Q3B7V5	Ras-related protein Rab-2b
G3V7M2	Integrin beta 7
B1WBW4	Armadillo repeat-containing protein 10
D3ZNG3	Signal peptide peptidase-like 2A
Q9Z1J7	Amino acid transporter
G3V9N0	Polyadenylate-binding protein 4
E9PTW1	Secretory carrier-associated membrane protein
G3V9N9	alpha-1,2-Mannosidase
Q6MGA3	Antigen peptide transporter 2
Q64232	Very-long-chain enoyl-CoA reductase
Q4KM49	Tyrosine--tRNA ligase cytoplasmic
Q5RK10	60S ribosomal protein L13a, isoform
Q5U2V8	ER membrane protein complex subunit 3
B1WC50	RNA-binding protein EWS-like
P61227	Ras-related protein Rap-2b
Q63377	Sodium/potassium-transporting ATPase subunit beta-3
M0RBJ0	Guanine nucleotide-binding protein gamma-2
Q64428	Trifunctional enzyme subunit alpha
Q63942	Ras-related protein Rab-3d
Q63584	Transmembrane emp24 domain-containing protein 10
Q5PQM0	Transmembrane protein 168
Q6EV76	Alpha-(1,6)-fucosyltransferase
F1LU48	Endoplasmic reticulum-golgi intermediate compartment 1
Q63945	Protein SET
D4A9X7	Hypermethylated in cancer 2 protein
P32551	Cytochrome b-c1 complex subunit 2 mitochondrial
B0BN52	Mitochondrial carrier homolog 2
E9PTX9	Solute carrier family 12 member 2
P61621	SEC61 alpha-1
B1WC67	Solute carrier family 25 member 24
F1LRK1	Sodium/potassium-transporting ATPase subunit alpha
Q7TSA0	Mitochondrial Rho GTPase 2
B1WC61	Acyl-CoA dehydrogenase family member 9
O89035	Solute carrier family 25 member 10
Q9WVB1	Ras-related protein Rab-6a

Q62839	Golgin subfamily A member 2
B2RYG6	Ubiquitin thioesterase OTUB1
Q3T1K5	F-actin-capping protein subunit alpha-2
Q7TP42	Translocation protein SEC62
P68370	Tubulin alpha-1A
Q5U316	Ras-related protein Rab-35
D3ZF97	Endoplasmic reticulum lectin 1
P11167	Solute carrier family 2, facilitated glucose transporter member 1
P04642	L-lactate dehydrogenase A chain
P62828	GTP-binding nuclear protein Ran
P70583	Deoxyuridine 5'-triphosphate nucleotidohydrolase
P70580	Membrane-associated progesterone receptor component 1
P23358	60S ribosomal protein L12
Q3T1J1	Eukaryotic translation initiation factor 5A-1
D4AEG2	Ras-related protein Rab-32
Q6P3V9	60S ribosomal protein L4
Q6P3V8	Eukaryotic initiation factor 4A-I
F1LP82	Ras-related protein Rab-2a
Q62636	Ras-related protein Rap-1b
Q3MIE4	Synaptic vesicle membrane protein VAT-1 homolog
P04636	Malate dehydrogenase mitochondrial
G3V7I3	Manganese-transporting ATPase 13A1
Q5U300	Ubiquitin-like modifier-activating enzyme 1
G3V7I0	Peroxiredoxin-3
P62815	V-type proton ATPase subunit B
Q4KLL4	Transmembrane 9 superfamily member 4
Q63198	Contactin 1
Q0R3W9	GTPase, IMAP family member 1
D3ZAY7	Band 4.1-like protein 2
E9PT29	ATP-dependent RNA helicase DDX17
Q925S8	ATP-dependent zinc metalloprotease YME1L1
O88941	Mannosyl-oligosaccharide glucosidase
Q4V7D9	Acid sphingomyelinase-like phosphodiesterase 3B
Q6S395	Plectin
F1LMP7	Granulins
Q3B7U9	Peptidyl-prolyl cis-trans isomerase FKBP8
Q5XI04	Erythrocyte band 7 integral membrane protein
Q4V7D1	Translocon-associated protein subunit alpha
P62804	Histone H4
P12001	60S ribosomal protein L18
G3V7J7	Eukaryotic translation initiation factor 5A-2
Q2YDU8	Protein spinster homolog 1
P12928	Pyruvate kinase PKLR
P13832	Myosin regulatory light chain RLC-A

G3V9S3	Tyrosine-protein phosphatase non-receptor type 13
B1WBY5	DnaJ homolog subfamily C member 11
Q62658	Peptidyl-prolyl cis-trans isomerase FKBP1A
Q5EAJ6	Inhibitor kappa-B kinase-interacting protein
B1WBY7	Erlin-1
D4A5K6	Zinc metallopeptidase STE24
P13838	Leukosialin
G3V637	Syntaxin-binding protein 2, isoform
M0RD75	40S ribosomal protein S6
Q505J9	ATPase family AAA domain-containing protein 1
P62859	40S ribosomal protein S28
Q6IN24	Galectin-8
Q6PEC3	Protein YIF1B
Q6IFV6	Keratin 35
F1LMK0	A-kinase anchoring protein 1 isoform 2
P09895	60S ribosomal protein L5
D3ZKB9	Teashirt homolog 3
P24049	60S ribosomal protein L17
P10111	Peptidyl-prolyl cis-trans isomerase A
Q5XIE0	Acidic leucine-rich nuclear phosphoprotein 32 family member E
Q4FZT0	Stomatin-like protein 2
P62260	14-3-3 protein epsilon
Q5XIE8	Integral membrane protein 2B
Q6IG03	Keratin, type II cytoskeletal 73
Q6IG02	Keratin, type II cytoskeletal 2 epidermal
Q6IG01	Keratin, type II cytoskeletal 1B
Q6IG00	Keratin, type II cytoskeletal 4
Q3SWT0	Platelet endothelial cell adhesion molecule (Pecam1)
Q6IN10	Endothelin-converting enzyme 1 Isoform
Q6IG05	Keratin, type II cytoskeletal 75
Q6IFW6	Keratin, type I cytoskeletal 10
P09495	Tropomyosin alpha-4
Q5XIF6	Tubulin alpha-4A
F1M0J3	Sodium bicarbonate cotransporter 3, isophorm 4
P62250	40S ribosomal protein S16
Q91Y81	Septin-2
P62634	Cellular nucleic acid-binding protein
P70550	Ras-related protein Rab-8b
Q99P74	Ras-related protein Rab-27b
Q99P75	Ras-related protein Rab-9a
Q6IG12	Keratin, type II cytoskeletal 7
P35704	Peroxiredoxin-2
Q2I6B2	V-type proton ATPase subunit A1
Q2I6B1	V-type proton ATPase subunit A2

P62630	Elongation factor 1-alpha 1
F1LMM5	Arachidonate 5-lipoxygenase
P62246	40S ribosomal protein S15a
P62832	60S ribosomal protein L23
P05712	Ras-related protein Rab-2a
D4A6A2	Heterogeneous nuclear ribonucleoprotein A3-like
P62836	Ras-related protein Rap-1a
P62243	40S ribosomal protein S8
E9PSV5	Phosphoserine aminotransferase
Q01827	Vesicular amine transporter 2
Q63413	Spliceosome RNA helicase Ddx39b
Q5BJK8	Golgi integral membrane protein 4
P36370	Antigen peptide transporter 1
P82995	Heat shock protein HSP 90-alpha
Q6IN37	GM2 ganglioside activator
Q5XID0	Protein YIPF5
P05708	Hexokinase-1
P80254	D-dopachrome decarboxylase
Q63610	Tropomyosin alpha-3
Q6IFZ5	Keratin, type II cytoskeletal 2 oral
Q5VLR5	Endoplasmic reticulum resident protein 44
B4F7C9	STT3A, catalytic subunit of the oligosaccharyltransferase complex
Q5HZY0	Erasin
Q5XID7	Armadillo repeat-containing X-linked protein 3
B4F7C2	Tubulin beta-4A
E9PT65	Radixin
P58405	Striatin-3
Q6MG61	Chloride intracellular channel protein 1
Q498C8	Protein RER1
Q6TXF3	Acyl-CoA-binding protein
B2RZ27	SH3 domain-binding glutamic acid-rich-like protein 3
F1LMG7	Leucine-rich repeat-containing protein 7
B2RYQ2	Serine/threonine-protein phosphatase 2A activator
E9PU37	Palmitoyltransferase
P05982	NAD(P)H dehydrogenase [quinone] 1
Q04462	Valine--tRNA ligase
P62898	Cytochrome c somatic
P04166	Cytochrome b5 type B
P69897	Tubulin beta-5
Q642E3	CDK5 regulatory subunit-associated protein 3, isoform
Q5U2X6	Coiled-coil domain-containing protein 47
P62890	60S ribosomal protein L30
P63095	Guanine nucleotide-binding protein G(s) subunit alpha
P20291	Arachidonate 5-lipoxygenase-activating protein

P52296	Importin subunit beta-1
D3ZTJ0	Protein XRP2
Q4QRB4	Tubulin beta-3
D4A1D8	Surfeit 4
Q6P9V9	Tubulin alpha-1B
M0RCS2	Transducin beta-like protein 2
D4A7Y9	Essential MCU regulator, mitochondrial
Q5PQX1	Torsin-1A-interacting protein 1
Q6P9V1	CD81 molecule
Q63635	Syntaxin-6
Q5RJM1	3-oxo-5-alpha-steroid 4-dehydrogenase 3
P13221	Aspartate aminotransferase cytoplasmic
Q09073	ADP/ATP translocase 2
P54921	Alpha-soluble NSF attachment protein (SNAP-alpha)
P25113	Phosphoglycerate mutase 1
P09626	Potassium-transporting ATPase alpha chain 1
D3ZG43	NADH dehydrogenase (ubiquinone) iron-sulfur protein 3
P61983	14-3-3 protein gamma
Q794E4	Heterogeneous nuclear ribonucleoprotein F
P49242	40S ribosomal protein S3a
Q75WE7	Von Willebrand factor A domain-containing protein 5A
B0BNA5	Coactosin-like protein
P02401	60S acidic ribosomal protein P2
F7FKI5	Pyruvate dehydrogenase component E1 alpha
Q5U2Z8	Elongation of very long chain fatty acids protein 1
Q5XIG4	OCIA domain-containing protein 1
P48500	Triosephosphate isomerase
Q5U2Z3	Nucleosome assembly protein 1-like 4
Q5SGE0	Leucine-rich PPR motif-containing protein mitochondrial
P23640	Ras-related protein Rab-27a
Q4V8F3	Rhomboid-related protein 4
O70595	ATP-binding cassette sub-family B member 6
B5DF51	Membrane magnesium transporter 1
Q6IFU9	keratin 16
Q6P9T8	Tubulin beta-4B
Q6IFU8	Keratin, type I cytoskeletal 17
Q5RJK5	Chromobox 3
P04157	Receptor-type tyrosine-protein phosphatase C (CD45)
G3V7A5	Low density lipoprotein receptor isoform
Q6IFV3	Keratin, type I cytoskeletal 15
Q6IFV1	Keratin, type I cytoskeletal 14
Q5XIH3	NADH dehydrogenase [ubiquinone] flavoprotein 1
P20070	NADH-cytochrome b5 reductase 3
Q6MG49	Large proline-rich protein BAG6

Q5XIH7	Prohibitin 2
P11348	Dihydropteridine reductase
D3ZP98	Minor histocompatibility antigen H13
D4A465	Ragulator complex protein LAMTOR2
Q5I0G4	Glycine--tRNA ligase
Q642A2	Type-1 angiotensin II receptor-associated protein
Q6AZ61	Probable lysosomal cobalamin transporter
Q5M843	2-oxoglutarate and iron-dependent oxygenase domain-containing protein 3
Q00238	Intercellular adhesion molecule 1 (ICAM1)
Q6AY20	Cation-dependent mannose-6-phosphate receptor
D3ZMR1	Translocase of outer mitochondrial membrane 7
Q6AXQ0	SUMO-activating enzyme subunit 1
Q6AY18	GTP-binding protein SAR1a
P19804	Nucleoside diphosphate kinase B
B0BNG0	ER membrane protein complex subunit 2
B0BNG3	Vesicular integral-membrane protein VIP36
Q5M7T4	Protein YIPF4
Q5I0H4	Calcium load-activated calcium channel (CLAC channel)
Q561S0	NADH dehydrogenase [ubiquinone] 1 alpha subcomplex subunit 10
Q6NYB7	Ras-related protein Rab-1a
G3V8Z1	Linker for activation of T-cells family member 2 (NTAL)
P19814	Trans-Golgi network integral membrane protein TGN38
P17074	40S ribosomal protein S19
Q5M7T6	V-type proton ATPase subunit D 1
B5DEN5	Eukaryotic translation elongation factor 1 beta 2
P84903	Stromal interaction molecule 1 (STIM1)
P63245	Receptor of activated protein C kinase 1 (RACK1)
Q8SEZ5	Cytochrome c oxidase subunit 2
A1L134	Ancient ubiquitous protein 1
Q32PX6	Rho-related GTP-binding protein RhoG
P05426	60S ribosomal protein L7
Q01621	Tyrosine-protein kinase LCK
Q6AXN3	Transmembrane emp24 domain-containing protein 5
F1LZX9	Integrin alpha 5
Q9JJ19	Na(+)/H(+) exchange regulatory cofactor NHE-RF1
Q4V8C2	Centromere/kinetochore protein zw10 homolog
D3Z9L0	Acylglycerol kinase
Q6MG08	ATP-binding cassette sub-family F member 1
P63269	Actin gamma-enteric smooth muscle
D4AD37	Inositol monophosphatase 3
P15429	Beta-enolase
Q63692	Hsp90 co-chaperone Cdc37
P97675	Ectonucleotide pyrophosphatase/phosphodiesterase family member 3
Q5XIA8	Growth hormone-inducible transmembrane protein

P0C5H9	Mesencephalic astrocyte-derived neurotrophic factor
Q5M7W6	Protein FAM234A
G3V6W2	Prolactin regulatory element-binding protein
P35289	Ras-related protein Rab-15
Q4FZX7	Signal recognition particle receptor beta
D3ZZR9	Peptidylprolyl isomerase
P63018	Heat shock cognate 71 kDa protein
Q5XIA1	Nicalin
P63012	Ras-related protein Rab-3a
P35565	Calnexin
Q9R0C5	Polypeptide N-acetylgalactosaminyltransferase 7
G3V784	ADP-dependent glucokinase
B0BNK1	Ras-related protein Rab-5c
Q75Q41	Mitochondrial import receptor subunit TOM22 homolog
Q9Z1Z1	Eukaryotic translation initiation factor 2-alpha kinase 3
P17046	Lysosome-associated membrane glycoprotein 2 (Lamp2)
P35571	Glycerol-3-phosphate dehydrogenase mitochondrial
O70521	Regulator of G-protein signaling 19
D3ZDR2	Charged multivesicular body protein 6
E9PTI1	V-type proton ATPase subunit H
P85108	Tubulin beta-2A
Q9WTR7	SEC11 homolog C
F1LX07	Solute carrier family 25 member 12
D3ZZS8	V-type proton ATPase subunit B, kidney isoform
D4AAE9	CDGSH iron-sulfur domain-containing protein 2
P26453	Basigin
Q08163	Adenylyl cyclase-associated protein 1
B0BNL4	Heme transporter HRG1
Q5M860	Rho GDP dissociation inhibitor 2
Q8CGU6	Nicastrin
P63039	60 kDa heat shock protein
A0JN29	Lunapark ER junction formation factor
D4A3S8	NOL1/NOP2/Sun domain family member 2
Q6IRF8	V-type proton ATPase subunit S1
B5DEL8	NADH dehydrogenase (Ubiquinone) iron-sulfur protein 5
P35952	Low-density lipoprotein receptor
Q9Z1X1	Extended synaptotagmin 1
P14562	Lysosome-associated membrane glycoprotein 1 (Lamp1)
Q91ZQ0	Vacuole membrane protein 1
P27881	Hexokinase-2
P49432	Pyruvate dehydrogenase component E1 beta
D3ZP13	Sulfhydryl oxidase 2
Q9ER34	Aconitate hydratase mitochondrial
Q3B7D2	Serine palmitoyltransferase 2

P63025	VAMP3
Q6IMF3	Keratin, type II cytoskeletal 1
A0JN30	Canopy 2 homolog
D3ZFK5	Signal peptidase complex subunit 1
Q920J4	Thioredoxin-like protein 1
B0BNJ1	Sorcin
P19627	Guanine nucleotide-binding protein G(z) subunit alpha
G3V9A3	14-3-3 protein sigma
P35280	Ras-related protein Rab-8a
Q62720	Zinc transporter 1
D3ZAF6	ATP synthase subunit F mitochondrial
Q5BK85	Transmembrane emp24 domain-containing protein 1
Q6P503	V-type proton ATPase subunit D
Q5XIU9	Membrane-associated progesterone receptor component 2
B5DEH2	Erlin-2
F1M2P8	Protein kinase C alpha
Q62726	Serine/threonine-protein kinase ICK
P21961	Mast cell carboxypeptidase A
Q5XIU4	B-cell receptor-associated protein 29
P26772	10 kDa heat shock protein mitochondrial
Q5U204	Ragulator complex protein LAMTOR3
Q6AYM8	Glycosylphosphatidylinositol anchor attachment 1
D3ZTX0	Transmembrane emp24 domain-containing protein 7
P50503	Hsc70-interacting protein
P06686	Sodium/potassium-transporting ATPase subunit alpha-2
P06687	Sodium/potassium-transporting ATPase subunit alpha-3
O08623	Sequestosome-1
P06685	Sodium/potassium-transporting ATPase subunit alpha-1
G3V993	SAM domain, SH3 domain and nuclear localization signals, 1 (SAMNS1)
Q62733	Lamina-associated polypeptide 2 isoform beta
F1M8X9	Golgi brefeldin A-resistant guanine nucleotide exchange factor 1
Q6AYN2	Store-operated calcium entry-associated regulatory factor (SARAF)
G3V6H5	Solute carrier family 25 member 11
G3V6H0	Ras-related protein Rab-1b
P27139	Carbonic anhydrase 2
Q66H50	Fatty acyl-CoA reductase 1
D3ZJS3	Translocase of outer mitochondrial membrane 6
Q9ES40	PRA1 family protein 3
P61589	Transforming protein RhoA
P67779	Prohibitin
Q5FVF4	Transmembrane 7 superfamily member 3
Q5BJY9	Keratin, type I cytoskeletal 18
G3V6G5	F-box-like/WD repeat-containing protein TBL1X
Q62991	Syntaxin-binding protein 1-like 2

O08651	D-3-phosphoglycerate dehydrogenase
O88884	A-kinase anchor protein 1, mitochondrial
P11762	Galectin-1
A1L1L6	Mitochondrial Rho GTPase 1
Q6AYK6	Calcyclin-binding protein
F1LT70	Pantothenate kinase 2
Q66H21	Cholinephosphotransferase 1
D3ZYM4	Macrophage stimulating 1 receptor
P07943	Aldose reductase
P32089	Citrate transport protein
M0RCF3	Integrin beta 3
G3V8V4	Ceramide synthase 2
P97887	Presenilin-1
P83732	60S ribosomal protein L24
Q5XIV1	Phosphoglycerate kinase 2
Q5BJZ3	Nicotinamide nucleotide transhydrogenase
P04764	Alpha-enolase
Q10758	Keratin, type II cytoskeletal 8
Q07936	Annexin A2
Q64578	Sarcoplasmic/endoplasmic reticulum calcium ATPase 1 (SERCA1)
Q9R1Z0	Voltage-dependent anion-selective channel protein 3
P85968	6-phosphogluconate dehydrogenase decarboxylating
Q6AYQ9	Peptidyl-prolyl cis-trans isomerase C
Q6PDU1	Serine/arginine-rich splicing factor 2
Q6AYQ4	Transmembrane protein 109
Q64541	Sodium/potassium-transporting ATPase subunit alpha-4
P34058	Heat shock protein HSP 90-beta
D3ZJX5	Mitochondrial import inner membrane translocase subunit TIM50
Q66H80	Coatomer subunit delta
P47819	Glial fibrillary acidic protein
Q91ZN1	Coronin-1A
P04785	Protein disulfide-isomerase
Q63016	Large neutral amino acids transporter small subunit 1
O35165	Golgi SNAP receptor complex member 2
R9PXR4	Mitochondrial import receptor subunit TOM70
P85971	6-phosphogluconolactonase
P12368	cAMP-dependent protein kinase type II-alpha regulatory subunit
Q9WU70	Syntaxin-binding protein 5
Q5I0E7	Transmembrane emp24 domain-containing protein 9
Q9Z2Q7	Syntaxin-8
A1L1J8	Ras-related protein Rab-5b
F1LM33	Leucine-rich PPR motif-containing protein mitochondrial isoform
P38983	40S ribosomal protein SA
O35987	NSFL1 cofactor p47

A1L1J9	Lipase maturation factor 2
F7FFV2	keratin 5
F1LQR8	Inositol 1,4,5-trisphosphate receptor type 2
P21708	Mitogen-activated protein kinase 3
P04797	Glyceraldehyde-3-phosphate dehydrogenase
O35567	Bifunctional purine biosynthesis protein PURH
G3V936	Citrate synthase
F1LZW6	Solute carrier family 25 member 13
Q9EPV5	Apoptotic protease-activating factor 1
Q5I0D1	Glyoxalase domain-containing protein 4
Q53B90	Ras-related protein Rab-43
P58195	Phospholipid scramblase 1
Q9JHW5	VAMP7
Q9JI85	Nucleobindin-2
D4A2H2	Serine palmitoyltransferase 1
P01830	Thy-1 membrane glycoprotein
O70377	Synaptosomal-associated protein 23 (SNAP-23)
Q68FP1	Gelsolin
D4A133	V-type proton ATPase catalytic subunit A
F1LNX2	Calcium release-activated calcium channel protein 1 (Orai1)
P21913	Succinate dehydrogenase [ubiquinone] iron-sulfur subunit mitochondrial
P51146	Ras-related protein Rab-4b
D3ZIJ5	Dolichyl-phosphate mannosyltransferase subunit 3
D3ZK56	Ras-related protein Rap-2c
Q07984	Translocon-associated protein subunit delta
P31977	Ezrin
P15999	ATP synthase subunit alpha mitochondrial
M0RCA3	Zinc finger protein-like 1
Q6AYQ1	Golgin subfamily A member 7
P60711	Actin cytoplasmic 1
O35353	Transducin beta-4
Q9Z2S9	Flotillin-2
Q9ES21	Phosphatidylinositide phosphatase SAC1
Q9Z2L0	Voltage-dependent anion-selective channel protein 1
Q68FR6	Elongation factor 1-gamma
P25235	Dolichyl-diphosphooligosaccharide protein glycosyltransferase subunit 2
P51635	Alcohol dehydrogenase [NADP(+)]
F1LM82	Heterogeneous nuclear ribonucleoproteins A2/B1
B2RZD1	Protein transport protein Sec61 subunit beta
Q5PPL3	Sterol-4-alpha-carboxylate 3-dehydrogenase decarboxylating
P11951	Cytochrome c oxidase subunit 6C2
Q5XIM7	Lysine--tRNA ligase
P61107	Ras-related protein Rab-14
Q5XIM5	Protein CDV3 homolog

P20793	Serine/threonine-protein kinase MAK
F1LPV0	Asparagine--tRNA ligase, cytoplasmic
M0R9L0	Nascent polypeptide-associated complex subunit alpha
P49134	Integrin beta 1
G3V712	keratin 7
Q68FQ0	T-complex protein 1 epsilon
D4A781	Importin 5
G3V8Q1	Coatomer subunit epsilon
F1M790	Prostaglandin F2 receptor negative regulator
O35532	Methylsterol monooxygenase 1
F1LQ81	Vesicle-fusing ATPase
Q63269	Inositol 1,4,5-trisphosphate receptor type 3
D4A0T0	NADH dehydrogenase 1 beta subcomplex subunit 10
Q6AYF6	Glycosyltransferase 8 domain-containing protein 1
G3V6P8	Guanine nucleotide-binding protein gamma-12
G3V6P7	Myosin heavy chain 9
Q04970	GTPase NRas
Q9WUD2	Transient receptor potential cation channel subfamily V member 2 (TRPV2)
R9PXU4	Thioredoxin reductase 1 cytoplasmic
F1LM66	116 kDa U5 small nuclear ribonucleoprotein component
F1LSN4	Fibroblast growth factor receptor
D4ADE8	ATP-dependent RNA helicase DDX3X
O88600	Heat shock 70 kDa protein 4
Q794F9	4F2 cell-surface antigen heavy chain
F1LNT8	VAMP8
Q6P791	Ragulator complex protein LAMTOR1
F1M6W2	Endoplasmic reticulum metallopeptidase 1
Q6P799	Serine--tRNA ligase cytoplasmic
D4A0W1	ER membrane protein complex subunit 4
P62703	40S ribosomal protein S4, X isoform
P62909	40S ribosomal protein S3
O35796	Complement component 1 Q subcomponent-binding protein mitochondrial
Q6AYC4	Macrophage-capping protein
P62907	60S ribosomal protein L10a
P48721	Mortalin
P10960	Prosaposin
Q5XIM9	T-complex protein 1 beta
D4ADD7	Glutaredoxin 5
F1M779	Clathrin heavy chain
P05065	Fructose-bisphosphate aldolase A
P0C1Q3	Lysophosphatidylcholine acyltransferase 2
Q5XFX0	Transgelin-2
Q5BK32	FAS-associated factor 2
P50398	Rab GDP dissociation inhibitor alpha

P61805	Dolichyl-diphosphooligosaccharide protein glycosyltransferase subunit DAD1
P53987	Monocarboxylate transporter 1
G3V908	keratin, type II Kb15
Q5XIN6	Mitochondrial proton/calcium exchanger protein
P09811	Glycogen phosphorylase liver form
A2VD12	Pre-B-cell leukemia transcription factor-interacting protein 1
G3V6N2	Transmembrane emp24 domain-containing protein 4
P20788	Cytochrome b-c1 complex subunit Rieske
Q62902	Lectin mannose-binding 1
O88813	Long chain fatty acid CoA ligase 5
P28494	Alpha-mannosidase 2
P61131	Beta-galactoside alpha-2,3-sialyltransferase 4
Q6PDV6	40S ribosomal protein S14
P50399	Rab GDP dissociation inhibitor beta
Q6PDV7	60S ribosomal protein L10
Q6AYD3	Proliferation-associated protein 2G4
Q7TQ86	Ac1158
Q05175	Brain acid soluble protein 1
M0R402	Protein disulfide-isomerase TMX3
P62278	40S ribosomal protein S13
D3ZIE9	Delta-1-pyrroline-5-carboxylate synthase
P62138	Serine/threonine-protein phosphatase PP1-alpha catalytic subunit
Q68FV6	Glycosylated lysosomal membrane protein
Q68FV1	Trimeric intracellular cation channel type B (TRIC-B)
P31399	ATP synthase subunit D mitochondrial
G3V8L7	Integrin alpha M
D4A720	Serine/arginine-rich splicing factor 7 isoform 2
Q62762	Casein kinase I isoform gamma-2
D4A4J3	Alpha-mannosidase 2x
Q62763	Casein kinase I isoform gamma-3
O35509	Ras-related protein Rab-11b
Q62761	Casein kinase I isoform gamma-1
D4A0Y9	Phosphatidylcholine translocator ABCB4
Q6AYI1	Probable ATP-dependent RNA helicase DDX5
Q62764	Y-box-binding protein 3
D4ABY2	Coatomer subunit gamma 2
F7EWC1	Vasodilator-stimulated phosphoprotein
Q66HA6	ADP-ribosylation factor-like protein 8B
G3V6T7	Protein disulfide-isomerase A4
P11598	Protein disulfide-isomerase A3
P62282	40S ribosomal protein S11
Q66HA4	Tax1-binding protein 1 homolog
G3V6T1	Coatomer subunit alpha
Q5XIP2	GPI-anchor transamidase

Q6P742	Proteolipid protein 2
P62914	60S ribosomal protein L11
P50339	Chymase 1
Q5M9I5	Cytochrome b-c1 complex subunit 6 mitochondrial
Q9Z122	Acyl-CoA 6-desaturase
Q5XIP9	Transmembrane protein 43 (Protein LUMA)
Q5XIP6	Flap endonuclease 1
Q9JID2	Guanine nucleotide-binding protein alpha-11
P11980	Pyruvate kinase PKM
Q9Z2J5	VAMP5
A3RLA8	Low affinity immunoglobulin gamma Fc region receptor II
P10719	ATP synthase subunit beta mitochondrial
Q6URK4	Heterogeneous nuclear ribonucleoprotein A3
G3V741	Solute carrier family 25 member 3
O54980	Synaptogyrin-2
Q6P6T6	Cathepsin D
Q68FX4	Hematopoietic cell-specific LYN substrate 1 (hcls1)
F1LQ92	Sel-1 homolog 1
F1LQJ7	Phosphoenolpyruvate carboxykinase [GTP], mitochondrial
Q9R1T1	Barrier-to-autointegration factor
D4AA91	Ceramide synthase 5
P22734	Catechol O-methyltransferase
Q63486	Ras-related GTP-binding protein A (Rag A)
Q63483	Ras-related protein Rab-38
F1LM93	Tyrosine-protein kinase Yes
Q9JKB7	Guanine deaminase
Q63083	Nucleobindin-1
Q63085	Cyclic GMP-inhibited phosphodiesterase B
D4A994	ER membrane protein complex subunit 1
Q63081	Protein disulfide-isomerase A6
P17425	Hydroxymethylglutaryl-CoA synthase cytoplasmic
Q62940	E3 ubiquitin-protein ligase NEDD4
P68255	14-3-3 protein theta
Q63803	Guanine nucleotide-binding protein G(s) subunit alpha isoforms XLas
Q6P6U0	Tyrosine-protein kinase Fgr
F1M2K3	Small ubiquitin-related modifier
Q62753	Syntaxin-binding protein 2
P00388	NADPH--cytochrome P450 reductase
Q06647	ATP synthase subunit O mitochondrial
G3V8I4	Syntaxin-4A
Q68FY0	Cytochrome b-c1 complex subunit 1 mitochondrial
Q63507	60S ribosomal protein L14
D3ZNS8	Aladin
G3V679	Transferrin receptor protein 1

P29995	Inositol 1,4,5-trisphosphate receptor type 2
B2GUZ9	Fam49b protein
Q9Z0V6	Thioredoxin-dependent peroxide reductase mitochondrial
D3ZSA9	Nodal modulator 1
D3ZWS0	Protein scribble homolog
P62963	Profilin-1
Q66HD0	Endoplasmin
G3V8G5	Golgi apparatus protein 1
Q09326	Alpha-1,6-mannosyl-glycoprotein 2-beta-N-acetylglucosaminyltransferase
P62775	Myotrophin
Q6P6Q2	Keratin, type II cytoskeletal 5
D3ZNQ6	NEDD8-conjugating enzyme Ubc12
Q3B8Q2	Eukaryotic initiation factor 4A-III
Q9JLT0	Myosin heavy chain 10
Q9JM53	Apoptosis-inducing factor 1
P42667	SEC11 homolog A
G3V8F5	Mitochondrial import receptor subunit TOM40 homolog
Q5RKH6	Protein OS-9
P13383	Nucleolin
P13386	High affinity immunoglobulin epsilon receptor subunit beta
Q66X93	Staphylococcal nuclease domain-containing protein 1
P62986	Ubiquitin-60S ribosomal protein L40
Q6RUV5	Ras-related C3 botulinum toxin substrate 1 (Rac1)
P62982	Ubiquitin-40S ribosomal protein S27a
P82471	Guanine nucleotide-binding protein G(q) subunit alpha
Q9EPH8	Polyadenylate-binding protein 1
P19944	60S acidic ribosomal protein P1
P19945	60S acidic ribosomal protein P0
P11240	Cytochrome c oxidase subunit 5A mitochondrial
P11232	Thioredoxin
P10252	CD48 antigen
Q3KRE0	ATPase family AAA domain-containing protein 3
P62329	Thymosin beta-4
Q3KRE8	Tubulin beta-2B
P10860	Glutamate dehydrogenase 1
D3ZGV2	Peroxisomal membrane protein PEX16
B2GUV5	V-type proton ATPase subunit G 1
B2GV71	NADH dehydrogenase [ubiquinone] 1 alpha subcomplex assembly factor 5
B2GV72	Carbonyl reductase 3
P18418	Calreticulin
Q6AYA5	Transmembrane protein 106 B
A7E3N2	Neutrophil cytosol factor 2
Q3KRD8	Eukaryotic translation initiation factor 6
F1LVC3	Ras-related protein Rab-6b

G3V8C4	Chloride intracellular channel protein 4
Q497A2	Solute carrier family 35 member B2
P50137	Transketolase
P09527	Ras-related protein Rab-7a
P29419	ATP synthase subunit E mitochondrial
P10888	Cytochrome c oxidase subunit 4 isoform 1 mitochondrial
Q66HG5	Transmembrane 9 superfamily member 2
Q5U3Z7	Serine hydroxymethyltransferase
Q63524	Transmembrane emp24 domain-containing protein 2
Q66HG9	Mitochondrial antiviral-signaling protein
P62747	Rho-related GTP-binding protein RhoB
B2GV92	Prostaglandin E synthase 3
Q4AEF8	Coatomer subunit gamma 1
B2GUX5	5'-nucleotidase
P62083	40S ribosomal protein S7
P20171	GTPase HRas
B2GV96	Coiled-coil domain containing 115
Q66HF2	Transmembrane 9 superfamily member 1
Q66HF1	NADH-ubiquinone oxidoreductase 75 kDa subunit mitochondrial
Q5BQE6	Delta(24)-sterol reductase
G3V8A5	Vacuolar protein sorting-associated protein 35
Q5U1Y2	Ras-related C3 botulinum toxin substrate 2 (Rac2)
P85007	Immediate early response 3-interacting protein 1
Q5EB77	Ras-related protein Rab-18
D3ZIP8	Endonuclease domain containing 1
D4ADS9	EFR3 homolog A
B2GVB9	Fermt3 protein
F1M391	Stimulator of interferon genes protein
P35355	Prostaglandin G/H synthase 2
Q5U1X1	Oligoribonuclease mitochondrial
Q6AXT5	Ras-related protein Rab-21
D3ZJ32	Extended synaptotagmin 2
D4A376	Ras-related protein Rab-12
P19511	ATP synthase F(0) complex subunit B1 mitochondrial
D3ZPR0	Exportin-2
Q76GL9	Neutral amino acid transporter A
F1LNF0	Myosin heavy chain 14
Q4KM73	UMP-CMP kinase
Q4KM74	Vesicle-trafficking protein SEC22b
P46462	Transitional endoplasmic reticulum ATPase
P35171	Cytochrome c oxidase subunit 7A2
P04961	Proliferating cell nuclear antigen
D3ZD11	Signal peptidase complex subunit 2
Q3KRC3	Signal recognition particle receptor alpha

Q6AXS5	Plasminogen activator inhibitor 1 RNA-binding protein
Q6AXS4	Renin receptor
D4A4T0	STIP1 homology and U box-containing protein 1
D4A0E2	NSF attachment protein gamma
P16617	Phosphoglycerate kinase 1
P45592	Cofilin-1
D3ZF12	Signal peptidase complex subunit 3
F1MAS0	keratin 32
P05369	Farnesyl pyrophosphate synthase
Q5FVQ4	Malectin
D3ZD09	Cytochrome c oxidase subunit 6B1
O35142	Coatomer subunit beta 2
D3ZF11	Ragulator complex protein LAMTOR5
P08753	Guanine nucleotide-binding protein G(k) subunit alpha
D3ZPP2	ADP-ribosylation factor-like protein 8A
O35152	BET1-like protein
D4A0G7	Ras-related protein Rab-37
D4A9L2	Serine/arginine-rich splicing factor 1
P35427	60S ribosomal protein L13a
Q5RKI8	ATP-binding cassette sub-family B member 8
F1LPK9	Phosphatidylinositol glycan anchor biosynthesis class U protein
P08461	Pyruvate dehydrogenase component E2
D4ABI7	Very-long-chain (3R)-3-hydroxyacyl-CoA dehydratase
Q66HR2	Microtubule-associated protein RP/EB family member 1
F1LNC4	Vesicle transport through interaction with t-SNAREs homolog 1B
Q9Z2Z8	7-dehydrocholesterol reductase
G3V6D3	ATP synthase subunit beta
Q6AXX5	Retinol dehydrogenase 11
P35435	ATP synthase subunit gamma mitochondrial
D3ZGM7	Peptidase inhibitor 16
Q5RKJ9	Ras-related protein Rab-10
G3V6E1	Myosin heavy chain 2
Q5FVL2	ER membrane protein complex subunit 8
Q07647	Solute carrier family 2, facilitated glucose transporter member 3
Q499R8	Heterogeneous nuclear ribonucleoprotein H
O70601	Linker for activation of T-cells family member 1 (LAT)
P14659	Heat shock-related 70 kDa protein 2
P00507	Aspartate aminotransferase mitochondrial
P84100	60S ribosomal protein L19
Q3MHS9	Chaperonin-containing TCP1 subunit 6A
M0RC99	Ras-related protein Rab-5a
P84817	Mitochondrial fission 1 protein
Q9JK11	Reticulon-4
P23711	Heme oxygenase 2

Q6AXV4	Sorting and assembly machinery component 50 homolog
P14668	Annexin A5
Q5U1Z9	Metaxin 2
Q9Z1E1	Flotillin-1
Q4G075	Leukocyte elastase inhibitor
Q5FVN0	Lysophospholipid acyltransferase 5
P24368	Peptidyl-prolyl cis-trans isomerase B
P19234	NADH dehydrogenase [ubiquinone] flavoprotein 2 mitochondrial
Q499P3	C1GALT1-specific chaperone 1
P23514	Coatomer subunit beta 1
Q99JC6	Tapasin

ID Term	GO Term	Fold Enriched	Count	*p* -value	FDR
**GO:0007010**	**CYTOSKELETON ORGANIZATION**	**1.63**	**93**	**2.94E-6**	**5.7E-3**
**Annotated proteins**					
**Protein Accession**			**Protein Identification**		
P63322			Ras-related protein Ral-A		
Q5U1Y2			Ras-related C3 botulinum toxin substrate 2 (Rac2)		
Q62839			Golgin subfamily A member 2		
Q505J9			ATPase family AAA domain-containing protein 1		
B2RZ27			SH3 domain-binding glutamic acid-rich-like protein 3		
Q3T1K5			F-actin-capping protein subunit alpha-2		
Q00238			Intercellular adhesion molecule 1 (ICAM1)		
P68370			Tubulin alpha-1A		
Q05030			Platelet-derived growth factor receptor beta		
G3V852			Talin-1		
D4ABM3			Dishevelled associated activator of morphogenesis 1		
P13084			Nucleophosmin		
P62329			Thymosin beta-4		
P69897			Tubulin beta-5		
D4A7U1			Zyxin		
Q66HR2			Microtubule-associated protein RP/EB family member 1		
P62828			GTP-binding nuclear protein Ran		
P47819			Glial fibrillary acidic protein		
Q3KRE8			Tubulin beta-2B		
P49134			Integrin beta 1		
P85108			Tubulin beta-2A		
P09456			cAMP-dependent protein kinase type I-alpha regulatory subunit		
Q91ZN1			Coronin-1A		
Q6IG02			Keratin, type II cytoskeletal 2 epidermal		
P52296			Importin subunit beta-1		
D3ZTJ0			Protein XRP2		

Q4QRB4	Tubulin beta-3
P07632	Superoxide dismutase [Cu-Zn]
Q6IG00	Keratin, type II cytoskeletal 4
Q3SWT0	Platelet endothelial cell adhesion molecule (Pecam1)
Q63116	Mast/stem cell growth factor receptor
Q08163	Adenylyl cyclase-associated protein 1
Q6P9V9	Tubulin alpha-1B
F7EWC1	Vasodilator-stimulated phosphoprotein
Q6AY56	Tubulin alpha-8
Q6IFW6	Keratin, type I cytoskeletal 10
Q5XIF6	Tubulin alpha-4A
P18418	Calreticulin
P09495	Tropomyosin alpha-4
P31000	Vimentin
P62963	Profilin-1
Q4QQV0	Tubulin beta-6
P61589	Transforming protein RhoA
P20417	Protein-tyrosine phosphatase 1B
F1LNF0	Myosin heavy chain 14
G3V6E1	Myosin heavy chain 2
Q66HD0	Endoplasmin
G3V6P7	Myosin heavy chain 9
P08644	GTPase KRas
Q5BJY9	Keratin, type I cytoskeletal 18
Q5M860	Rho GDP dissociation inhibitor 2
Q32PX6	Rho-related GTP-binding protein RhoG
P04797	Glyceraldehyde-3-phosphate dehydrogenase
D3ZAY7	Band 4.1-like protein 2
F1LRV9	Myosin heavy chain 1
Q8CFN2	Cell division control protein 42 homolog (CDC42)
P62775	Myotrophin
P45592	Cofilin-1
Q6S395	Plectin
Q78P65	Rap2A-like protein
Q68FX4	Hematopoietic cell-specific LYN substrate 1 (hcls1)
P01830	Thy-1 membrane glycoprotein
P62909	40S ribosomal protein S3
M0RCF3	Integrin beta 3
Q68FP1	Gelsolin
Q4V8C2	Centromere/kinetochore protein zw10 homolog
Q9JJ19	Na(+)/H(+) exchange regulatory cofactor NHE-RF1
Q6AYC4	Macrophage-capping protein
Q5XI34	Serine/threonine-protein phosphatase 2A 65 kDa regulatory subunit A alpha isoform
Q5XI32	F-actin-capping protein subunit beta

Q9JLT0	Myosin heavy chain 10
Q6AYZ1	Tubulin alpha-1C
Q9JKB7	Guanine deaminase
P20171	GTPase HRas
P31977	Ezrin
M0RDJ4	Glia maturation factor beta
Q5XI38	Lymphocyte cytosolic protein 1
Q6IFU9	keratin 16
Q6P9T8	Tubulin beta-4B
Q5XI73	Rho GDP dissociation inhibitor 1
Q6IFU8	Keratin, type I cytoskeletal 17
Q63610	Tropomyosin alpha-3
P07150	Annexin A1
Q4KM97	Tyrosine-protein kinase BLK
Q10758	Keratin, type II cytoskeletal 8
Q6IFV3	Keratin, type I cytoskeletal 15
G3V908	keratin, type II Kb15
P53987	Monocarboxylate transporter 1
Q6IFV1	Keratin, type I cytoskeletal 14
Q6IFZ5	Keratin, type II cytoskeletal 2 oral
Q6RUV5	Ras-related C3 botulinum toxin substrate 1 (Rac1)
E9PT65	Radixin
B4F7C2	Tubulin beta-4A

ID Term	GO Term	Fold Enriched	Count	*p* -value	FDR
**GO:0022610**	**BIOLOGICAL ADHESION**	**1.63**	**122**	**7.33E-8**	**1.43E-4**

Annotated proteins

Protein Accession	Protein Identification
M0RD75	40S ribosomal protein S6
Q68FR6	Elongation factor 1-gamma
Q5U1Y2	Ras-related C3 botulinum toxin substrate 2 (Rac2)
F1M2P8	Protein kinase C alpha
Q6IN24	Galectin-8
G3V8I4	Syntaxin-4A
Q00238	Intercellular adhesion molecule 1 (ICAM1)
G3V852	Talin-1
D4A7U1	Zyxin
G3V679	Transferrin receptor protein 1
Q6PCU4	Emerin
B2GVB9	Fermt3 protein
Q4FZT0	Stomatin-like protein 2
P49134	Integrin beta 1

P97546	Neuroplastin
P62260	14-3-3 protein epsilon
Q641Y0	Oligosaccharyl transferase 48 kDa subunit
Q5XI52	Vezatin
Q62733	Lamina-associated polypeptide 2 isoform beta
Q3SWT0	Platelet endothelial cell adhesion molecule (Pecam1)
Q6NYB7	Ras-related protein Rab-1a
D3ZJ32	Extended synaptotagmin 2
D3ZWS0	Protein scribble homolog
P62963	Profilin-1
P61589	Transforming protein RhoA
P20417	Protein-tyrosine phosphatase 1B
G3V7M2	Integrin beta 7
Q9Z269	VAMP-associated protein B
G3V6P7	Myosin heavy chain 9
P20411	High affinity immunoglobulin epsilon receptor subunit gamma
Q91Y81	Septin-2
Q5BJY9	Keratin, type I cytoskeletal 18
P35704	Peroxiredoxin-2
P11762	Galectin-1
Q6AXS5	Plasminogen activator inhibitor 1 RNA-binding protein
D4ADE8	ATP-dependent RNA helicase DDX3X
P06761	Endoplasmic reticulum chaperone BiP
Q9QYV0	Disintegrin and metalloproteinase domain-containing protein 15
P63102	14-3-3 protein zeta/delta
Q01621	Tyrosine-protein kinase LCK
F1LZX9	Integrin alpha 5
P61227	Ras-related protein Rap-2b
O35796	Complement component 1 Q subcomponent-binding protein mitochondrial
M0RCF3	Integrin beta 3
Q6AYC4	Macrophage-capping protein
Q1WIM3	Cell adhesion molecule 3
P97887	Presenilin-1
Q9JLT0	Myosin heavy chain 10
D3ZWZ1	Integrin alpha X
B2RYF6	Cleft lip and palate associated transmembrane protein 1
Q5XI38	Lymphocyte cytosolic protein 1
Q5XFX0	Transgelin-2
G3V667	Integrin alpha 6
Q66X93	Staphylococcal nuclease domain-containing protein 1
D4AAL4	Adhesion G protein-coupled receptor L3
Q6RUV5	Ras-related C3 botulinum toxin substrate 1 (Rac1)
Q07936	Annexin A2
P05197	Elongation factor 2

E9PT65	Radixin
Q6MG61	Chloride intracellular channel protein 1
O88767	Protein/nucleic acid deglycase DJ-1
G3V8L7	Integrin alpha M
P10252	CD48 antigen
P04642	L-lactate dehydrogenase A chain
P35213	14-3-3 protein beta/alpha
O35509	Ras-related protein Rab-11b
Q66HR2	Microtubule-associated protein RP/EB family member 1
P34058	Heat shock protein HSP 90-beta
Q9QYE7	Integrin alpha D
G3V6D3	ATP synthase subunit beta
O08775	Vascular endothelial growth factor receptor 2
Q920P6	Adenosine deaminase
P09456	cAMP-dependent protein kinase type I-alpha regulatory subunit
Q91ZN1	Coronin-1A
P63095	Guanine nucleotide-binding protein G(s) subunit alpha
G3V7Q7	Ras GTPase-activating-like protein IQGAP1
Q63116	Mast/stem cell growth factor receptor
P26453	Basigin
F7EWC1	Vasodilator-stimulated phosphoprotein
Q5RKJ9	Ras-related protein Rab-10
P18418	Calreticulin
G3V964	Neurotrimin
O88777	Presenilin-2
P38983	40S ribosomal protein SA
Q63198	Contactin 1
P11980	Pyruvate kinase PKM
P63039	60 kDa heat shock protein
Q8CGU6	Nicastrin
O70601	Linker for activation of T-cells family member 1 (LAT)
P10719	ATP synthase subunit beta mitochondrial
O35567	Bifunctional purine biosynthesis protein PURH
Q0R3W9	GTPase, IMAP family member 1
Q08849	Syntaxin-3
Q8CFN2	Cell division control protein 42 homolog (CDC42)
F6T9E7	P-selectin glycoprotein ligand 1
Q5I0D1	Glyoxalase domain-containing protein 4
Q6S395	Plectin
Q08851	Syntaxin-5
Q9JK11	Reticulon-4
P01830	Thy-1 membrane glycoprotein
Q68FP1	Gelsolin
P23640	Ras-related protein Rab-27a

Q91ZQ0	Vacuole membrane protein 1
Q07014	Tyrosine-protein kinase Lyn
P62747	Rho-related GTP-binding protein RhoB
M0RAU0	Advanced glycosylation end product-specific receptor
P31977	Ezrin
Q63085	Cyclic GMP-inhibited phosphodiesterase B
Q9Z1E1	Flotillin-1
P63025	VAMP3
Q63081	Protein disulfide-isomerase A6
P07151	Beta-2-microglobulin
P13832	Myosin regulatory light chain RLC-A
P04157	Receptor-type tyrosine-protein phosphatase C (CD45)
P07150	Annexin A1
Q62940	E3 ubiquitin-protein ligase NEDD4
Q62658	Peptidyl-prolyl cis-trans isomerase FKBP1A
P60711	Actin cytoplasmic 1
B2RYB8	Integrin beta 2
Q63803	Guanine nucleotide-binding protein G(s) subunit alpha isoforms XLas
Q9Z2S9	Flotillin-2
P13838	Leukosialin
F1LVV4	Protein RCC2
